# Descriptions and Experiences with Medical Assistance in Dying Models Across Canada: A Mixed Methods Study

**DOI:** 10.3390/healthcare14060797

**Published:** 2026-03-20

**Authors:** Tania Stafinski, Christina Rumsey, Devidas Menon, Clinton Ekaeze

**Affiliations:** School of Public Health, Dianne and Irving Kipnes Health Research Academy, University of Alberta, Edmonton, AB T6G 1C9, Canada; tanias@ualberta.ca (T.S.); menon@ualberta.ca (D.M.); ekaeze@ualberta.ca (C.E.)

**Keywords:** medical assistance in dying, Canada, service and delivery, oversight, assessment, bereavement, training, safeguards, qualitative research, quantitative research

## Abstract

**Background:** Medical Assistance in Dying (MAiD) was first legalized in Canada in 2016, with legislation expanding from foreseeable to non-foreseeable natural deaths. A sole underlying medical condition of mental illness is expected to be added in 2027. Although legislation and reporting requirements are federally mandated, the implementation and delivery of MAiD are the responsibility of individual provinces and territories. **Objectives:** The aim of this study is to compare the organization, delivery, and oversight of MAiD programs across provinces and territories in consideration of access, equity, and safeguards. **Methods**: This study used a mixed methods approach to collect data. A comprehensive and systematic search for published peer reviewed literature on MAiD programs in Canada was conducted along with qualitative interviews with key informants using purposive and snowball sampling. A qualitative descriptive design was used for qualitative data, including content analysis. To facilitate a detailed comparative analysis of MAiD across jurisdictions, separate tables were created for each component or element, organizing the results of the literature review and qualitative analysis by jurisdiction. Patterns within these tables were identified through qualitative interpretation. The findings were then summarized in a narrative format. **Results**: A total of 113 interviews were conducted, representing all provinces and territories but Nunavut. Findings showed varied practices throughout the MAiD process between jurisdictions. **Conclusions**: The main findings of this study are that the organization of MAiD programs, oversight, reporting methods to Health Canada, intake, preliminary assessments, assessments, provision, and bereavement support vary. In addition, specific policies related to potentially vulnerable populations are lacking and jurisdictional practices also vary. Centralized, multidisciplinary MAiD programs with strong oversight mechanisms may strengthen issues related to access, equity, and safeguards.

## 1. Introduction

In December of 2015, the Act Respecting End-of-Life Care came into force in Québec, Canada, making it the first Canadian province/territory to create legislation on medical assistance in dying (MAiD) [1]. MAiD then became legal throughout Canada with the passage of Bill C-14 in June of 2016, allowing eligible individuals whose natural death was foreseeable (Track 1) to exercise greater autonomy over their end-of-life care [2]. This federal legislation established eligibility criteria, procedural safeguards, and reporting requirements for the purpose of monitoring to ensure transparency and accountability [2,3]. Nearly five years later, in March of 2021, Bill C-7 was passed, enhancing reporting requirements and expanding MAiD eligibility to include individuals whose natural death is not reasonably foreseeable (Track 2) [4]. Eligibility for persons where mental illness is the sole underlying medical condition (MI-SUMC) has been postponed until March of 2027 [5]. While eligibility, safeguards, and reporting requirements are federally mandated, the implementation of MAiD is determined by individual provinces and territories. Consequently, the delivery of MAiD varies across Canada [6].

This study is unique in offering a pan-Canadian comparison of MAiD service and delivery. Canada is amongst a number of countries that have legalized both assisted dying (clinician prescribed medication for self-administration) and voluntary euthanasia (clinician-administered medication) including the Netherlands, Belgium, New Zealand, Luxembourg, and parts of Australia for example [7]. Other countries, such as Switzerland and parts of the United States, have legalized assisted dying only [7]. Studies comparing international programs include identifying the underlying disease amongst MAiD recipients and a general overview of procedural requirements and safeguards [7,8]. However, the extent and depth to which the literature describes policies and processes for initiating MAiD requests through to the provision and bereavement support is limited. In our view such information is critical in understanding what may be needed to improve the delivery of MAiD services in any jurisdiction.

Previous research on MAiD services in Canada has offered a broad understanding of the roles and challenges of healthcare professionals involved [9]. Role specificity within specific MAiD models of service delivery, however, is missing for comparison. Patient and family perspectives on challenges with access to MAiD, including unclear eligibility criteria; unsupportive healthcare providers, including conscientious objection; and limited guidance and information sharing is also evident in recent literature [10,11,12,13,14,15,16].

There has been ongoing debate regarding adequate safeguards especially for individuals most vulnerable to systemic oppression. As MAiD has expanded to Track 2 and Canada prepares for MI-SUMC, concerns have been raised regarding inadequate data collection to understand system barriers and social determinants of health as they relate to MAiD provisions; lack of oversight to ensure clear pathways for investigating concerns or complaints regarding potentially inappropriate provisions; unclear eligibility terminology in Canada’s MAiD legislation; and inadequate disability supports and palliative care that may result in requests and provisions of MAiD [17]. Previous research has highlighted a need for holistic, multidisciplinary, comprehensive MAiD service and delivery [9,10,11,12,13,14,15,16,18]. In addition, Serota et al. [10] recommend transparency, accountability, and ethics consultation for complex MAiD cases.

The objective of this study therefore is to compare the organization, delivery, and oversight of MAiD programs across provinces and territories. This includes operational policies and procedures guiding each step of the process, from initial inquiries by patients and families to provision and bereavement support. This comparison provides valuable considerations for improvements to MAiD models of service and delivery related to access, equity, and safeguards. International jurisdictions may also find this research useful in consideration of their own implementation of MAiD services and legislation.

## 2. Materials and Methods

This study draws on data collected from a larger Health Canada-funded project examining the implementation of MAiD service and delivery across the country. Phase 1.0 of the study comprised a jurisdictional scan using a mixed methods approach. This involved a comprehensive review of relevant literature and key informant interviews with policymakers, program administrators, and health professionals involved in MAiD across Canada.

The design reflects the expertise of the lead researchers in qualitative and quantitative methods for conducting health technology assessments and broader health evidence reviews. One of the four researchers had previous experience as a social worker in a hospice setting, providing support to patients who requested and received MAiD and their families. Their experience offered a general understanding of the MAiD process and the potential needs of patients and families from the initial request to post-provision within a specific jurisdiction.

### 2.1. Literature Review

A comprehensive and systematic search for published peer-reviewed literature on MAiD programs in Canada was conducted in collaboration with an experienced health information specialist/research librarian. The search followed internationally recognized methodological guidelines and was validated by an independent research librarian. The health information specialist developed and tested the search strategies through an iterative process in consultation with the review team. Using the multifile option and deduplication tool available on the Ovid platform, we searched Ovid MEDLINE^®^ ALL, Embase, and APA PsycInfo. We also searched CINAHL and Public Affairs Index on Ebsco, the Web of Science Core Collection, and PubMed. All searches were undertaken on 26 April 2023. The strategies utilized a combination of controlled vocabulary (e.g., “Suicide, Assisted”, “Canada”) and keywords (e.g., “assisted dying”, “MAiD”, “Alberta”). Vocabulary and syntax were adjusted across the databases, and where possible, animal-only records were removed from the results. Records were downloaded and deduplicated using EndNote version 9.3.3 (Clarivate Analytics, Philadelphia, PA, USA).

A comprehensive and systematic search for non-academic sources of information (grey literature), such as organizational reports, program manuals and procedural documents was also performed. Various keyword combinations, identified during the search for peer-reviewed literature, were applied to the Google^®^ search engine, and the first 50 ‘hits’ generated from each combination were reviewed. In addition, targeted searches were conducted on the websites of federal, provincial, and territorial ministries of health, regional health authorities, healthcare delivery organizations, and advocacy associations (see Supplementary Material S1 for the MAiD Services PRISMA Flow Diagram).

Relevant information from the searches was systematically collected using standardized data extraction tables for content analysis, which included key elements from jurisdictional MAiD programs relating to organizational structure, service delivery, and oversight. Categories for data extraction were informed by the research questions which asked about how MAiD services are delivered in jurisdictions including steps in the process, such as initiating a request, receiving a request, intake, assessment, provision, and post-provision bereavement support. The tables also captured details on the health workforce, including the composition and roles of MAiD program staff, their scopes of practice, training, education, and remuneration. It documented care coordination services, standards of practice, and other mechanisms for ensuring safe, high quality, person-centered care. Additionally, it included information on policies and programs for supporting vulnerable populations and individuals in rural or remote communities, as well as provincial and territorial processes for monitoring and reporting. For quality assurance, all searches were performed independently by two experienced researchers who then met to compare results and resolve any discrepancies. Further, all information from documents identified through the searches was extracted independently by two researchers who then met to compare their findings and resolve any discrepancies. The information collected was synthesized by program or jurisdiction, depending on the province or territory, and organized into tables to facilitate a qualitative comparative analysis.

### 2.2. Key Informant Interviews

Both purposive and snowball sampling were used to select participants. Interview participants were identified through the following approaches:

(1) Literature review: Potential participants were identified from names and contact information found in scholarly and grey literature related to MAiD in Canada.
(2) Federal/Provincial/Territorial Working Group on MAiD: Working Group members provided a list of names and contact information for individuals involved in MAiD policy development or program delivery within their jurisdiction.
(3) Referrals from other interview participants: Interviewees suggested other individuals and agreed to contact them. Those willing to participate then contacted the project team.
(4) The Canadian Association of MAiD Assessors and Providers: The lead for research was contacted and asked to share information about the review with members of the association.

Participants who responded to the request were sent an email describing the purpose of the study and expectations regarding the interview process (see Supplementary Material S2 for the MAiD Recruitment Letter and Supplementary Material S3 for the MAiD Consent Form). Separate interview guides were developed for participants involved in setting provincial or territorial government policies and those involved in the delivery of MAiD-related services (see Supplementary Material S4 for Interview Guide for MAiD Teams and Supplementary Material S5 for Interview Guide for MAiD Program Administrative and Clinical Team Members). Interview questions were pilot tested with two senior-level health executives who had relevant content expertise. Interviews were one-on-one, semi-structured using open-ended questions, conducted virtually, recorded on Zoom, and transcribed. Efforts were made to interview multiple individuals from within a program or jurisdiction to increase the likelihood of collecting accurate information. The number of interviews conducted was based on attempts to recruit participants from all provinces and territories and the need to ensure saturation was reached. ‘Saturation’ refers to the point when no new information, themes, or insights are emerging from additional interviews. When discrepancies were identified, relevant individuals were contacted in order to resolve them through discussion. Lastly, summaries of information collected for a program or jurisdiction were sent to individuals from that program or jurisdiction for their review. 

A qualitative descriptive design was used, including content analysis, to remain as close to the data as possible [19,20]. Interview transcripts were cleaned and analyzed and incorporated into the same tables as the literature review. Interview content was identified in the tables using participant codes beginning with the sequence ‘P00’, while the literature review content was identified using a numerical citation system with a corresponding reference list compiled from the reviewed reports. Combining findings from both methods (the interviews and the literature review) into a single comprehensive data set for each jurisdiction allowed for a detailed comparative analysis of MAiD across jurisdictions. Publicly available information on MAiD delivery processes within jurisdictions across Canada was limited. Therefore, the study relied on interviews as the primary data source. However, where information from both sources could be compared, the findings were consistent.

Patterns within these tables were identified through qualitative interpretation (see Supplementary Material S6 for MAiD Services Coding Framework). The findings were then summarized in a narrative format. The term jurisdiction was used to include all MAiD programs contacted, including provincial, territorial, regional, organizational, institutional, geographical areas, and communities of practice.

## 3. Results

**Figure 1:**
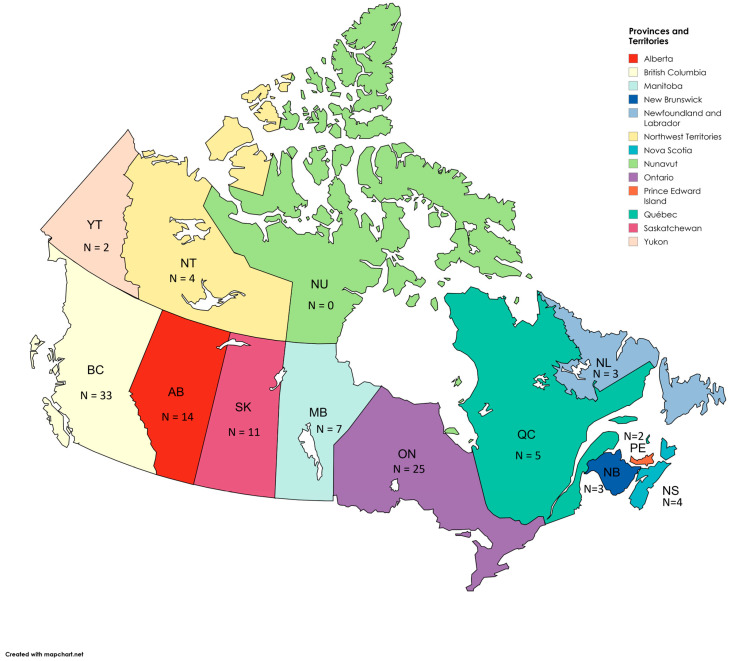
Visual map showing the breakdown of participants in each province or territory.

Limited information on the structure, organization and delivery of MAiD-related services was found from the literature review of published, peer-reviewed and grey literature. As a result, responses from key informant interviews comprised the main information source (see Supplementary Material S7 for MAiD Categorized Tables of Results). A total of 113 interviews were conducted, representing all provinces and territories but Nunavut. The number of participants interviewed in each province or territory was: 14 in Alberta, 33 in British Columbia, seven in Manitoba, three in New Brunswick, three in Newfoundland and Labrador, four in the Northwest Territories, four in Nova Scotia, 25 in Ontario, two in Prince Edward Island, five in Québec, 11 in Saskatchewan, and two in the Yukon Mapchart.net was used to generate Figure 1: Visual map showing the breakdown of participants in each province or territory. The interviewees held diverse roles, including government policymakers, legal advisors, MAiD program medical and administrative leads, care coordinators/navigators, social workers, registered nurses, and MAiD assessors and providers (physicians and nurse practitioners). Additionally, unregulated health professionals offering end-of-life support to patients, such as death doulas and spiritual care providers, were also interviewed.

### 3.1. Organization of MAiD Programs

MAiD programs are situated within provincial and territorial (P/T) departments, health authorities (HAs), regional health authorities (RHAs), or zones. Most have MAiD teams which can include clinical professionals and operational/administrative staff (see Appendix A for Table A1: Possible MAiD team members).

Jurisdictions have implemented centralized, decentralized, or hybrid models of MAiD programs and service delivery (see Appendix B for Table A2: Jurisdiction organization). Centralized models involve access and coordination of referrals and services at the P/T or regional level, depending on how the delivery of healthcare is organized. Decentralized models have no single point of access to MAiD services. Referrals, assessments, and provisions are neither standardized nor coordinated at a regional or P/T health authority/department/ministerial level. Instead, individual organizations, institutions, and practitioners within a region or geographical area have established their own processes. A hybrid model includes both a centralized coordination service and individual practitioners coordinating referrals, assessments, and provisions. The organizational structure of MAiD programs and services varies across provinces and territories. MAiD programs may be situated within P/T departments, HAs, or RHAs/zones. In British Columbia, the RHAs and the Provincial Health Services Authority ensure the delivery of MAiD services specific to their geographical areas or programs. In Manitoba, the provincial health authority, Shared Health, holds this responsibility for all of the RHAs. Ontario’s organizational structure includes a provincial care coordination program, regional programs, service organizations, regional facilities, and community practices, all providing MAiD service and delivery.

### 3.2. Remuneration

Typically, physicians completing assessments and provisions are remunerated based on MAiD-specific P/T billing codes, although a small number provide services as part of their salaried position or contract with a jurisdiction. Nurse practitioners (NPs) completing assessments and provisions hold contract positions in the organization with operational oversight of the MAiD program. In some jurisdictions, arrangements are in place to compensate NPs for additional hours worked in the evenings or on weekends to accommodate patient and family preferences. Travel compensation to rural areas for provisions varies across jurisdictions and between physicians and NPs. For example, in Alberta, physician mileage is compensated by the Ministry of Health, and NP travel is compensated by the provincial health authority. In British Columbia, the Ministry of Health provides funding for health authorities to cover the costs associated with MAiD provision under the Medical Assistance in Dying Travel and Training Assistance Program. This funding compensates physicians for travel time and related costs, including meals, lodging, and travel. Individual RHAs determine compensation for NPs travelling to provide MAiD. In Manitoba, the Ministry of Health funds physicians to travel to rural communities, and if the location is more than three hours away, they are flown there. To date, NPs have not been involved in travel. Similarly, in Québec, New Brunswick, and Nova Scotia, the Ministries of Health fund travel for physicians to rural communities. In the Northwest Territories, Yukon, and Ontario, the Ministries/Departments of Health fund travel for both physicians and NPs. However, in Ontario, travel by air, train, or ferry is not reimbursed. In both Prince Edward Island and Saskatchewan, the Ministries of Health fund travel for physicians, while HAs are responsible for remunerating mileage for NPs, since they are in salaried positions.

### 3.3. Program Oversight

Approaches to program oversight have comprised the creation of oversight committees. In general, provinces and territories (P/Ts) have structured these committees in one of two ways: (1) as independent or external to the MAiD program or (2) as a part of the MAiD program itself. In general, where a single P/T health authority/department exists, an independent committee also exists. Among provinces with RHAs, about half have convened independent committees at the provincial level, in addition to committees/teams at the regional or institutional level which are connected to MAiD programs.

While the membership of oversight committees varies, it is always multi-disciplinary and multi-sectoral. In some P/Ts, it includes representatives from the ministries/departments of health, RHAs, professional regulatory colleges, and/or organizations under which the MAiD program falls. The range of professional disciplines represented spans pharmacists, nurses, NPs, social workers, ethicists, legal counsel, and spiritual care providers. In several provinces, the oversight committee includes patient/family partners. In Ontario and the Yukon, there is representation from the First Nations and disability advocacy community, respectively.

The role of the oversight committee varies. At the P/T/ministerial level, it can include addressing specific policy issues and developing recommendations, drafting strategic policy and standards, ensuring compliance with safeguards and processes, and reviewing select individual case files, which typically consist of cases involving concerns around eligibility for MAiD. At the regional or organizational level, it generally includes developing operational standards, policies, and processes, directly offering guidance to and resources for staff, assessors, and providers, and ensuring alignment of the delivery of MAiD services with policy directions. In some jurisdictions, the oversight committee reviews all provision documentation for accuracy, completeness, and compliance with legislation, regulations, and standards. In other jurisdictions, the committee also reviews all documentation for accuracy and completeness for reporting purposes, but not to confirm appropriateness of the provision. In some P/Ts there are additional processes in place. For example, in Alberta and PEI, the oversight committee reviews select individual case files, which typically consist of cases involving concerns around eligibility for MAiD. In Ontario, the Office of the Chief Coroner reviews all cases after provision and submission of documentation by providers and follows up with them when clarification or education around federal legislation and regulations is needed. Similarly, Québec’s oversight committee, the Commission on End-of-Life Care, conducts bi-weekly reviews of all cases after provision. Finally, the New Brunswick Vitalité Health Network oversight committee reviews all cases before the provision of MAiD.

### 3.4. Reporting

P/Ts complete mandatory standardized reporting to Health Canada. In about half, assessors, providers, and pharmacists report assessments, provisions, and medications themselves. In the other half, reporting is completed on their behalf through delegated reporting processes (see Appendix C for Table A3: Reporting process). Specifically, the centralized MAiD coordination service or the ministries of health collect all required documentation from assessors, providers, and pharmacists and submit it to Health Canada. In Ontario, it is the responsibility of the Office of the Chief Coroner to submit all documentation related to provisions to Health Canada. The extent to which information on preliminary assessments is reported to Health Canada varies. Briefly, preliminary assessments are conducted to determine whether a person expressing interest in or requesting MAiD meets basic eligibility criteria (e.g., at least 18 years of age and is covered through P/T health insurance). In several P/Ts, they comprise the first step in the MAiD process and may be conducted and reported to Health Canada by a range of different individuals. In other provinces, preliminary assessments do not take place because they are considered duplicative (i.e., eligibility is already determined through the formal MAiD assessment).

In addition to mandatory reporting to Health Canada, two provinces have created provincial mechanisms for generating monthly and quarterly reports of MAiD-related statistics. In Ontario and Québec, these are prepared by the Office of the Chief Coroner and Commission on End-of-Life Care, respectively.

### 3.5. Translation and Interpretive Services

All MAiD programs have access to translation and interpretive services, but the range of communication methods available (by telephone, virtually, or in person), source of these services, and training requirements vary. In most jurisdictions, MAiD programs reach out to formal translation services that exist at the P/T, regional, and/or institutional level to support all healthcare services. While some jurisdictions rely on informal translation by family or friends, particularly in geographical areas where centralized systems do not exist, others do not, unless there is no other option. British Columbia Fraser Health requires those providing translation services to complete MAiD-specific training.

### 3.6. Intake and Preliminary Assessment

Centralized intake systems involve a single pathway for requests and referrals that leads to a dedicated MAiD coordination service at a regional or P/T level. Decentralized intake systems consist of several pathways for requesting MAiD within a jurisdiction, which operate at regional, institutional, and individual provider levels within a single jurisdiction. In general, it is possible for individuals to request MAiD services from individual physicians (bypassing any formal coordination service). Where there are centralized intake systems, intake is usually completed by a nurse coordinator, either virtually or in person, using a standardized form. This form may contain fields for all of the necessary information and voluntary sociodemographic data requested by Health Canada. Across jurisdictions, the intake process consists of some or all of the following elements: screening, gathering patient information and consent, communicating with patients and families, triaging patients for assessment, reviewing documentation, and providing educational sessions. In some jurisdictions, this process also helps to identify patients and families who could benefit from specific support services and connects them to those services.

### 3.7. Assessment

In all jurisdictions, assessments (and provisions) can be conducted by physicians and NPs who meet minimal eligibility requirements (professional credentials). However, in most, processes for determining their suitability (whether they have the requisite skills, comfort, and capacity to conduct MAiD assessments) and training have been established. Privileging is managed at the facility or regional/provincial/territorial health authority/department level or by the MAiD medical director or hospital chief of staff. Training typically includes mentorship, shadowing, and online modules, but whether it is mandatory or voluntary varies.

As mandated in federal legislation, two independent assessments are required for the determination of a patient’s eligibility for MAiD. In most jurisdictions with centralized intake systems, the MAiD program coordinates and assigns first and second assessors. However, where individual physicians or NPs receive the initial MAiD request, they typically manage the assignment of assessors themselves. In general, assessments can be completed virtually or in person. Only a small number of jurisdictions stipulate that they must be conducted in person. Most jurisdictions use provincially, regionally, or territorially created standardized MAiD assessment forms. What constitutes an ‘independent’ assessment varies. In some jurisdictions, contact between assessors is not permitted; therefore, the second assessor is unaware of (blinded to) the outcomes of the first assessment. However, there are exceptional circumstances under which discussions can take place. For example, simultaneous assessments may occur when one of the assessors is a specialist or a patient is quickly approaching end-of-life. In other jurisdictions, the second assessor may communicate with the first assessor prior to determining eligibility, given that the second assessor is still making the decision on their own. Some jurisdictions have implemented additional safeguards for ensuring independence of assessments (e.g., not storing assessment findings in the patient’s electronic medical record or requiring that assessments be conducted at different times). In some cases, a specialist assessment may be warranted to explore treatment options or resolve concerns over suicidality.

Most jurisdictions have established processes for managing disagreements between assessors. Typically, they involve a third assessment if disagreement persists after assessors have met to discuss the case. Disagreements often stem from variation in the interpretation of the terms ‘reasonably foreseeable’ (Track 1) and ‘non-reasonably foreseeable’ natural death (Track 2). Federal legislation does not include an explicit definition of either term for the purposes of determining MAiD eligibility. Therefore, jurisdictions use different guidelines. They include those issued by the Canadian Medical Protective Association, the P/T colleges of physicians and surgeons and the Commission on End-of-Life Care in Québec. Though not formally endorsed by jurisdictions, many assessors rely on the guidelines developed by the Canadian Association of MAiD Assessors and Providers (CAMAP). While the interpretation of ‘foreseeability’ varies and often depends on the assessor’s experience, there is a consistent focus on the trajectory towards death and the patient’s vulnerability or fragility. Various timeframes for describing death as ‘foreseeable’ have been used, including the “not too distant future”, “days to weeks”, “weeks to months”, “up to 6 months”, “6–12 months”, “up to 18 months”, “up to 2 years”, and “5–10 years”. Some jurisdictions require a specialist to interpret ‘reasonably foreseeable death’, explore available treatment options, or ensure all efforts have been taken to reverse an acute failure.

Most jurisdictions who participated in this study had limited or no experience with ‘non-reasonably foreseeable natural deaths’. Nonetheless, approaches to interpreting a ‘non-reasonably foreseeable natural death’ include consideration of how the 90-day assessment period (often referred to by jurisdictions as the ’90 day waiting period’) is calculated, how assessors determine suffering, what timeframe constitutes ‘non-reasonably foreseeable’, and whether assessors are required to explore alternative treatment options with patients. In most jurisdictions, this period is calculated based on the date of the first assessment. In all jurisdictions, the 90 days may be reduced if the patient is at risk of losing capacity. Typically, this is confirmed when both assessors are in agreement. Almost all jurisdictions rely on consultations with specialists to determine whether a patient has been offered all reasonable and available means to relieve suffering.

In several jurisdictions, multiple follow-up assessments are conducted to identify changes in a patient’s health status and prognosis over time. Over the follow-up period, their status may be reclassified as ‘reasonably foreseeable natural death’. Often, this decision is made in consideration of criterion 4 of the CAMAP guidelines on “The interpretation and role of “reasonably foreseeable” in the practice of medical assistance in dying” [21].

### 3.8. Provision

The MAiD provision process includes supports leading up to and during the provision, final consent, protocols for the administration of medication, and completion of the death certificate. Where MAiD teams exist, patients and families are usually supported by one or more team members, who may facilitate access to support services, encourage patients to have conversations with families and suggest ways to manage them, offer information on available resources for families, and provide counselling services. Where MAiD services are not coordinated by a MAiD team, assessors and/or providers often take on these roles. Patients eligible for MAiD who have no family or friends are supported directly by the MAiD team or indirectly through referrals to existing hospital or community-based services.

In all jurisdictions, processes for accommodating preferences of patients and families for the provision have been established. They involve one or more conversations with the patient and family organized by the MAiD team or by the physician or NP scheduled to perform the provision. Conversations may be guided by policies developed by the jurisdiction, which highlight the importance of respecting a patient’s dignity and autonomy and emphasize the need to ensure conversations address patients’ cultural and religious beliefs, spiritual and linguistic needs, values, and wishes. Often, MAiD teams and providers are also involved in supporting the patient and family with logistical arrangements regarding the timing and location of the provision. While efforts are made to accommodate the requests of patients and families, they are ultimately determined by provider availability and urgency of provision. In some jurisdictions, provisions only take place during set time periods (e.g., weekdays). In all jurisdictions, patients are contacted a few days in advance to confirm the date and their plans to proceed with the provision. Importantly, they are reminded that they can withdraw/change their mind at any time up until the point of final consent, which typically occurs just before MAiD medications are administered. In most jurisdictions, final consent can be made verbally or in writing. However, a few accept written consent only, unless the patient is unable to speak or write. While all jurisdictions allow waivers of the final consent, British Columbia is the exception, specifying that they cannot be created on the same day as the provision, and if an alternative provider becomes responsible for the provision, another waiver must be signed.

In general, MAiD protocols for provider-administered medications are standardized at the P/T or regional levels. However, where there is no provincially/territorially standardized protocol, providers refer to CAMAP protocols. In some jurisdictions, patients have the option of self-administered MAiD, in which a physician or NP prescribes medication to be ingested orally. Importantly, all jurisdictions offering self-administration, with the exception of Ontario, require a back-up plan to be in place in the event that it is unsuccessful. This consists of written consent enabling a provider to intervene with IV MAiD if the patient loses capacity after an agreed upon/specific amount of time.

With respect to post-provision planning (i.e., care of the remains), most jurisdictions consider it the patient’s responsibility or that of their family/friends/executor of the will. Regarding completion of the death certificate following a provision, in all but one jurisdiction, it is the responsibility of the MAiD provider. The exception is Alberta, where the Office of the Chief Medical Examiner certifies the death. The cause of death stated on the death certificate varies across jurisdictions, but the manner of death is almost always stated as ‘natural’. The exceptions are Saskatchewan and Alberta, where it is recorded as ‘unclassified’.

### 3.9. Bereavement

Several jurisdictions have dedicated grief and bereavement services within their MAiD programs, including a support group through British Columbia-Island Health, up to six free sessions with a social worker in Manitoba, bereavement sessions on request in Nova Scotia, and grief resources and counselling with a mental health nurse in Ontario-Hamilton Family Health Team. In all jurisdictions, the MAiD team itself, or the physician or NP who performed the provision, offers immediate emotional support following a provision and usually leaves the family with their contact details. In addition, they may provide information on available resources. In some jurisdictions, the MAiD team ‘checks in’ with family members, and social workers or mental health nurses on the team may provide some grief support, as needed. In others, MAiD programs rely on non-MAiD-specific grief and bereavement services offered at the institutional, regional or P/T health authority/department levels. Across jurisdictions, families are referred to community-based support groups organized by non-profit organizations, such as Bridge C-14, Dying with Dignity, MAID Family Support Society, and the Canadian Virtual Hospice.

### 3.10. Specific Populations

#### 3.10.1. Individuals Who Are Incarcerated

In all jurisdictions, experience with incarcerated patients is limited, and no specific policies exist within MAiD programs. Some MAiD programs have worked collaboratively with correctional facilities, adhering to federal and P/T policies governing them. Eligibility assessments may be conducted in person or virtually, and efforts to accommodate preferences often involve arrangements for provision in the community following compassionate release. This depends upon a patient’s security level and history, and some jurisdictions acknowledge that it may not be possible to accommodate patient and family preferences.

#### 3.10.2. Individuals Who Are Indigenous

In most jurisdictions, there are no specific MAiD policies for Indigenous patients and families. However, MAiD teams often collaborate with Indigenous liaisons/health advocates in an institution or health authority/department. Indigenous liaisons have been involved in creating and supporting engagement plans with First Nations and Métis communities, facilitating access to MAiD information and specific community-based resources, resolving difficult situations relating to community resistance to MAiD, connecting patients with Indigenous leaders and Elders to ensure support is provided in a culturally sensitive way, identifying Indigenous assessors and providers, and making arrangements for MAiD provisions that incorporate Indigenous practices. Specifically, in Alberta, Manitoba, and the New Brunswick Vitalité Health Network, MAiD programs proactively work with Indigenous supports in the community (e.g., First Nations coordinators or a network of spiritual care providers) to facilitate access to MAiD information and specific community-based resources available to them. In Prince Edward Island, the MAiD program collaborates with the Indigenous Native Council to make presentations to Native Council, Indigenous health care teams, and community members. In the Northwest Territories and the Yukon territory, MAiD programs contact local Indigenous Band Offices to connect patients and families with community Elders, community social workers, or local Indigenous wellness programs.

#### 3.10.3. Individuals Who Are Housing-Insecure

Although no jurisdictions have developed specific MAiD policies for patients who are housing-insecure, when cases are identified, MAiD programs collaborate with existing community-based resources and organizations.

### 3.11. Training and Education for Healthcare Professionals Outside of MAiD Teams

In some jurisdictions, MAiD programs have developed training sessions to support a broad range of healthcare staff. Participants may include MAiD care coordinators, home and community care nurses, social workers, speech pathologists, long term care staff, palliative care physicians, legal professionals, and educators. In other jurisdictions, staff are referred to training modules offered by CAMAP. In addition to training sessions, some programs have developed education/information sessions for healthcare professionals in general. The content covered varies, but can include the MAiD process, referral procedures, conscientious objection, eligibility criteria, responsibilities of healthcare professionals, available resources for staff, and available resources for patients and families.

### 3.12. Public Awareness

Several jurisdictions provide educational sessions to the public. Educational sessions are usually hosted by the P/T medical lead for the MAiD program. In several jurisdictions, programs provide one-on-one sessions at the request of patients and families. In British Columbia, Vancouver Coastal Health provides Indigenous-led educational sessions for Indigenous communities.

### 3.13. Patient and Family Feedback

Patient and family feedback refers to initiatives designed to elicit the views of patients and families for quality improvement purposes. The extent to which MAiD pro-grams seek feedback from patients and families varies across jurisdictions. In British Co-lumbia, Fraser Health and Vancouver Coastal Health programs post optional surveys on their website. Several jurisdictions conduct follow-up phone calls with family members post-provision or encourage patients and families to share feedback with care coordi-nators. In other jurisdictions, programs rely on generic patient and family feedback options available at the institutional or health authority/department level.

## 4. Discussion

To our knowledge, this study is the first of its kind in providing a comprehensive description of MAiD programs across Canada. Based on our findings there is no one-size-fits-all approach to the structure, organization, delivery, and oversight of MAiD-related services across provinces and territories.

Key aspects of MAiD programs related to access, equity, and safeguards may include consideration of centralized programs that are generally more robust in their composition of multidisciplinary team members and provide a broader range of services to patients and families. This adds to research previously cited that encourages centralized [13,14], coordinated services that are more holistic in nature to ensure that the unmet needs of patients and families are addressed, such as social determinants of health [9,12,15] and the provision of information, guidance, and emotional support [11,12,13,14,15,16,18].

This study may provide a further understanding of the accessibility of MAiD related to unclear pathways and the potential for healthcare professionals to be unresponsive to requests, especially those related to conscientious objection [11,12,13,14,16]. Jurisdictions vary in their processes for requests and intake—many offer both options for individual physicians and NPs or centralized programs to receive requests and complete intakes—which may cause confusion for patients and families [11,12,13,14]. In turn, additional supports such as ongoing guidance and grief and bereavement services may or may not be available to patients and families based on individual practitioner practices versus centralized programs. However, it remains unclear the extent to which centralized programs provide holistic services to patients and families requesting and receiving MAiD.

Findings from this study include varying interpretations of safeguards such as the definition of independent assessments and specific definitions and timelines related to foreseeable and non-foreseeable natural deaths. This in turn may help to understand family concerns related to comprehensive and accurate assessments of their loved ones who have received MAiD [10]. Varied training practices or lack thereof based on voluntary versus mandatory policies may also offer some insight into previously noted concerns related to guidance, information sharing, and clear eligibility requirements for patients and families [11,12,13,14,16,18]. Further standardization of practices and definitions of eligibility criteria along with required training for assessors may address such concerns. Western Australia for example includes mandatory training for medical practitioners involved in their voluntary assisted dying program, and legislation includes detailed requirements regarding the process, including specific prognosis criteria [8,22,23]. Programs with strong oversight and monitoring may ensure safeguards and best practices are met and addressed [17]. Columbia, for example, requires approval by an independent committee prior to euthanasia taking place [8]. Increased transparency and accountability, including regular reporting on jurisdictional findings at P/T levels, may also begin to highlight areas for quality improvement and possible legislative updates related to procedures and safeguards [10,17].

Finally, findings from this study associated with individuals more vulnerable to systemic and structural oppression such as those who are incarcerated, housing-insecure, or Indigenous show that clear policies are missing and practices are varied. The United Kingdom has included the requirement of a disability advisory board to report regularly on the impact of their assisted dying bill when it comes into operation [24]. Such practices may aid in the ongoing development and improvement of safeguards for more vulnerable populations. Programs and services that include culturally sensitive, respectful, patient- and family-centered, trauma-informed policies and practices may also begin to address concerns related to individuals who are vulnerable and potentially requesting MAiD due to unmet needs [25,26,27].

## 5. Conclusions

As Canada prepares for further expansion of MAiD in 2027 and the number of inter-national jurisdictions continue to grow in the implementation of assisted dying legislation and programs, lessons can be learned from this comprehensive review. From the initial request to assessments, provisions, and post-provision bereavement supports, jurisdictions throughout Canada vary. Further standardization of MAiD procedures and practices, clarity on eligibility criteria, and increased monitoring and reporting, legislatively and/or within provinces and territories may begin to address the unmet needs and social determinants of health of vulnerable populations and patient and family concerns regarding the process.

### 5.1. Study Limitations

Our sample does not include information from the territory of Nunavut and therefore does not provide a fully pan-Canadian understanding of MAiD, with the Indigenous perspective and experience of MAiD being limited. Sample sizes within some jurisdictions are also limited and may therefore not provide a fully comprehensive understanding of policies and practices within those MAiD programs and services. This is especially significant in light of findings that MAiD practices may vary between decentralized jurisdictional programming and between healthcare practitioners within jurisdictions.

### 5.2. Future Research

Future research is suggested related to assessment practices to further understand their comprehensiveness, including social determinants of health as they relate to unmet needs, and to consider best practices. A further understanding of jurisdictional oversight and monitoring practices may also provide further insight into concerns such as access, equity, and safeguards and offer quality improvements. A comparison of centralized and decentralized programs is also suggested to consider high-quality models of care that will address challenges such as unclear pathways, a lack of guidance and information, clarity of eligibility requirements, and ongoing emotional support.

## Data Availability

The original contributions presented in this study are included in the Supplementary Materials as the MAiD Categorized Tables. Further inquiries can be directed to the corresponding author.

## Supplementary Materials

The following supporting information can be downloaded at: https://www.mdpi.com/article/10.3390/healthcare14060797/s1, S1: MAiD Services PRISMA Flow Diagram. S2: MAiD Recruitment Letter. S3: MAiD Consent Form. S4: Interview Guide for MAiD Teams. S5: Interview Guide for MAiD Program Administrative and Clinical Team Members. S6: MAiD Services Coding Framework. S7: MAiD Categorized Tables of Results. Refs. [28,29,30,31,32,33,34,35,36,37,38,39,40,41,42,43,44,45,46,47,48,49,50,51,52,53,54,55,56,57,58,59,60,61,62,63,64,65,66,67,68,69,70,71,72,73,74,75,76,77,78,79,80,81,82,83,84,85,86,87,88,89,90,91,92,93,94,95,96,97,98,99,100,101,102,103,104,105,106,107,108,109,110,111,112,113,114,115,116,117,118,119,120,121,122,123,124,125,126,127,128,129,130,131,132,133,134,135,136,137,138,139,140,141,142,143,144,145,146,147,148,149,150,151,152,153,154,155,156,157,158,159,160,161,162,163,164,165,166,167,168,169,170,171,172,173,174,175,176,177,178,179,180,181,182,183,184,185,186,187,188,189,190,191,192,193,194,195,196,197,198,199,200,201,202,203,204,205,206] are cited in Supplementary Materials.

## Abbreviations

The following abbreviations are used in this manuscript:

AB	Alberta
BC	British Columbia
CAMAP	Canadian Association of MAiD Assessors and Providers
CMDP	Council of Physicians, Dentists, and Pharmacists
HAs	Regional Health Authorities
IV	Intravenous
MAiD	Medical Assistance in Dying
MB	Manitoba
NB	New Brunswick
NL	Newfoundland and Labrador
NP	Nurse Practitioner
NPs	Nurse Practitioners
NS	Nova Scotia
NT	Northwest Territories
NU	Nunavut
ON	Ontario
PEI	Prince Edward Island
P/T	Provincial and territorial
P/Ts	Provinces and territories
QC	Québec
RHAs	Regional Health Authorities
SK	Saskatchewan
YT	Yukon

## Appendix A

**Table A1:** Possible MAiD team members. X = Applies.

Jurisdiction	Medical Director	Clinical Lead	Program Manager/Lead	Coordinator/Nurse Navigator	Social Worker	Mental Health Clinician/Nurse	Indigenous Advisor
Alberta—Alberta Health Services	X		X	X			
British Columbia—Ministry of Health							
British Columbia—Fraser Health	X		X	X		X	
British Columbia—Interior Health	X			X			
British Columbia—Island Health	X		X	X			
British Columbia—Northern Health	X			X			
British Columbia—Vancouver Coastal Health	X			X			X
British Columbia—Provincial Health Services Authority	X		X	X			
Manitoba—Shared Health	X		X	X	X [207]		
New Brunswick—Horizon Health Network	X			X			
New Brunswick—Vitalité Health Network							
Newfoundland and Labrador—Eastern Zone	X			X			
Newfoundland and Labrador—Western Zone				X			
Newfoundland and Labrador—Central Zone			X	X			
Northwest Territories		X	X	X			
Nova Scotia—Nova Scotia Health		X	X	X	X		
Prince Edward Island—Health PEI		X	X	X	X		
Saskatchewan—Saskatchewan Health Authority	X		X	X	X		
Yukon—Department of Health and Social Services							
Ontario—Ministry of Health			X	X			
Ontario—Home and Community Care Support Services, Central East			X	X			
Ontario—Home and Community Care Support Services, Waterloo Region				X			
Ontario—Home and Community Care Support Services, South East				X			
Ontario—Home and Community Care Support Services, South West				X			
Ontario—Champlain Regional MAiD Network, The Ottawa Hospital			X	X			
Ontario—Peterborough Regional Health Centre				X	X		
Ontario—Mount Sinai Healthcare Facility, Toronto				X			
Ontario—University Health Network	X			X			
Ontario—Grand River Hospital				X			
Ontario—Hamilton Family Health Team			X	X		X	

## Appendix B

**Table A2:** Jurisdiction organization. X = Applies.

Jurisdiction	Centralized	Decentralized	Hybrid
Alberta-Alberta Health Services	X [208,209,210]		
British Columbia—Fraser Health	X [211]		
British Columbia—Interior Health	X		
British Columbia—Island Health			X
British Columbia—Northern Health	X		
British Columbia—Vancouver Coastal Health	X		
British Columbia—Provincial Health Services Authority	X		
Manitoba—Shared Health	X [207]		
New Brunswick—Horizon Health Network	X		
New Brunswick—Vitalité Health Network		X	
Newfoundland and Labrador—Eastern Zone	X [212]		
Newfoundland and Labrador—Western Zone	X		
Newfoundland and Labrador—Central Zone	X		
Northwest Territories	X [213]		
Nova Scotia—Nova Scotia Health	X		
Prince Edward Island—Health PEI	X		
Saskatchewan—Saskatchewan Health Authority	X		
Yukon—Department of Health and Social Services		X [214]	
Ontario—Ministry of Health		X	
**Service Organizations/Regional Facilities**			
Ontario—Home and Community Care Support Services, Central East	X		
Ontario—Home and Community Care Support Services, Waterloo Region	X		
Ontario—Home and Community Care Support Services, South East	X		
Ontario—Home and Community Care Support Services, South West	X		
Ontario—Champlain Regional MAiD Network, The Ottawa Hospital	X		
**Healthcare Facilities**			
Ontario—Peterborough Regional Health Centre	X		
Ontario—Mount Sinai Healthcare Facility, Toronto	X		
Ontario—University Health Network	X		
Ontario—Grand River Hospital	X		
**Community of Practice**			
Ontario—Hamilton Family Health Team	X		
Ontario—Niagara Community MAiD Team, St. Catharine’s, Niagara		X	
Québec—Ministry of Health and Social Services		X	
**Integrated Health and Social Services Centres**			
Québec—CISS Montérégie	X		
**Integrated University Health and Social Services Centres**			
Québec—CIUSS Capitale-Nationale	X [215]		
Québec—University of Montreal Hospital Center	X		
Québec—McGill University Health Centre	X		

## Appendix C

**Table A3:** Reporting process. X = Applies.

Jurisdiction	Direct	Delegated	Mixed
Alberta		X	
British Columbia		X	
Manitoba	X		
New Brunswick	X		
Newfoundland and Labrador	X		
Northwest Territories		X	
Nova Scotia	X		
Ontario			X
Prince Edward Island	X		
Québec		X	
Saskatchewan		X	
Yukon	X		
