# Supplementary material for: Descriptions and Experiences with Medical Assistance in Dying Models Across Canada: A Mixed Methods Study
Source: Healthcare (Basel). 2026 Mar 20;14(6):797. doi: 10.3390/healthcare14060797 (PMC13027146; doi:10.3390/healthcare14060797)
Supplement: Supplementary file 1 [file healthcare-14-00797-s001.zip › S2-MAiD Recruitment Letter .pdf]

Health Technology & Policy Unit
School of Public Health
4-341 Edmonton Clinic Health Academy
11405 87 Avenue, Edmonton, AB T6G 1C9
Tel: 780-492-9080

Medical Assistance in Dying (MAiD): Descriptions of and Experiences with Models Across Canada (Research Ethics ID: Pro00129886)

Principal Investigator: Devidas Menon, PhD, MHSA
Professor
School of Public Health
University of Alberta
4-341 Edmonton Clinic Health Academy
11405 87 Ave NW, Edmonton, AB T6G 1C9
Tel: 780-492-9080
E-mail: menon@ualberta.ca

[Insert date]

Dear [insert name of MAiD program director or manager]:

My name is Devidas Menon, and I am a Professor of Health Policy and Management in the School of Public Health at the University of Alberta. My research group has been funded by Health Canada to carry out a review of MAiD services across Canada. The purpose of this review is to better understand how MAiD services have been rolled out in different jurisdictions and whether access varies across patient populations.

The review will include interviews with individuals involved in the management and delivery of MAiD programs across Canada. Interviews will last about 45 minutes and be conducted over the phone or virtually through Zoom, Google Meets or Microsoft Teams. They will be one-on-one and kept confidential.

I am writing to you to ask if you would consider participating in one of these interviews. Attached is a copy of the information letter and consent form. If you require further information or have any questions, please do not hesitate to contact me (The Principal Investigator).

Sincerely,

Dev Menon
