# Supplementary material for: Descriptions and Experiences with Medical Assistance in Dying Models Across Canada: A Mixed Methods Study
Source: Healthcare (Basel). 2026 Mar 20;14(6):797. doi: 10.3390/healthcare14060797 (PMC13027146; doi:10.3390/healthcare14060797)
Supplement: Supplementary file 1 [file healthcare-14-00797-s001.zip › S3-MAiD Consent Form_EN copy.pdf]

Project Title: Medical Assistance in Dying (MAiD): Descriptions of and Experiences with Models Across Canada

Principal Investigator

Devidas Menon, PhD, MHSA
Professor
School of Public Health
University of Alberta
4-341 Edmonton Clinic Health Academy
11405 87 Ave NW, Edmonton, AB T6G 1C9
Tel: 780-492-9080
E-mail: menon@ualberta.ca

Introduction

You are being invited to participate in a project about MAiD services in Canada.

This project will explore the experiences of healthcare providers who have been involved in MAiD services in Canada. Services include consultations, counselling, assessments and provision of MAiD.

This form contains information about the project. A member of the team will explain the project to you in detail. Please feel free to ask questions about anything you do not understand. You will be given a copy of this form for your records.

Objective of the Research Project

In Canada, MAiD became legal in 2016. Since then, programs offering these services have been established across the country, and many patients and families have accessed them. We hope to learn from your experience so we can understand, from your perspective, what is working well and what needs to be improved.

Nature of my Participation

We would like to conduct an interview with you to talk about your experience with MAiD program in your province or territory. We will ask about the MAiD program and services

offered, as well as how you feel about them. The interview will last about 45 - 60 minutes and be conducted by phone or via an online platform such as Zoom or Google Meets at a time that is convenient for you. The interview will be audio-recorded and transcribed.

Project Timeline

This project will last approximately eighteen months. However, there are no additional time commitments required from you once you sign the consent form and complete the interview.

Risk and Inconveniences potentially resulting from my participation

The project has minimal risk. Your participation is completely voluntary. While it is not possible to know all of the risks that may happen in a project, the team has taken all reasonable safeguards to minimize any known risks to a participant.

Potential Benefits of my participation

There are no direct benefits to you by participating in project. However, you will be helping us learn more about existing MAiD services and how they can be improved to better serve those involved in MAiD, including healthcare providers, patients and families.

Voluntary Participation and Right to Withdraw

If you agree to participate in the project, you will be asked to sign this consent form and we will schedule an interview. Being in this project is your choice. If you choose to participate, you can change your mind and withdraw from the project at any time. If you would like us to destroy data collected from you, then we will make every effort to accommodate your request. But to be sure that we are able to destroy your data, please let us know that you are withdrawing within 3 months from the date of your interview. After this time, it may no longer be possible, as your data would have been analyzed with other data and cannot be removed or destroyed from the study. If you leave the project, we will not collect new information about you, but we will need to keep the data that we have already collected.

Financial Compensation

You will not receive no compensation for your participation in this research

Confidentiality and Anonymity

During the project, we will be collecting information about you. We will do everything we can to make sure that it is kept private. No data including your name will be released outside of the project team's office or published by the project team. Sometimes, by law, we may have to release your information with your name, so we cannot guarantee absolute privacy. However, we will make every legal effort to make sure that your information is kept private.

During the project, it is important that the data we get are accurate. For this reason, your data, including your name, may be looked at by the principal investigator and/or the study coordinator. Any personal information that we get from these records will be only what is needed for the project.

UNIVERSITY OF ALBERTA

Health Technology & Policy Unit
School of Public Health
4-341 Edmonton Clinic Health Academy
11405 87 Avenue, Edmonton, AB T6G 1C9
Tel: 780-492-9080

By signing this consent form you are saying it is okay for the project team to collect, use and disclose information about you as described above.

All documentation with identifying information will be kept in a secure and confidential location in the Health Technology and Policy Unit at the School of Public Health at the University of After the project is done, we will still need to securely store data we collected as part of the project for a minimum of 5 years. All the information collected during the process will be kept strictly confidential and will only be available to the project team.

We will protect your identity by labelling your data with a project identification code rather than your name. Data will be summarized in a manner that prevents readers from being able to identify the setting in which it is collected. All identifying information will be removed.

Quotations from the interviews may be used in the final report to help explain the results. However, names and identifying information will not be included. The final report may be submitted for publication in a peer-reviewed journal and presented at conferences.

Contact Persons

If you have any questions about the research now or later, please contact the PI at 780-492-9080.

Declaration by the participant

By signing below, you understand:

- That you have read the above information and have had anything that you do not understand explained to you to your satisfaction.
- That you will be taking part in a research project.
- That you may freely leave the research study at any time.
- That you understand the interviews will be audio-recorded strictly for the purpose of this project
- That you do not waive your legal rights by being in the project
- That the legal and professional obligations of the investigators and involved institutions are not changed by your taking part in this project
- That you will receive a signed copy form of this consent form.

Initials of Participant: ___________________________

____

Name of Participant

____________________________________ ________________________

Signature of Participant Date

____

Role of Participant

____________________________________ ____________________________________

E-mail address Phone Number

Declaration by the person responsible for obtaining the consent

I, ______________________________________, undersigned, attest having explained the terms of this form to the prospective participant, having answered the prospective participant’s questions, and having clearly indicated that the prospective participant is free to terminate participation in the above project at any time.

____________________________________ ________________________

Name of Person Obtaining Consent Contact Number

____________________________________ ________________________

Signature of Person Obtaining Consent Date

Initials of Participant: ___________________________
