# Supplementary material for: Descriptions and Experiences with Medical Assistance in Dying Models Across Canada: A Mixed Methods Study
Source: Healthcare (Basel). 2026 Mar 20;14(6):797. doi: 10.3390/healthcare14060797 (PMC13027146; doi:10.3390/healthcare14060797)
Supplement: Supplementary file 1 [file healthcare-14-00797-s001.zip › S4-Interview Guide for MAiD Teams.pdf]

Supplementary Material S4: Interview Guide for MAiD Teams

Preamble:

Thank you for agreeing to participate in this project. I am interviewing you to better understand the MAiD program in [insert name of organization or jurisdiction] and your experiences as part of that program. I realize you have already had an opportunity to ask questions and signed the consent form, but I would be happy to review your rights as a participant again and answer any questions you might have before we get started.

The interview will take about an hour and will be audio recorded and transcribed. There are no right or wrong answers to these questions. All responses will be kept confidential. You can withdraw from the project at any time prior to the end of data analysis without having to give a reason. After data analysis has been completed and the report has been written, it will no longer be possible for you to withdraw from the project. If you are OK, I will start recording this session.

Introduction:

1. What services are provided as part of the MAiD program?

2. Who is involved in the delivery of services provided as part of the program?

    a) Is there a multidisciplinary team? If yes, who comprises the team?

    b) Who is full-time and who is part-time?

3. How does the MAiD program operate? For example, is it clinic based?

4. Where does the MAiD program ‘live’ within the organization?

5. Who oversees the program?

6. What is your role in the program?

7. For how long have you been in this role?

8. What made you decide to become a part of the program?

The following questions are about the steps involved in the MAiD process.

Referral to the MAiD program:
1. What role does the MAiD program play in increasing public awareness of MAiD or educating patients, families and healthcare providers about MAiD?

2. In your experience, how do patients typically become aware of MAiD?

    a) Is MAiD ever raised as an option by a healthcare provider?

    b) Does a patient and/or family member need to have a conversation with their primary care provider before referring themselves to the MAiD program?

    c) What happens if a patient requests a referral for MAiD from their primary care provider but their provider declines?

3. How are referrals to the MAiD program managed?

    a) Is the process centralized or decentralized?

4. Who can make a referral?

    a) Can a healthcare provider on behalf of a patient?

    b) Can a family member?

5. What happens when your program receives a referral?

a) Is the request reviewed by someone within the program before the patient and patient's family are contacted? If yes, who reviews it and what does that review involve?

6. Does anything else happen before an initial consultation or appointment is scheduled with the patient?

7. Where does the initial appointment or consultation take place?

8. Who decides on the time and location of that appointment?

9. What happens during that appointment?

Assessment for MAiD:
1. After the decision is made to move forward with an assessment, how is the time and place of that assessment determined?

2. Are there ever requests for a specific gender of assessor? If yes, how are those requests managed?

3. What's involved in the assessment?

4. How do you determine whether a patient meets the eligibility criteria?

5. For Track 1 patients, what does "reasonably foreseeable natural death" mean? What does it look like?

a. Can you provide a couple of examples?

6. For Track 2 patients, in whom death is not foreseeable, how do you determine whether they have “given serious consideration to means to relieve their suffering and have been offered consultations with professionals who provide these services and treatments”?

    a. What would be an example of such a patient?

7. I understand that two independent assessments are needed. Who determines when and where the second independent assessment takes place?

8. What happens during the second assessment – is the process identical to the first assessment?

    a. Again, does the patient have a say in terms of the gender of the assessor?

9. What happens when the patient does not speak English or they are unable to speak?

10. What happens when the two assessors disagree?

11. Should a third assessment be required, what efforts are made to reduce the burden on the patient?

12. What does the third assessment look like and who is involved?

13. What happens when a patient wants MAiD but their family does not support it?

14. If the patient is deemed eligible for MAiD, is there a time by which MAiD must be delivered?

15. How are requests to incorporate religious, ethnic or cultural traditions managed? Could you provide some examples?

Provision of MAiD:

1. Is it possible to deliver MAiD when and wherever the patient chooses?

    a. What about rural and remote communities?

    b. What about on reserves?

    c. What if the patient is incarcerated?

2. What happens if the patient resides in a facility that does not support MAiD?

3. What happens if a patient changes their mind at the last minute?

4. What steps are taken to ensure that the MAiD provider is aware of a patient's wishes in terms of loved ones they want present and activities that are carried out?

5. What steps are taken to ensure that those wishes are fulfilled?

6. What options for medications used in MAiD are available?

7. Who decides how MAiD will be administered?

8. Can you walk me through what's involved in the final appointment when MAiD is carried out?

9. What happens when a patient has no family or friends to be with them during MAiD?

10. Who is responsible for reporting the death and completing any necessary paperwork?

Support and bereavement:

1.To what extent is the program involved in helping the family navigate next steps once their loved one has passed away?

2. Are arrangements made with a funeral home prior to the MAiD appointment?

3. What supports are available to the family after their loved one passes away?

4. Who in the MAiD team is involved in providing grief and bereavement support?

5. What happens when there is no family to handle arrangements after MAiD has been carried out?

Patients who are Indigenous:

1. Does your program have dedicated resources or supports for Indigenous populations?

2. How does the program work with Indigenous patients and families to provide MAiD in a way that honors their beliefs and values?

3. Are you aware of any barriers to accessing MAiD for Indigenous patients? If yes, please describe them.

4. In your view, what should be done to reduce or even remove those barriers?

5. If there doesn't appear to be any barriers, what do you think has enabled your program to achieve such a success?

Patients with specific cultural, ethnic or religious beliefs:

1. Has your program experienced any challenges when working with families with specific cultural, ethnic or religious beliefs?

    a. Would you be able to share some of those challenges and how they were managed?

2. Has your program experienced any particular successes when working with families with specific cultural, ethnic or religious beliefs?

    a. Would you be able to share some of those successes?

Patients who are unhoused/experiencing homelessness:

1. How does a patient who is unhoused or experiencing homelessness access the MAiD program?

2. What are some of the challenges involved in carrying out consultations, assessments, and if determined appropriate, the provision of MAiD?

3. How have these challenges been managed?

4. What do you think could be done to improve access for this population?

Patients who are incarcerated:

1. How does someone who is incarcerated access the MAiD program?

2. How are consultations, assessments and if appropriate, the provision of MAiD carried out?

3. To your knowledge, has the MAiD program ever received a referral from an incarcerated patient?

    a. If yes, to what extent did the process differ from that for patients who are not incarcerated?

4. What are some of the challenges involved in carrying out consultations, assessments and, if determined appropriate, MAiD in this population?

5. What could be done to address those challenges?

6. If an incarcerated patient is deemed eligible for MAiD and chooses to proceed with it, is there an opportunity for loved ones to be present and/or special activities or ceremonies to be performed?

Funding:

1. How are MAiD assessors and providers remunerated?

3. How is travel to remote or rural communities compensated?

4. How are appointments in the evenings and weekends compensated?

5. In your view, to what extent does financial compensation play a role in healthcare providers' willingness to be part of the MAiD program?

Wrap up:

1. Is there anything else you would like to share?

2. If I have additional questions or would like to clarify anything, can I reach out to you?

3. Is there anyone else you think we should reach out to?

a) If yes, could you please ask them if you can share their contact information with us?

Thank you again for taking the time to participate in this project. Your insights are greatly appreciated.
