# Supplementary material for: Descriptions and Experiences with Medical Assistance in Dying Models Across Canada: A Mixed Methods Study
Source: Healthcare (Basel). 2026 Mar 20;14(6):797. doi: 10.3390/healthcare14060797 (PMC13027146; doi:10.3390/healthcare14060797)
Supplement: Supplementary file 1 [file healthcare-14-00797-s001.zip › S5-MAiD Interview Guide - Program Administrative and Clinical Team Members.pdf]

Supplementary Material S5: Interview Guide for MAiD Program Administrative and Clinical Team Members

Preamble:

Thank you for agreeing to participate in this project. We are chatting with you today to better understand your MAiD program and your experiences as part of the program. I realize you have already signed the consent form, but I would be happy to review anything or answer any questions you might have before we get started.

We are hoping to record the interview so that we don't miss anything, but all of your responses will be kept confidential. Are you okay with that? Just as a reminder since it has probably been a couple of weeks since you signed the consent form - you can withdraw from the project at any time prior to the end of data analysis without having to give a reason. After data analysis has been completed and the report has been written, it will no longer be possible for you to withdraw from the project. If you are OK, I will start recording this session.

We are going to start out with some questions that are quite broad.

Introduction

1. Can you give us an overview of the MAiD program you're involved in and the services that are provided as part of the MAiD program?
2. Who is involved in the delivery of services provided as part of the program?
   a) Is there a multidisciplinary team? If yes, who comprises the team?
      b) Who is full-time and who is part-time?

Probe: Is there a MAID coordinating Team? If yes, how does their role differ from those involved in the delivery of MAID services?

3. To what extent has the MAiD team been involved in setting up processes within facilities like hospitals and continuing care or long-term care centres?
4. How does the MAiD program operate? For example, is it clinic based?
5. Where does the MAiD program 'live' within the organization?
6. Does [province/territory] have a MAID policy? If yes, what does it include?
   Probe: Does it define steps to support a patient through the MAiD process?
7. Who oversees the MAiD program?
8. How are palliative care programs or organizations involved in the MAiD program?

9. How are hospices involved in the MAiD program?

10. What is your role in the program and where would you come in from the time a patient or family member contacts the MAiD team with a request for a referral to the time a patient receives MAiD?

11. For how long have you been in this role?

12. What made you decide to become a part of the program?

We are now going to move on to questions are about the specific steps involved in the MAiD process.

Referral to the MAiD program

1. What role does the MAiD program play in increasing public awareness of MAiD or educating patients, families and healthcare providers about MAiD?
2. In your experience, how do patients typically become aware of MAiD?

Probe:

A. Is there a central phone number or e-mail and if yes, how do patients and families typically find out about it?

B. Are there request forms on a website that patients and families can download, print, complete and send somewhere?

C. If they aren't publicly accessible, how do they access them, and do you know what some of the reasons behind the decision not to make them publicly accessible might have been?

3. Is MAiD ever raised as an option by a healthcare provider?
4. Does a patient and/or family member need to have a conversation with their primary care provider before referring themselves to the MAiD program?
5. What happens if a patient requests a referral for MAiD from their primary care provider but their provider declines? Who is responsible for reporting the request to Health Canada?

   Probe: If it comes to your attention that a patient explicitly requested MAiD from their primary care provider and they were told that they wouldn't be eligible, who is required to report that to Health Canada?
6. How are referrals to the MAiD program managed? Is the process centralized or decentralized? What we mean by centralized is a single point of entry or access into the MAiD program.

7. Who can make a referral?
   A. Can a healthcare provider on behalf of a patient?
   B. Can a family member?
   C. If a physician objects to the referral, what is the process to ensuring the patients is provided with MAiD services?
8. Who is responsible for reporting the request? Does it need to be reported?
   Probe: Who is responsible for reporting an explicit request for MAiD (verbal, email, text message- NOT the legal form) to the province/Health Canada?
9. What happens when your program receives a referral?
   A. Is the request reviewed by someone within the program before the patient and patient's family are contacted? If yes, who reviews it and what does that review involve?
10. When would a preliminary assessment take place?
    Probe: What is the purpose of that assessment? Is it mainly to screen patient requests for MAiD to determine whether they are likely or not likely to meet the eligibility criteria (from the document) for MAiD prior to assigning an assessor(s)?
11. Who would be responsible for reporting this to Health Canada? Who is responsible for reporting an explicit request for MAiD (verbal, email, text message- NOT the legal form) to the province/Health Canada?
12. Does anything else happen before an initial consultation or appointment is scheduled with the patient?
    Probe: Is information on options for relief from suffering, whatever that might look like provided or does that conversation happen at some other point (e.g., during the initial appointment or later during the assessment)?
13. Where does the initial appointment or consultation take place?
14. How are the time and location of that appointment determined (e.g., who decides)?
15. What happens during that appointment?

Preliminary assessor questions

1. Do you conduct preliminary assessments?

If yes, ask underlisted questions. If no, skip to question 8

2. We understand that there are preliminary assessment forms that need to be filled out if a preliminary assessment takes place. Who would be a preliminary assessor?

Probe: What policies or guidance governs the qualifications and role of a preliminary assessor, including a regulated professional in a care team, a care coordination service, or other relevant setting in the MAiD assessment process?

3. When would a preliminary assessment take place?

Probe: A. What is the purpose of that assessment?

B. Is it mainly to screen patient requests for MAiD to determine whether they are likely or not likely to meet the eligibility criteria (from the document) for MAiD prior to assigning an assessor(s)?

4. How does a preliminary assessor differentiate between an explicit request for MAID and a request for information regarding "applicability of eligibility"?

5. How does a preliminary assessor determine whether eligibility or ineligibility is likely?

6. If a preliminary assessment does take place, under what circumstances does the preliminary assessment form need to be completed and submitted to Health Canada?

Probe: If a preliminary assessor is in the community, how do they report?

7. What is a preliminary assessor required to report to Health Canada if they determine a patient would not be eligible?

8. If there is no preliminary assessment, are all requests received for MAID (not for information only) automatically sent on to a provider and assessor?

Assessment for MaiD

1. After the decision is made to move forward with an assessment, how is the time and place of that assessment determined?

Probe: Are assessors only available during specific times? If yes, when?

2. Who conducts the assessment?

3. How does a person become authorized to conduct an assessment?

4. Are there ever requests for a specific gender of assessor? If yes, how are those requests managed?

5. What's involved in the assessment?

Probe: Are there any forms or aids to guide assessors through the assessment? If yes, they accessible to patients and families or just to the MAiD team?

Probe: Has assessment being provided to patients via digital technology? If yes, what is the process to achieving this?

6. How do you determine whether a patient meets the eligibility criteria?

7. For Track 1 patients, what does “reasonably foreseeable natural death” mean? What does it look like?

   a. Can you provide a couple of examples?

8. For Track 2 patients, in whom death is not foreseeable, how do you determine whether they have “given serious consideration to means to relieve their suffering and have been offered consultations with professionals who provide these services and treatments”?

Probe: How do you determine the length of time a patient has received these services?

9. I understand that two independent assessments are needed. Who determines when and where the second independent assessment takes place?

10. Is the second assessor made aware of the first assessor’s findings before they conduct their assessment?

11. What happens during the second assessment – is the process identical to the first assessment?

   a. Again, does the patient have a say in terms of the gender of the assessor?

12. What happens when the two assessors disagree?

13. What happens when the patient does not speak English or they are unable to speak?

Probe: If translators are required, what safeguards are in place to ensure they are accurately translating information to the patient?

Probe: What about patients who are unable to speak? Are there specific gestures that must be used to obtain consent? – non-verbal cue for the patients

14. Should a third assessment be required, what efforts are made to reduce the burden on the patient?

15. What does the third assessment look like and who is involved?

16. What happens when a patient wants MAiD but their family does not support it?

17. In general, how long does the assessment process take from start to finish for a Track 1 patient?

18. In general, how long does the assessment process take from start to finish for a Track 2 patient?

19. If the patient is deemed eligible for MAiD, is there a time by which MAiD must be delivered?

21. Who provides patient and family support and counselling throughout the assessment process to the time of death and thereafter?

Reporting before MAiD provision:

1. Who is required to report on findings from the assessments and how do they do that?

Probe: A. Does each assessor submit their form directly to Health Canada or does it go to someone at the provincial or regional level first?

B. Does the last assessor compile both assessments and then submit?

C. What is included in the information collected during the MAiD process (ask specific for (gender, race, Indigenous identity, disability)

D. Are patient aware of the reason why this information is being collected?

2. If an assessor finds a patient ineligible, who is required to report the finding to Health Canada?

3. If a patient is found eligible but it is later determined that safeguards are not met, who is required to report and what documentation is used to report this?

   Probe: What does this process look like when a patient withdraws their request after assessment?

   B. If a patient puts their MAiD request on hold after the assessments have taken place, how does this impact reporting? Does someone need to report that to Health Canada? Does reporting depend on the outcome of the assessment?

4. If a patient makes either a verbal or a written request for MAID and you begin your assessment, but the patient dies before you can complete it. What are you required to report and to whom?

   Probe: What is done with the patient's 'file' during this time? Are on-hold requests being tracked in [province/territory]?

5. What specific information are you required to report to them and how, regarding requests that verbal or by text and do not proceed to a formal assessment? Does the MAID coordination team that consulted you follow up regarding the outcome of all referrals?

Support provided to Patients

1. Do you ask questions about disability support available to patients and how do you determine the length of time of receiving this support?

2. If a patient has more than one serious incurable illness, disease or disability, how is it determined which illness, disease or disability is most responsible for that patient's suffering/leading to request?

3. For track 2 cases, what are assessors/providers required to report to Health Canada in terms of length of assessment? Are both required to report separately, or only one?

Provision of MaiD

1. Is it possible to deliver MAiD when and wherever the patient chooses?
    a. What about rural and remote communities?
    b. What about on reserves?
    c. What if the patient is incarcerated?
2. What happens if the patient resides in a facility that does not support MAiD?
3. What happens if a patient changes their mind at the last minute?
4. What steps are taken to ensure that the MAiD provider is aware of a patient's wishes in terms of loved ones they want present and activities that are carried out?
5. What steps are taken to ensure that those wishes are fulfilled?
6. What options for medications used in MAiD are available?
7. From where are those medications obtained?
   Probe: If hospital pharmacies only, does that affect access for patients who live in rural or remote areas and want to receive MAiD at home?
8. Who is responsible for reporting the dispensation of medications?
9. Who decides how MAiD will be administered? (provider vs self-administered)
10. Can you walk me through what's involved in the final appointment when MAiD is carried out?
    Probe: Does appointment look different depending on whether a patient chooses self-administered or provider administered MAiD?
11. What happens when a patient has no family or friends to be with them during MAiD?
    Probe: Are there time factors that need to be considered in terms of when medications must be administered once the provider has set everything up?
12. How are requests to incorporate religious, ethnic or cultural traditions managed? Could you provide some examples?
13. Who is involved in making arrangements relating to the care of a patient's remains following MAiD?
    Probe: Does it differ depending on whether MAiD takes place in a facility or a private

residence?

Reporting after MAiD Provision

1. We understand that there are various MAID reporting requirements of provinces and territories, some of which are quite specific. In your province or territory, how do you ensure that those requirements are met for each case?
2. Can you clarify whether providers and assessors are responsible for reporting MAiD to Health Canada in [province/territory], or does a provincial body assumes this responsibility? Depending on the response (Scenarios), use the under listed questions.

Scenario 1: Providers and assessors responsible for reporting MAiD to Health Canada

A. What specific information are you required to report to Health Canada and how?

Probe: Does the required information change if the provider/assessor decides not to proceed with the formal assessment? If yes, what information is required?

B. Does the MAID coordination team follow up regarding the outcome of all referrals?
C. What are the reporting deadlines in [province/territory]?

Scenario 2: Provincial body responsible for reporting to Health Canada

A. How does your province align its reporting requirements with the federal regulations, and are there any specific variations or additional requirements? If yes, could you describe what the variations are?
B. What specific information is the provincial body required to report to Health Canada regarding MAiD?
C. What is the role of the MAiD coordination team in the reporting process? Does anyone review the data from assessors/providers to ensure completeness and regulatory compliance?
D. What are the reporting deadlines for [province/territory] to Health Canada?
E. How do you receive information from preliminary assessors, providers, assessors, and pharmacists?

3. What are the policies regarding self-administration in [province/territory], and within what timeframe are providers required to report a patient's death resulting from self-administration?

4. Are providers/assessors actively required to seek out information if a patient has died from causes other than MAiD in [province/territory]?

Pharmacy Reporting

1. In [province/territory], what role do pharmacists play in terms of documentation and reporting?

   Probe:

   A. Do pharmacists report directly to Health Canada or to a provincial body responsible for reporting?
   B. Who is responsible for verifying and signing off on the substance once it is prepared for MAID?

2. If a person withdraws their request for MAiD on the date of provision and medications are not administered, is this required to be reported to Health Canada?

Support and bereavement

1. To what extent is the program involved in helping the family navigate next steps once their loved one has passed away?
2. Are arrangements made with a funeral home prior to the MAiD appointment?
3. What supports are available to the family after their loved one passes away?
4. Who in the MAiD team is involved in providing grief and bereavement support?
5. What happens when there is no family to handle arrangements after MAiD has been carried out?

Patients who are Indigenous

1. Does your program have dedicated resources or supports for Indigenous populations?
2. How does the program work with Indigenous patients and families to provide MAiD in a way that honors their beliefs and values?

3. Are you aware of any barriers to accessing MAiD for Indigenous patients? If yes, please describe them.
4. In your view, what should be done to reduce or even remove those barriers?
5. If there doesn't appear to be any barriers, what do you think has enabled your program to achieve such a success?

Patients with specific cultural, ethnic or religious beliefs

1. Has your program experienced any challenges when working with families with specific cultural, ethnic or religious beliefs?
    a. Would you be able to share some of those challenges and how they were managed?
2. Has your program experienced any particular successes when working with families with specific cultural, ethnic or religious beliefs? Would you be able to share some of those successes?

Patients who are unhoused/experiencing homelessness

1. How does a patient who is unhoused or experiencing homelessness access the MAiD program?
2. What are some of the challenges involved in carrying out consultations, assessments, and if determined appropriate, the provision of MAiD? How have these challenges been managed?
3. What do you think could be done to improve access for this population?

Patients who are incarcerated

1. How does someone who is incarcerated access the MAiD program?
2. How are consultations, assessments and if appropriate, the provision of MAiD carried out?
3. To your knowledge, has the MAiD program ever received a referral from an incarcerated patient? If yes, to what extent did the process differ from that for patients who are not incarcerated?
4. What are some of the challenges involved in carrying out consultations, assessments and, if determined appropriate, MAiD in this population?
5. What could be done to address those challenges?

6. If an incarcerated patient is deemed eligible for MAiD and chooses to proceed with it, is there an opportunity for loved ones to be present and/or special activities or ceremonies to be performed?
7. If an inmate/patient in a correctional facility explicitly requests MAiD, who is responsible for reporting to the province/Health Canada?

Funding

1. How are MAiD assessors and providers remunerated?
2. How is travel to remote or rural communities compensated?
3. How are appointments in the evenings and weekends compensated?
4. In your view, to what extent does financial compensation play a role in healthcare providers' willingness to be part of the MAiD program?

Wrap up:

1. Is there anything else you would like to share?
2. If I have additional questions or would like to clarify anything, can I reach out to you?
3. Is there anyone else you think we should reach out to?
    A. If yes, could you please ask them if you can share their contact information with us?

Thank you again for taking the time to participate in this project. Your insights are greatly appreciated.
