# Supplementary material for: Descriptions and Experiences with Medical Assistance in Dying Models Across Canada: A Mixed Methods Study
Source: Healthcare (Basel). 2026 Mar 20;14(6):797. doi: 10.3390/healthcare14060797 (PMC13027146; doi:10.3390/healthcare14060797)
Supplement: Supplementary file 1 [file healthcare-14-00797-s001.zip › S6-MAiD Services Coding Framework.pdf]

Supplementary Material S6: MAiD Services Coding Framework

A. Access and eligibility pathway

A1. Entry point / referral source

- *Definition:* How a person first connects with MAiD (self-referral, clinician referral, family inquiry).
- *Include:* “My family doctor referred me…” “We called the MAiD line…”
- *Exclude:* general comments about awareness (use A2).

A2. Awareness and information clarity

- *Definition:* Understanding of MAiD options, process, timelines, and what to expect.
- *Include:* confusion, misinformation, clarity of explanations, health literacy supports.
- *Exclude:* consent/capacity discussions (use B1).

A3. Eligibility criteria and assessment logistics

- *Definition:* How eligibility is assessed and organized (number of assessors, scheduling, documentation).
- *Include:* “second assessor was delayed,” “paperwork,” “assessment visit.”
- *Exclude:* emotions about waiting (use A4) unless tied to logistics.

A4. Timeliness and wait-related impacts

- *Definition:* Delays, urgency, pacing, and consequences of waiting (symptom burden, distress, loss of capacity).
- *Include:* “We were afraid she’d lose capacity before the date.”
- *Exclude:* symptom management details unless linked to wait (else D2).

A5. Equity and barriers

- *Definition:* Barriers related to geography, language, disability, socioeconomic status, housing, digital access, stigma, etc.
- *Include:* rural access, travel, interpreter needs, cultural barriers.
- *Exclude:* values-based conflict from providers (use F2).

B. Decision-making, consent, and safeguards

B1. Capacity and informed consent process

- *Definition:* How capacity is assessed and how informed consent is supported.
- *Include:* discussions about understanding, voluntariness, re-confirmation.

- *Exclude:* family disagreement (use B3).

B2. Voluntariness and coercion safeguards

- *Definition:* Safeguards to ensure the request is voluntary and free from pressure.
- *Include:* private conversations, screening for pressure.
- *Exclude:* general family involvement (use B3).

B3. Family involvement and dynamics

- *Definition:* Role of family/friends (supportive, conflicted, excluded, communication issues).
- *Include:* conflict, alignment, decision-support.
- *Exclude:* bereavement after death (use E3).

B4. Alternatives and supports discussed

- *Definition:* Conversations about palliative care, symptom management, psychosocial supports, spiritual care.
- *Include:* hospice referral, counseling offered.
- *Exclude:* quality of symptom control (use D2).

C. Care coordination and continuity

C1. Coordination roles and handoffs

- *Definition:* Who coordinates care and how handoffs occur (MAiD coordinator, nursing, pharmacy, home care).
- *Include:* missed communications, smooth transitions.

C2. Interprofessional collaboration

- *Definition:* Teamwork across disciplines/sectors (acute care, community, hospice, LTC).
- *Include:* "home care nurse and MAiD team coordinated."
- *Exclude:* conflict about conscientious objection (use F2).

C3. Documentation and communication quality

- *Definition:* Completeness/clarity of documentation and communication with patient/family/team.
- *Include:* unclear notes, duplicate forms, privacy issues.
- *Exclude:* legal/ethics policy concerns (use F1).

D. Clinical care experience

D1. Symptom burden and clinical complexity

- *Definition:* Complexity that shapes planning (frailty, cognitive changes, rapid decline, comorbidities).
- *Include:* “declined quickly,” “complex pain control.”
- *Exclude:* pure emotional distress (use E1).

D2. Symptom management and comfort measures

- *Definition:* Comfort-focused care before MAiD and on the day (palliative supports, medications, nursing comfort care).
- *Include:* management that affects experience and timing.
- *Exclude:* medication preparation steps in detail (keep high-level; use D3).

D3. MAiD procedure logistics

- *Definition:* Practical planning for the provision (location, equipment, pharmacy coordination, scheduling).
- *Include:* “pharmacy delivery,” “setting up at home.”
- *Exclude:* graphic or step-by-step procedural description.

D4. Setting and environment

- *Definition:* Location and atmosphere (home, hospice, hospital, LTC) and how it affected experience.
- *Include:* privacy, noise, room availability, staff interruptions.
- *Exclude:* family grief after (use E3).

E. Psychosocial, cultural, and emotional dimensions

E1. Emotional experience of patient

- *Definition:* Fear, relief, control, uncertainty, meaning, dignity, suffering (as described).
- *Include:* “felt at peace,” “anxious about timing.”

E2. Emotional experience of family/caregivers

- *Definition:* Family distress, relief, moral tension, caregiver strain (before the death).
- *Exclude:* bereavement follow-up (use E3).

E3. Bereavement and aftercare supports

- *Definition:* Supports offered/used after the death (follow-up calls, grief resources, cultural supports).

E4. Cultural/spiritual considerations

- *Definition:* Spiritual care needs, cultural safety, beliefs affecting decisions or care planning.
- *Include:* ceremonies, Elders, faith leader support, culturally safe communication.

F. Ethics, law, and values in practice

F1. Policy/legal navigation

- *Definition:* How policies, regulations, or institutional rules shape the pathway (paperwork, site rules).
- *Include:* “policy required transfer,” “institution rules delayed it.”

F2. Conscientious objection and moral distress

- *Definition:* Provider refusal, access pathways to alternate providers, team moral distress.
- *Include:* respectful objection, conflict, impacts on continuity.

F3. Safety, quality concerns, and incident triggers

- *Definition:* Any safety/quality concern raised (communication failures, near-misses, lack of supports).
- *Keep descriptive:* don’t assign blame in coding—capture what happened and perceived contributors.

G. System capacity and improvement opportunities

G1. Staffing and service capacity

- *Definition:* Workload, coverage gaps, availability of assessors/providers, on-call structures.

G2. Training and competency supports

- *Definition:* Training needs, mentorship, comfort with process, education gaps.

G3. Resource needs (practical)

- *Definition:* Transportation, equipment, interpreter access, space, IT systems, funding supports.

G4. Suggestions for improvement

- *Definition:* Explicit recommendations from participants (what would make it better).
- *Tip:* Also capture “implicit improvements” but keep them clearly labeled.
