# Supplementary material for: Descriptions and Experiences with Medical Assistance in Dying Models Across Canada: A Mixed Methods Study
Source: Healthcare (Basel). 2026 Mar 20;14(6):797. doi: 10.3390/healthcare14060797 (PMC13027146; doi:10.3390/healthcare14060797)
Supplement: Supplementary file 1 [file healthcare-14-00797-s001.zip › S7-MAiD Categorized Tables of Results final .pdf]

Supplementary Material S7: MAiD Categorized Tables of Results

*Interview content is identified in the tables using participant codes beginning with 'P00"

**Literature review content is identified using a using a numerical citation system with a corresponding reference list compiled from the reviewed reports

Table 1: Organization overview

Jurisdiction	Where is the MAiD program located within the organizational structure?	Organization			Who are the members of the MAiD team?
		**Is it centralized, and how?**	**Is it decentralized, and how?**	**Is it hybrid, and how?**	
Alberta - *Alberta Health Services*	• Continuing Care (P00B6, P00A5)	• Centralized (Provincially)<br>○ Central point of access and coordination of referrals and services (P00A5, P00B9, P00B13, P00C10) (2-5)	• Not applicable	• Not applicable	• Provincial MAiD team (Central MAiD Coordination office)<br>○ Provincial Medical Director, Provincial Seniors Health and Continuing Care/MAiD Lead<br>○ Provincial Medical Lead, MAiD (P00A5)<br>○ Office administrator (P00A5)<br>• Regional MAiD team (Regional (zone) MAiD Coordination offices)<br>○ Program manager (P00B13)<br>○ Regional Medical Lead (P00C39, P00B9, P00B13)<br>○ Coordinators (Nurse navigators) (P00B13, P00A5)<br>▪ Registered nurses (P00B13, P00A5)<br>▪ Emergency medical specialist (Calgary zone) (P00B13)<br>○ Office administrator (P00B13, P00A5)
British Columbia - *Ministry of Health*	• Not applicable	• Not applicable	• Decentralized (Provincially)<br>○ Multiple points of access and coordination of referrals and services (P00A4)<br>○ Regional MAiD offices are responsible for coordinating their respective MAiD processes (P00A4)	• Not applicable	• Not applicable
British Columbia - *Fraser Health*	• Home and Community Care Regional Services (P00A10)	• Centralized (Regionally)<br>○ Central point of access and coordination of referrals and services (6)	○ Not applicable	• Not applicable	• Medical Director, MAiD (P00A10)<br>• Manager/Lead (P00A10)<br>• Clinical coordinators (P00A10)<br>○ Registered nurses<br>○ Social workers<br>• Mental health clinician/clinical coordinator (P00A10)<br>○ Social worker (P00A10)<br>• Office administrator coordinator (P00A10)<br>• Nurse practitioner (P00A10)
British Columbia - *Interior Health*	• Palliative Care and End of Life Program (P00B14)	• Centralized (Regionally)<br>○ Central point of access and coordination of referrals and services (P00B14, P00C29)	• Not applicable	• Not applicable	• Director, Palliative care and end of life services (P00B14)<br>• Senior Medical Director, Palliative care and MAiD (P00B14)<br>• Regional Medical Director, Palliative care and MAiD (P00B14)<br>• Coordinators (P00B14)<br>○ Registered nurses (P00B14)<br>○ Social workers (P00B14)
British Columbia - *Island Health*	• Palliative care and End of Life Program (P00A13)	• Not applicable	• Not applicable	• Hybrid (Regionally)<br>○ Central point of access and coordination of referrals and services (P00A9, P00A13)<br>○ Alternate points of access and coordination of referrals and services<br>▪ Physicians in smaller communities have established communities of practice to coordinate services (P00A13)	• Medical Director, MAiD (P00A9)<br>• Program manager (P00A9)<br>• Coordinators (P00A9)<br>○ Registered nurses (P00A9)<br>• Office administrator (P00A9)<br>• Nurse practitioners (P00A9)<br>• Registered nurse (P00A9)<br>• Research assistant (P00A9)

Jurisdiction	Where is the MAiD program located within the organizational structure?	Organization			Who are the members of the MAiD team?
		Is it centralized, and how?	Is it decentralized, and how?	Is it hybrid, and how?	
British Columbia - *Northern Health*	• Northern Health Medical Advisory Committee (P00A8)	• Centralized (Regionally)<br>○ Central point of access and coordination of referrals and services (P00A8)	• Not applicable	• Not applicable	• Medical Director, MAiD (P00A8)<br>• Coordinator (P00A8)<br>○ Social worker (P00A8)
British Columbia - *Vancouver Coastal Health*	• Quality and Patient Safety (P00A7, P00B15, P00B16)	• Centralized (Regionally)<br>○ Central point of access and coordination of referrals and services (P00A7, P00B15, P00B12)	• Not applicable	• Not applicable	• Medical Director, MAiD (P00B10)<br>• Director, MAiD program (P00A7)<br>• Clinical operations manager (P00B10)<br>• Coordinators (P00B12)<br>○ Registered nurses (P00A7)<br>○ Social worker (P00A7)<br>• Indigenous advisor (P00A7, P00B16)
British Columbia - *Provincial Health Services Authority*	• Risk Portfolio (P00A11)<br>○ Transitioning to Medical Affairs (P00A11)	• Centralized (Provincially)<br>○ Central point of access and coordination of referrals and services (P00A11)	• Not applicable	• Not applicable	• Medical Director, MAiD (P00A11)<br>• Coordinator (Risk director) (P00A11)<br>○ Registered nurse (P00A11)<br>• Project manager (P00A11)<br>○ Registered nurse (P00A11)
Manitoba - *Shared Health*	• Shared Health (P00B19, P00B21)	• Centralized (Provincially)<br>○ Central point of access and coordination of referrals and services (7) (P00B21, P00C33)	• Not applicable	• Not applicable	• Medical Director, MAiD (P00C33, P00C34)<br>• Program manager (MAiD team manager) (P00C34)<br>• Coordinators (P00B19, P00C34, P00B21)<br>○ Registered nurses (P00B19, P00C34, P00B21)<br>• Office administrator (P00C33)<br>• Nurse practitioner (P00C33)<br>• Social workers (8)
New Brunswick - *Horizon Health Network*	• Risk Management (P00B11, P00C15)<br>○ Transitioning to Clinical Services (P00C15)	• Centralized (Regionally)<br>○ Central point of access and coordination of referrals and services (P00B11, P00C15)	• Not applicable	• Not applicable	• Medical Director, Palliative care service and MAiD (P00B11)<br>• Coordinator (Regional lead) (P00B11)<br>○ Registered nurse (P00B11) (9)<br>• Administrative support (P00B11)
New Brunswick - *Vitalité Health Network*	• Risk Management (P00C48)	• Not applicable	• Decentralized (Regionally)<br>○ Multiple points of access and coordination of referrals and services (P00C48)<br>○ Practitioners are responsible for coordinating their respective MAiD processes (P00C48)	• Not applicable	• No Regional MAiD team (P00C48)
Newfoundland and Labrador - *Department of Health and Community Services*	• Not applicable	• Not applicable	• Decentralized (Provincially)<br>○ Multiple points of access and coordination of referrals and services (10)<br>○ Regional MAiD offices are responsible for coordinating their respective MAiD processes (10)	• Not applicable	• Not applicable
Newfoundland and Labrador - *Eastern Zone*	• Palliative Care and End of Life Program (P00C43)	• Centralized (Regionally)<br>○ Central point of access and coordination of referrals and services (P00C43) (10)	• Not applicable	• Not applicable	• Medical Director, MAiD (P00C43)<br>• Coordinator (P00C43)<br>○ Registered nurse (P00C43)
Newfoundland and Labrador - *Western Zone*	• Palliative Care Department (P00C44)<br>○ Undergoing restructuring (P00C44)	• Centralized (Regionally)<br>○ Central point of access and coordination of referrals and services (P00B44, P00C44)	• Not applicable	• Not applicable	• Coordinator (P00C44)<br>○ Administrative assistant (P00C44)
Newfoundland and Labrador - *Central Zone*	• Home and Community Nursing - Palliative Care (P00B22)	• Centralized (Regionally)<br>○ Central point of access and coordination of referrals and services (P00B22)	• Not applicable	• Not applicable	• Palliative and end of life program manager (P00B22)<br>• Coordinator (P00B22)<br>○ Social worker (P00B22)<br>• Clerk (P00B22)<br>• Palliative care physician (P00B22)

Jurisdiction	Where is the MAiD program located within the organizational structure?	Organization: Is it centralized, and how?	Organization: Is it decentralized, and how?	Organization: Is it hybrid, and how?	Who are the members of the MAiD team?
Northwest Territories	• Continuing Care (P00A3)	• Centralized (Territorially)<br>○ Central point of access and coordination of referrals and services (P00A2) (11)	• Not applicable	• Not applicable	• Clinical lead, MAiD (P00A3)<br>○ Physician (P00B24)<br>• Coordinator (Territorial MAiD Specialist) (P00A2, P00A3)<br>○ Registered nurse (P00A2, P00A3)
Nova Scotia - *Nova Scotia Health*	• Medicine and Clinical Care Network (P00A6)	• Centralized (Provincially)<br>○ Central point of access and coordination of referrals and services (P00A6, P00A12, P00C38)	• Not applicable	• Not applicable	• Senior Director, MAiD and palliative care networks (P00A6)<br>• Clinical lead, MAiD (P00A6)<br>○ Physician (P00A6)<br>• MAiD program lead (P00A6)<br>• Coordinators (Nurse navigators) (P00A12)<br>○ Registered nurses (P00A12)<br>• Office administrator (Unit clerk) (P00A12)<br>• Nurse practitioners (12-14)<br>• Social workers (P00A12, P00A6)
Prince Edward Island - *Health PEI*	• Community and Primary Care Division (P00B17)	• Centralized (Provincially)<br>○ Central point of access and coordination of referrals and services (P00B8, P00B17)	• Not applicable	• Not applicable	• Clinical lead, MAiD (P00B8)<br>○ Physician (P00B8)<br>• Coordinator (P00B8)<br>○ Registered nurse (P00B8)<br>• Office administrator (P00B8, P00B17)<br>• Physicians (P00B8, P00B17)<br>• Social worker (P00B8)<br>• Nurse practitioners (P00B8, P00B17)
Saskatchewan - *Saskatchewan Health Authority*	• Community Care Services (P00B1, P00A1)	• Centralized (Provincially)<br>○ Central point of access and coordination of referrals and services (P00A1, P00B1, P00C1)	• Not applicable	• Not applicable	• Director, Provincial Health line and MAiD program (P00B1)<br>• Medical Director, MAiD (P00B1)<br>• Program manager (P00B1)<br>• Coordinators (P00A1, P00B1, P00C1)<br>○ Registered nurses (P00A1)<br>• Office Administrator (P00B1, P00A1)<br>• Social workers (P00A1)<br>• Nurse practitioners (P00B3, P00C19)<br>• Health information management practitioner (P00A1)
Yukon - *Department of Health and Social Services*	• Continuing care (P00C14)	• Not applicable	• Decentralized (Territorially)<br>○ Multiple points of access and coordination of referrals and services (15)<br>○ Practitioners are responsible for coordinating their respective MAiD processes (P00C14)	• Not applicable	• No territorial MAiD team
Ontario - *Ministry of Health*	• Not applicable	• Not applicable	• Decentralized (Provincially)<br>○ Multiple points of access and coordination of referrals and services (P00B4, P00C16)<br>○ Regional institutions/practitioners are responsible for coordinating their respective MAiD processes (P00B4)	• Not applicable	• Manager (P00C16)<br>• Coordinators (P00C16)<br>○ Nurse practitioner (P00C16)<br>○ Registered nurses (P00C16)
SERVICE ORGANIZATIONS/REGIONAL FACILITIES					
Ontario - *Home and Community Care Support Services, Central East*	• Home and Community Care Support Services (P00C2)	• Centralized (Regionally)<br>○ Central point of access and coordination of referrals and services (P00C2)	• Not applicable	• Not applicable	• Program manager (P00C2)<br>• Coordinator (P00C2)<br>• Technical assistants (P00C2)
Ontario - *Home and Community Care Support Services, Waterloo Region*	• Undergoing restructuring (P00C4)	• Centralized (Regionally)<br>○ Central point of access and coordination of referrals and services (P00C4)	• Not applicable	• Not applicable	• Coordinators (Nurse navigators) (P00C4)<br>○ Registered nurses (P00C4)

Jurisdiction	Where is the MAiD program located within the organizational structure?	Organization			Who are the members of the MAiD team?
		Is it centralized, and how?	Is it decentralized, and how?	Is it hybrid, and how?	
Ontario - *Home and Community Care Support Services, South East*	• Home and Community Care Support Services (P00C20)	• Centralized (Regionally)<br>○ Central point of access and coordination of referrals and services (P00C20)	• Not applicable	• Not applicable	• Coordinators (P00C20)<br>○ Registered psychiatric nurses (P00C20)
Ontario - *Home and Community Care Support Services, South West*	• Home and Community Care Support Services (P00C23)	• Centralized (Regionally)<br>○ Central point of access and coordination of referrals and services (P00C23)	• Not applicable	• Not applicable	• Coordinators (P00C23)<br>○ Nurse practitioner (P00C23)<br>○ Registered nurse (P00C23)
Ontario - *Champlain Regional MAiD Network, The Ottawa Hospital*	• Director of Ethics at the Ottawa Hospital (P00C5)	• Centralized (Regionally)<br>○ Central point of access and coordination of referrals and services (P00C5)	• Not applicable	• Not applicable	• Advanced practice nurse and program manager (P00C5)<br>• Coordinators (Nurse navigators) (P00C5)<br>○ Registered nurses (P00C5)<br>• Physicians (P00C5)<br>• Nurse practitioners (P00C5)<br>• Administrative assistant (P00C5)
HEALTHCARE FACILITIES					
Ontario - *Peterborough Regional Health Centre*	• Director of Medical Affairs (P00C3)	• Centralized (Institutionally)<br>○ Central point of access and coordination of referrals and services (P00C3)	• Not applicable	• Not applicable	• Coordinator (Patient relations consultant) (P00C3)<br>○ Registered nurse (P00C3)<br>• Physicians (P00C3)<br>• Nurse practitioner (P00C3)<br>• Social worker (P00C3)
Ontario - *Mount Sinai Healthcare Facility, Toronto*	• Medical Advisory Committee (P00C36)	• Centralized (Institutionally)<br>○ Central point of access and coordination of referrals and services (P00C36)	• Not applicable	• Not applicable	• Coordinators (P00C36)<br>○ Registered nurses (P00C36)<br>○ Bioethicist (P00C36)
Ontario - *University Health Network*	• Department of Anesthesia and Pain Services (P00C8)	• Centralized (Institutionally)<br>○ Central point of access and coordination of referrals and services (P00C8)	• Not applicable	• Not applicable	• Medical Director, MAiD (P00C8)<br>• Coordinators (P00C8)<br>○ MAiD clinical nurse specialist (P00C8)<br>▪ Advanced practice nurse (P00C8)<br>○ MAiD clinical coordinator (P00C8)<br>▪ Registered nurse (P00C8)<br>• Administrative assistant (P00C8)<br>• Physicians (P00C8)<br>• Nurse practitioners (P00C8)
Ontario - *Grand River Hospital*	• Quality and Patient Relations (P00C50)	• Centralized (Institutionally)<br>○ Central point of access and coordination of referrals and services (P00C50)	• Not applicable	• Not applicable	• Coordinator (P00C50)<br>○ Nurse practitioner (P00C50)<br>• Physicians (P00C50)
COMMUNITY OF PRACTICE					
Ontario - *Hamilton Family Health Team*	• Hamilton Family Health Team (P00C32)	• Centralized (Regionally)<br>○ Central point of access and coordination of referrals and services (P00B18)	• Not applicable	• Not applicable	• Manager (P00B18)<br>• Coordinators (P00B18)<br>○ Registered nurse (P00B18)<br>○ Registered practical nurse (P00B18)<br>• Mental Health Nurse (P00B18)<br>• Physicians (P00B18)
Ontario - *Niagara Community MAiD Team, St. Catharine's, Niagara*	• Niagara Community MAiD Team (P00C11)	• Not applicable	• Decentralized (Regionally)<br>○ Multiple points of access and coordination of referrals and services (P00C11)<br>○ Practitioners are responsible for coordinating their respective MAiD processes (P00C11)	• Not applicable	• No MAiD team
Québec - *Ministry of Health and Social Services*	• Not applicable	• Not applicable	• Decentralized (Provincially)<br>○ Multiple points of access and coordination of referrals and services (P00C40)<br>○ Individual health establishments are responsible for coordinating their respective MAiD processes (P00C40)	• Not applicable	• Not applicable

Jurisdiction	Where is the MAiD program located within the organizational structure?	Organization			Who are the members of the MAiD team?
		Is it centralized, and how?	Is it decentralized, and how?	Is it hybrid, and how?	
INTEGRATED HEALTH AND SOCIAL SERVICES CENTRES					
Québec – *CISS Montérégie*	• Not specified	• Centralized (Regionally)<br>○ Central point of access and coordination of referrals and services (P00C27)	• Not applicable	• Not applicable	• Not specified
INTEGRATED UNIVERSITY HEALTH AND SOCIAL SERVICES CENTRES					
Québec – *CIUSS Capitale-Nationale*	• Not specified	• Centralized (Regionally)<br>○ Central point of access and coordination of referrals and services (16)	• Not applicable	• Not applicable	• Not specified
Québec – *University of Montreal Hospital Center*	• Not specified	• Centralized (Institutionally)<br>○ Central point of access and coordination of referrals and services (P00C40)	• Not applicable	• Not applicable	• MAiD Coordinator<br>○ Registered nurse (P00C40)
Québec – *McGill University Health Centre*	• Cancer care, supportive and palliative care (P00C9)	• Centralized (Institutionally)<br>○ Central point of access and coordination of referrals and services (P00C9)	• Not applicable	• Not applicable	• MAiD Coordinator (P00C9)<br>○ Advanced Practice Nurse (P00C9)<br>• Administrative assistant (P00C9)

Table 2: Relationship between Ministry of Health and MAiD program

Jurisdiction	What is the role of the Ministry of Health with the MAiD program?
Alberta – *Alberta Health*	• Provide policy oversight in delivery of MAiD services (P00A5, P00B6)<br>• Collaborate with Regulatory Review Committee to identify policy priorities (P00B6)
British Columbia – *Ministry of Health*	• Develop policies and guidelines to govern delivery of MAiD services (P00A4)<br>• Coordinate MAiD Oversight Advisory Committee to enhance delivery of MAiD services (P00A4)<br>• Develop and provide provincial standardized forms for MAiD requests, assessments and provisions (P00A4)<br>• Oversee reporting of MAiD assessments and provisions through MAiD Oversight Unit (P00A4)
Manitoba – *Manitoba Health*	• Provide policy oversight in delivery of MAiD services (P00B19)
New Brunswick – *Department of Health*	• Provide policy oversight in delivery of MAiD services (P00C15, P00C48)
Newfoundland and Labrador - *Department of Health and Community Services*	• Provide policy oversight in delivery of MAiD services (P00C44)<br>• Collaborate with provincial MAiD working group to identify policy priorities (P00C43, P00C44, P00B22)
Northwest Territories – *Department of Health and Social Services*	• Develop policies and guidelines to govern delivery of MAiD services (P00A3)<br>• Develop and provide territorial standardized forms for MAiD requests, assessments and provisions (P00A3)<br>• Oversee reporting of MAiD assessments and provisions through MAiD Review Committee (P00A3)
Nova Scotia – *Department of Health and Wellness*	• Provide policy oversight in delivery of MAiD services (P00A12)
Prince Edward Island – *Ministry of Health*	• Develop policies and guidelines to govern delivery of MAiD services (P00B8)
Saskatchewan – *Ministry of Health*	• Develop policies and guidelines to govern delivery of MAiD services (P00A1, P00B3)<br>• Collaborate with Quality and Safety Committee to enhance delivery of MAiD services (P00A1)
Yukon - *Department of Health and Social Services*	• Develop policies and guidelines to govern delivery of MAiD services (P00C14)<br>• Develop and provide territorial standardized forms for MAiD requests, assessments and provisions (P00C14)<br>• Coordinate MAiD working group to enhance delivery of MAiD services (P00C14)
Ontario - *Ministry of Health*	• Support coordination of care for patients through provincial care coordination services (P00B4)<br>○ For patients who do not access MAiD services directly from their healthcare provider (P00B4)<br>• Develop policies and guidelines to govern delivery of MAiD services (P00B4)<br>• Develop and provide voluntary provincial standardized forms for MAiD requests, assessments and provisions (P00B4)
Québec – *Ministry of Health and Social Services*	• Develop policies and guidelines to govern delivery of MAiD services (17)<br>• Coordinate Commission on end-of-life to enhance delivery of MAiD services (17)<br>• Develop and provide provincial standardized forms for MAiD requests, assessments and provisions (17)<br>• Oversee reporting of MAiD assessments and provisions through Commission on end-of-life (17)

Table 3: Program oversight and monitoring

Jurisdiction	Program oversight		How is the MAiD program monitored?
	**What is the role of the oversight team/committee?**	**Who are the members of the oversight team/committee?**	
Alberta - *Alberta Health Services*	• MAiD oversight team (MAiD Regulatory Review Committee)<br>○ Address policy issues and develops recommendations (P00A5, P00B6)<br>○ Review case files that have concerns (P00A5)<br>○ Provide recommendations to Alberta Health Services (P00A5)	• MAiD Regulatory Review Committee<br>○ Alberta College of Pharmacy (P00A5)<br>○ Ministry of Health (P00A5)<br>○ College of Registered Nurses of Alberta (CRNA) (P00A5)<br>○ College of Physicians and Surgeons of Alberta (CPSA) (P00A5)<br>○ Chief Medical Examiner (P00A5)	• Provincial MAiD team (Central MAiD Coordination office)<br>○ Oversee coordination of MAiD services (P00A5)<br>○ Review forms for accuracy and completeness (P00B9, P00C10, P00C13)<br>○ Compare and validate MAiD deaths forms with Chief Medical Examiner (P00A5)
British Columbia - *Ministry of Health*	• MAiD oversight team (MAiD Oversight Advisory Committee)<br>○ Provides advice to Ministry of Health regarding oversight-related matters<br>▪ Advisory on policies and standards (P00A4)	• MAiD Oversight Advisory Committee<br>○ Professional colleges (P00A4)<br>○ MAiD providers (P00A4)<br>○ Pharmacy representatives (P00A4)<br>○ Health Authority representatives (P00A4)	• MAiD Program Oversight unit<br>○ Review forms for accuracy and completeness and compliance with all applicable legislation, regulation, and standards (P00B14)<br>○ Create reports on MAiD data and send them to MAiD Oversight Advisory Committee (P00A4)
British Columbia - *Fraser Health*	• MAiD oversight team (MAiD steering committee and Regional MAiD team)<br>○ Develop MAiD processes and policies for health authority (P00A10)<br>○ Support assessors and providers in delivery of MAiD services (P00A10)	• MAiD steering committee<br>○ Members not specified<br>• Regional MAiD team<br>○ Medical Director, MAiD (P00A10)<br>○ Manager/lead (P00A10)<br>○ Clinical coordinators (P00A10)	• Regional MAiD team<br>○ Oversee coordination of MAiD services in region (P00A10)<br>○ Review forms for accuracy and completeness before submitting to provincial oversight unit (P00A10)
British Columbia - *Interior Health*	• MAiD oversight team (Regional MAiD team)<br>○ Develop MAiD standards and processes for health authority (P00B14)<br>○ Provide resources for assessors and providers (P00B14)	• Regional MAiD team<br>○ Director, Palliative and end of life services (P00B14)<br>○ Senior Medical Director, Palliative care and MAiD (P00B14)<br>○ Regional Medical Director, Palliative care and MAiD (P00B14)<br>○ Coordinators (P00B14)	• Regional MAiD team<br>○ Oversee coordination of MAiD services in region (P00B14)<br>○ Review forms for accuracy and completeness (P00B14)<br>○ Care coordination services keep record of assessed patients (P00C29)
British Columbia - *Island Health*	• MAiD oversight team (Regional MAiD team)<br>○ Develop MAiD standards and processes for health authority (P00A13)<br>○ Provides resources for assessors and providers (P00A9)<br>○ Support assessors and providers in delivery of MAiD (P00A9)	• Regional MAiD team<br>○ Medical Director, MAiD (P00A9)<br>○ Program manager (P00A9)<br>○ MAiD coordinators (P00A9)<br>○ Administrative staff (P00A13)<br>○ Research assistant (P00A13)	• Regional MAiD team<br>○ Oversee coordination of MAiD services in region (P00A13)<br>○ Review forms for accuracy and completeness (P00A9)<br>○ Responsible for the quality of care through privileging clinicians, health authority facilities or employees (P00A13)<br>○ Healthcare sites, MAiD providers, and MAiD oversight unit send MAiD death records to care coordination service for statistical purposes and quality assurance (P00A13)<br>○ Assessors and providers send forms for documentation and record keeping (P00A9, P00A13)<br>▪ Information is uploaded into electronic health records (P00A9)
British Columbia - *Northern Health*	• MAiD oversight team (Regional MAiD team)<br>○ Develop MAiD standards and processes for health authority (P00A8)<br>○ Provide resources for assessors and providers (P00A8)	• Regional MAiD team<br>○ Medical Director, MAiD (P00A8)<br>○ MAiD coordinator (P00A8)	• Regional MAiD team<br>○ Oversee coordination of MAiD services (P00A8)<br>○ Medical Director, MAiD sits on provincial MAiD Oversight Advisory Committee (P00A8)
British Columbia - *Vancouver Coastal Health*	• MAiD oversight team (Regional MAiD team)<br>○ Develop MAiD standards and policies for health authority (P00A7)<br>○ Provide resources for assessors and providers (P00A7)	• Regional MAiD team<br>○ Director, MAiD program (P00A7)<br>○ Medical Director, MAiD (P00A7)<br>○ Clinical operations manager (P00A7)<br>○ Coordinators (P00A7)	• Regional MAiD team<br>○ Oversee coordination of MAiD service (P00A7, P00B10)<br>○ Review forms for accuracy and completeness (P00A7, P00B10, P00B16)<br>○ Medical Director, MAiD program director and clinical operations manager sit on provincial MAiD Oversight Advisory Committee (P00B10)
British Columbia - *Provincial Health Services Authority*	• No program oversight team or committee specified	• No program oversight team or committee specified	• No monitoring mechanisms or procedures specified
Manitoba - *Shared Health*	• MAiD oversight team (Provincial MAiD team)<br>○ Develop MAiD policies and processes (P00C34)<br>○ Develop standards for delivery of MAiD (P00C34)<br>○ Provides resources for assessors and providers (P00C34)<br>○ Support assessors and providers in delivery of MAiD (P00C34)	• Provincial MAiD team<br>○ Medical Director, MAiD (P00C34)<br>○ Program manager (P00C33)	• Provincial MAiD team<br>○ Oversee coordination of MAiD service (P00B19, P00C34)<br>○ Review forms for accuracy and completeness (P00B19)<br>▪ Uses actuarial system for tracking patients through different stages of MAiD (P00C34)

Jurisdiction	Program oversight		How is the MAiD program monitored?
	What is the role of the oversight team/committee?	Who are the members of the oversight team/committee?	
	○ Review cases to ensure compliance with federal legislation (P00C34)		
New Brunswick - *Horizon Health Network*	• MAiD oversight team (MAiD steering committee)<br>○ Committee is not currently active	• Regional MAiD team<br>○ Head, Regional Risk Management (P00B11)<br>○ Medical Director, Palliative care service and MAiD (P00B11)<br>○ Coordinator (Regional lead) (P00B11)	• Regional MAiD team<br>○ Oversee coordination of MAiD service (P00B19, P00C15)<br>• Risk Management team<br>○ Review forms for accuracy and completeness (P00B19, P00C15)
New Brunswick - *Vitalité Health Network*	• MAiD oversight team (Risk management committee)<br>○ Review MAiD assessment forms (P00C48)<br>○ Review cases to ensure compliance with federal legislation (eligibility and safeguards) (P00C48)	• Risk management committee<br>○ Committee members not specified	• Risk Management Committee<br>○ Review forms for accuracy and completeness (P00C48)<br>○ Approves assessments following confirmation of adherence to federal legislation (P00C48)
Newfoundland and Labrador - *Department of Health and Community Services*	• MAiD oversight team (Provincial MAiD working group)<br>○ Team is restructuring	• Provincial MAiD Working Group<br>○ Team is restructuring	• Not applicable<br>○ Not involved in MAiD service delivery
Newfoundland and Labrador - *Eastern Zone*	• MAiD oversight team (Regional MAiD team)<br>○ Develop MAiD policies and standards (P00C43)<br>○ Support providers and assessors in delivery of MAiD services (P00C43)	• Regional MAiD team<br>○ Medical Director, MAiD (P00C43)<br>○ Coordinator (P00C43)	• Regional MAiD team<br>○ Oversee coordination of MAiD services (P00C43)
Newfoundland and Labrador - *Western Zone*	• MAiD oversight team (Regional MAiD team)<br>○ Support providers and assessors in delivery of MAiD services (P00C44)	• Regional MAiD team<br>○ Coordinator (P00C44)	• Regional MAiD team<br>○ Oversee coordination of MAiD services (P00C44)
Newfoundland and Labrador - *Central Zone*	• MAiD oversight team (Regional MAiD team)<br>○ Support providers and assessors in delivery of MAiD services (P00B22)	• Regional MAiD team<br>○ Coordinator (P00B22)<br>○ Palliative care physician (P00B22)	• Regional MAiD team<br>○ Oversee coordination of MAiD services (P00B22)<br>○ Coordinator requests assessors and providers to send forms for review and documentation (P00B22)<br>○ Palliative care physician provides leadership support and clinical expertise (P00B22)<br>○
Northwest Territories	• MAiD oversight team (MAiD review committee)<br>○ Ensure compliance with safeguards and processes (P00A2, P00A3)<br>○ Responsible for reporting to Health Canada (P00A3)	• MAiD review committee<br>○ Nurses (P00A2) (11)<br>○ Social worker (P00A3)	• Territorial MAiD team<br>○ Oversee coordination of MAiD services (P00A4)<br>○ Review forms for accuracy and completeness(P00A2, P00A3) (11)
Nova Scotia - *Nova Scotia Health*	• MAiD oversight team (MAiD advisory committee)<br>○ Provide advice on the delivery of MAiD services<br>○ Provide recommendation on best practices across different jurisdictions<br>○ Develop performance indicators for monitoring MAiD program	• MAiD advisory committee (currently undergoing transitioning)<br>○ Senior Director, MAiD and palliative care networks (P00A12)<br>○ Clinical lead, MAiD (P00A12)<br>○ MAiD program lead (P00A12)<br>○ Physicians (P00A12)	• Provincial MAiD team<br>○ Oversee coordination of MAiD services (P00A12)<br>○ Collect forms for documentation (P00A12)<br>○ Provide reports to MAiD advisory committee (P00A12)
Prince Edward Island - *Health PEI*	• MAiD oversight team (Provincial MAiD team)<br>○ Develop processes and standards (P00B17)<br>○ Provide resources to assessors and providers (P00B17)<br>○ Review challenging cases with clinical advisory committee (P00B8)<br>▪ Developed a review process with Nova Scotia MAiD clinical team (P00B8)<br>○ Conduct retrospective chart review for standardization of documentation (P00B8)	• Provincial MAiD team<br>○ Clinical lead, MAiD (P00B17)<br>○ Clinical advisory committee (P00B8)<br>▪ Physicians (P00B8)<br>▪ MAiD coordinator (P00B17)<br>▪ Social worker (P00B8)	• Provincial MAiD team<br>○ Oversee coordination of MAiD services (P00B17)
Saskatchewan - *Saskatchewan Health Authority*	• MAiD oversight team (Quality and Safety Committee)<br>○ Provide quality control on MAiD polices, processes and standard (P00A1)	• Quality and safety committee<br>○ College of Physicians and Surgeons of Saskatchewan (P00A1, P00B1, P00B3)<br>○ College of Registered Nurses (P00A1, P00B1, P00B3)<br>○ College of Pharmacy (P00A1, P00B1, P00B3)<br>○ Director, MAiD program (P00A1, P00B1, P00B3)<br>○ Representative, Ministry of health (P00A1, P00B1, P00B3)	• Provincial MAiD team<br>○ Oversee coordination of MAiD services (P00B1, P00A1)<br>○ Review forms to ensure accuracy and completeness (P00A1)

Jurisdiction	Program oversight<br>What is the role of the oversight team/committee?	Who are the members of the oversight team/committee?	How is the MAiD program monitored?
		○ College of Registered Psychiatric Nurses (P00A1, P00B1, P00B3)<br>○ Director, Quality and safety (P00A1, P00B1, P00B3)<br>○ Ethicist (P00A1, P00B1, P00B3)<br>○ Legal counsel (P00A1, P00B1, P00B3)<br>○ Privacy officer (P00A1, P00B1, P00B3)<br>○ Patient family partners (P00B1)	
Yukon - *Department of Health and Social Services*	• MAiD oversight team (Working group)<br>○ Develop processes and standards (P00C14)<br>○ Develop standardized forms for reporting MAiD assessment and provision (P00C14)<br>○ Provide resources for MAiD assessors and providers (P00C14)	• Working group<br>○ Medical Council of Yukon (P00C14)<br>○ Registered Nurses Association (P00C14)<br>○ Physicians (P00C14)<br>○ Hospital corporation (P00C14)<br>○ First Nations representatives (P00C14)	• Independent privileged physicians/nurse practitioners oversee coordination of MAiD services (P00C14)
Ontario - *Ministry of Health*	• MAiD oversight team<br>○ Ministry of Health<br>▪ Responsible for strategy, legislation, and funding (P00B4)<br>▪ Support provincial care coordination service (P00B4)<br>○ Office of the Chief Coroner<br>▪ Oversee reporting of MAiD provisions (P00B20)	• Office of the Chief Coroner<br>○ MAiD Death Review Committee<br>▪ Representatives from disability advocacy groups (P00B20)<br>▪ Legal professionals (P00B20)<br>▪ Physicians (P00B20)<br>▪ Nurse practitioners (P00B20)<br>▪ Nurses (P00B20)<br>▪ Social worker (P00B20)<br>▪ Ethicists (P00B20)<br>▪ Public member (P00B20)<br>○ MAiD Review team<br>▪ Registered nurses (P00B20)<br>▪ Manager (P00B20)<br>▪ Administrative support (P00B20)	• Office of the Chief Coroner<br>○ Provide monthly and quarterly reporting to Ministry of Health and relevant stakeholders (P00B20)<br>○ Review all MAiD deaths to ensure legislative compliance and accuracy of Health Canada mandatory reporting (P00B20)
SERVICE ORGANIZATIONS/REGIONAL FACILITIES			
Ontario - *Home and Community Care Support Services, Central East*	• MAiD oversight team (Regional MAiD program)<br>○ Provide resources and guidance for assessors and providers (P00C2)<br>○ Develop MAiD standards, processes and policies (P00C2)	• Regional MAiD Program<br>○ Program manager (P00C2)	• Regional MAiD team<br>○ Oversee coordination of MAiD services (P00C2)<br>• Providers must report all death cases to MAiD review team for review (P00C2)
Ontario - *Home and Community Care Support Services, Waterloo Region*	• MAiD oversight team (MAiD committee)<br>○ Role not specified	• MAiD committee<br>○ Director of Medicine (P00C4)	• Providers must report all death cases to MAiD review team for review (P00C4)
Ontario - *Home and Community Care Support Services, South East*	• MAiD oversight team (Regional MAiD program)<br>○ Provide resources to assessors and providers (P00C20)	• Regional MAiD program<br>○ Manager (P00C20)	• Regional MAiD team<br>○ Oversee coordination of MAiD services (P00C20)<br>• Providers must report all death cases to MAiD review team for review (P00C20)
Ontario - *Home and Community Care Support Services, South West*	• MAiD oversight team (Regional MAiD program)<br>○ Develop MAiD standards and processes (P00C23)	• Regional MAiD program<br>○ MAiD coordinator (P00C23)	• Regional MAiD team<br>○ Oversee coordination of MAiD services (P00C16, P00C23)<br>• Providers must report all death cases to MAiD review team for review (P00C16, P00C23)
Ontario - *Champlain Regional MAiD Network, the Ottawa Hospital*	• MAiD oversight team (MAiD oversight team)<br>○ Provide resources and guidance for assessors and providers (P00C5)	• MAiD oversight team<br>○ Director of Ethics (P00C5)<br>○ Advanced practice nurse and program manager (P00C5)	• Institutional MAiD team<br>○ Oversee coordination of MAiD services (P00C5)<br>• Providers must report all death cases to MAiD review team for review (P00C5)
HEALTHCARE FACILITIES			
Ontario - *Peterborough Regional Health Centre*	• MAiD oversight team (Institutional MAiD program)<br>○ Develop MAiD standards and processes (P00C3)	• Institutional MAiD program<br>○ Director, Medical Affairs (P00C3)	• Institutional MAiD team<br>○ Oversee coordination of MAiD services (P00C3)<br>• Providers must report all death cases to MAiD review team for review (P00C3)
Ontario - *Mount Sinai Healthcare Facility, Toronto*	• MAiD oversight team (Steering committee)<br>○ Develop MAiD standards and processes and polices (P00C36)	• Steering committee<br>○ Vice President, Professional Practice (P00C36)<br>○ Medical Lead (P00C36)	• Institutional MAiD team<br>○ Oversee coordination of MAiD services (P00C36)

Jurisdiction	Program oversight: What is the role of the oversight team/committee?	Program oversight: Who are the members of the oversight team/committee?	How is the MAiD program monitored?
			• Providers must report all death cases to MAiD review team for review (P00C36)
Ontario - *University Health Network*	• MAiD oversight team (Institutional MAiD program)<br>○ Provide resources and guidance for assessors and providers (P00C8)	• Institutional MAiD program<br>○ Head of Anesthesia and Pain Services (P00C8)<br>○ Senior Director, Clinical and Organizational Ethics (P00C8)<br>○ Medical Director, MAiD (P00C8)<br>○ MAiD Clinical Nurse Specialist (P00C8)<br>○ MAiD Clinical Coordinator (P00C8)<br>○ Director, Professional Practice (P00C8)<br>○ Manager, Professional Practice (P00C8)	• Institutional MAiD team<br>○ Oversee coordination of MAiD services (P00C8)<br>• Providers must report all death cases to MAiD review team for review (P00C8)
Ontario - *Grand River Hospital*	• No oversight team	• Not applicable	• Institutional MAiD team<br>○ Oversee coordination of MAiD services (P00C50)<br>• Providers must report all death cases to MAiD review team for review (P00C50)
COMMUNITY OF PRACTICE			
Ontario - *Hamilton Family Health Team*	• MAiD oversight team<br>○ Provide resources and guidance for assessors and providers (P00B18)	• MAiD oversight team<br>○ Manager (P00B18)<br>○ Coordinators (P00B18)	• MAiD team<br>○ Ensure assessment forms are completed properly (P00C32)<br>• Providers must report all death cases to MAiD review team for review (P00C32)
Ontario - *Niagara Community MAiD Team, St. Catharine's, Niagara*	• Not applicable<br>○ Organized by individual physicians/nurse practitioners	• Not applicable<br>○ Organized by individual physicians/nurse practitioners	• Providers must report all death cases to MAiD review team for review (P00C11)
GEOGRAPHICAL AREAS			
Ontario - *Oakville & Mississauga area*	• Not applicable<br>○ Not associated with a MAiD program (P00C18)	• Not applicable<br>○ Not associated with a MAiD program (P00C18)	• Providers must report all death cases to MAiD review team for review (P00C18)
Ontario - *Renfrew County*	• Not applicable<br>○ Not associated with a MAiD program (P00C22)	• Not applicable<br>○ Not associated with a MAiD program (P00C22)	• Providers must report all death cases to MAiD review team for review (P00C22)
Ontario - *Oakville, Mississauga & Burlington area*	• Not applicable<br>○ Not associated with a MAiD program (P00C21)	• Not applicable<br>○ Not associated with a MAiD program (P00C21)	• Providers must report all death cases to MAiD review team for review (P00C21)
Ontario - *Greater Toronto Area*	• Not applicable<br>○ Not associated with a MAiD program (P00C24)	• Not applicable<br>○ Not associated with a MAiD program (P00C24)	• Providers must report all death cases to MAiD review team for review (P00C24)
Ontario - *Waterloo area*	• Not applicable<br>○ Not associated with a MAiD program (P00C25)	• Not applicable<br>○ Not associated with a MAiD program (P00C25)	• Providers must report all death cases to MAiD review team for review (P00C25)
Ontario - *Noelville, Sudbury, Elliot Lake, Sturgeon Falls area*	• Not applicable<br>○ Not associated with a MAiD program (P00C26)	• Not applicable<br>○ Not associated with a MAiD program (P00C26)	• Providers must report all death cases to MAiD review team for review (P00C26)
Québec - *Ministry of Health and Social Services*	• MAiD oversight team (Commission on end-of-life care)<br>○ Develop policies and guidelines to support the delivery of MAiD services (P00C7)<br>○ Provides quality control of MAiD processes and provisions (P00C37)<br>○ Ensures compliance of procedures and provisions with Québec law (P00C37)<br>○ Report irreconcilable problems to the College of Medicine in Québec (P00C37)<br>○ Publish annual reports on MAiD related data (P00C7)<br>○ Biweekly review of all MAiD provisions in line with Québec legislation (P00C7)<br>• Council of Physicians, Dentists, and Pharmacists (CMDP)<br>○ Provides quality control of MAiD processes and provisions within each region (P00C7)	• Commission on end-of-life care<br>○ Physicians (17)<br>○ Nurses (17)<br>○ Pharmacists (17)<br>○ Social workers (17)<br>○ Lawyers and notaries (17)<br>○ Patient representative (17)<br>○ Ethicist (17)	• All providers/institutions must report all MAiD requests and deaths to the commission on end-of-life care and the CMDP (P00C37, P00C7)

Jurisdiction	Program oversight: What is the role of the oversight team/committee?	Program oversight: Who are the members of the oversight team/committee?	How is the MAiD program monitored?
INTEGRATED HEALTH AND SOCIAL SERVICES CENTRES			
Québec – *CISS Montérégie*	• MAiD oversight team (Interdisciplinary Support Group)<br>○ Develop policies and guidelines to govern delivery of MAiD (P00C27)<br>○ Ensure alignment in policy directions and delivery of MAiD services (P00C27)<br>○ Support staff in the delivery of MAiD services (P00C27)	• MAiD oversight team (Interdisciplinary Support Group)<br>○ Interdisciplinary Support Group members not specified	• Interdisciplinary Support Group (ISG) monitors legal compliance of MAiD processes and provisions (P00C27)
INTEGRATED UNIVERSITY HEALTH AND SOCIAL SERVICES CENTRES			
Québec – *CIUSS Capitale-Nationale*	• MAiD oversight team (Interdisciplinary Support Group)<br>○ Develop MAiD process and policies for the health region (16)<br>○ Provide resources for assessors and providers (16)<br>○ Support staff in delivery of MAiD services (16)<br>○ Provide education to the general public and health care professionals (16)	• MAiD oversight team (Interdisciplinary Support Group)<br>○ Manager (16)<br>○ Coordinators (Clinical nurses) (16)<br>○ Physicians (16)<br>○ Pharmacist (16)<br>○ Social workers (16)<br>○ Ethicist (16)<br>○ Spiritual care professional (16)	• Interdisciplinary Support Group (ISG) monitors legal compliance of MAiD processes and provisions (P00C37)
Québec – *University of Montreal Hospital Center*	• MAiD oversight team (Institutional MAiD program)<br>○ Develop policies and guidelines to govern delivery of MAiD (P00C40)<br>○ Ensure alignment in policy directions and delivery of MAiD services (P00C40)<br>○ Support staff in the delivery of MAiD services (P00C40)	• MAiD oversight team (Institutional MAiD program)<br>○ Coordinator (Registered nurse) (P00C40)	• Interdisciplinary Support Group (ISG) monitors legal compliance of MAiD processes and provisions (P00C9)
Québec – *McGill University Health Centre*	• MAiD oversight team (Interdisciplinary Support Group)<br>○ Develop policies, processes and guidelines to govern delivery of MAiD (18) (P00C9)<br>○ Ensure alignment in policy directions and delivery of MAiD services (P00C9)<br>○ Support staff in the delivery of MAiD services (18, 19)	• MAiD oversight team (Interdisciplinary Support Group)<br>○ Director, Professional Services (P00C9)<br>○ Coordinator (Advance practice nurse) (P00C9)<br>○ Palliative care physician (P00C9)<br>○ Psychiatrist (P00C9)<br>○ Registered nurses (P00C9)<br>○ Spiritual care professional (P00C9)<br>○ Social worker (P00C9)<br>○ Communication specialist (P00C9)<br>○ Pharmacist (P00C9)<br>○ Patient representative (P00C9)	• Interdisciplinary Support Group (ISG) monitors legal compliance of MAiD processes and provisions (P00C9)

Table 4: Reporting requirements

Jurisdiction	Reporting<br>Who reports preliminary assessment?	Who reports assessments?	Who is required to report provision?	What process is used to ensure reporting accuracy?
Alberta - *Alberta Health Services*	• No preliminary assessment completed (P00B13)	• Delegated reporting<br>○ Federal reporting<br>▪ Provincial care coordination service (P00C39, P00A5)<br>○ Provincial reporting (Provincial care coordination service)<br>▪ Assessors (P00A5, P00C39)	• Delegated reporting<br>○ Federal reporting<br>▪ Provincial coordination service (P00C39, P00A5)<br>○ Provincial reporting (Provincial care coordination service)<br>▪ Providers (P00C39)<br>▪ Pharmacists (P00C39)	• Provincial care coordination service reviews forms for completeness and accuracy (P00B9, P00C10, P00B13)<br>• The Chief Medical Examiner validates MAiD deaths prior to reporting (P00A5)
British Columbia - *Ministry of Health*	• No preliminary assessment completed (P00A4)	• Delegated reporting<br>○ Federal reporting<br>▪ MAiD oversight unit (Ministry of Health) (P00A4)<br>○ Provincial reporting (MAiD oversight unit)<br>▪ Assessors (P00A4)	• Delegated reporting<br>○ Federal reporting<br>▪ MAiD oversight unit (Ministry of Health) (P00A4)<br>○ Provincial reporting (MAiD oversight unit)<br>▪ Providers (P00A4)<br>▪ Pharmacists (P00A4)	• MAiD oversight unit reviews forms for accuracy and completeness and compliance with all applicable legislation, regulations, and standards (P00A4)
British Columbia - *Fraser Health*	• No preliminary assessment completed (P00A4)	• Delegated reporting<br>○ Federal reporting<br>▪ MAiD oversight unit (Ministry of Health) (P00A4)<br>○ Provincial reporting (MAiD oversight unit)<br>▪ Assessors (P00A4)	• Delegated reporting<br>○ Federal reporting<br>▪ MAiD oversight unit (Ministry of Health) (P00A10)<br>○ Provincial reporting (MAiD oversight unit)<br>▪ Regional care coordination service<br>- Submit forms to oversight unit on behalf of some providers (P00A10)<br>▪ Providers (P00A10)<br>▪ Pharmacists (P00A4)	• MAiD Oversight unit reviews forms for accuracy and completeness and compliance with all applicable legislation, regulations, and standards (P00A4)
British Columbia - *Interior Health*	• No preliminary assessment completed<br>○ Ineligibility determined during assessment (P00B14)	• Delegated reporting<br>○ Federal reporting<br>▪ MAiD oversight unit (Ministry of Health) (P00A4)<br>○ Provincial reporting (MAiD oversight unit)<br>▪ Assessors (P00A4)	• Delegated reporting<br>○ Federal reporting<br>▪ MAiD oversight unit (Ministry of Health) (P00B14, P00C29)<br>○ Provincial reporting (MAiD oversight unit)<br>▪ Providers (P00A10, P00B14)<br>▪ Pharmacists (P00A10, P00B14)	• MAiD oversight unit reviews forms for accuracy and completeness and compliance with all applicable legislation, regulations, and standards (P00A4)
British Columbia - *Island Health*	• No preliminary assessment completed (P00A4)	• Delegated reporting<br>○ Federal reporting<br>▪ MAiD oversight unit (Ministry of Health) (P00A4)<br>○ Provincial reporting (MAiD oversight unit)<br>▪ Assessors (P00A4)	• Delegated reporting<br>○ Federal reporting<br>▪ MAiD oversight unit (Ministry of Health) (P00A9, P00A13)<br>○ Provincial reporting (MAiD oversight unit)<br>▪ Providers (P00A10)<br>▪ Pharmacists (P00A4)	• MAiD oversight unit reviews forms for accuracy and completeness and compliance with all applicable legislation, regulations, and standards (P00A4)
British Columbia - *Northern Health*	• No preliminary assessment completed (P00A4)	• Delegated reporting<br>○ Federal reporting<br>▪ MAiD oversight unit (Ministry of Health) (P00A8)<br>○ Provincial reporting (MAiD oversight unit)<br>▪ Assessors (P00A8)	• Delegated reporting<br>○ Federal reporting<br>▪ MAiD oversight unit (Ministry of Health) (P00A8)<br>○ Provincial reporting (MAiD oversight unit)<br>▪ Providers (P00A8)<br>▪ Pharmacists (P00A4)	• MAiD oversight unit reviews forms for accuracy and completeness and compliance with all applicable legislation, regulations, and standards (P00A4)
British Columbia - *Vancouver Coastal Health*	• No preliminary assessment completed (P00B16)<br>○ Ineligible requests are informally document on internal system (P00B16)	• Delegated reporting<br>○ Federal reporting<br>▪ MAiD oversight unit (Ministry of Health) (P0B10, P00B16)<br>○ Provincial reporting (MAiD oversight unit)<br>▪ Assessors (P00B16)	• Delegated reporting<br>○ Federal reporting<br>▪ MAiD oversight unit (Ministry of Health) (P00B10, P00B16)<br>○ Provincial reporting (MAiD oversight unit)<br>▪ Providers (P00B10, P00B16)<br>▪ Pharmacists (P00A4)	• MAiD oversight unit reviews forms for accuracy and completeness and compliance with all applicable legislation, regulations, and standards (P00A4)

Jurisdiction	Reporting			
	Who reports preliminary assessment?	Who reports assessments?	Who is required to report provision?	What process is used to ensure reporting accuracy?
British Columbia - *Provincial Health Services Authority*	• No preliminary assessment completed (P00A4)	• Delegated reporting<br>○ Federal reporting<br>▪ MAiD oversight unit (Ministry of Health) (P00A11)<br>○ Provincial reporting (MAiD oversight unit)<br>▪ Assessors (P00A11)	• Delegated reporting<br>○ Federal reporting<br>▪ MAiD oversight unit (Ministry of Health) (P00A11)<br>○ Provincial reporting (MAiD oversight unit)<br>▪ Providers (P00A11)<br>▪ Pharmacists (P00A4)	• MAiD oversight unit reviews forms for accuracy and completeness and compliance with all applicable legislation, regulations, and standards (P00A4)
Manitoba - *Shared Health*	• Physician (P00C33, P00C34)<br>○ Medical Director (P00C33, P00C34)	• Direct reporting<br>○ Federal reporting<br>▪ Assessors (P00C34, P00C33)	• Direct reporting<br>○ Federal reporting<br>▪ Providers (P00C33)<br>▪ Pharmacists (P00B19, P00C34)	• Assessors and providers review their own forms (P00C33)
New Brunswick - *Horizon Health Network*	• Coordinator (Regional lead) (P00B11)<br>○ Registered nurse (P00B11)	• Direct reporting<br>○ Federal reporting<br>▪ Assessors (P00B11)	• Direct reporting<br>○ Federal reporting<br>▪ Providers (P00B11)<br>▪ Pharmacists (P00B11) (20)	• Assessors and providers review their own forms (P00C15)
New Brunswick - *Vitalité Health Network*	• No preliminary assessment completed (P00C48)	• Direct reporting<br>○ Federal reporting<br>▪ Assessors (P00C48)	• Direct reporting<br>○ Federal reporting<br>▪ Providers (P00C48)<br>▪ Pharmacists (20)	• Risk management reviews forms for completeness and accuracy prior to provision (P00C48)
Newfoundland and Labrador - *Department of Health and Community Services*	• Not applicable<br>○ Assessors and providers complete direct reporting	• Not applicable<br>○ Assessors and providers complete direct reporting	• Not applicable<br>○ Assessors, providers and pharmacists complete direct reporting	• Not applicable<br>○ Assessors and providers complete direct reporting
Newfoundland and Labrador - *Eastern Zone*	• Physician (P00C43)<br>○ Medical Director (P00C43)	• Direct reporting<br>○ Federal reporting<br>▪ Assessors (Primary assessors) (P00C43)	• Direct reporting<br>○ Federal reporting<br>▪ Providers (P00C43)<br>▪ Pharmacists (21)	• Assessors and providers review their own forms (P00C43)
Newfoundland and Labrador - *Western Zone*	• Physicians and nurse practitioners (P00C4)<br>○ Primary Assessors (P00C44)	• Direct reporting<br>○ Federal reporting<br>▪ Assessors (Primary assessors) (P00C44)	• Direct reporting<br>○ Federal reporting<br>▪ Providers (P00C44)<br>▪ Pharmacists (21)	• Assessors and providers review their own forms (P00C44)
Newfoundland and Labrador - *Central Zone*	• No preliminary assessment completed (P00B22)	• Direct reporting<br>○ Federal reporting<br>▪ Assessors (P00B22)	• Direct reporting<br>○ Federal reporting<br>▪ Providers (P00B22)<br>▪ Pharmacists (21)	• Coordinator requests assessors and providers to send forms for review (P00B22)
Northwest Territories	• No preliminary assessment completed (P00C35)	• Delegated reporting<br>○ Federal reporting<br>▪ The Review Committee (P00A3, P00C35)<br>○ Territorial reporting (Review committee)<br>▪ Assessors (P00C35)	• Delegated reporting<br>○ Federal reporting<br>▪ The Review Committee (P00A3, P00C35)<br>○ Territorial reporting (Review committee)<br>▪ Providers (11)<br>▪ Pharmacists (11)	• MAiD review committee reviews forms for completeness and accuracy (P00C35)<br>• Assessors and providers review their own forms (P00A3)<br>○ Ensure patient charts contains updated form (P00A3)
Nova Scotia - *Nova Scotia Health*	• No preliminary assessment completed (P00A12)	• Direct reporting<br>○ Federal reporting<br>▪ Assessors (P00A12)	• Direct reporting<br>○ Federal reporting<br>▪ Providers (P00C38)<br>▪ Pharmacists (P00A12)	• Assessors and providers review their own forms (P00C38)
Prince Edward Island - *Health PEI*	• No preliminary assessment completed (P00B17)	• Direct reporting<br>○ Federal reporting<br>▪ Assessors (P00B17)	• Direct reporting<br>○ Federal reporting<br>▪ Providers (22) (P00B32)<br>▪ Pharmacists (23) (P00B17)	• Assessors and providers review their own forms (P00B17)<br>• Availability of procedural reporting document to ensure compliance with Health Canada reporting (P00B8)
Saskatchewan - *Saskatchewan Health Authority*	• Coordinator (Registered nurse) (P00A1)<br>• Health Information Management Practitioner (P00A1)	• Delegated reporting<br>○ Federal reporting<br>▪ MAiD coordination program (P00B1, P00A1, P00C19, P00B5)<br>○ Provincial reporting (MAiD coordination program)<br>▪ Assessors (P00A1)	• Delegated reporting<br>○ Federal reporting<br>▪ MAiD coordination program (P00B1, P00A1, P00C19, P00B5)<br>○ Provincial reporting (MAiD coordination program)<br>▪ Providers (P00B1, P00A1, P00C19, P00B5)<br>▪ Pharmacists (P00B1, P00A1, P00C19, P00B5)	• MAiD coordination program reviews forms for completeness and accuracy (P00C1)

Jurisdiction	Reporting			
	**Who reports preliminary assessment?**	**Who reports assessments?**	**Who is required to report provision?**	**What process is used to ensure reporting accuracy?**
Yukon - *Department of Health and Social Services*	• No preliminary assessment completed (P00C14)	• Direct reporting<br>○ Federal reporting<br>▪ Assessors (First Assessor) (P00C14)	• Direct reporting<br>○ Federal reporting<br>▪ Providers (P00C14)<br>▪ Pharmacists (15)	• Assessors and providers review their own forms (P00C14)
Ontario - *Ministry of Health*	• Non-medical practitioners complete preliminary assessments and report ineligibility (24)	• Direct reporting<br>○ Federal reporting<br>▪ Assessors (P00B4)	• Direct reporting<br>○ Federal reporting<br>▪ Pharmacists (24, 25)	• Assessors review their own forms (P00B20)<br>• Ministry of Heath receives monthly and quarterly statistics on reported MAiD deaths from Office of the Chief Coroner (P00B20)
Ontario - *Office of the Chief Coroner*	• Not applicable	• Not applicable	• Delegated reporting<br>○ Federal reporting<br>▪ Office of the Chief Coroner (P00B20)<br>○ Provincial reporting (Office of the Chief Coroner)<br>▪ Providers (P00B20)	• Office of the Chief Coroner reviews provision forms for accuracy and completeness and compliance with all applicable legislation, regulations, and standards with quarterly submissions to Health Canada (P00B20)<br>• Follow up for clarifications and education with providers (P00B20)
SERVICE ORGANIZATIONS/REGIONAL FACILITIES				
Ontario - *Home and Community Care Support Services, Central East*	• No preliminary assessment completed (P00C2)	• Direct reporting<br>○ Federal reporting<br>▪ Assessors (P00C2)	• Direct reporting<br>○ Federal reporting<br>▪ Pharmacists (24)<br>• Delegated reporting<br>○ Federal reporting<br>▪ Office of the Chief Coroner (P00C2)<br>○ Provincial reporting (Office of the Chief Coroner)<br>▪ Providers (P00C2)	• Office of the Chief Coroner reviews provision forms completeness and accuracy (P00B20)<br>• Assessors and providers review their own forms (P00C2)
Ontario - *Home and Community Care Support Services, Waterloo Region*	• No preliminary assessment completed (P00C4)	• Direct reporting<br>○ Federal reporting<br>▪ Assessors (P00B4)	• Direct reporting<br>○ Federal reporting<br>▪ Pharmacists (24)<br>• Delegated reporting<br>○ Federal reporting<br>▪ Office of the Chief Coroner (P00C4)<br>○ Provincial reporting (Office of the Chief Coroner)<br>▪ Providers (P00C4)	• Office of the Chief Coroner reviews provision forms for accuracy and completeness (P00B20)<br>• Assessors and providers review their own forms (P00C4)
Ontario - *Home and Community Care Support Services, South East*	• No preliminary assessment completed (P00C20)	• Direct reporting<br>○ Federal reporting<br>▪ Assessors (P00C20)	• Direct reporting<br>○ Federal reporting<br>▪ Pharmacists (23)<br>• Delegated reporting<br>○ Federal reporting<br>▪ Office of the Chief Coroner (P00C36)<br>○ Provincial reporting (Office of the Chief Coroner)<br>▪ Providers (P00C36)	• Office of the Chief Coroner reviews provision forms for completeness and accuracy (P00B20)<br>• Assessors and providers review their own forms (P00C20)
Ontario - *Home and Community Care Support Services, South West*	• No preliminary assessment completed (P00C23)	• Direct Reporting<br>○ Federal reporting<br>▪ Assessors (P00C32)	• Direct reporting<br>○ Federal reporting<br>▪ Pharmacists (24)<br>• Delegated reporting<br>○ Federal reporting<br>▪ Office of the Chief Coroner (P00C32)<br>○ Provincial reporting (Office of the Chief Coroner)<br>▪ Providers (P00C32)	• Office of the Chief Coroner reviews provision forms for completeness and accuracy (P00B20)<br>• Assessors and providers review their own forms (P00C23)
Ontario - *Champlain Regional MAiD Network, the Ottawa Hospital*	• Advanced practice nurse and program manager (P00C5)	• Direct reporting<br>○ Federal reporting<br>▪ Assessors (P00C5)	• Direct reporting<br>○ Federal reporting<br>▪ Pharmacists (24)<br>• Delegated reporting<br>○ Federal reporting<br>▪ Office of the Chief Coroner (P00C5)<br>○ Provincial reporting (Office of the Chief Coroner)<br>▪ Providers (P00C5)	• Office of the Chief Coroner reviews provision forms for completeness and accuracy (P00B20)<br>• Assessors and providers review their own forms (P00C5)
HEALTHCARE FACILITIES				

Jurisdiction	Reporting: Who reports preliminary assessment?	Who reports assessments?	Who is required to report provision?	What process is used to ensure reporting accuracy?
Ontario - *Peterborough Regional Health Centre*	• No preliminary assessment completed (P00C3)	• Direct reporting<br>○ Federal reporting<br>▪ Assessors (P00C3)	• Direct reporting<br>○ Federal reporting<br>▪ Pharmacists (24)<br>• Delegated reporting<br>○ Federal reporting<br>▪ Office of the Chief Coroner (P00C3)<br>○ Provincial reporting (Office of the Chief Coroner)<br>▪ Providers (P00C3)	• Office of the Chief Coroner reviews provision forms for completeness and accuracy (P00B20)<br>• Assessors and providers review their own forms (P00C3)
Ontario - Mount Sinai Healthcare Facility, Toronto	• No preliminary assessment completed<br>○ Did not want to create barriers to access (P00C36)<br>○ Assessors conduct an assessment, the preliminary assessment seemed duplicative (P00C36)	• Direct reporting<br>○ Federal reporting<br>▪ Assessors (P00C36)	• Direct reporting<br>○ Federal reporting<br>▪ Pharmacists (24)<br>• Delegated reporting<br>○ Federal reporting<br>▪ Office of the Chief Coroner (P00C36)<br>○ Provincial reporting (Office of the Chief Coroner)<br>▪ Providers (P00C36)	• Office of the Chief Coroner reviews provision forms for completeness and accuracy (P00B20)<br>• Assessors and providers review their own forms (P00C36)
Ontario - *University Health Network*	• No preliminary assessment completed (P00C8)	• Direct reporting<br>○ Federal reporting<br>▪ Assessors (P00C8)	• Direct reporting<br>○ Federal reporting<br>▪ Pharmacists (24)<br>• Delegated reporting<br>○ Federal reporting<br>▪ Office of the Chief Coroner (P00C8)<br>○ Provincial reporting (Office of the Chief Coroner)<br>▪ Providers (P00C8)	• Office of the Chief Coroner reviews provision forms for completeness and accuracy (P00B20)<br>• Assessors and providers review their own forms (P00C8)
Ontario - *Grand River Hospital*	• Nurse practitioner (Coordinator) (P00C50)<br>○ If patient dies prior to MAiD provision (P00C50)<br>○ If patient declines to proceed with MAiD (P00C50)	• Direct reporting<br>○ Federal reporting<br>▪ Assessors (P00C50)	• Direct reporting<br>○ Federal reporting<br>▪ Pharmacists (24)<br>• Delegated reporting<br>○ Federal reporting<br>▪ Office of the Chief Coroner (P00C50)<br>○ Provincial reporting (Office of the Chief Coroner)<br>▪ Providers (P00C50)	• Office of the Chief Coroner reviews provision forms for completeness and accuracy (P00B20)<br>• Assessors and providers review their own forms (P00C50)
COMMUNITY OF PRACTICE				
Ontario - *Hamilton Family Health Team*	• No preliminary assessment completed (P00C32)	• Direct reporting<br>○ Federal reporting<br>▪ Assessors (Primary assessors) (P00C32)	• Direct reporting<br>○ Federal reporting<br>▪ Pharmacists (24)<br>• Delegated reporting<br>○ Federal reporting<br>▪ Office of the Chief Coroner (P00C32)<br>○ Provincial reporting (Office of the Chief Coroner)<br>▪ Providers (P00C32)	• Office of the Chief Coroner reviews provision forms for completeness and accuracy (P00B20)<br>• Assessors and providers review their own forms (P00C32)
Ontario - *Niagara Community MAiD Team, St. Catharine's, Niagara*	• No preliminary assessment completed (P00C11)	• Direct reporting<br>○ Federal reporting<br>▪ Assessors (P00C11)	• Direct reporting<br>○ Federal reporting<br>▪ Pharmacists (24)<br>• Delegated reporting<br>○ Federal reporting<br>▪ Office of the Chief Coroner (P00C11)<br>○ Provincial reporting (Office of the Chief Coroner)<br>▪ Providers (P00C11)	• Office of the Chief Coroner reviews provision forms for completeness and accuracy (P00B20)<br>• Assessors and providers review their own forms (P00C11)
GEOGRAPHICAL AREAS				

Jurisdiction	Reporting			
	Who reports preliminary assessment?	Who reports assessments?	Who is required to report provision?	What process is used to ensure reporting accuracy?
Ontario - *Oakville & Mississauga area*	• No preliminary assessment completed (P00C18)	• Direct reporting<br>○ Federal reporting<br>▪ Assessors (P00C18)	• Direct reporting<br>○ Federal reporting<br>▪ Pharmacists (24)<br>• Delegated reporting<br>○ Federal reporting<br>▪ Office of the Chief Coroner (P00C18)<br>○ Provincial reporting (Office of the Chief Coroner)<br>▪ Providers (P00C18)	• Office of the Chief Coroner reviews provision forms for completeness and accuracy (P00B20)<br>• Assessors and providers review their own forms (P00C18)
Ontario - *Oakville, Mississauga & Burlington area*	• No preliminary assessment completed (P00C21)	• Direct reporting<br>○ Federal reporting<br>▪ Assessors (P00C21)	• Direct reporting<br>○ Federal reporting<br>▪ Pharmacists (24)<br>• Delegated reporting<br>○ Federal reporting<br>▪ Office of the Chief Coroner (P00C21)<br>○ Provincial reporting (Office of the Chief Coroner) Providers (P00C21)	• Office of the Chief Coroner reviews provision forms for completeness and accuracy (P00B20)<br>• Assessors and providers review their own forms (P00C21)
Ontario - *Renfrew County*	• No preliminary assessment completed (P00C22)	• Direct reporting<br>○ Federal reporting<br>▪ Assessors (P00C22)	• Direct reporting<br>○ Federal reporting<br>▪ Pharmacists (24)<br>• Delegated reporting<br>○ Federal reporting<br>▪ Office of the Chief Coroner (P00C22)<br>○ Provincial reporting (Office of the Chief Coroner)<br>▪ Providers (P00C22)	• Office of the Chief Coroner reviews provision forms for completeness and accuracy (P00B20)<br>• Assessors and providers review their own forms (P00C22)
Ontario - *Greater Toronto Area*	• No preliminary assessment completed (P00C24)	• Direct reporting<br>○ Federal reporting<br>▪ Assessors (P00C24)	• Direct reporting<br>○ Federal reporting<br>▪ Pharmacists (24)<br>• Delegated reporting<br>○ Federal reporting<br>▪ Office of the Chief Coroner (P00C24)<br>○ Provincial reporting (Office of the Chief Coroner)<br>▪ Providers (P00C24)	• Office of the Chief Coroner reviews provision forms for completeness and accuracy (P00B20)<br>• Assessors and providers review their own forms (P00C24)
Ontario - *Waterloo area*	• No preliminary assessment completed (P00C25)	• Direct reporting<br>○ Federal reporting<br>▪ Assessor (P00C25)	• Direct reporting<br>○ Federal reporting<br>▪ Pharmacists (24)<br>• Delegated reporting<br>○ Federal reporting<br>▪ Office of the Chief Coroner (P00C25)<br>○ Provincial reporting (Office of the Chief Coroner)<br>▪ Providers (P00C25)	• Office of the Chief Coroner reviews provision forms for completeness and accuracy (P00B20)<br>• Assessors and providers review their own forms (P00C25)
Ontario - *Noelville, Sudbury, Elliot Lake, Sturgeon Falls area*	• No preliminary assessment completed (P00C26)	• Direct reporting<br>○ Federal reporting<br>▪ Primary assessors (P00C26)	• Direct reporting<br>○ Federal reporting<br>▪ Pharmacists (24)<br>• Delegated reporting<br>○ Federal reporting<br>▪ Office of the Chief Coroner (P00C26)<br>○ Provincial reporting (Office of the Chief Coroner)<br>▪ Providers (P00C26)	• Office of the Chief Coroner reviews provision forms for completeness and accuracy (P00B20)<br>• Assessors and providers review their own forms (P00C26)
Québec - *Ministry of Health and Social Services*	• Not applicable	• Delegated reporting<br>○ Federal reporting<br>▪ Ministry of Health and Social Services (P00C27)<br>○ Provincial reporting (Ministry of Health and Social Services)<br>▪ Assessors (P00C27)	• Delegated reporting<br>○ Federal reporting<br>▪ Ministry of Health and Social Services (P00C27)<br>○ Provincial reporting (Ministry of Health and Social Services)<br>▪ Providers (P00C27)	• Commission on end-of-life reviews all forms for completeness and compliance with legislation (P00C37)<br>○ Refer all non-complaint cases to the College of Medicine of Québec (P00C37)<br>• Ministry of Health and Social Sciences receives periodic reports from the Commission on end-of-life (P00C37)

Jurisdiction	Reporting			
	Who reports preliminary assessment?	Who reports assessments?	Who is required to report provision?	What process is used to ensure reporting accuracy?
INTEGRATED HEALTH AND SOCIAL SERVICES CENTRES				
Québec – *CISS Montérégie*	• No preliminary assessment completed (P00C27)	• Delegated reporting<br>○ Federal reporting<br>▪ Ministry of Health and Social Services (P00C27)<br>○ Provincial reporting (Ministry of Health and Social Services)<br>▪ Assessors (P00C27)	• Delegated reporting<br>○ Federal reporting<br>▪ Ministry of Health and Social Services (P00C27)<br>○ Provincial reporting (Ministry of Health and Social Services)<br>▪ Providers (P00C27)	• Commission on end-of-life reviews all forms for completeness and compliance with legislation (P00C37)<br>○ Refer all non-compliant cases to the College of Medicine of Québec (P00C37)
INTEGRATED UNIVERSITY HEALTH AND SOCIAL SERVICES CENTRES				
Québec – *CIUSS Capitale-Nationale*	• No preliminary assessment completed (P00C37)	• Delegated reporting<br>○ Federal reporting<br>▪ Ministry of Health and Social Services (P00C37)<br>○ Provincial reporting (Ministry of Health and Social Services)<br>▪ Assessors (P00C37)	• Delegated reporting<br>○ Federal reporting<br>▪ Ministry of Health and Social Services (P00C37)<br>○ Provincial reporting (Ministry of Health and Social Services)<br>▪ Providers (P00C37)	• Commission on end-of-life reviews all forms for completeness and compliance with legislation (P00C37)<br>○ Refer all non-compliant cases to the College of Medicine of Québec (P00C37)
Québec – *University of Montreal Hospital Center*	• No preliminary assessment completed (P00C40)	• Delegated reporting<br>○ Federal reporting<br>▪ Ministry of Health and Social Services (P00C40)<br>○ Provincial reporting (Ministry of Health and Social Services)<br>▪ Assessors (P00C40)	• Delegated reporting<br>○ Federal reporting<br>▪ Ministry of Health and Social Services (P00C40)<br>○ Provincial reporting (Ministry of Health and Social Services)<br>▪ Providers (P00C40)	• Commission on end-of-life reviews all forms for completeness and compliance with legislation (P00C37)<br>○ Refer all non-compliant cases to the College of Medicine of Québec (P00C37)
Québec – *McGill University Health Centre*	• No preliminary assessment completed (P00C9)	• Delegated reporting<br>○ Federal reporting<br>▪ Ministry of Health and Social Services (P00C9)<br>○ Provincial reporting (Ministry of Health and Social Services)<br>▪ Assessors (P00C9)	• Delegated reporting<br>○ Federal reporting<br>▪ Ministry of Health and Social Services (P00C9)<br>○ Provincial reporting (Ministry of Health and Social Services)<br>▪ Providers (P00C9)	• Commission on end-of-life reviews all forms for completeness and compliance with legislation (P00C9)<br>○ Refer all non-compliant cases to the College of Medicine of Québec (P00C9)

Table 5: Referral process and coordination

Jurisdiction	Where can information initially be accessed?	How can services be contacted?	Who can make the request to start the referral process?	What are the structural elements of care coordination services?	
				**Team Structure and Process**	**Roles and Responsibilities**
Alberta - *Alberta Health Services*	• Provincial care coordination service (P00B9, P00B13, P00C39)<br>• Healthcare providers (4)<br>• Website (4)<br>• Printed material<br>○ Booklet (3)<br>• Health link (P00A5)<br>○ 811	• Email (26)<br>• Telephone (26)<br>• Fax (P00C39)	• Healthcare providers (P00C6)<br>• Self-referral (P00B9, P00B13, P00C39)	Centralized Provincial<br>• Regional (zone) coordination teams (P00B9, P00A5)<br>○ Central and Calgary zone navigator team (P00C39)<br>▪ Coordinators (Navigators)<br>- 2 Registered nurses (P00C39)<br>- 1 Emergency medical specialist (up to 0.7 FTE) (P00C39)<br>○ Edmonton and north zone navigator team<br>▪ Coordinators (Navigators) (P00A5, P00B9)<br>- 2 Registered nurses (1.0FTE) (P00A5, P00B9)<br>- 1 Support nurse (up to 0.6 FTE) (P00A5, P00B9)<br>▪ Office administrator (Casual) (P00B9)<br>○ South zone navigator team<br>▪ Coordinator (Navigator)<br>- 1 Registered nurse (up to 0.8 FTE) (P00B9)<br>• Not all MAiD referrals are routed through this service (P00A5)<br>○ Assessments and provisions can be completed without the involvement of the care coordination service (P00A5)<br>○ Care coordination service must be notified of a MAiD provision for reporting purpose (P00A5)	• Provide MAiD information (P00B13)<br>• Accept and process referrals (P00B13)<br>• Coordinate assessments and provisions (P00C39)<br>○ Contact patient's primary care provider (P00B9, P00B13)<br>• Support completion of forms in compliance with legislation (P00B9, P00C10, P00B13)<br>• Provide assistance, support, and/or guidance<br>○ Facilitate referrals to support services (P00B13)<br>○ Coordinate with pharmacy services (P00B9)<br>○ Provide logistics support (P00B13)<br>○ Convey patient preferences to staff (P00B13)
British Columbia - *Ministry of Health*	• Website (27)	• Not applicable<br>○ Decentralized into regions	• Not applicable<br>○ Decentralized into regions	• Not applicable<br>○ Decentralized into regions	• Not applicable<br>○ Decentralized into regions
British Columbia - *Fraser Health*	• Regional care coordination service (6)<br>• Healthcare providers (6)<br>• Website (6)<br>• Printed material<br>○ Pamphlet (28)<br>○ Fact sheet (29)<br>○ MAiD process guide (30)<br>• Community education session (P00A10)	• Email (6)<br>• Telephone (6)<br>• Fax (28)	• Healthcare providers (P00A10)<br>• Self-referral (P00A10)	Centralized Regional<br>• Coordination team<br>○ Clinical coordinators<br>▪ 2 Registered nurses (P00A10)<br>▪ 2 Social workers (P00A10)<br>○ 1 Mental health clinician/clinical coordinator (P00A10)<br>▪ Social worker<br>○ 1 Office administrator coordinator (P00A10)<br>• All MAiD referrals are routed through this service (P00A10)	• Provide MAiD information (6)<br>• Accept and process referrals (28)<br>• Coordinate assessments and provisions (6, 28)<br>○ Contact patients' primary care provider for assessment (P00A10)<br>• Support completion of forms in compliance with legislation (31)<br>• Coordinate patient transfers (6)<br>• Assist in identifying independent witnesses (6, 28)<br>• Provide assistance, support, and/or guidance (P00A10)<br>○ Provide education and resources to staff (31)<br>○ Convey patient preferences to staff (P00A10)
British Columbia - *Interior Health*	• Regional care coordination service (32)<br>• Healthcare providers (33)<br>• Website (33)<br>• Printed material<br>○ Pamphlets (32-34)<br>○ Support guide (32-34)	• Email (32)<br>• Telephone (32)<br>• Toll-free number (32)	• Healthcare providers (P00C29)<br>• Self-referral (P00C29)	Centralized Regional<br>• Coordination team<br>○ 7 Coordinators<br>▪ Registered nurses (P00B14)<br>▪ Social workers (P00B14)<br>• Not all MAiD referrals are routed through this service (P00B14)<br>○ Assessments and provisions can be conducted without the involvement of the care coordination service (P00B14)	• Provide MAiD information (32-34)<br>• Accept and process referrals (32-34)<br>• Coordinate assessments and provisions (32-34)<br>○ Contact patients' primary care provider for assessment (32-34)<br>• Support completion of forms in compliance with legislation (32-34)<br>• Assist in identifying independent witness (32-34)<br>• Provide assistance, support, and/or guidance (32-34)<br>○ Document patients and families' wishes and preferences (P00B14)
British Columbia - *Island Health*	• Regional care coordination service (35)<br>• Healthcare providers (35, 36)<br>• Website (35)<br>• Printed material<br>○ Brochures (36) (P00A9)<br>○ Pamphlets (36) (P00A9)<br>○ Instruction booklets (36) (P00A9)	• Email (35)<br>• Telephone (35)<br>• Toll-free number (35)<br>• Other<br>○ Local care teams (P00A9)	• Healthcare providers (P00A13)<br>• Self-referral (P00A13)	Hybrid Regional<br>• Coordination team<br>○ Coordinators<br>▪ 3 Registered nurses (1.0 FTE) (P00A9)<br>• Not all MAiD referrals are routed through this service (P00A13)<br>○ Community of practice in small and remote communities complete assessments and provisions without involvement of the care coordination service (P00A13)	• Provide MAiD information (P00A9)<br>• Accept and process referrals (P00A9)<br>• Coordinate assessments and provisions (P00A9)<br>○ Contact patients' primary care provider for assessment (P00A9)<br>• Support completion of forms in compliance with legislation (P00A9)<br>• Coordinate patient transfers (P00A13)<br>• Assist in identifying independent witness (P00A9)<br>• Provide assistance, support, and guidance (P00A9, P00A13)

Jurisdiction	Where can information initially be accessed?	How can services be contacted?	Who can make the request to start the referral process?	What are the structural elements of care coordination services?	
				Team Structure and Process	Roles and Responsibilities
					○ Provide information on care support services including bereavement and subsidized or private services (P00A9)
British Columbia - *Northern Health*	• Regional care coordination service (37)<br>• Healthcare providers (37)<br>• Website (37)	• Email (37)<br>• Telephone (37)	• Healthcare providers (P00A8)<br>• Self-referral (P00A8)	Centralized Regional<br>• Coordination team<br>○ Coordinator<br>▪ 1 Social worker (0.5 FTE) (P00A8)<br>• Not all MAiD referrals are routed through this service (P00A8)<br>○ Assessments and provisions can be conducted without the involvement of the care coordination service (P00A8)	• Provide MAiD information (37)<br>• Accept and process referrals (37)<br>• Coordinate assessments and provisions (37)
British Columbia - *Vancouver Coastal Health*	• Regional care coordination service (P00B10)<br>• Healthcare providers (38)<br>• Website (38)<br>• Printed materials<br>○ Pamphlet (39)<br>○ MAiD support guide (40)	• Email (38)<br>• Telephone (38)<br>• Toll-free number (38)	• Healthcare provider (P00A7)<br>• Self-Referral (P00A7)	Centralized Regional<br>• Coordination team<br>○ Coordinators<br>▪ 1 Registered nurse (1.0 FTE) (P00B12)<br>▪ 4 Social workers (1.0 FTE) (P00B12)<br>• Not all MAiD referrals are routed through this service (P00A7)<br>○ Assessments and provisions can be conducted without the involvement of the care coordination service (P00A7)	• Provide MAiD information (P00B12)<br>• Accept and process referrals (P00B12)<br>• Coordinate assessments and provisions (P00B10)<br>• Coordinate patient transfers (P00B15)<br>• Resolve issues raised by assessors and providers (P00B12)<br>• Provide assistance, support, and/or guidance (P00A7)<br>○ Provide logistics support (P00A7)<br>○ Document patients and families' wishes and preferences (P00A7)<br>○ Offer informal emotional support for clients and families (P00B15, P00B12)
British Columbia - Provincial *Health Services Authority*	• Website (41)	• Email (41)<br>• Telephone (41)	• Healthcare providers (P00A11)<br>• Self-referral (P00A11)	Centralized Provincial<br>• Coordination team<br>○ Coordinator (Risk director) (P00A11)<br>▪ Registered nurse (P00A11)<br>○ Project manager (P00A11)<br>▪ Registered nurse (P00A11)<br>• All MAiD referrals for inpatient cancer patients are routed through this service (P00A11)	• Accept and process referrals (P00A11)<br>• Coordinates requests from BC cancer centers (P00A11)
Manitoba - *Shared Health*	• Provincial care coordination service (P00B21)<br>• Healthcare providers (42)<br>• Website (43)<br>• Printed materials<br>○ Pamphlet (43)	• Email (P00B19, P00B21, P00C33)<br>• Telephone (7)<br>• Toll-free number (7)<br>• Fax (7)	• Healthcare providers (P00C34, P00B19)<br>• Self-Referral (P00C33, P00B21, P00B19)	Centralized Provincial<br>• Coordination team<br>○ Coordinators<br>▪ 5 Registered nurses (3.0 FTE) (P00B21)<br>• All MAiD referrals are routed through this service (P00B21, P00B19)	• Provide MAiD information (P00B19)<br>• Accept and process referrals (P00B19)<br>• Coordinate assessments and provisions (P00B19)<br>• Coordinate patient transfers (P00C33, P00C34)<br>• Eligibility review (P00B21)<br>• Provide assistance, support, and/or guidance (44)<br>○ Document patients and families' wishes and preferences (P00C34)<br>○ Convey patient preferences to staff (P00C34)
New Brunswick - *Horizon Health Network*	• Regional care coordination service (P00B11)<br>• Healthcare providers (45)<br>• Website (45)<br>• Printed materials<br>○ Pamphlet (9)	• Email (45)<br>• Toll-free number (45)<br>• Fax (P00B11)<br>• Other<br>○ Contact through healthcare team (P00C15)	• Healthcare providers (45)<br>• Self-Referral (P00B11)	Centralized Regional<br>• Coordination team<br>○ Coordinator (Regional MAiD lead) (P00B11)<br>▪ 1 Registered nurse<br>○ Administrative support (P00B11)<br>• Not all MAiD referrals are routed through this service (P00B11)<br>○ Assessments and provisions can be coordinated without the involvement of the care coordination service (P00B11)	• Provide MAiD information (P00B11)<br>• Accept and process referrals (P00B11)<br>• Coordinate assessments and provisions (P00B11)<br>○ Support patients to identify practitioners for assessment (P00B11)<br>• Coordinate patient transfers (P00B11)<br>• Provide assistance, support, and/or guidance<br>○ Coordinate with pharmacy services (P00B11)<br>○ Provide logistics support (P00B11)
New Brunswick - *Vitalité Health Network*	• Healthcare providers (P00C48)<br>• Website (46)<br>• Printed materials<br>○ Pamphlet (47)	• Email (46)<br>• Telephone (46) (P00C48)<br>• Other<br>○ Patient experience department (46)	• Healthcare providers (P00C48)<br>• Self-Referral (P00C48)	• No coordination team (P00C48)	• Not applicable

Jurisdiction	Where can information initially be accessed?	How can services be contacted?	Who can make the request to start the referral process?	What are the structural elements of care coordination services?	
				Team Structure and Process	Roles and Responsibilities
Newfoundland and Labrador - *Department of Health and Community Services*	• Not applicable<br>○ Decentralized into regions	• Not applicable<br>○ Decentralized into regions	• Not applicable<br>○ Decentralized into regions	• Not applicable<br>○ Decentralized into regions	• Not applicable<br>○ Decentralized into regions
Newfoundland and Labrador - *Eastern Zone*	• Regional care coordination service (48)<br>• Healthcare providers (48)<br>• Website (48)<br>• Printed materials<br>○ Instruction booklets (10)	• Email (10)<br>• Telephone (10)<br>• Toll-free number (10)<br>• Fax (10)	• Healthcare providers (10)<br>• Self-referral (10)	Centralized Regional<br>• Coordination team<br>○ Coordinators (P00C43)<br>▪ 2 Registered nurses (P00C43)<br>▪ Shared with the palliative care program<br>• Not all MAiD referrals are routed through this service (P00C43)<br>○ Assessments can be coordinated without the involvement of the care coordination service (P00C43)	• Provide MAiD information (48)<br>• Accept and process referrals (48)<br>• Coordinate assessments and provisions (48)<br>○ Support patients to identify practitioners for assessment (48)<br>• Coordinate patient transfers (P00C43)<br>• Provide assistance, support, and/or guidance<br>○ Coordinate pharmacy and nursing services (48)<br>○ Facilitate referrals to grief services (48)<br>○ Provide logistics support (P00C43)
Newfoundland and Labrador - *Western Zone*	• Regional care coordination service (10)<br>• Website (10)<br>• Printed materials<br>○ Instruction booklets (10)	• Email (10)<br>• Telephone (10)<br>• Fax (10)	• Healthcare providers (P00C44)<br>• Self-referral (P00C44)	Centralized Regional<br>• Coordination team<br>○ Coordinator<br>▪ 1 Administrative assistant (P00C44)<br>• Not all MAiD referrals are routed through this service (P00C44)<br>○ Assessments can be coordinated without the involvement of the care coordination service (P00C44)	• Provides MAiD information (P00C44)<br>• Accept and process referrals (P00C44)<br>• Coordinate assessments and provisions (P00C44)<br>○ Support patients to identify practitioners for assessment (P00C44)
Newfoundland and Labrador - *Central Zone*	• Regional care coordination service (P00B22)<br>• Website (10)<br>• Printed materials<br>○ Instruction booklets (10)	• Email (10)<br>• Telephone (24)	• Healthcare providers (P00B22)<br>• Self-referral (P00B22)	Centralized Regional<br>• Coordination team<br>○ Coordinator (P00B22)<br>▪ 1 Social worker<br>○ Manager (P00B22)<br>• Not all MAiD referrals are routed through this service (P00B22)<br>○ Assessments and provisions can be coordinated without the involvement of the care coordination service (P00B22)	• Provide MAiD information (P00B22)<br>• Accept and process referrals (P00B22)<br>• Coordinate assessments and provisions (P00B22)<br>○ Support patients to identify practitioners for assessment (P00B22)<br>• Support completion of forms in compliance with legislation (P00B22)
Northwest Territories	• Territorial care coordination service (11)<br>• Healthcare providers (11)<br>• Website (11)<br>• Printed material<br>○ General Questions (49) (P00A3)<br>○ Information guide (11)	• Email (11)<br>• Telephone (11)<br>• Toll-free number (11)	• Healthcare providers (11)<br>• Self-referral (11)	Centralized Territorial<br>• Coordination team<br>○ Coordinator (Territorial MAiD specialist) (11)<br>▪ 1 Registered nurse (P00A2)<br>• Not all MAiD referrals are routed through this service (P00A2)<br>○ Assessments can be coordinated without the involvement of the care coordination service (P00A2)	• Provide MAiD information (11)<br>• Accept and process referrals (11)<br>• Coordinate assessments and provisions (P00A3)<br>○ Support patients to identify practitioners for assessment (P00A2) (11)<br>• Support completion of forms in compliance with legislation (P00A2)<br>• Provide assistance, support, and/or guidance (P00A2)<br>○ Arrange end of life support to patients and families (P00A3)<br>○ Discuss insurance (P00A3)<br>○ Connect families to support groups (P00A2, P00A3)<br>○ Facilitate education sessions with providers (P00A2)<br>○ Document patients and families' wishes and preferences (P00A2)
Nova Scotia - Nova *Scotia Health*	• Regional care coordination service (12)<br>• Healthcare providers (12, 50)<br>• Website (51)<br>• Printed materials<br>○ MAiD support guide (52) (P00A12)	• Email (51)<br>• Telephone (51)<br>• Toll-free number (51)<br>• Fax (51)	• Healthcare providers (P00C38)<br>• Self-referral (P00A12)	Centralized Provincial<br>• Coordination team<br>○ 4 Coordinators (Nurse navigators) (0.5 FTE)<br>▪ Registered nurses (P00A6)<br>○ 1 unit clerk (P00A6)<br>• All MAiD referrals are routed through this service (P00A6)	• Provide MAiD information (12)<br>• Accept and process referrals (12, 50)<br>• Coordinate assessments and provisions (P00A6)<br>○ Support patients to identify practitioners for assessment (12)<br>• Upload copies of all documentations provided by practitioners to electronic medical record (P00A12)<br>• Assist in identifying independent witness (12, 50)<br>• Provide assistance, support, and/or guidance<br>○ Arrange end of life support to patients and families (12, 50)<br>○ Connect patients and families to support during MAiD process (P00A6)<br>○ Coordinate procedures with staff and pharmacies (12)

Jurisdiction	Where can information initially be accessed?	How can services be contacted?	Who can make the request to start the referral process?	What are the structural elements of care coordination services?	
				Team Structure and Process	Roles and Responsibilities
					○ Provide logistics support (P00C38)<br>○ Facilitate referrals to grief services (12)<br>○ Contact providers when Health Canada reports discrepancies in provided documentation (P00A12)
Prince Edward Island - *Health PEI*	• Provincial care coordination service (53)<br>• Website (53)<br>• Printed materials<br>○ MAiD support guide (54)<br>○ Patient information booklet (P00B8)	• Email (53)<br>• Telephone (53)<br>• Fax (53)	• Healthcare providers (P00B17)<br>• Self-referral (P00B17)	Centralized Provincial<br>• Coordination team<br>○ Coordinator (P00B8)<br>▪ 1 registered nurse (1.0 FTE) (P00B8, P00B17)<br>• All MAiD referrals are routed through this service (P00B8, P00B17)	• Provide MAiD information (P00B8)<br>• Accept and process referrals (P00B8)<br>• Coordinate assessments and provisions (P00B17)<br>○ Contact patient's primary care provider for assessment (P00B8)<br>• Assist in identifying independent witness (P00B17)<br>• Provide assistance, support, and/or guidance<br>○ Connect families with grief and bereavement support services (P00B8)<br>○ Provide logistics support (P00B17)<br>• Coordinate with Allied Health providers and teams for optimal patient care (P00B8)
Saskatchewan - *Saskatchewan Health Authority*	• Provincial care coordination service (55)<br>• Website (55) (P00A1, P00B7)<br>• Printed materials<br>○ Pamphlet (56)<br>○ MAiD support guide (57)	• Telephone (55)<br>• Fax (55)	• Healthcare providers (P00B1, P00B3, P00B7, P00C1)<br>• Self-referral (P00B1, P00B3, P00B7, P00C1)	Centralized Provincial<br>• Coordination team<br>○ Coordinators (P00A1)<br>▪ Registered nurses (P00A1)<br>○ Office administrator (P00A1)<br>• All MAiD referrals are routed through this service (P00A1)	• Provide MAiD information (P00A1)Accept and process referrals (58)<br>• Coordinate assessments and provisions (58) (P00C1, P00C19)<br>• Support completion of forms in compliance with legislation (58) (P00C1)<br>• Coordinate patient transfers (58)<br>• Assist in identifying independent witness (58)<br>• Provide assistance, support, and/or guidance<br>○ Arrange end of life support to patients and families (58)<br>○ Document patient wishes and preferences (P00B7)<br>○ Convey patient preferences to staff (P00B7)
Yukon - *Department of Health and Social Services*	• Healthcare providers (15)<br>• Website (15)	• No centralized point of access for patients (P00C7)	• Healthcare providers (P00C7, P00C14)<br>• Self-referral (P00C7)	• No coordination team (P00C7)	• Not applicable<br>○ No coordination team (P00C7)
Ontario - *Ministry of Health*	• Provincial care coordination service (59)<br>• Website (59)<br>• Printed materials<br>○ Information guide (60)	• Telephone (59)<br>• Email (24)	• Healthcare providers (P00B4)<br>• Self-referral (P00B4)	Decentralized Provincial<br>• Coordination team<br>○ Coordinators (P00C16)<br>▪ 2 Nurse practitioner (P00C16)<br>▪ Registered nurses (P00C16)<br>○ Manager (P00C16)<br>• Not all MAiD referrals are routed through this service (P00C16)	• Provide MAiD information (59)<br>• Accept and process referrals (59)<br>• Coordinate assessments and provisions (59)<br>• Refer cases to regional care coordination services (P00B4)<br>• Coordinate assessments and provisions (59)<br>○ Support patients to identify practitioners for assessment (59)<br>• Communicate with external parties about how MAiD is delivered (P00B4)
SERVICE ORGANIZATIONS/REGIONAL FACILITIES					
Ontario - *Home and Community Care Support Services, Central East*	• Regional care coordination service (59)<br>• Healthcare providers (P00C2)	• Email (61)<br>• Toll-free number (61)<br>• Fax (61)	• Provincial care coordination service (P00C2)<br>• Healthcare providers (P00C2)<br>• No self-referrals accepted (P00C2)	Centralized Regional<br>• Coordination team<br>○ MAiD care coordinator (P00C2)<br>○ Technical Assistants (P00C2)<br>• All MAiD referrals are routed through this service (P00C2)	• Provide MAiD information (P00C2)<br>• Accept and process referrals (P00C2)<br>• Coordinate assessments and provisions (P00C2)<br>○ Support patients to identify practitioners for assessment (P00C2)
Ontario - *Home and Community Care Support Services, Waterloo Region*	• Provincial care coordination service (59)<br>• Healthcare providers (P00C4)	• Email (62)<br>• Toll-free number (62)<br>• Fax (62)	• Provincial care coordination service (P00C4)<br>• Healthcare providers (P00C4)<br>• Self-referral (P00C4)	Centralized Regional<br>• Coordination team<br>○ Coordinators (Nurse navigators) (P00C4)<br>▪ 2 Registered nurses (P00C4)<br>• All MAiD referrals are routed through this service (P00C4)	• Provide MAiD information (P00C4)<br>• Accept and process referrals (P00C4)<br>• Coordinate assessments and provisions (P00C4)<br>○ Support patients to identify practitioners for assessment (P00C4)

Jurisdiction	Where can information initially be accessed?	How can services be contacted?	Who can make the request to start the referral process?	What are the structural elements of care coordination services? Team Structure and Process	Roles and Responsibilities
Ontario - *Home and Community Care Support Services, South East*	• Provincial care coordination service (59)<br>• Healthcare providers (P00C20)	• Email (63)<br>• Toll-free number (63)<br>• Fax (63)	• Provincial care coordination service (P00C20)<br>• Healthcare providers (P00C20)<br>• Self-referral (P00C20)	Centralized Regional<br>• Coordination team<br>○ Coordinators (P00C20)<br>▪ 3 Registered psychiatric nurses (P00C20)<br>• All MAiD referrals are routed through this service (P00C20)	• Provide MAiD information (P00C20)<br>• Accept and process referrals (P00C20)<br>• Coordinate assessments and provisions (P00C20)
Ontario - *Home and Community Care Support Services, South West*	• Provincial care coordination service (59)<br>• Institutional care coordination service (P00C23)<br>• Healthcare providers (P00C23)	• Email (64)<br>• Toll-free number (64)<br>• Fax (64)	• Provincial care coordination service (P00C23)<br>• Healthcare providers (P00C23)<br>• No self-referral accepted (P00C23)	Centralized Regional<br>• Coordination team<br>○ Coordinator (P00C23)<br>▪ 1 Nurse practitioner (1 FTE) (P00C23)<br>▪ 1 Registered nurse (P00C23)<br>• All MAiD referrals are routed through this service (P00C23)	• Provide MAiD information (P00C23)<br>• Accept and process referrals (P00C23)<br>• Coordinate assessments and provisions (P00C23)<br>○ Support patients to identify practitioners for assessment (P00C23)
Ontario - *Champlain Regional MAiD Network, the Ottawa Hospital*	• Provincial coordination service care coordination service (59)<br>• Healthcare providers (P00C5)<br>• Website (65)	• Telephone (66)<br>• Toll-free number (66)<br>• Fax (66)	• Provincial coordination service care coordination service (P00C5)<br>• Healthcare providers (P00C5)<br>• No self-referrals accepted (P00C5)	Centralized Regional<br>• Coordination team<br>○ 1 Advanced practice nurse and Program Manager (P00C5)<br>○ Coordinators (Nurse navigators) (P00C5)<br>▪ 2 Registered nurses (P00C5)<br>○ 1 MAiD coordinator (P00C5)<br>○ 1 Administrative assistant (P00C5)<br>• All MAiD referrals are routed through this service (P00C5)	• Provide MAiD information (P00C5)<br>• Accept and process referrals (P00C5)<br>• Coordinate assessments and provisions (P00C5)<br>• Support completion of forms in compliance with legislation (P00C5)<br>• Provide assistance, support, and/or guidance<br>○ Provide logistics support (P00C5)<br>○ Document patients and families' wishes and preferences (P00C5)
HEALTHCARE FACILITIES					
Ontario - *Peterborough Regional Health Centre*	• Provincial care coordination service (59)<br>• Institutional care coordination service (P00C3)<br>• Website (67)<br>• Printed materials<br>○ Brochure (P00C3)	• Email (61)<br>• Toll-free number (61)	• Provincial care coordination service (P00C3)<br>• Healthcare providers (P00C3)<br>• Self-referral (P00C3)	Centralized Institutional<br>• Coordination team<br>○ Coordinator (Patient relations consultant) (P00C3)<br>▪ 1 Registered nurse (P00C3)<br>• All MAiD referrals are routed through this service (P00C3)	• Provide MAiD information (P00C3)<br>• Accept and process referrals (P00C3)<br>• Coordinate assessments and provisions (P00C3)
Ontario - *Mount Sinai Healthcare Facility, Toronto*	• Provincial care coordination service (59)<br>• Healthcare providers (P00C36)	• Email (68)<br>• Toll-free number (68)<br>• Fax (68)	• Provincial care coordination service (P00C36)<br>• Healthcare providers (P00C36)<br>• Self-referral (P00C36)	Centralized Institutional<br>• Coordination team<br>○ Coordinator (P00C36)<br>▪ 2 Registered nurses (<1 FTE) (P00C36)<br>▪ 1 Bioethicist (P00C36)<br>• All MAiD referrals are routed through this service (P00C36)	• Provide MAiD information (P00C36)<br>• Accept and process referrals (P00C36)<br>• Coordinate assessments and provisions (P00C36)<br>• Collect and organize data from forms (P00C36)
Ontario - *University Health Network*	• Provincial care coordination service (59)<br>• Healthcare providers (P00C8)<br>• Website (69)	• Telephone (69)<br>• Other<br>○ Contact through healthcare team (69)	• Healthcare providers<br>○ Only most responsible physician in a network hospital can refer (P00C8)<br>• No self-referral or external referrals accepted (P00C8)	Centralized Institutional<br>• Coordination team<br>○ Coordinator<br>▪ MAiD clinical nurse specialist (P00C8)<br>- 1 Advanced practice nurse (P00C8)<br>▪ MAiD clinical coordinator (P00C8)<br>- 1 Registered nurse (P00C8)<br>○ 1 MAiD administrative assistant (P00C8)<br>• All MAiD referrals are routed through this service (P00C8)	• Provide MAiD information (P00C8)<br>• Accept and process referrals (P00C8)<br>• Coordinate assessments and provisions (P00C8)<br>• Provide assistance, support, and/or guidance<br>○ Facilitate educational support to staff (P00C8)<br>○ Provide logistic support (P00C8)<br>○ Support inter-professional collaboration (P00C8)<br>○ Support medication safety checks and preparations (P00C8)<br>○ Support staff and teams navigate MAiD process (P00C8)
Ontario - *Grand River Hospital*	• Healthcare providers (P00C50)	• Healthcare providers (P00C50)	• Healthcare providers (P00C50)<br>• Self-referral (P00C50)	Centralized Institutional<br>• Coordination team<br>○ Coordinator<br>▪ 1 Nurse practitioner (P00C50)<br>• All MAiD referrals are routed through this service (P00C50)	• Provide MAiD information (P00C50)<br>• Accept and process referrals (P00C50)<br>• Coordinate assessments and provisions (P00C50)<br>• Provide assistance, support, and/or guidance<br>○ Facilitate educational support to staff (P00C50)<br>○ Provide logistics support (P00C50)<br>○ Document patient wishes and preferences (P00C50)<br>○ Support staff and teams navigate MAiD process (P00C50)

Jurisdiction	Where can information initially be accessed?	How can services be contacted?	Who can make the request to start the referral process?	What are the structural elements of care coordination services? Team Structure and Process	Roles and Responsibilities
COMMUNITY OF PRACTICE					
Ontario - *Hamilton Family Health Team*	• Provincial care coordination service (59)<br>• Regional care coordination service (P00B18)<br>• Website (P00B18)	• Email (70)<br>• Toll-free number (70)<br>• Fax (70)	• Provincial care coordination service (P00B18)<br>• Healthcare providers (P00B18)<br>• Self-referral (P00B18)	Centralized Regional<br>• Coordination team<br>○ Coordinators (P00C32)<br>▪ 1 Registered nurse (0.5 FTE) (P00B18)<br>▪ 1 Registered practical nurse (1 FTE) (P00B18)<br>○ 1 Manager (0.5FTE) (P00B18)<br>• All MAiD referrals are routed through this service (P00B18)	• Provide MAiD information (P00B18)<br>• Accept and process referrals (P00B18)<br>• Coordinate assessments and provisions (P00B18)<br>○ Support patients to identify practitioners for assessment (P00B18)<br>• Support completion of forms in compliance with legislation (P00B18)<br>• Assist in identifying independent witness (P00B18)
Ontario - *Niagara Community MAiD Team, St. Catharine's, Niagara*	• Provincial care coordination service (59)<br>• Healthcare providers (P00C11)	• Email (70)<br>• Toll-free number (70)<br>• Fax (70)	• Provincial care coordination service (P00C11)<br>• Healthcare providers (P00C11)<br>• Self-referral (P00C11)	• No coordination team (P00C11)	• Not applicable<br>○ No coordination team (P00C11)
GEOGRAPHICAL AREAS					
Ontario - *Oakville & Mississauga area*	• Provincial care coordination service (59)<br>• Healthcare providers (P00C18)	• Other<br>○ Contact through provincial care coordination service (P00C18)<br>○ Contact through the regional care coordination service<br>○ Contact through the institutional care coordination service (P00C18)<br>○ Healthcare providers (P00C18)	• Provincial care coordination service (P00C18)<br>• Regional care coordination service (P00C18)<br>• Institutional care coordination service (P00C18)<br>• Healthcare providers (P00C18)<br>• Self-referral (P00C18)	• No coordination team (P00C18)	• Not applicable<br>○ No coordination team (P00C18)
Ontario - *Oakville & Mississauga Area, Burlington area*	• Provincial care coordination service (59)<br>• Healthcare providers (P00C21)	• Other<br>○ Contact through provincial care coordination service (P00C21)<br>○ Contact through the regional care coordination service (P00C21)<br>○ Healthcare providers (P00C21)	• Provincial care coordination service (P00C21)<br>• Regional care coordination service (P00C21)<br>• Healthcare providers (P00C21)<br>• Self-referral (P00C21)	• No coordination team (P00C21)	• Not applicable<br>○ No coordination team (P00C21)
Ontario - *Renfrew County*	• Provincial care coordination service (59)<br>• Healthcare providers (P00C22)	• Other<br>○ Contact through provincial care coordination service (P00C22)<br>○ Contact through Champlain regional MAiD network (P00C22)<br>○ Healthcare providers (P00C22)	• Provincial care coordination service (P00C22)<br>• Champlain regional MAiD network (P00C22)<br>• Healthcare providers (P00C22)<br>• Self-referral P00C22)	• No coordination team (P00C22)	• Not applicable<br>○ No coordination team (P00C22)

Jurisdiction	Where can information initially be accessed?	How can services be contacted?	Who can make the request to start the referral process?	What are the structural elements of care coordination services?	
				**Team Structure and Process**	**Roles and Responsibilities**
Ontario - *Greater Toronto Area*	• Provincial care coordination service (59)<br>• Healthcare providers (P00C24)	• Other<br>○ Contact through provincial care coordination service (P00C24)<br>○ Healthcare providers (P00C24)	• Provincial care coordination service (P00C24)<br>• Healthcare providers (P00C24)	• No coordination team (P00C24)	• Not applicable<br>○ No coordination team (P00C24)
Ontario - *Waterloo area*	• Provincial care coordination service (59)<br>• Healthcare providers (P00C25)	• Other<br>○ Contact through provincial care coordination service (P00C24)<br>○ Contact through regional care coordination service (P00C25)<br>○ Healthcare providers (P00C25)	• Provincial care coordination service (P00C25)<br>• Regional care coordination service (P00C25)<br>• Healthcare providers (P00C25)<br>• No self-referrals accepted (P00C25)	• No coordination team (P00C25)	• Not applicable<br>○ No coordination team (P00C25)
Ontario - *Noelville, Sudbury, Elliot Lake, Sturgeon Falls area*	• Provincial care coordination service (59)<br>• Healthcare providers (P00C26)	• Other<br>○ Contact through provincial care coordination service (P00C26)<br>○ Healthcare providers (P00C26)	• Provincial care coordination service (P00C26)<br>• Healthcare providers (P00C26)<br>• Self-referral (P00C26)	• No coordination team (P00C26)	• Not applicable<br>○ No coordination team (P00C26)
Québec - *Ministry of Health and Social Services*	• Website (71)	• Telephone (71)	• Not applicable<br>○ Decentralized into regions	• Not applicable<br>○ Decentralized into regions	• Not applicable<br>○ Decentralized into regions
INTEGRATED HEALTH AND SOCIAL SERVICES CENTRES					
Québec – *CISS Montérégie*	• Website (72)<br>• Healthcare providers (P00C27)	• Telephone (P00C27)<br>• Other<br>○ Healthcare providers (P00C27)<br>○ Local health or social services (P00C27)	• Healthcare providers (P00C27)<br>• Local health or social services (P00C27)	Centralized Regional<br>• Coordination team<br>○ Coordinator (P00C27)<br>▪ Registered nurse (P00C27)<br>• Not all MAiD referrals are routed through this service (P00C27)<br>○ Assessments and provisions can be coordinated without the involvement of the care coordination service (P00C27)	• Provide MAiD information (P00C27)<br>• Accept and process referrals (P00C27)<br>• Coordinate assessments and provisions (P00C27)<br>• Review and update status of MAiD requests (P00C27)<br>• Provide assistance, support, and guidance (P00C27)<br>○ Support physicians with resources to meet patient demands (P00C27)<br>○ Provide logistical support for provision day (P00C27)
INTEGRATED UNIVERSITY HEALTH AND SOCIAL SERVICES CENTRES					
Québec – *CIUSS Capitale-Nationale*	• Website (73)<br>• Healthcare providers (P00C37)	• Telephone (73)<br>• Other<br>○ Healthcare providers (73)<br>○ Local health or social services (P00C37)	• Healthcare providers (73)<br>• Local health or social services (P00C37)	Centralized Regional<br>• Coordination team<br>○ Coordinator (16)<br>▪ Clinical nurse (16) (P00C37)<br>• Not all MAiD referrals are routed through this service (P00C37)<br>○ Assessments and provisions can be coordinated without the involvement of the care coordination service (P00C37)	• Provide MAiD information (16)<br>• Accept and process referrals (16)<br>• Coordinate assessments and provisions (16)<br>• Provide assistance, support, and/or guidance (16)<br>○ Provide support with reporting platform (16)

Jurisdiction	Where can information initially be accessed?	How can services be contacted?	Who can make the request to start the referral process?	What are the structural elements of care coordination services?	
				**Team Structure and Process**	**Roles and Responsibilities**
Québec – *University of Montreal Hospital Center*	• Website (74)<br>• Healthcare providers (P00C40)	• Email (74)<br>• Telephone (P00C40)<br>• Other<br>○ Healthcare providers (P00C40)	• Healthcare providers (P00C40)	Centralized Institutional<br>• Coordination team<br>○ Coordinator (P00C40)<br>▪ Registered nurse (P00C40)<br>• All MAiD referrals are routed through this service (P00C40)	• Provide MAiD information (P00C40)<br>• Accept and process referrals (P00C40)<br>• Coordinate assessments and provisions (P00C40)<br>○ Support patients to identify practitioners for assessment and provision (P00C40)
Québec – *McGill University Health Centre*	• Website (75)<br>• Healthcare providers (P00C9)	• Email (75) (P00C9)<br>• Telephone (P00C9)<br>• Other<br>○ Healthcare providers (P00C9)	• Healthcare providers (P00C9)	Centralized Institutional<br>• Coordination team<br>○ Coordinator (P00C9)<br>▪ Advance practice nurse (P00C9)<br>○ Administrative assistant (P00C9)<br>• All MAiD referrals are routed through this service (P00C9)	• Provide MAiD information (P00C9)<br>• Accept and process referrals (P00C9)<br>• Coordinate assessments and provisions (P00C9)<br>○ Support patients to identify practitioners for assessment and provision (P00C9)<br>• Provide assistance, support, and/or guidance (P00C9)<br>○ Connect families to support groups (P00C9)<br>○ Facilitate education sessions with providers (P00C9)

Table 6: Preliminary assessment and intake process

Jurisdiction	In what form are requests received?	What does the intake process involve?	What are the common elements of the intake process	What does the preliminary assessment involve?	What is done once a patient is found ineligible at the referral stage?	Who is responsible for reporting ineligible cases at the referral stage?
Alberta - *Alberta Health Services*	• Written (76, 77)	Location<br>• Virtual (P00A5, P00C6, P00B9)<br>Who<br>• Coordinators (Navigators) (P00A5, P00C6, P00B9)<br>Process<br>1. Inform and screen patients against federal criteria (P00B9, P00B13, P00A5, P00C39)<br>2. Support patients in understanding how health conditions align with federal criteria (P00B13)<br>3. Review patients' files after obtaining formal requests (P00B13)<br>4. Triage patients and coordinate their assessments (P00B9, P00B13, P00C39)<br>5. Collaborate with social workers and unit managers (P00C6)<br>6. Connect families to support resources (P00B13)<br>7. Review forms for accuracy and completeness (P00C39)<br>8. Confirm consent to continue with MAiD process after completion of record of request (P00C39)	• Screening<br>○ Screen against federal criteria (P00B9, P00B13, P00A5, P00C39)<br>• Patient information and consent<br>○ Confirm consent (P00C39)<br>• Communication with patients and families<br>○ Inform patients of federal criteria (P00B13)<br>• Coordination<br>○ Coordinate assessments (P00B9, P00B13, P00C39)<br>○ Collaborate with social workers and unit managers (P00C6)<br>• Triage and priority setting<br>○ Triage patients, (P00B9, P00B13, P00C39)<br>• Form review and documentation<br>○ Review forms for accuracy and completeness (P00B13)<br>• Support services<br>○ Connect family to support resources (P00B13)	• No preliminary assessment (P00A5, P00C39, P00B13)	• Coordinators discuss complex or unclear cases with medical lead, or send cases to assessors (P00C39)<br>• Re-evaluation of ineligible patients possible at any time if condition declines (P00C10)	• Not applicable<br>○ No reporting is completed without an assessment
British Columbia - *Ministry of Health*	• Written (78-80)<br>○ Email or text (78, 81)<br>• Verbal<br>○ Discussion (78, 81)	• Not applicable	• Not applicable	• No preliminary assessment province wide (P00A4, P00B16)	• Not applicable	• Not applicable<br>○ No reporting is completed without an assessment
British Columbia - *Fraser Health*	• Written (6, 80)	Who<br>• Coordinators (P00A10)<br>Process<br>1. Contact applicants upon submission of request forms (P00A10)<br>2. Determine necessity of additional supports (P00A10)<br>3. Inquire assessment preferences (P00A10)<br>4. Contact patients' primary care provider or specialists for first assessments (P00A10)<br>○ Alternatively, team can assign both assessors from MAiD team	• Patient information and consent<br>• Communication with patients and families<br>○ Contact applicants upon submission of request form (P00A10)<br>○ Inquire assessment preferences (P00A10)<br>• Coordination<br>○ Contact patients' primary care provider or specialists for first assessment (P00A10)<br>• Support services<br>○ Determine necessity of additional supports (P00A10)	• No preliminary assessment province wide (P00A4, P00B16)	• Not applicable<br>○ Ineligibility is not determined at referral	• Not applicable<br>○ No reporting is completed without an assessment

Jurisdiction	In what form are requests received?	What does the intake process involve?	What are the common elements of the intake process	What does the preliminary assessment involve?	What is done once a patient is found ineligible at the referral stage?	Who is responsible for reporting ineligible cases at the referral stage?
British Columbia - *Interior Health*	• Written (32, 34, 80)	Who<br>• Coordinators (P00B14)<br>Process<br>1. Contact applicants upon submission of request forms (P00B14)<br>2. Determine priority of cases (P00B14)<br>3. Invite appropriate primary care provider to be assessors and providers (P00B14)	• Communication with patients and families<br>○ Contact applicants upon submission of request form (P00B14)<br>• Coordination<br>○ Invite appropriate primary care providers to be assessors and providers (P00B14)<br>• Triage and priority setting<br>○ Determine priority of cases (P00B14)	• No preliminary assessment province wide (P00A4, P00B16)	• Not applicable<br>○ Ineligibility is not determined at referral	• Not applicable<br>○ No reporting is completed without an assessment
British Columbia - *Island Health*	• Written (35, 80)	Who<br>• Coordinators (Navigators) (P00A9)<br>• Independent physicians (P00A9)<br>○ Community of practice<br>Process<br>1. Review forms for accuracy and completeness (P00A9)<br>2. Contact applicants upon submission of request forms (P00A9)<br>3. Contact patients' primary care provider or local clinicians who have assessed and provided MAiD for first assessments (P00A9)<br>○ Alternatively, team can assign both assessors from MAiD team	• Communication with patients and families<br>○ Contact applicants upon submission of request form (P00A9)<br>• Coordination<br>○ Contact patients' primary care provider or specialists for first assessment (P00A9)<br>• Form review and documentation<br>○ Review forms for accuracy and completeness (P00A9)	• No preliminary assessment province wide (P00A4, P00B16)	• Not applicable<br>○ Ineligibility is not determined at referral	• Not applicable<br>○ No reporting is completed without an assessment
British Columbia - *Northern Health*	• Written (37, 80)	• Intake process not specified	• Not applicable<br>○ Intake process not specified	• No preliminary assessment province wide (P00A4, P00B16)	• Not applicable<br>○ Ineligibility is not determined at referral	• Not applicable<br>○ No reporting is completed without an assessment
British Columbia - *Vancouver Coastal Health*	• Written (38, 40, 80)	Who<br>• Coordinators (P00B16)<br><br>Process (Triage) (P00B16)<br>1. Review forms for accuracy and completeness (P00B12)<br>2. Determine priority of cases (P00A7)<br>3. Contact patients' primary care provider or specialists for first assessments (P00B12)<br>○ Alternatively, team can assign both assessors from MAiD team<br>4. Update and inform patients and families about processes (P00B12)<br>5. Coordinate assessments and provisions (P00B12)<br>6. Determine necessity of additional supports (P00B12)	• Communication with patients and families<br>○ Update and inform patients about processes (P00B12)<br>• Coordination<br>○ Contact patients' primary care provider or specialists for first assessments (P00B12)<br>○ Coordinate assessments and provisions (P00B12)<br>• Triage and priority setting<br>○ Determine priority (P00A7)<br>• Form review and documentation<br>○ Review forms for accuracy and completeness (P00B12)<br>• Support services<br>○ Determine necessity of additional supports (P00B12)	• No preliminary assessment province wide (P00A4, P00B16)	• Not applicable<br>○ Ineligibility is not determined at referral	• Not applicable<br>○ No reporting is completed without an assessment
British Columbia - *Provincial Health Services Authority*	• Written (80)	• Intake process not specified	• Not applicable<br>○ Intake process not specified	• No preliminary assessment province wide (P00A4, P00B16)	• Not applicable<br>○ Ineligibility is not determined at referral	• Not applicable<br>○ No reporting is completed without an assessment

Jurisdiction	In what form are requests received?	What does the intake process involve?	What are the common elements of the intake process	What does the preliminary assessment involve?	What is done once a patient is found ineligible at the referral stage?	Who is responsible for reporting ineligible cases at the referral stage?
Manitoba - *Shared Health*	• Written (82)<br>• Verbal<br>○ Patient must explicitly ask for MAiD directly to their healthcare provider (P00C34)<br>○ Patient gives permission for team to access medical records (42) (P00C33)	Location<br>• Virtual (P00C34)<br>Who<br>• Office administrators (P00C34)<br>• Registered nurses (P00B19) coordinators<br>Intake process<br>○ Only to determine potential for eligibility (P00B19)<br>1. Receive calls for MAiD inquiries, and inform them about federal eligibility criteria (P00C34)<br>○ Complete forms with basic demographic information (P00B19)<br>2. Send eligible, or close to eligible, cases to triage (P00B19, P00B21, P00C34, P00C33)<br>Triage process<br>3. Complete triage/preliminary assessment form (P00C34, P00C33)<br>4. Ask patients' primary care providers for information surrounding request for MAiD via letters (P00C33)<br>5. Connect with patients and families to better understand their cases (P00C33)<br>6. Determine priority for cases (P00C33)<br>7. Review psychosocial aspects of cases (P00B19)<br>8. Determine necessity of additional supports<br>○ Language consults with speech language pathologist (P00C34)<br>9. Coordinate assessments (P00C33)<br>10. Review patient medical charts P00C33, P00B19)	• Screening<br>○ Yes (P00B19)<br>• Patient information and consent<br>○ Ask patients' primary care providers for information surrounding request for MAiD via letters (P00C33)<br>○ Review psychosocial aspects of cases (P00B19<br>○ Review patient medical charts (P00B19)<br>• Communication with patients and families<br>○ Connect with patients and families to better understand their cases (P00C33)<br>• Coordination<br>○ Connect with practitioners to schedule patient's assessment (P00C33)<br>• Triage and priority setting<br>○ Complete triage/preliminary assessment form (P00C34, P00C33)<br>○ Send eligible, or close to eligible, cases to triage (P00C34, P00C33, P00B19, P00B21)<br>○ Determine priority for cases (P00C33)<br>• Form review and documentation<br>○ Form with basic demographic information (P00B19)<br>• Support services<br>○ Consult with speech language pathologist (P00C34)	• Potential for eligibility is determined during initial triage (P00C34, P00B19)	• Social workers or coordinators inform Medical Director of ineligible cases (P00C34, P00B19)<br>• Coordinators directly refer patients and families to support services (P0034)	• Medical Director (P00B19, P00B21, P00C33, P00C34)
New Brunswick - *Horizon Health Network*	• Written (P00C15)<br>• Verbal<br>○ Patient must explicitly ask for MAiD directly to their primary care provider (P00C15)	Who<br>• Coordinators (Navigators) (P00B11)<br>○ Intake is not completed for all referrals (P00C15)<br>Process<br>1. Complete form with basic demographic information (P00B11)<br>2. Refer to needed services (P00B11)<br>○ For example, MAiD family support, organization links, and a link to Bridge C-14 (P00B11)<br>3. Inform patients and families about processes (P00B11)<br>4. Discuss patients and families' expectations about processes (P00B11)<br>5. Connect with patients and families to understand family dynamics (P00B11)<br>6. Review patients' medical information in detail (P00C15)	• Patient information and consent<br>○ Review patients' medical information in detail (P00C15)<br>○ Connect with patients' health care provider or specialist (P00C15)<br>• Communication with patients and families<br>○ Inform patients about processes (P00B11)<br>○ Discuss patients and families' expectations about processes (P00B11)<br>○ Connect with patients to understand family dynamics (P00B11)<br>• Form review and documentation<br>○ Complete form with basic demographic information (P00B11)<br>• Support services<br>○ Refer to needed services (P00B11)	• Potential for eligibility is determined during initial intake, ineligible cases reported to medical lead or continued onto assessment process (P00B11)	• Coordinators discuss complex or unclear cases of eligibility with medical lead or send them to an assessor (P00B11)	• Coordinator (Regional lead)<br>○ Only for defined ineligible patients to Health Canada, (e.g., if they are underage or have no medical condition) (P00B11)

Jurisdiction	In what form are requests received?	What does the intake process involve?	What are the common elements of the intake process	What does the preliminary assessment involve?	What is done once a patient is found ineligible at the referral stage?	Who is responsible for reporting ineligible cases at the referral stage?
		7. Connect with patients' health care providers or specialists (P00C15)				
New Brunswick - *Vitalité Health Network*	• Written (P00C48) (83)	• Intake process not specified	• Not applicable<br>○ Intake process not specified	• No preliminary assessment completed (P00C48)	• Not applicable<br>○ Ineligibility is not determined at referral (P00C48)	• Not applicable<br>○ No preliminary assessment completed (P00C48)
Newfoundland and Labrador - *Department of Health and Community Services*	• Not applicable<br>○ Decentralized into zones	• Not applicable<br>○ Decentralized into zones	• Not applicable<br>○ Decentralized into zones	• Not applicable<br>○ Decentralized into zones	• Not applicable<br>○ Decentralized into zones	• Not applicable<br>○ Decentralized into zones
Newfoundland and Labrador - *Eastern Zone*	• Written (84)	Who<br>• Coordinators (Navigators) (P00C43)<br>Process<br>1. Accept requests (P00C43)<br>2. Disseminate emails to assessors (P00C43)<br>3. Discuss eligibility criteria (P00C43)<br>4. Provide information about MAiD (P00C43)	• Screening<br>○ Discuss eligibility criteria (P00C43)<br>• Communication with patients and families<br>○ Provide information about MAiD (P00C43)<br>• Coordination<br>○ Disseminate emails to assessors (P00C43)	• Potential for eligibility is determining during initial triage; ineligible cases are sent to Medical Director (P00C43)	• Coordinators inform medical lead of ineligible cases (P00C43)	• Physician (P00C43)<br>○ Medical Director (P00C43)
Newfoundland and Labrador - *Western Zone*	• Written (84)	Who<br>• Coordinators (Navigators) (P00C44)<br>Process<br>1. Accept requests (P00C44)<br>2. Disseminate emails to assessors (P00C44)<br>3. Discuss eligibility criteria (P00C44)<br>4. Provide information about MAiD (P00C44)	• Screening<br>○ Discuss eligibility criteria (P00C44)<br>• Communication with patients and families<br>○ Provide information about MAiD (P00C44)<br>• Coordination<br>○ Disseminate emails to assessors (P00C44)	• No preliminary assessment (P00C44)	• Not applicable<br>○ Ineligibility is not determined at referral	• Physicians and nurse practitioners (P00C4)<br>○ No reporting is completed without an assessment
Newfoundland and Labrador - *Central Zone*	• Written (84)	Who<br>• Coordinators (Navigators) (P00B22)<br>Process<br>1. Contact applicants upon submission of request forms (P00B22)<br>2. Contact patients' primary care providers or specialists for first assessments (P00B22)	• Communication with patients and families<br>○ Contact applicants upon submission of request forms (P00B22)<br>• Coordination<br>○ Contact patients' primary care providers or specialists for first assessments (P00B22)	• No preliminary assessment (P00B22)	• Not applicable<br>○ Ineligibility is not determined at referral	• Not applicable<br>○ No reporting completed without an assessment<br>○ Coordinators cannot determine ineligibility and do not report (P00B22)
Northwest Territories	• Written (11, 85)	Who<br>• Coordinators (Navigators) (P00A2)<br>○ Territorial MAiD specialist (P00A3)<br>Process<br>• Review patients' chart and past medical history (P00A3)<br>• Support patients and families to understand timelines and what to expect (P00A3)<br>• Complete intake form (P00A2)	• Patient information and consent<br>○ Review patients' charts and past medical history<br>• Communication with patients and families<br>○ Support patients and families to understand timelines and what to expect (P00A3)<br>• Form review and documentation<br>○ Complete intake form (P00A2)	• No preliminary assessment (11)	• Not applicable<br>○ Ineligibility is not determined at referral	• Not applicable<br>○ No reporting is completed without an assessment

<table>
<tr><th>Jurisdiction</th><th>In what form are requests received?</th><th>What does the intake process involve?</th><th>What are the common elements of the intake process</th><th>What does the preliminary assessment involve?</th><th>What is done once a patient is found ineligible at the referral stage?</th><th>Who is responsible for reporting ineligible cases at the referral stage?</th></tr>
<tr><td>Nova Scotia - Nova Scotia Health</td><td>• Written (12, 50, 86)</td><td>Location
• Virtual (P00A6)
Who
• Coordinators (Navigators) (P00A12)

Process
1. Contact patients who have requested MAiD (P00A12)
2. Capture information using MAiD Nurse navigator intake form (P00A12)
○ These MAiD documents are sent to health information service (P00A12)
3. Review patients' charts and past medical history (P00A6)
4. Support patients and families to understand timelines and what to expect (P00A6)
5. Triage track 1 and 2 cases (P00A6)
6. Ask for consent for participation in clinical studies (P00A12)
7. Document patient and family wishes (P00C38)
8. Inquire preferences about location and time of provisions, and funeral arrangements (P00C38)
9. Inquire necessity of bereavement supports (P00A12)
10. Provide support in coordinating assessments (P00C38)</td><td>• Patient information and consent
○ Review patients' charts and past medical history (P00A6)
○ Ask for consent for participation in clinical studies (P00A12)
• Communication with patients and families
○ Contact patients who have requested MAiD (P00A12)
○ Support patients' and families in understand timelines and what to expect (P00A6)
○ Document patient and family wishes (P00C38)
○ Inquire preferences about location and time of provisions, and funeral arrangements (P00C38)
• Coordination
○ Provide support in coordinating assessments (P00C38)
• Triage and priority setting
○ Triage track 1 and 2 cases (P00A6)
• Form review and documentation
○ MAiD Nurse navigator intake form (P00A12)
▪ These MAiD documents are sent to health information service (P00A12)
• Support services
○ Inquire necessity of bereavement supports (P00A12)</td><td>• No preliminary assessment (P00A12)</td><td>• Not applicable
○ Ineligibility is not determined at referral</td><td>• Not applicable
○ No reporting is completed without an assessment
○ Coordinators cannot determine ineligibility and do not report (P00A12)</td></tr>
<tr><td>Prince Edward Island - Health PEI</td><td>• Written (53, 87) (P00B17)</td><td>Who
• Coordinator (Navigator) (P00B17)
○ Registered nurse (P00B8)
Process
1. Review patients' charts to determine whether patient likely meets criteria (P00B8)
2. Complete pre-assessment using specific intake forms (P00B17)
3. Assess priority levels of cases (P00B8)
4. Determine whether assessments or education sessions are required (P00B8)
5. Contact primary care providers who sent referrals (P00B8)
6. Review information to determine patient's status and reasons for requests (P00B8)</td><td>• Screening
○ Review patients' charts to determine whether patient likely meets criteria (P00B8)
○ Review information to determine patient's status and reasons for requests (P00B8)
○ Determine whether assessments or education sessions are required (P00B8)
• Patient information and consent
○ Contact primary care providers who sent referrals (P00B8)
• Triage and priority setting
○ Assess priority levels of cases (P00B8)
▪ Completion in conjunction with the physician lead (P00B8)
• Form review and documentation
▪ Complete pre-assessment using specific intake forms (P00B17)</td><td>• No preliminary assessment (P00B17)</td><td>• Not applicable
○ Ineligibility is not determined at an referral (P00B17)
• If patient is clearly ineligible, coordinator will inform them that they cannot connect them with an assessor at that current point in time (P00B17)
• Ineligible cases are reviewed by medical lead (P00B8, P00B17)</td><td>• Not applicable
○ No reporting is completed without an assessment
○ Coordinators cannot determine ineligibility and do not report (P00B17)</td></tr>
<tr><td>Saskatchewan - Saskatchewan Health Authority</td><td>• Written (88) (P00A1, P00B1, P00C19)</td><td>Location
• Virtual (P00A1)
• In person (P00A1)</td><td>• Patient information and consent
○ Patients' medical backgrounds and histories are collected (P00A1)</td><td>• Coordinators (Registered nurses) act as preliminary assessors (P00A1)</td><td>• If patient is clearly ineligible, coordinator will inform them that</td><td>• Coordinator (Registered nurse) (P00A1)</td></tr>
</table>

Jurisdiction	In what form are requests received?	What does the intake process involve?	What are the common elements of the intake process	What does the preliminary assessment involve?	What is done once a patient is found ineligible at the referral stage?	Who is responsible for reporting ineligible cases at the referral stage?
		Who<br>• Coordinators (Navigators) (P00B1, P00A1)<br>○ Registered nurse<br>• Office assistant (P00B1, P00A1) or<br>• Nurse practitioner (P00B1, P00A1)<br>Process<br>1. Collect information using an intake form (P00A1, P00C1)<br>2. Provide information sessions to patients and families (P00A1)<br>3. Triage patients on basis of recommendations of a registered nurse who reviewed intake forms (P00A1)<br>4. Collect patients' medical backgrounds and histories (P00A1)<br>5. Consult patients' primary care providers for information (P00A1)<br>6. Discuss options to relieve suffering (P00A1, P00C19)<br>7. Review complex cases (P00A1)	○ Patients' primary care providers are consulted for information (P00A1)<br>• Communication with patients and families<br>○ Discuss options to relieve suffering (P00A1, P00C19)<br>• Triage and priority setting<br>○ Patients are triaged on basis of recommendations of a coordinator who reviewed intake forms (P00A1)<br>○ Review complex cases (P00A1)<br>• Form review and documentation<br>○ Intake form collects federal data related to<br>▪ Palliative care (P00A1, P00C1)<br>▪ Indigenous identity (P00A1)<br>▪ Racial, ethnic and cultural group (P00A1)<br>▪ Disability (P00A1)<br>▪ Usual place of residence (P00A1)<br>• Educational sessions<br>○ Team provides information sessions to patients and families (P00A1)	○ Limited rejection of patients due to inability of registered nurses to diagnose mental health or addiction (P00A1)	they cannot connect them with an assessor at that point (P00A1)<br>• Coordinators discuss complex or unclear cases of eligibility with medical lead or send them to an assessor (P00A1)	• Health Information Management Practitioner (P00A1)<br>○ If patients do not meet all the eligibility criteria (P00A1)
Yukon - *Department of Health and Social Services*	• Written (89, 90)	• Intake process not specified	• Not applicable<br>○ Intake process not specified	• Referrals go directly to practitioners for assessment (P00C7)	• Not applicable<br>○ Ineligibility is not determined at referral	• Not applicable<br>○ No reporting is completed without an assessment
Ontario - *Ministry of Health*	• Verbal (91)<br>• Written (91)	Who<br>• Coordinators (Navigators) (P00C16)<br>Process<br>1. Gather patient information (P00C16)<br>2. Contact patients' primary care providers (P00C16)	• Patient information and consent<br>○ Gather information from patients (P00C16)<br>○ Contact patients' primary care providers (P00C16)	• No preliminary assessment (P00B4)	• Not applicable<br>○ Ineligibility is not determined at referral	• Not applicable<br>○ No reporting is completed without an assessment
SERVICE ORGANIZATIONS/REGIONAL FACILITIES						
Ontario - *Home and Community Care Support Services, Central East*	• Written (P00C2)	• Intake process not specified	• Not applicable	• Not applicable	• Not applicable	• Not applicable<br>○ No reporting is completed without an assessment
Ontario - *Home and Community Care Support Services, Waterloo Region*	• Written (P00C4)	Who<br>• MAiD team (P00C18)<br>Process<br>• Screen patient eligibility (P00C4)	• Screening<br>○ Screen patient eligibility (P00C4)	• No preliminary assessment (P00C4)	• Information not specified	• Not applicable<br>○ No reporting is completed without an assessment
Ontario - *Home and Community Care Support Services, South East*	• Written (P00C20)	Process<br>• Gather demographic information (P00C20)	• Patient information and consent<br>○ Gather demographic information (P00C20)	• No preliminary assessment (P00C20)	• Not applicable<br>○ Ineligibility is not determined at referral (P00C20)	• Not applicable<br>○ No reporting is completed without an assessment
Ontario - *Home and Community Care Support Services, South West*	• Written (P00C23)	Who<br>• Assessors (P00C23)<br>Process<br>• Assessors or providers are responsible for gathering details (P00C23)	• Patient information and consent<br>○ Assessors or providers are responsible for gathering details (P00C23)	• No preliminary assessment (P00C23)	• Not applicable<br>○ Ineligibility is not determined at referral (P00C23)	• Not applicable<br>○ No reporting is completed without an assessment

Jurisdiction	In what form are requests received?	What does the intake process involve?	What are the common elements of the intake process	What does the preliminary assessment involve?	What is done once a patient is found ineligible at the referral stage?	Who is responsible for reporting ineligible cases at the referral stage?
Ontario - *Champlain Regional MAiD Network, the Ottawa Hospital*	• Written (P00C5)	Process<br>• Intake process involves<br>○ Communicate with families and patients (P00C5)<br>○ Clarify legal requirements (P00C5)<br>○ Set expectations (P00C5)<br>○ Triage and screen patients (P00C5)<br>• Support completion of written requests (P00C5)	• Screening<br>○ Yes (P00C5)<br>• Communication with patients and families<br>○ Communicate with patients and families (P00C5)<br>○ Clarify legal requirements (P00C5)<br>○ Set expectations (P00C5)<br>• Triage and priority setting<br>○ Yes (P00C5)<br>• Form review and documentation<br>○ Support completion of written requests (P00C5)	• Potential for eligibility is determined during initial intake (P00C5)	• Information not specified	• Advanced practice nurse/program manager (P00C5)<br>○ If patients do not meet all the eligibility criteria
HEALTHCARE FACILITIES						
Ontario - *Peterborough Regional Health Centre*	• Written (67)	Who<br>• Coordinators (Navigators) (P00C3)<br>Process<br>• Initial assessment of what patient is looking for (P00C3)	• Communication with patients and families<br>○ Initial assessment of what patient is looking for (P00C3)	• No preliminary assessment (P00C3)	• Not applicable<br>○ Ineligibility is not determined at referral (P00C3)	• Not applicable<br>○ No reporting is completed without an assessment
Ontario - *Mount Sinai Healthcare Facility, Toronto*	• Written (P00C36)	• Intake process not specified	• Not applicable	• No preliminary assessment (P00C36)	• Not applicable<br>○ Ineligibility is not determined at referral (P00C36)	• Not applicable<br>○ No reporting is completed without an assessment
Ontario - *University Health Network*	• Written (69) (P00C8)	Process<br>• Involves gathering information on the following (P00C8)<br>○ Diagnosis<br>○ Prognosis and medical history<br>○ Urgency<br>○ Preferred location<br>○ Most responsible provider notes<br>○ Isolation requirements<br>○ Communication needs<br>○ Privacy concerns	• Patient information and consent<br>○ Involves gathering information on the following (P00C8)<br>▪ Diagnosis<br>▪ Prognosis and medical History<br>▪ Urgency<br>▪ Preferred location<br>▪ Most responsible provider notes<br>▪ Isolation precautions<br>▪ Communication needs<br>▪ Privacy concerns	• No preliminary assessment completed (P00C8)	• Not eligible<br>○ Ineligibility is not determined at referral (P00C8)	• Not eligible<br>○ Ineligibility is not determined at referral (P00C8)
Ontario - *Grand River Hospital*	• Written (P00C50)<br>• Verbal (P00C50)	Process<br>• Intake process involves<br>○ Communicate with patients and families (P00C50)<br>○ Clarify legal requirements (P00C50)<br>○ Set expectations (P00C50)<br>○ Triage and screen patients (P00C50)	• Screening<br>○ Yes (P00C50)<br>• Communication with patients and families<br>○ Communicate with patients and families (P00C50)<br>○ Clarify legal requirements (P00C50)<br>○ Set expectations (P00C50)<br>• Triage and priority setting<br>○ Yes (P00C50)	• High level screening of patients against eligibility criteria (P00C50)	• Information not specified	• Nurse practitioner (Coordinator) (P00C50)
COMMUNITY OF PRACTICE						
Ontario - *Hamilton Family Health Team*	• Written (P00B18)	Who<br>• Coordinators (Navigators) (P00B18)<br>Process<br>• Preliminary screen (P00B18)<br>1. Collect Health Canada's voluntary sociodemographic information (P00B18, P00B32)	• Screening<br>○ Review cases to see that mental health is not sole reason for request. If it is, patient is informed that they are not eligible (P00B18)<br>• Patient information and consent	• No preliminary assessment (P00B18)	• Not applicable<br>○ Ineligibility is not determined at referral (P00B18)<br>• Coordinators discuss unclear cases of eligibility with medical lead, or they send cases to assessors (P00B18)	• Not applicable<br>○ No reporting is completed without an assessment<br>• Coordinators cannot determine eligibility (P00B18)

Jurisdiction	In what form are requests received?	What does the intake process involve?	What are the common elements of the intake process	What does the preliminary assessment involve?	What is done once a patient is found ineligible at the referral stage?	Who is responsible for reporting ineligible cases at the referral stage?
		2. Review cases to see that mental health is not sole reason for request. If it is, patient is informed that they are not eligible (P00B18)<br>3. Call eligible patients to collect information and consent to contact their primary care providers (P00B18)<br>4. Explore options to relieve suffering (P00B18)<br>5. Review assessment availability for patient (P00C38)<br>6. Contact physicians for assessment (P00B18)<br>7. Review cases of unclear eligibility (P00B18)	○ Call eligible patients to collect information and consent to contact their primary care providers (P00B18)<br>• Communication with patients and families<br>○ Call eligible patients to collect information and consent to contact their healthcare providers (P00B18)<br>• Coordination<br>○ Contact physicians for assessment (P00B18)<br>• Form review and documentation<br>○ Collect Health Canada's voluntary sociodemographic information (P00B18, P00B32)			
Ontario - *Niagara Community MAiD Team, St. Catharine's, Niagara*	• Written (P00C11)	Who<br>• Independent practitioners (P00C11)<br>Process<br>1. Contact patients or corresponding family members to arrange home visits upon receiving requests (P00C11)<br>2. Receive consent to access patients' electronic health records (P00C11)<br>○ A referral from provincial care coordinating services center automatically gives consent (P00C11)<br>3. Complete home visits in three parts<br>○ Introduce MAiD and provide information patients or families need (P00C11)<br>○ Introduce MAiD provision, including procedures and settings (P00C11)<br>○ If patient wishes to proceed, independent practitioners will start formal assessment process (P00C11)	• Patient information and consent<br>○ Receive consent to access patients' electronic health records (P00C11)<br>• Communication with patients and families<br>○ Introduce MAiD and provide information patients or families need (P00C11)<br>○ Introduce MAiD provision, including procedures and settings (P00C11)	• Referrals go directly to practitioners for assessment (P00C11)	• Not applicable<br>○ Ineligibility is not determined at referral (P00C11)	• Not applicable<br>○ No reporting is completed without an assessment (P00C11)
GEOGRAPHICAL AREAS						
Ontario - *Oakville & Mississauga area*	• Written (P00C18)<br>• Verbal (P00C18)	Who<br>• Independent practitioners (P00C18)<br>Process<br>1. Provide intake form to clients in their primary assessments (P00C18)?<br>2. Explain MAiD processes to patients (P00C18)	• Communication with patients and families<br>○ Primary assessors explain MAiD processes to patients (P00C18)<br>○<br>• Form review and documentation<br>○ Primary assessors provide intake form to clients in their primary assessments (P00C18)	• Referrals go directly to practitioners for assessment (P00C18)	• Not applicable<br>○ Ineligibility is not determined at referral (P00C18)	• Not applicable<br>○ No reporting is completed without an assessment (P00C18)
Ontario - *Oakville & Mississauga Area, Burlington area*	• Written (P00C24)	• Not applicable<br>○ No intake process (P00C24)	• Not applicable<br>○ No intake process (P00C24)	• Referrals go directly to practitioners for assessment (P00C21)	• Not applicable<br>○ Ineligibility is not determined at referral (P00C21)	• Not applicable<br>○ No reporting is completed without an assessment (P00C21)

Jurisdiction	In what form are requests received?	What does the intake process involve?	What are the common elements of the intake process	What does the preliminary assessment involve?	What is done once a patient is found ineligible at the referral stage?	Who is responsible for reporting ineligible cases at the referral stage?
Ontario - *Renfrew County*	• Written (P00C22)	Who<br>• Independent practitioners (P00C22)<br>Process<br>1. Ask patients about confidentiality requirements<br>○ E.g., If they want their family doctor to know about MAiD request or if they could be secondary assessor (P00C22)<br>2. Arrange assessments based on priority (P00C22)<br>3. Coordinate secondary assessors for referrals from Champlain MAiD network (P00C22)<br>○ If not, independent practitioners would assist in finding one (P00C22)	• Communication with patients and families<br>○ Ask patients about confidentiality requirements (P00C22)<br>• Coordination<br>○ Arrange assessments based on priority (P00C22)<br>• Triage and priority setting<br>○ Arrange assessments based on priority (P00C22)	• Referrals go directly to practitioners for assessment (P00C22)	• Not applicable<br>○ Ineligibility is not determined at referral (P00C22)	• Not applicable<br>○ No reporting is completed without an assessment (P00C22)
Ontario - *Greater Toronto Area*	• Written (P00C24)<br>• Verbal (P00C26)	• Not applicable<br>○ No intake process (P00C24)	• Not applicable<br>○ No intake process (P00C24)	• Referrals go directly to practitioners for assessment (P00C24)	• Not applicable<br>○ Ineligibility is not determined at referral (P00C24)	• Not applicable<br>○ No reporting is completed without an assessment (P00C24)
Ontario - *Waterloo area*	• Written (P00C25)	Who<br>• ON-HCCSS-Waterloo Coordinators (Navigators) (P00C25)<br>Process<br>• Identify and screen requests for information and requests for MAiD (P00C25)	• Screening<br>○ Identify and screen out requests information and requests for MAiD (P00C25)	• No preliminary assessment (P00C25)	• Coordinators connect patients to additional supports (P00C25)	• Not applicable<br>○ No reporting is completed without an assessment (P00C25)
Ontario - *Noelville, Sudbury, Elliot Lake, Sturgeon Falls area*	• Written (P00C26)<br>• Verbal (P00C26)	• Not applicable<br>○ No intake process specified (P00C26)	• Not applicable<br>○ No intake process specified (P00C26)	• Referrals go directly to practitioners for assessment (P00C26)	• Not applicable<br>○ Ineligibility is not determined at referral (P00C26)	• Not applicable<br>○ No reporting is completed without an assessment (P00C26)
Québec - *Ministry of Health and Social Services*	• Written (71)<br>• Verbal (71)	• Not applicable	• Not applicable	• No preliminary assessment (P00C37)	• Not applicable	• Not applicable<br>○ No reporting is completed without an assessment (P00C37)
INTEGRATED HEALTH AND SOCIAL SERVICES CENTRES						
Québec – *CISS Montérégie*	• Written (71) (P00C27)<br>• Verbal (71) (P00C27)	• No intake process specified	• Not applicable	• No preliminary assessment (P00C37)	• Not applicable<br>○ Ineligibility is not determined at referral (P00C27)	• Not applicable<br>○ No reporting is completed without an assessment (P00C27)
INTEGRATED UNIVERSITY HEALTH AND SOCIAL SERVICES CENTRES						
Québec – *CIUSS Capitale-Nationale*	• Written (71)<br>• Verbal (71)	• No intake process specified	• Not applicable	• No preliminary assessment (P00C37)	• Not applicable<br>○ Ineligibility is not determined at referral (P00C37)	• Not applicable<br>○ No reporting is completed without an assessment (P00C37)
Québec –*University of Montreal Hospital Center*	• Written (71)<br>• Verbal (71)	• No intake process specified	• Not applicable	• No preliminary assessment (P00C37)	• Not applicable<br>• Ineligibility is not determined at referral (P00C37)	• Not applicable<br>• No reporting is completed without an assessment (P00C37)
Québec – *McGill University Health Centre*	• Written (71) (P00C9)<br>• Verbal (71) (P00C9)	Who<br>• Coordinator (Advance practice nurse) (P00C9)<br>Triage Process<br>1. Connect with patients to better understand their cases (P00C9)	• Screening<br>○ Yes (P00C9)<br>• Communication with patients and families<br>○ Connect better understand their cases (P00C9)<br>• Coordination	• No preliminary assessment (P00C37)	• Coordinators connect patients to additional supports (P00C9)	• Not applicable<br>○ No reporting is completed without an assessment (P00C37)

Jurisdiction	In what form are requests received?	What does the intake process involve?	What are the common elements of the intake process	What does the preliminary assessment involve?	What is done once a patient is found ineligible at the referral stage?	Who is responsible for reporting ineligible cases at the referral stage?
		2. Review form with information about MAiD request<br>○ Review referral form from referring practitioner/clinic (P00C9)<br>3. Determine priority for cases (P00C9)<br>4. Determine necessity of additional supports (P00C9)<br>5. Coordinate assessments (P00C9)	○ Connect with practitioners to schedule patient's assessment (P00C9)<br>• Triage and priority setting<br>○ Determine priority for cases (P00C9)<br>• Form review and documentation<br>○ Review form with information about MAiD request (P00C9)<br>• Support services<br>○ Determine necessity of additional supports (P00C9)			

Table 7: Written request and consent for assessment table

Jurisdiction	Form Title	Patient information	Patient eligibility criteria	Informed consent	Declaration of independent witness	Where to submit completed form	Additional information
Alberta – *Alberta Health Service*	• Record of Request for MAiD (76)	• Name (76)<br>• Date of birth (76)<br>• Health care insurance number (76)<br>• Sex (76)<br>• Gender (76)<br>• Address (76)<br>• Phone number (76)	• Age<br>○ ≥ 18 (76)<br>• Patient explicit request for MAiD (76)<br>• Patient is eligible to receive healthcare services in Canada (76)<br>• Patient MAiD request is voluntary (76)<br>• Patient reserves right to withdraw MAiD request (76)<br>• Patient has been informed by practitioners about medical condition (76)<br>○ Understands severity of medical condition (76)<br>○ In an advanced state of irreversible decline in capability (76)<br>○ Illness, disease or disability or state of decline causes enduring intolerable physical or psychological suffering (76)<br>• Patient understands the timeline for eligibility (76)<br>• Consent to share information with the Federal Minister of Health (76)<br>• Patient understands outcome once MAiD medication is administered (76)	• No consent statement	• Age<br>○ ≥ 18 (76)<br>• Understand nature of MAiD request (76)<br>• Present during signing of request form (76)<br>• Does not know or believe to be a financial or material beneficiary from patient's death (76)<br>• Does not own or operate a healthcare facility providing care or residential facility to patient (76)<br>• Not involved in assessment of patient eligibility for MAiD or provision (76)	• AHS care coordination service (76)<br>• Physician/nurse practitioner (76)	• Indigenous Identity (76)<br>○ First Nations (76)<br>○ Métis (76)<br>○ Inuk/Inuit (76)<br>• Racial, ethnic or cultural identity (76)<br>• Disability (76)<br>• Usual place of residence (76)
British Columbia – *Ministry of Health*	• Request for MAiD (92)	• Name (92)<br>• Date of birth (92)<br>• Health care insurance number (92)<br>• Sex (92)<br>• Gender (92)<br>• Address (92)<br>• Phone number (92)<br>• Diagnosis (92)<br>• Primary healthcare provider (92)<br>• Contact person for MAiD request (92)	• Patient is eligible to receive health services in Canada (92)<br>• Patient MAiD request is voluntary (92)<br>• Patient has been informed by a practitioner about medical condition (92)<br>○ Understands severity of medical condition (92)<br>○ Medical condition is serious and cannot be relieved by any means acceptable to patient (92)<br>• Patient consents to share information with other health professionals (92)<br>• Patient reserves right to withdraw MAiD request (92)<br>• Patient accepts responsibility to seek advice on life insurance policy (92)	• No consent statement	• Age<br>○ ≥ 18 (92)<br>• Understands nature of MAiD request (92)<br>• Present during signing of request form (92)<br>• Does not know or believe to be a financial or material beneficiary from patient's death (92)<br>• Does not own or operate a healthcare facility or facility providing care or residence to patient (92)<br>• Provide paid health care services or personal care services to patient as a primary occupation (92) and<br>○ Not involved in assessment of patient eligibility for MAiD or provision (92) OR<br>○ Does not provide healthcare services or personal care services directly to patient (92)<br>• Knows patient or has proof of identity (92)	• Regional Health MAiD program (92)<br>• Physician/nurse practitioner (92)	• Indigenous Identity (92)<br>○ First Nations (92)<br>○ Métis (92)<br>○ Inuit (92)<br>• Racial, ethnic or cultural identity (92)<br>• Disability (92)<br>• Usual place of residence (92)

Jurisdiction	Form Title	Patient information	Patient eligibility criteria	Informed consent	Declaration of independent witness	Where to submit completed form	Additional information
British Columbia – *Fraser Health*	• Request for MAiD (92)	• Name (92)<br>• Date of birth (92)<br>• Health care insurance number (92)<br>• Sex (92)<br>• Gender (92)<br>• Address (92)<br>• Phone number (92)<br>• Diagnosis (92)<br>• Primary Health Care Provider (92)<br>• Contact person for MAiD request (92)	• Patient reserves right to withdraw MAiD request (92)<br>• Patient is eligible to receive health services in Canada (92)<br>• Patient MAiD request is voluntary (92)<br>• Patient has been informed by practitioners about medical condition (92)<br>○ Understands severity of medical condition (92)<br>○ Medical condition is serious and cannot be relieved by any means acceptable to patient (92)<br>• Patient consents to share information with other health professionals (92)<br>• Patient accepts responsibility to seek advice on life insurance policy (92)	• No consent statement	• Age<br>○ ≥ 18 (92)<br>• Understands nature of MAiD request (92)<br>• Present during signing of request form (92)<br>• Does not know or believe to be a financial or material beneficiary from patient's death (92)<br>• Does not own or operate a healthcare facility or facility providing care or residence to patient (92)<br>• Provide paid health care services or personal care services to patient as a primary occupation (92) and<br>○ Not involved in assessment of patient eligibility for MAiD or provision (92) OR<br>○ Does not provide healthcare services or personal care services directly to patient (92)<br>• Knows patient or has proof of identity (92)	• Regional Health MAiD program (92)<br>• Physician/nurse practitioner (92)	• Indigenous Identity (92)<br>○ First Nations (92)<br>○ Métis (92)<br>○ Inuit (92)<br>• Racial, ethnic or cultural identity (92)<br>• Disability (92)<br>• Usual place of residence (92)
British Columbia – *Interior Health*	• Request for MAiD (92)	• Name (92)<br>• Date of birth (92)<br>• Health care insurance number (92)<br>• Sex (92)<br>• Gender (92)<br>• Address (92)<br>• Phone number (92)<br>• Diagnosis (92)<br>• Primary Health Care Provider (92)<br>• Contact person for MAiD request (92)	• Patient reserves right to withdraw MAiD request (92)<br>• Patient is eligible to receive health services in Canada (92)<br>• Patient MAiD request is voluntary (92)<br>• Patient has been informed by practitioners about medical condition (92)<br>○ Understands severity of medical condition (92)<br>○ Medical condition is serious and cannot be relieved by any means acceptable to patient (92)<br>• Patient consents to share information with other health professionals (92)<br>• Patient accepts responsibility to seek advice on life insurance policy (92)	• No consent statement	• Age<br>○ ≥ 18 (92)<br>• Understands nature of MAiD request (92)<br>• Present during signing of request form (92)<br>• Does not know or believe to be a financial or material beneficiary from patient's death (92)<br>• Does not own or operate a healthcare facility or facility providing care or residence to patient (92)<br>• Provide paid health care services or personal care services to patient as a primary occupation (92) and<br>○ Not involved in assessment of patient eligibility for MAiD or provision (92) OR<br>○ Does not provide healthcare services or personal care services directly to patient (92)<br>• Knows patient or has proof of identity (92)	• Regional Health MAiD program (92)<br>• Physician/nurse practitioner (92)	• Indigenous Identity (92)<br>○ First Nations (92)<br>○ Métis (92)<br>○ Inuit (92)<br>• Racial, ethnic or cultural identity (92)<br>• Disability (92)<br>• Usual place of residence (92)
British Columbia – *Island Health*	• Request for MAiD (92)	• Name (92)<br>• Date of birth (92)	• Patient reserves right to withdraw MAiD request (92)	• No consent statement	• Age<br>○ ≥ 18 (92)	• Regional Health MAiD program (92)	• Indigenous Identity (92)<br>○ First Nations (92)<br>○ Métis (92)

Jurisdiction	Form Title	Patient information	Patient eligibility criteria	Informed consent	Declaration of independent witness	Where to submit completed form	Additional information
		• Health care insurance number (92)<br>• Sex (92)<br>• Gender (92)<br>• Address (92)<br>• Phone number (92)<br>• Diagnosis (92)<br>• Primary Health Care Provider (92)<br>• Contact person for MAiD request (92)	• Patient is eligible to receive health services in Canada (92)<br>• Patient MAiD request is voluntary (92)<br>• Patient has been informed by practitioners about medical condition (92)<br>○ Understands severity of medical condition (92)<br>○ Medical condition is serious and cannot be relieved by any means acceptable to patient (92)<br>• Patient consents to share information with other health professionals (92)<br>• Patient accepts responsibility to seek advice on life insurance policy (92)		• Understands nature of MAiD request (92)<br>• Present during signing of request form (92)<br>• Does not know or believe to be a financial or material beneficiary from patient's death (92)<br>• Does not own or operate a healthcare facility or facility providing care or residence to patient (92)<br>• Provide paid health care services or personal care services to patient as a primary occupation (92) and<br>○ Not involved in assessment of patient eligibility for MAiD or provision (92) OR<br>○ Does not provide healthcare services or personal care services directly to patient (92)<br>• Knows patient or has proof of identity (92)	• Physician/nurse practitioner (92)	○ Inuit (92)<br>• Racial, ethnic or cultural identity (92)<br>• Disability (92)<br>• Usual place of residence (92)
British Columbia – *Northern Health*	• Request for MAiD (92)	• Name (92)<br>• Date of birth (92)<br>• Health care insurance number (92)<br>• Sex (92)<br>• Gender (92)<br>• Address (92)<br>• Phone number (92)<br>• Diagnosis (92)<br>• Primary Health Care Provider (92)<br>• Contact person for MAiD request (92)	• Patient reserves right to withdraw MAiD request (92)<br>• Patient is eligible to receive health services in Canada (92)<br>• Patient MAiD request is voluntary (92)<br>• Patient has been informed by practitioners about medical condition (92)<br>○ Understands severity of medical condition (92)<br>○ Medical condition is serious and cannot be relieved by any means acceptable to patient (92)<br>• Patient consents to share information with other health professionals (92)<br>• Patient accepts responsibility to seek advice on life insurance policy (92)	• No consent statement	• Age<br>○ ≥ 18 (92)<br>• Understands nature of MAiD request (92)<br>• Present during signing of request form (92)<br>• Does not know or believe to be a financial or material beneficiary from patient's death (92)<br>• Does not own or operate a healthcare facility or facility providing care or residence to patient (92)<br>• Provide paid health care services or personal care services to patient as a primary occupation (92) and<br>○ Not involved in assessment of patient eligibility for MAiD or provision (92) OR<br>○ Does not provide healthcare services or personal care services directly to patient (92)<br>• Knows patient or has proof of identity (92)	• Regional Health MAiD program (92)<br>• Physician/nurse practitioner (92)	• Indigenous Identity (92)<br>○ First Nations (92)<br>○ Métis (92)<br>○ Inuit (92)<br>• Racial, ethnic or cultural identity (92)<br>• Disability (92)<br>• Usual place of residence (92)
British Columbia – *Vancouver Coastal Health*	• Request for MAiD (92)	• Name (92)<br>• Date of birth (92)<br>• Health care insurance number (92)	• Patient reserves right to withdraw MAiD request (92)<br>• Patient is eligible to receive health services in Canada (92)	• No consent statement	• Age<br>○ ≥ 18 (92)<br>• Understands nature of MAiD request (92)	• Regional Health MAiD program (92)<br>• Physician/nurse practitioner (92)	• Indigenous Identity (92)<br>○ First Nations (92)<br>○ Métis (92)<br>○ Inuit (92)<br>• Racial, ethnic or cultural identity (92)

Jurisdiction	Form Title	Patient information	Patient eligibility criteria	Informed consent	Declaration of independent witness	Where to submit completed form	Additional information
		• Sex (92)<br>• Gender (92)<br>• Address (92)<br>• Phone number (92)<br>• Diagnosis (92)<br>• Primary Health Care Provider (92)<br>• Contact person for MAiD request (92)	• Patient MAiD request is voluntary (92)<br>• Patient has been informed by practitioners about medical condition (92)<br>○ Understands severity of medical condition (92)<br>○ Medical condition is serious and cannot be relieved by any means acceptable to patient (92)<br>• Patient consents to share information with other health professionals (92)<br>• Patient accepts responsibility to seek advice on life insurance policy (92)		• Present during signing of request form (92)<br>• Does not know or believe to be a financial or material beneficiary from patient's death (92)<br>• Does not own or operate a healthcare facility or facility providing care or residence to patient (92)<br>• Provide paid health care services or personal care services to patient as a primary occupation (92) and<br>○ Not involved in assessment of patient eligibility for MAiD or provision (92) OR<br>○ Does not provide services or personal care services directly to patient (92)<br>• Knows patient or has proof of identity (92)		• Disability (92)<br>• Usual place of residence (92)
Manitoba – *Shared Health*	• Written Request Form (93)	• Name (93)<br>• Date of birth (93)<br>• Health care insurance number (93)<br>• Gender (93)<br>• Address (93)<br>• Diagnosis (93)	• Patient reserves right to withdraw request (93)<br>• Patient MAiD request is voluntary (93)<br>• Patient has been informed by a practitioner about medical condition (93)<br>○ Understands the severity of medical condition (93)<br>○ In an advanced state of irreversible decline (93)<br>• Patient understands outcome after MAiD medication is administered (93)<br>• Patient consents to share information with other health professionals involved with care (93)	• No consent statement	• Age<br>○ ≥ 18 (93)<br>• Understands nature of request for MAiD (93)<br>• Present during signing of request form (93)<br>• Does not know or believe to be a financial or material beneficiary from patient's death (93)<br>• Does not own or operate a healthcare facility or facility providing care or residence to patient (93)<br>• Provide paid health care services or personal care services to patient as a primary occupation (93)<br>○ Not involved in assessment of patient eligibility for MAiD or provision (93)<br>• Knows patient or provided with proof of identity (93)	• Provincial MAiD program (93)	• No additional information requested
New Brunswick – *Horizon Health Network*	• Patient Request form (94)	• Name (94)<br>• Date of birth (94)<br>• Health care insurance number (94)<br>• Diagnosis (94)<br>• Primary care practitioner (94)<br>• Phone number (94)<br>• Medicare number (94)	• Patient MAiD request is voluntary (94)<br>• Patient understands process to determine eligibility to receive MAiD (94)<br>• Patient consents to medical records being reviewed by healthcare team(94)<br>• Patient reserves right to withdraw MAiD request (94)out of order	• No consent statement	• Age<br>○ ≥ 18 (94)<br>• Understands nature of MAiD request (94)<br>• Present during signing of request form (94)<br>• Does not know or believe to be a financial or material beneficiary from patient's death (94)	• Regional MAiD program (94)	• No additional information requested

Jurisdiction	Form Title	Patient information	Patient eligibility criteria	Informed consent	Declaration of independent witness	Where to submit completed form	Additional information
			• Patient understands outcome after MAiD medication is administered<br>○ self or intravenous (94)<br>• Patient consents to information used for monitoring MAiD process (94)		• Does not own or operate a healthcare facility or facility providing care or residence to patient (94)<br>• Not involved in assessment of patient eligibility for MAiD or provision (94)<br>• Declares patient MAiD request is voluntary (94)		
New Brunswick – *Vitalité Health* Network	• Patient Request (83)	• Name (83)<br>• Date of birth (83)<br>• Health care insurance number (83)<br>• Family physician/nurse practitioner (83)<br>• Phone number (83)<br>• Diagnosis (83)<br>• Medicare number (83)	• Patient MAiD request is voluntary (83)<br>• Medical condition is serious and cannot be relieved by any means acceptable to patient (83)<br>• Patient understands process to determine eligibility to receive MAiD (83)<br>• Patient understands process of administering MAiD medications<br>○ Intravenous (83)<br>○ Self-administration (83)<br>• Patient consents to medical records being reviewed by healthcare team(83)<br>• Patient reserves right to withdraw MAiD request (83)<br>• Patient consents to use information for monitoring MAiD process (83)<br>• Patient understands the timeline for eligibility<br>• (83)<br>• Patients understand location for MAiD administration (83)<br>○ Patient's place of residence<br>○ Vitalité Health Network designated facility (83)	• No consent statement	• Age<br>○ ≥ 18 (83)<br>• Understands nature of MAiD request (83)<br>• Present during signing of request form (83)<br>• Does not know or believe to be a financial or material beneficiary from patient's death (83)<br>• Provide paid health care services or personal care to patient (83)<br>○ Not involved in assessment of patient eligibility for MAiD or provision (83)<br>• Declares patient MAiD request is voluntary (83)	• Physician/nurse practitioner (83)	• No additional information requested
Newfoundland and Labrador – *Eastern Zone*	• Patient Request Record (95)	• Name (95)<br>• Date of birth (95)<br>• Health care insurance number (95)<br>• Gender (95)<br>• Address (95)<br>• Diagnosis (95)	• Age<br>○ ≥ 18 (95)<br>• Patient explicitly request for MAiD (95)<br>• Patient MAiD request is voluntary (95)<br>• Patient has been informed about medical condition (95)<br>○ Understands the severity of medical condition (95)<br>○ In an advanced state of irreversible decline in capability (95)<br>○ Illness, disease or disability or state of decline causes enduring intolerable physical or psychological suffering (95)	• Consent statement (95)<br>○ "*I consent to be assessed for eligibility and capability by one or more colleagues of my physician or nurse practitioner and, if I am eligible, that a pharmacist and other staff will be contacted to aid in addressing my request*" (95)	• Age<br>○ ≥ 18 (95)<br>• Understands nature of request for MAiD (95)<br>• Present during signing of request form (95)<br>• Does not know or believe to be a financial or material beneficiary from patient's death (95)<br>• Does not own or operate a healthcare facility or facility providing care or residence to patient (95)<br>• Not directly involved in providing health care services or personal care to patients (95) OR	• Regional Health MAiD office (95)	No additional information requested

Jurisdiction	Form Title	Patient information	Patient eligibility criteria	Informed consent	Declaration of independent witness	Where to submit completed form	Additional information
			○ Medical condition is serious and cannot be relieved by any means acceptable to patient<br>• Patient has received information about available treatment options (95)<br>• Patient assents to receive MAiD if eligible (95)<br>• Patient has received additional information to requests (95)<br>• Patient reserves right to withdraw request (95)<br>• Patient understands the process of medication administration is intravenous (95)		• Provide paid health care services or personal care to patient as a primary occupation (95)<br>○ Not involved in assessment of patient eligibility for MAiD or provision (95)<br>• Knows patient or provided with proof of identity (95)		
Newfoundland and Labrador – *Western Zone*	• Patient Request Record (95)	• Name (95)<br>• Date of birth (95)<br>• Health care insurance number (95)<br>• Gender (95)<br>• Address (95)<br>• Diagnosis (95)	• Age<br>○ ≥ 18 (95)<br>• Patient explicitly request for MAiD (95)<br>• Patient MAiD request is voluntary (95)<br>• Patient has been informed about medical condition (95)<br>○ Understands the severity of medical condition (95)<br>○ In an advanced state of irreversible decline in capability (95)<br>○ Illness, disease or disability or state of decline causes enduring intolerable physical or psychological suffering (95)<br>○ Medical condition is serious and cannot be relieved by any means acceptable to patient<br>• Patient has received information about available treatment options (95)<br>• Patient assents to receive MAiD if eligible (95)<br>• Patient has received additional information to requests (95)<br>• Patient reserves right to withdraw request (95)<br>• Patient understands the process of medication administration is intravenous (95)	• Consent statement (95)<br>○ *"I consent to be assessed for eligibility and capability by one or more colleagues of my physician or nurse practitioner and, if I am eligible, that a pharmacist and other staff will be contacted to aid in addressing my request*" (95)	• Age<br>○ ≥ 18 (95)<br>• Understands nature of request for MAiD (95)<br>• Present during signing of request form (95)<br>• Does not know or believe to be a financial or material beneficiary from patient's death (95)<br>• Does not own or operate a healthcare facility or facility providing care or residence to patient (95)<br>• Not directly involved in providing health care services or personal care to patients (95) OR<br>• Provide paid health care services or personal care to patient as a primary occupation (95)<br>○ Not involved in assessment of patient eligibility for MAiD or provision (95)<br>• Knows patient or provided with proof of identity (95)	• Regional Health MAiD office (95)	No additional information requested
Newfoundland and Labrador – *Central Zone*	• Patient Request Record (95)	• Name (95)<br>• Date of birth (95)<br>• Health care insurance number (95)<br>• Gender (95)<br>• Address (95)<br>• Diagnosis (95)	• Age<br>○ ≥ 18 (95)<br>• Patient explicitly request for MAiD (95)<br>• Patient MAiD request is voluntary (95)<br>• Patient has been informed about medical condition (95)<br>○ Understands the severity of medical condition (95)	• Consent statement (95)<br>○ *"I consent to be assessed for eligibility and capability by one or more colleagues of my physician or nurse practitioner and, if I am eligible, that a pharmacist and other staff will be contacted to aid in*	• Age<br>○ ≥ 18 (95)<br>• Understands nature of request for MAiD (95)<br>• Present during signing of request form (95)	• Health information and management department (95)	No additional information requested

Jurisdiction	Form Title	Patient information	Patient eligibility criteria	Informed consent	Declaration of independent witness	Where to submit completed form	Additional information
			○ In an advanced state of irreversible decline in capability (95)<br>○ Illness, disease or disability or state of decline causes enduring intolerable physical or psychological suffering (95)<br>○ Medical condition is serious and cannot be relieved by any means acceptable to patient<br>• Patient has received information about available treatment options (95)<br>• Patient assents to receive MAiD if eligible (95)<br>• Patient has received additional information to requests (95)<br>• Patient reserves right to withdraw request (95)<br>• Patient understands the process of medication administration is intravenous (95)	*addressing my request*" (95)	• Does not know or believe to be a financial or material beneficiary from patient's death (95)<br>• Does not own or operate a healthcare facility or facility providing care or residence to patient (95)<br>• Not directly involved in providing health care services or personal care to patients (95) OR<br>• Provide paid health care services or personal care to patient as a primary occupation (95)<br>○ Not involved in assessment of patient eligibility for MAiD or provision (95)<br>• Knows patient or provided with proof of identity (95)		
Northwest Territories	• Formal Written Request (85)<br>○ Completed after patients has been informed by a practitioner about medical condition (85)	• Name (85)<br>• Date of birth (85)<br>• Health care insurance number (85)<br>• Sex (85)<br>• Usual place of Residence (85)<br>• Postal code (85)	• Patient is eligible to receive health services in Canada (85)<br>• Patient explicitly request for MAiD (85)<br>• Patient MAiD request is voluntary (85)<br>• Patient has been informed by a practitioner about medical condition (85)<br>○ Understands the severity of medical condition (85)<br>○ In an advanced state of irreversible decline in capability (85)<br>○ Illness, disease or disability or state of decline causes enduring intolerable physical or psychological suffering (85)<br>○ Medical condition is serious and cannot be relieved by any means acceptable to patient (85)<br>• Patient is capable of making healthcare decision (85)	• Consent statement (85)<br>○ *"This request is my informed consent to receive MAiD. I understand that can withdraw this request at any time and in any manner"* (85)	• Age<br>○ ≥ 18 (85)<br>• Understands nature of MAiD request (85)<br>• Does not know or believe to be a financial or material beneficiary from patient's death (85)<br>• Does not own or operate a healthcare facility or facility providing care or residence to patient (85)<br>• Not directly involved in providing health care services and personal care to patient (85) OR<br>• Provides health care services or personal care to patient as a primary occupation (85)<br>○ Not involved in assessment of patient eligibility for MAiD or provision (85)	• Territorial MAiD Program (85)<br>○ MAiD review committee (85)<br>• Patient's medical record (85)<br>○ Included on patient's medical record (85)	• Indigenous Identity (85)<br>○ First Nations (85)<br>○ Métis (85)<br>○ Inuit (85)<br>• Racial, ethnic or cultural identity (85)<br>• Disability (85)<br>• Gender pronouns (85)<br>• Gender identity (85)
Nova Scotia – *Nova Scotia Health*	• Patient Request and Informed Consent Form (96, 97)<br>○ Reasonably foreseeable natural death (96)<br>○ Reasonably non-foreseeable natural deaths (97)	• Name (96, 97)<br>• Date of birth (96, 97)<br>• Health care insurance number (96, 97)<br>• MAiD case number (96, 97)	• Patient MAiD request is voluntary (96, 97)<br>• Patient understands process to determine eligibility to receive MAiD (96, 97)<br>• Patient understands outcome after MAiD medication is administered (96, 97)<br>• Patient consents to sharing information with other healthcare professional involved in care (96, 97)	• Consent statement (completed after first assessment) (96, 97)<br>○ *"I believe I am fully informed with respect to the medical condition which has led me to request MAiD, including its nature, expected outcome, treatment options available, and potential*	• Age<br>○ ≥ 18 (96, 97)<br>• Understands nature of MAiD request (96, 97)<br>• Present during signing of request form (96, 97)<br>• Does not know or believe to be a financial or material beneficiary from patient's death (96, 97)	• Provincial MAiD program (96, 97)	• No additional information requested

Jurisdiction	Form Title	Patient information	Patient eligibility criteria	Informed consent	Declaration of independent witness	Where to submit completed form	Additional information
			• Patient reserves right to withdraw MAiD request<br>• Patient consents to use information for monitoring MAiD process (96, 97)	*complications*<br>○ *My medical condition causes me enduring suffering that is intolerable to me, which cannot be relieved by any treatment that I consider acceptable*<br>○ *I have been informed by a medical doctor/nurse practitioner about the options available to help relieve my suffering, including palliative care*<br>○ *I understand that the purpose and goal of MAiD is to bring about my death, and the risks of proceeding with MAiD have been explained to me*<br>○ *I am aware that I will be asked to provide consent again immediately before MAiD is provided unless alternative arrangements have been made*<br>○ *After providing my final consent to have MAiD, I authorize my MAiD Provider to administer intravenous medications that will bring about my death*<br>○ *My questions have been answered in a way that was understandable to me*<br>○ *I agree to the involvement of pharmacist(s), nurse(s), assistants or associates or other health professionals as may be appropriate for the purpose of MAiD, including a community nurse who may be asked to insert intravenous lines*" (96, 97)	• Does not own or operate a healthcare facility or facility providing care or residence to patient (96, 97)<br>• Not involved in assessment of patient eligibility for MAiD or provision (96, 97)<br>• Knows patient or provided with proof of identity (97)		
Prince Edward Island – *Health PEI*	• MAiD Patient Request and Consent Form (87)	• Name (87)<br>• Date of birth (87)<br>• Health care insurance number (87)<br>• Address (87)<br>• Phone (87)<br>• Diagnosis (87)<br>• Contact person for MAiD	• Patient is eligible to receive health services in Canada (87)<br>• Patient explicitly request for MAiD (87)<br>• Patient MAiD request is voluntary (87)<br>• Patient has been informed by a practitioner about medical condition (87)<br>○ Understands severity of medical	• No consent statement	• Age<br>○ ≥ 18 (87)<br>• Understands nature of MAiD request (87)<br>• Present during signing of request form (87)	• Physician/nurse practitioner (87)<br>• Provincial MAiD program (87)	• Indigenous Identity (87)<br>○ First Nations (87)<br>○ Métis (87)<br>○ Inuit (87)<br>• Racial, ethnic or cultural identity (87)<br>• Disability (87)<br>• Usual place of residence (87)

Jurisdiction	Form Title	Patient information	Patient eligibility criteria	Informed consent	Declaration of independent witness	Where to submit completed form	Additional information
		request (87)	condition (87)<br>• Patient consents to sharing information with other healthcare professional involved in care (87)<br>• Patient reserves right to withdraw MAiD request or ask questions (87)		• Does not know or believe to be a financial or material beneficiary from patient's death (87)<br>• Does not own or operate a healthcare facility or facility providing care or residence to patient (87)<br>• Does not provide health care services or personal care services to patient (87) OR<br>• Provides paid health care services or personal care services to patient as a primary occupation (87)<br>○ Not involved in assessment of patient eligibility for MAiD or provision (87)<br>• Knows patient or provided with proof of identity (87)		
Saskatchewan – *Saskatchewan Health Authority*	• Patient Written Request (98)	• Name (98)<br>• Date of birth (98)<br>• Health care insurance number (98)<br>• Phone number (98)<br>• Postal code (98)	• Age<br>○ ≥ 18 (98)<br>• Patient MAiD request is voluntary (98)<br>• Patient has received information about medical condition (98)<br>○ With available options to relieve suffering (98)<br>• Patient has been informed by a practitioner about medical condition (98)<br>○ Understands severity of medical condition (98)<br>○ In an advanced state of irreversible decline in capability (98)<br>• Patient understands process to determine eligibility to receive MAiD (98)<br>• Patient reserves right to withdraw MAiD request (98)<br>• Patient understands outcome after MAiD medication is administered (98)	• No consent statement	• Age<br>○ ≥ 18 (98)<br>• Understands nature of MAiD request (98)<br>• Present during signing of request form (98)<br>• Does not know or believe to be a financial or material beneficiary from patient's death (98)<br>• Does not own or operate a healthcare facility or facility providing care or residence to patient (98)<br>• Not an unpaid caregiver to patient (98)<br>• Knows patient or provided with proof of identity (98)	• Provincial MAiD program (98)	• No additional information requested
Yukon	• Patient request for Medical Assistance in Dying (89)	• Name (89)<br>• Date of birth (89)<br>• Health care insurance number (89)<br>• Diagnosis (89)	• Patient has been informed about medical condition (89)<br>○ Understands severity of medical condition (89)<br>○ In an advanced state of irreversible decline in capability (89)<br>○ Illness, disease or disability or state of decline causes enduring intolerable physical or psychological suffering (89)	• No consent statement	• Age<br>○ ≥ 18 (89)<br>• Understands nature of MAiD request (89)<br>• Present during signing of request form (89)<br>• Does not know or believe to be a financial or material beneficiary from patient's death (89)<br>• Does not own or operate a healthcare facility or facility	• Physician/nurse practitioner (89)	• No additional information requested

Jurisdiction	Form Title	Patient information	Patient eligibility criteria	Informed consent	Declaration of independent witness	Where to submit completed form	Additional information
					providing care or residence to patient (89)<br>• Not directly involved in providing unpaid health or personal care services to patient (89)<br>• Not involved in assessment of patient eligibility for MAiD or provision (89)		
Ontario – *Ministry of Health*	• Patient Request for MAiD (99)	• Name (99)<br>• Gender (99)<br>• Date of birth (99)<br>• Health care insurance number (99)<br>• Postal Code (99)	• Age<br>○ ≥ 18 (99)<br>• Patient is eligible to receive health services in Canada (99)<br>• Patient explicitly request for MAiD (99)<br>• Patient MAiD request is voluntary (99)<br>• Patient has been informed by a practitioner about medical condition (99)<br>○ Understands severity of medical condition (99)<br>○ In an advanced state of irreversible decline in capability (99)<br>○ Illness, disease or disability or state of decline causes enduring intolerable physical or psychological suffering (99)	• Consent statement (99)<br>○ *"I am giving my informed consent to receive MAiD and have been informed of the means that are available to me to relieve my suffering, including palliative care"* (99)	• Age<br>○ ≥ 18 (99)<br>• Understands nature of MAiD request (99)<br>• Present during signing of request form (99)<br>• Does not know or believe to be a financial or material beneficiary from patient's death (99)<br>• Does not own or operate a healthcare facility or facility providing care or residence to patient (99)<br>• Provide paid health care services or personal care services to patient<br>○ Not involved in assessment of patient eligibility for MAiD or provision (99)	• Physician/nurse practitioner (99)	• No additional information requested
Québec - *Ministry of Health and Social Services*	• Request for Medical Aid in Dying (100)	• Name (100)<br>• Date of birth (100)<br>• Address (100)<br>• Phone number (100)<br>• Health care insurance number (100)<br>• Postal code (100)	• Patient explicitly request for MAiD (100)<br>• Patient has been informed about medical conditions (100)<br>• Patient request a physician/nurse practitioner to administer MAiD (100)<br>• Patient authorize pharmacist to provide medication (100)	• No consent statement (100)	• Age<br>○ ≥ 18 (100)<br>• Understand nature of MAiD request<br>• Present during signing of request form (100)<br>• Does not know or believe to be a financial or material beneficiary from patient's death (100)<br>• Does not own or operate a healthcare facility or facility providing care or residence to patient (100)<br>• Provide paid health care services or personal care services to patients<br>○ Not involved in assessment of patient eligibility for MAiD (100)	• Physician/nurse practitioner (100)	• No additional information requested

Table 8: Translation and interpretive services

Jurisdiction	Are translation services available?	Who provides translation services?	What communication methods and/or interpretive services are available for non-verbal patients?	What is Translator/Speech Language Pathologist training requirements?
Alberta - *Alberta Health Services*	• Yes (P00A5)	Formal<br>• Provincial translation services<br>○ Alberta Health Services translators (P00A5)<br>○ Language Line (P00B9, P00A5, P00C6, P00B13)<br>Informal<br>• Bilingual assessor (P00B9, P00A5, P00C6, P00B13)<br>• Family/Friends (P00B9, P00A5, P00C6, P00B13)	• Closed-ended questions (P00C6)<br>• Speech language pathologists (P00C39)<br>• Assistive devices (P00B13)<br>○ Communication board (P00B13)<br>○ Tablet/computer (P00B13)	• Professional
British Columbia - *Ministry of Health*	• Yes (P00A9)	Formal<br>• Provincial translation services (P00A9)	• Information on communication methods/interpretive services not specified	• Professional
British Columbia - *Fraser Health*	• Yes (P00C17)	Formal<br>• Regional translation services (P00C17)	• Speech language pathologists (P00A10)	• Professional<br>• MAiD specific (P00A10)
British Columbia - *Interior Health*	• Yes (P00B14)	Formal<br>• Regional translation services (P00B14)<br>○ Phone (P00C29)	• Information on communication methods/interpretive services not specified	• Professional
British Columbia - *Island Health*	• Yes (P00A9)	Formal<br>• Provincial translation services (P00A9)	• Information on communication methods/interpretive services not specified	• Professional
British Columbia - *Northern Health*	• Information on translation services not specified	• Information on translation services not specified	• Information on communication methods/interpretive services not specified	• Information on training requirement not specified
British Columbia - *Vancouver Coastal Health*	• Yes (P00B10, P00B12, P00B16)	Formal<br>• Provincial translation services (P00B10, P00B12, P00B16)<br>○ In-person (P00B15)<br>○ Phone (during provision) (P00B12)	• Speech language pathologists (P00B10)	• Professional
British Columbia - *Provincial Health Services Authority*	• Information on translation services not specified	• Information on translation services not specified	• Information on communication methods/interpretive services not specified	• Information on training requirement not specified
Manitoba - *Shared Health*	• Yes (P00C34, P00B19)	Formal<br>• Provincial translation services (P00C34)<br>○ Phone (P00C34, P00B19)<br>Informal<br>• Translating through the family is avoided where possible (P00C34)	• Speech language pathologists (P00B19)	• Professional<br>• Contracted Speech language pathologist has specialized training on navigating legal procedures (P00B19)
New Brunswick - *Horizon Health Network*	• Yes (P00C15)	Formal<br>• Regional translation service<br>○ Phone (P00C15)	• Speech language pathologists (P00C15)	• Professional
New Brunswick - *Vitalité Health Network*	• Yes (101)	Formal<br>• Regional translation service<br>○ Phone (101)	• Information on communication methods/interpretive services not specified	• Professional
Newfoundland and Labrador - *Department of Health and Community Services*	• Information on translation services not specified	• Information on translation services not specified	• Speech language pathologists (10)	• Professional
Newfoundland and Labrador - *Eastern Zone*	• Information on translation services not specified	• Information on translation services not specified	• Speech language pathologists (10)	• Professional
Newfoundland and Labrador - *Western Zone*	• Yes (P00C44)	Formal<br>• Translation services<br>○ Phone (P00C44)	• Speech language pathologists (P00C44)	• Professional
Newfoundland and Labrador - *Central Zone*	• Information on translation services not specified	• Information on translation services not specified	• Speech language pathologists (10)	• Professional
Northwest Territories	• Yes (P00A3)	Formal<br>• CANTalk (P00A3)<br>• Stanton Indigenous wellness program (P00A3)<br>• Office of the client experience (P00A3)	• Familiarization with patient provided communication methods (P00A3)	• Professional
Nova Scotia - *Nova Scotia Health*	• Yes (P00A12)	Formal<br>• Provincial translation services<br>○ Virtual (P00A12)	• Written communication (P00A12)	• Professional
Prince Edward Island - *Health PEI*	• Yes (B00B17)	Formal	• Speech language pathologists (P00B8)	• Professional

Jurisdiction	Are translation services available?	Who provides translation services?	What communication methods and/or interpretive services are available for non-verbal patients?	What is Translator/Speech Language Pathologist training requirements?
		• Provincial translation services (P00B17)	• Language board (P00B8)	
Saskatchewan - *Saskatchewan Health Authority*	• Yes (P00C1, P00B1, P00A1)	Formal<br>• Provincial translation services (P00C1, P00B1)<br>Informal<br>• Indigenous language app (P00A1)	• Speech language pathologists (P00C1, P00B1)	• Professional
Yukon - *Department of Health and Social Services*	• Yes (P00C7)	Formal<br>• Territorial translation services (P00C7)<br>Informal<br>• Family (P00C1)	• Familiarization with patient provided communication methods (P00C14)	• Professional
Ontario - *Ministry of Health*	• Yes (P00C16)	Formal<br>• Provincial translation services (P00A4) (59)<br>• Regional translation services<br>○ Home and Community Care Support Services (P00C16)	• Speech language pathologists s (P00C16)<br>• Communication tools shared through Canadian Association of MAiD Assessors and Providers (CAMAP) (P00C16)<br>• Teletypewriter services (59)	• Professional
SERVICE ORGANIZATIONS/REGIONAL FACILITIES				
Ontario - *Home and Community Care Support Services, Central East*	• Information on translation services not specified	• Information on translation services not specified	• Information on communication methods/interpretive services not specified	• Information on training requirement not specified
Ontario - *Home and Community Care Support Services, Waterloo Region*	• Yes (P00C4)	Informal<br>• Independent translators (P00C4)	• Information on communication methods/interpretive services not specified	• Professional
Ontario - *Home and Community Care Support Services, South East*	• Yes (P00C20)	Formal<br>• Regional translation service<br>○ Home and Community Care Support Services (P00C20)	• CAMAP non-verbal communication guide (P00C20)	• Professional
Ontario - *Home and Community Care Support Services, South West*	• Yes (P00C23)	Formal<br>• Regional translation service<br>○ In-Person (P00C23)<br>○ Virtual (P00C23)<br>Informal<br>• Family (P00C23)	• Closed-ended questions<br>○ Gestures (P00C23)<br>• Ocular movement boards (P00C23)<br>• Familiarization with patient provided communication methods (P00C23)	• Professional
Ontario - *Champlain Regional MAiD Network, The Ottawa Hospital*	Yes (P00C5)	Formal<br>• Translation services (P00C5)	• Speech language pathologists (P00C5)	• Professional
HEALTHCARE FACILITIES				
Ontario - *Peterborough Regional Health Centre*	• Information on translation services not specified	• Information on translation services not specified	• Information on communication methods/interpretive services not specified	• Information on training requirement not specified
Ontario - *Mount Sinai Healthcare Facility, Toronto*	• Yes (P00C36)	Formal<br>• Institutional translation services<br>○ In-Person (P00C36)	• Speech language pathologists (P00C36)	• Professional
Ontario - *University Health Network*	• Yes (P00C8)	Formal<br>• Institutional translation services (P00C8)	• American Sign Language interpretation (P00C8)	• Professional
Ontario - *Grand River Hospital*	• Yes	Formal<br>• Translation application (P00C50)	• Closed ended questions (P00C50)<br>• Gestures (P00C50)	• Professional
COMMUNITY OF PRACTICE				
Ontario - *Hamilton Family Health Team*	• Yes (P00B18)	Formal<br>• No official translators (P00B18)<br>Informal<br>• Family (P00B18)<br>• Bilingual medical provider (P00B18)	• Information on communication methods/interpretive services not specified	• Professional
Ontario - *Niagara Community MAiD Team, St. Catharine's, Niagara*	• Yes (P00C11)	Formal<br>• Provincial translation service<br>○ Virtual (P00C11)<br>Informal<br>• Neighbors (P00C11)	• Closed-ended questions (P00C11)<br>• Gestures (P00C11)	• Professional
GEOGRAPHICAL AREAS				

Jurisdiction	Are translation services available?	Who provides translation services?	What communication methods and/or interpretive services are available for non-verbal patients?	What is Translator/Speech Language Pathologist training requirements?
Ontario - *Oakville & Mississauga area*	• Yes (P00C18)	Formal<br>• Regional translation service<br>○ Home and Community Care Support Services<br>▪ Phone (P00C18)	• Closed-ended questions (P00C18)<br>• Ocular movement boards (P00C18)	• Professional
Ontario – *Oakville, Mississauga, & Burlington area*	• Yes (P00C21)	Informal<br>• Family (P00C21)	• Closed-ended questions (P00C18)<br>• Gestures (P00C21)<br>• Familiarization with patient provided communication methods (P00C21)	• Information on training requirement not specified
Ontario - *Renfrew County*	• No experience (P00C22)	• Information on translation services not specified	• Information on communication methods/interpretive services not specified	• Information on training requirement not specified
Ontario - *Greater Toronto Area*	• Yes (P00C24)	Informal<br>• Family (P00C24)	• Speech language pathologists (P00C24)<br>• Writing boards (P00C24)<br>• Closed-ended questions (P00C24)<br>• CAMAP non-verbal communication guide (P00C24)	• Information on training requirement not specified
Ontario - *Waterloo area*	• Yes (P00C25)	Formal<br>• Translation services (P00C25)<br>○ Virtual (P00C25)	• Ocular movement boards (P00C25)<br>• Multiple choice questions and answers (P00C25)<br>• Written communication (P00C25)	• Professional
Ontario - *Noelville, Sudbury, Elliot Lake, Sturgeon Falls area*	• Yes (P00C26)	Formal<br>• No official translation services (P00C26)<br>• No access to provincial translation service (P00C26)<br>Informal<br>• Family/friends (P00C26)	• Written communication (P00C26)	• Information on training requirement not specified
Québec - *Ministry of Health and Social Services*	• Information on translation services not specified	• Information on translation services not specified	• Information on communication methods/interpretive services not specified	• Information on training requirement not specified
INTEGRATED HEALTH AND SOCIAL SERVICES CENTRES				
Québec – *CISS Montérégie*	• Information on translation services not specified	• Information on translation services not specified	• Information on communication methods/interpretive services not specified	• Information on training requirement not specified
INTEGRATED UNIVERSITY HEALTH AND SOCIAL SERVICES CENTRES				
Québec – *CIUSS Capitale-Nationale*	• No experience (P00C37)	• No experience (P00C37)	• No experience (P00C37)	• No experience (P00C37)
Québec –*University of Montreal Hospital Center*	• Yes (P00C40)	• Information on translation services not specified	• Information on communication methods/interpretive services not specified	• Information on training requirement not specified
Québec – *McGill University Health Centre*	• Yes (P00C9)	Informal<br>• Family (P00C9)	• Speech language pathologists (P00C9)	• Professional

Table 9: Interpretation of reasonably foreseeable natural deaths

Jurisdiction	Common elements within definitions: What guidelines do practitioners use to inform foreseeability of death?	How do practitioners interpret foreseeability?	What do practitioners use to determine foreseeability?	Is there a specific timeframe to determine foreseeability of death?	Is there any requirement that has to be considered?
Government of Canada	• Guideline used to inform foreseeability of death not specified	• Clinician experience<br>○ "Physicians and nurse practitioners have the necessary expertise to evaluate each person's unique circumstances..." (102)	• Trajectory towards death<br>○ "...change in the state of their medical condition so that it has become fairly clear that they are on an irreversible path toward death..." (102)	• No<br>○ "... person's death would need to be foreseeable in the not-too-distant future" (102)	• No additional requirements specified
Alberta - *Alberta Health Services*	• Canadian Medical Protective Association (CMPA) guidelines (P00A5)<br>• Canadian Association of Medical Assessors and Providers (CAMAP) guidelines (P00C39)	• Clinician experience<br>○ "Varies based on the assessor's and provider's judgment" (P00A5, P00C6, P00C12)<br>• Patients' capacity to make decision (P00C10)<br>• As a legal term<br>○ Advice from lawyers (P00A5)	• Use of specific prediction tools<br>○ "Assessors also consider the trajectory of decline to inform their decision on foreseeability, using prediction tools to aid in their judgment" (P00A5, P00B13, P00C39)<br>• Trajectory towards death<br>○ "...signifies predictable decline or trajectory towards death" (P00B9, P00C39)	• No<br>○ "Varies between weeks to months" (P00A5, P00B9, P00B13, P00C6, P00C12)	• Yes<br>○ Specialist assessment (P00C39)<br>○ Assessors explore options to relieve suffering (P00C12, P00C39)
British Columbia - *Fraser Health*	• CAMAP guidelines (P00C17)<br>○ Not endorsed for official use by Ministry of Health (P00A4)	• Clinician experience<br>○ "Varies among providers, ...based on the providers' experience" (P00C17)<br>○ Adoption of a liberal approach (P00C17)	• Patient vulnerability (P00A10)<br>• Trajectory towards death (P00A10)	• No<br>○ Depends on assessor's interpretation (P00A10)	• No additional requirements specified
British Columbia - *Interior Health*	• CAMAP guidelines (P00C29)<br>○ Not endorsed for official use by Ministry of Health (P00A4)	• Assessment of patient's medical records/history (P00C29)	• Trajectory towards death (P00C29)<br>• Patient vulnerability (P00C29)	• Yes<br>○ "Within 6-12 months" (P00C29)	• Yes<br>○ Assessors discuss options to relieve suffering (P00B14)
British Columbia - *Island Health*	• CAMAP guidelines (P00A9, P00C13)<br>○ Not endorsed for official use by Ministry of Health (P00A4)	• Clinician experience<br>○ "Assessors bring their lived and professional experiences..." (P00A9, P00C13)<br>• As a legal term<br>○ "It is a legal term..., only the courts can interpret..." (P00A13, P00C13)	• Trajectory towards death<br>○ "...trajectory of patients with certain diseases is predictable" (P00A13, P00C13)	• No<br>○ "The exact time is not predictable..." (P00A13, P00C13)	• Yes<br>○ Patients are provided with information about options to relieve suffering (P00C13)<br>▪ Patients are provided with brochures on palliative care and other support services (P00A9)
British Columbia - *Northern Health*	• BC College of Physicians and Surgeons guidelines (P00A8)	• Clinician experience<br>○ Adoption of a liberal approach (P00A8)<br>• As a legal term<br>○ "Only the supreme court can advise on how the term is defined" (P00A8)	• Trajectory towards death (P00A8)	• No<br>○ Varies between weeks to months (P00A8)	• No additional requirements specified
British Columbia - *Vancouver Coastal Health*	• CAMAP guidelines (P00B12)<br>○ Not endorsed for official use by Ministry of Health (P00A4)	• Clinician experience<br>○ "Varying definition as providers' experiences grow" (P00A7, P00B15, P00B10, P00B16)<br>• As a legal term<br>○ "Reasonably foreseeable natural death is a legal term..." (P00B15, P00B16)	• Trajectory towards death (P00B15)<br>• Patient vulnerability (P00B15)	• Yes<br>○ Varies between weeks to months (P00A7, P00B15)	• Yes<br>○ Assessors discuss options to relieve suffering (P00B10)
British Columbia - *Provincial Health Services Authority*	• Guideline used to inform foreseeability of death not specified	• The approach to interpret foreseeability not specified	• The definition of foreseeability of death not specified	• Timeframe for foreseeability of death not specified	• No additional requirements specified
Manitoba - *Shared Health*	• Guideline used to inform foreseeability of death not specified	• Clinician experience<br>○ "Varying definition among practitioners" (P00B19, P00C33, P00C34)<br>• Assessment of patient's medical record/history (P00C33)	• Trajectory towards death (P00B19, P00C33, P00C34)<br>• Patient vulnerability (P00B19)<br>• The surprise question<br>○ "Would I be surprised if..." (P00C33)	• Yes<br>○ "6-12 months, up to 2 years" (P00C33)	• Yes<br>○ Specialist assessment (P00C33)<br>▪ "...we need patients to meet with cancer specialist to go over their treatment options..." (P00C34)<br>▪ Explore available treatment options that can reduce suffering (P00B19, P00C34)

Jurisdiction	Common elements within definitions<br>What guidelines do practitioners use to inform foreseeability of death?	How do practitioners interpret foreseeability?	What do practitioners use to determine foreseeability?	Is there a specific timeframe to determine foreseeability of death?	Is there any requirement that has to be considered?
New Brunswick - *Horizon Health Network*	• Clinician specific (P00C15)	• Clinician experience<br>○ "Adoption of a more liberal approach..." (P00C15)	• The surprise question<br>○ "Would I be surprised if ..." (P00C15)<br>• Patient vulnerability<br>○ " Consideration for factors such as the patient's fragility" (P00C15)	• Yes<br>○ Varies between weeks to months (P00C15)	• Yes<br>○ Specialist assessment (P00C15)
New Brunswick - *Vitalité Health Network*	• Guideline used to inform foreseeability of death not specified	• Clinician experience (P00C48)	• Trajectory towards death (P00C48)	• No<br>○ "... person's death would happen at some point from the situation" (P00C48)	• No additional requirements specified
Newfoundland and Labrador – *Eastern Zone*	• CAMAP guidelines (P00C43)	• Clinician experience (P00C43)	• Trajectory towards death<br>○ "Prognosis of a specific length..." (P00C43)	• Yes<br>○ "...until the patient's death naturally happens"<br>○ Varies from days to weeks (P00C43)	• No additional requirements specified
Newfoundland and Labrador – *Western Zone*	• Guideline used to inform foreseeability of death not specified	• Clinician experience (P00C44)	• Trajectory towards death (P00C44)<br>• Patient vulnerability (P00C44)	• Timeframe for foreseeability of death not specified	• Yes<br>○ Patients are provided with information about options to relieve suffering (P00C44)
Newfoundland and Labrador – *Central Zone*	• Guideline used to inform foreseeability of death not specified	• The approach to interpret foreseeability not specified	• The definition of foreseeability of death not specified	• Timeframe for foreseeability of death not specified	• No additional requirements specified
Northwest Territories	• CAMAP guidelines (P00A3, P00C35)	• Clinician experience (P00A2)	• Trajectory towards death<br>○ "Does not require a prognosis..." (11)<br>• The surprise question<br>○ "Would I be surprised if..." (P00C35)	• No<br>○ "Does not require the length of time the patient may live" (11)	• No additional requirements specified
Nova Scotia - *Nova Scotia Health*	• Guideline used to inform foreseeability of death not specified	• Clinician experience (P00C38)<br>• As a legal term<br>○ Reliance on legal experts for interpretation (P00A12)<br>• Assessment of patient's medical record/history (P00A12)	• Trajectory towards death<br>○ "...healthcare professional can reasonably predict the natural death" (P00A12, P00C38)<br>• Patient vulnerability<br>○ "Taking all medical circumstances into account" (P00A12, P00C38)	• Timeframe to determine foreseeability of death not specified	• No additional requirements specified
Prince Edward Island - *Health PEI*	• Guideline used to inform foreseeability of death not specified	• Clinician experience (P00B8)	• The surprise question<br>○ "Would I be surprised if ..." (P00B8, P00B17)	• Yes<br>○ Varies from weeks to months (P00B8)	• No additional requirements specified
Saskatchewan - *Saskatchewan Health Authority*	• CAMAP guidelines (P00C1)<br>• Federal legislation (P00B1)	• Clinician experience (P00C1)<br>• As a legal term<br>• Reliance on legal interpretation (P00C1)	• Trajectory towards death (P00A1, P00B1, P00C1, P00C19)<br>• Patient vulnerability (P00A1)	• Yes<br>○ Up to 6 months (P00C19, P00C1, P00B1, P00A1)	• No additional requirements specified
Yukon - *Department of Health and Social Services*	• Guideline used to inform foreseeability of death not specified	• Clinician experience (P00C7, P00C14)	• Trajectory towards death (P00C14)	• No<br>○ "Death could happen quickly" (P00C14)	• No additional requirements specified
Ontario - *Ministry of Health*	• Federal legislation (P00B4)<br>• Clinician specific (P00B4)	• The approach to interpret foreseeability not specified	• The surprise question<br>○ "Would I be surprised if ..." (P00C16)<br>• Patient vulnerability<br>○ "...patient's capability to complete daily activities (P00C16)	• No<br>○ No timeline is set (P00B4)	• Yes<br>○ Specialist assessment (P00C16)
SERVICE ORGANIZATIONS/REGIONAL FACILITIES					
Ontario - *Home and Community Care Support Services, Central East*	• CAMAP guidelines (P00C2)	• The approach to interpret foreseeability not specified	• Use of predictive tools<br>○ Prognostic Indicator Guideline (P00C2)<br>○ Rockwood frailty scale (P00C2)<br>○ Palliative predictive scale (P00C2)	• No<br>○ No timeline is set (P00C2)	• No additional requirements specified

Jurisdiction	Common elements within definitions<br>What guidelines do practitioners use to inform foreseeability of death?	How do practitioners interpret foreseeability?	What do practitioners use to determine foreseeability?	Is there a specific timeframe to determine foreseeability of death?	Is there any requirement that has to be considered?
Ontario - *Home and Community Care Support Services, Waterloo Region*	• Guideline used to inform foreseeability of death not specified	• Clinician experience (P00C4)	• Trajectory towards death (P00C4)<br>• Patient vulnerability (P00C4)	• No<br>○ No timeline is set (P00C4)	• No additional requirements specified
Ontario - *Home and Community Care Support Services, South East*	• Guideline used to inform foreseeability of death not specified	• The approach to interpret foreseeability not specified	• Trajectory towards death (P00C20)<br>• Patient's vulnerability (P00C20)	• Timeframe to determine foreseeability of death not specified	• No additional requirements specified
Ontario - *Home and Community Care Support Services, South West*	• Guideline used to inform foreseeability of death not specified	• A legal term (P00C23)	• Use of predictive tools (P00C23)<br>• The surprise question<br>○ "Would I be surprised if…" (P00C23)	• Timeframe to determine foreseeability of death not specified	• No additional requirements specified
Ontario - *Champlain Regional MAiD Network, the Ottawa Hospital*	• Guideline used to inform foreseeability of death not specified	• Clinician experience (P00C5)	• Trajectory towards death (P00C5)<br>• Patient vulnerability (P00C5)	• Timeframe to determine foreseeability of death not specified	• No additional requirements specified
HEALTHCARE FACILITIES					
Ontario - *Peterborough Regional Health Centre*	• Guideline used to inform foreseeability of death not specified	• Clinician experience<br>○ "...considered fairly liberal" (P00C3)	• Trajectory towards death (P00C3)<br>• The surprise question<br>○ "Would I be surprised if …" (P00C3)	• Timeframe to determine foreseeability of death not specified	• No additional requirements specified
Ontario - *Mount Sinai Healthcare Facility, Toronto*	• Guideline used to inform foreseeability of death not specified	• A legal term (P00C36)	• Trajectory towards death (P00C36)	• Yes<br>○ Up to 6 months (P00C36)	• No additional requirements specified
Ontario - *University Health Network*	• Guideline used to inform foreseeability of death not specified	• Clinician experience (P00C8)	• Trajectory towards death (P00C8)	• Yes<br>○ Varies from weeks to months (P00C8)	• No additional requirements specified
Ontario - *Grand River Hospital*	• Guideline used to inform foreseeability of death not specified	• Clinician experience (P00C50)<br>• Assessment of patient's medical record/history (P00C50)	• Trajectory towards death (P00C50)<br>• Use of predictive tools (P00C50)<br>○ Palliative predictive scale	• Timeframe to determine foreseeability of death not specified	• Yes<br>○ Specialist assessment (P00C50)<br>▪ "... they do all usual things to reverse the acute failure and realize they can't..." (P00C50)
COMMUNITY OF PRACTICE					
Ontario - *Hamilton Family Health Team*	• Guideline used to inform foreseeability of death not specified	• The approach to interpret foreseeability not specified	• The surprise question<br>○ "Would I be surprised if…" (P00C32)	• Timeframe to determine foreseeability of death not specified	• No additional requirements specified
Ontario - *Niagara Community MAiD Team, St. Catharine's, Niagara*	• Guideline used to inform foreseeability of death not specified	• Clinician experience<br>○ "...a liberal approach..." (P00C11)	• Trajectory towards death (P00C11)	• Yes<br>○ "5-10 years" (P00C11)	• No additional requirements specified
GEOGRAPHICAL AREAS					
Ontario - *Oakville & Mississauga area*	• Guideline used to inform foreseeability of death not specified	• Clinician experience (P00C18)	• Trajectory towards death (P00C18)	• Timeframe to determine foreseeability of death not specified	• No additional requirements specified
Ontario - *Oakville, Mississauga & Burlington area*	• Guideline used to inform foreseeability of death not specified	• Clinician experience (P00C21)<br>• Frailty scale (P00C21)	• The surprise question<br>○ "Would I be surprised if…" (P00C21)	• Timeframe to determine foreseeability of death not specified	• No additional requirements specified
Ontario - *Renfrew County*	• CAMAP guidelines (P00C22)	• The approach to interpret foreseeability not specified	• Use of predictive tools (P00C22)	• Timeframe to determine foreseeability of death not specified	• No additional requirements specified
Ontario - *Greater Toronto Area*	• CAMAP guidelines (P00C24)	• Clinician experience (P00C24)	• Trajectory towards death (P00C24)	• Yes<br>○ Up to 2 years (P00C24)	• No additional requirements specified
Ontario - *Waterloo area*	• CAMAP guidelines (P00C25)	• The approach to interpret foreseeability not specified	• Trajectory towards death (P00C25)<br>• Patient's vulnerability (P00C25)	• Timeframe to determine foreseeability of death not specified	• No additional requirements specified

Jurisdiction	Common elements within definitions<br>What guidelines do practitioners use to inform foreseeability of death?	How do practitioners interpret foreseeability?	What do practitioners use to determine foreseeability?	Is there a specific timeframe to determine foreseeability of death?	Is there any requirement that has to be considered?
Ontario - *Noelville, Sudbury, Elliot Lake, Sturgeon Falls area*	• Guideline used to inform foreseeability of death not specified	• The approach to interpret foreseeability not specified	• Trajectory towards death (P00C26)	• Yes<br>○ Varies between 6 weeks to 3 years, or up to 10 years (P00C26)	• No additional requirements specified
Québec - *Ministry of Health and Social Services*	• College of Medicine of Québec (103)<br>• Commission on end-of-life guidelines (103)	• Clinician experience (103)	• Trajectory towards death (103)	• Timeframe to determine foreseeability of death not specified	• Yes<br>○ Specialist assessment (103)
INTEGRATED HEALTH AND SOCIAL SERVICES CENTRES					
Québec – *CISS Montérégie*	• Provincial legislation (P00C27)<br>• Commission on end-of-life guidelines (103) (P00C27)	• The approach to interpret foreseeability not specified	• The definition of foreseeability of death not specified	• Timeframe to determine foreseeability of death not specified	• No additional requirements specified
INTEGRATED UNIVERSITY HEALTH AND SOCIAL SERVICES CENTRES					
Québec – *CIUSS Capitale-Nationale*	• Commission on end-of-life guidelines (103)	• Clinician experience (P00C37)	• Trajectory towards death (P00C37)	• Timeframe to determine foreseeability of death not specified	• Yes<br>○ Specialist assessment (P00C37)
Québec – *University of Montreal Hospital Center*	• Commission on end-of-life guidelines (103)	• The approach to interpret foreseeability not specified	• The definition of foreseeability of death not specified	• Timeframe to determine foreseeability of death not specified	• No additional requirements specified
Québec – *McGill University Health Centre*	• Provincial legislation (P00C9)<br>• Commission on end-of-life guidelines (103) (P00C9)	• Clinician experience (P00C9)	• The definition of foreseeability of death not specified	• Yes<br>○ Within 18 months (P00C9)	• No additional requirements specified

Table 10: Interpretation of non-reasonably foreseeable natural deaths

	Common elements within definitions					
**Jurisdiction**	**How is the 90-day waiting period calculated?**	**Can the 90-day waiting period be reduced?**	**What is the role of specialist consult?**	**How do assessors determine suffering?**	**What is timeframe to determine foreseeability of death?**	**Is there a requirement for the assessors to explore alternative treatment options with the patient?**
Government of Canada	• From date first assessment begins to provision day (104)	• Yes<br>○ If both assessors agree patient is at risk of losing capacity (105)	• Provide options to relieve suffering (105)	• Methods to determine suffering by assessors not described	• Timeframe to determine foreseeability of death not identified	• Yes<br>○ Informed of available and appropriate means to relieve suffering, including counselling services, mental health and disability support services, community services, and palliative care (105)<br>○ Assessors are required to discuss treatment options to relieve suffering (105)<br>○ Ensures patient has given considerations to means to relieve suffering (105)
Alberta - *Alberta Health Services*	• From date first assessment begins to provision day (77)	• Yes<br>○ If both assessors agree patient is at risk of losing capacity (77)	• Provide options to relieve suffering (P00C39)<br>• Examine current health status and prognosis (P00B13)	• Review medical records (P00C12)<br>• Review specialists consult notes and outcomes (P00C39)<br>• Confirm impact of medical conditions on quality of life (P00C6)<br>• Ask questions about patients' experience (P00C6)<br>• Conduct follow up assessments (P00C6)	• Within months to years (P00A5, P00B9)	• Yes<br>○ Determines treatment options patient has explored (P00C39)<br>▪ Assessors consider whether a person would have tried treatment options (P00C39, P00C12)
British Columbia - *Fraser Health*	• From date first assessment begins to provision day (6, 28, 106)	• Yes<br>○ If both assessors agree patient is at risk of losing capacity (27)	• Act as an assessor to determine eligibility (P00A10)<br>• Provide options to relieve suffering (P00A10)	• Review medical records (P00A10)<br>• Ask questions related to suffering (P00A10)<br>○ Explore nature and extent of suffering (P00A10)	• Timeframe to determine foreseeability of death not identified	• Requirement for exploring alternative treatment options not described
British Columbia - *Interior Health*	• From date first assessment begins to provision day (33)	• Yes<br>○ If both assessors agree patient is at risk of losing capacity (27)	• Role of specialist consults not described	• Review medical records (P00C29)<br>• Use predictive tools<br>○ Supportive & Palliative Care Indicators Tool (SPICT) to determine frailty (P00B14)<br>• Ask questions related to suffering (P00C29)<br>• Clarify reasons for requesting MAiD (P00C29)<br>• Rely on their expertise (P00C29)	• Timeframe to determine foreseeability of death not identified	• Yes<br>○ Assessors discuss options to relieve suffering (P00B14)
British Columbia - *Island Health*	• From date first assessment begins to provision day (107)	• Yes<br>○ If both assessors agree patient is at risk of losing capacity (27)(P00A9)	• Provide options to relieve suffering (P00C13)	• Review consults notes (P00A13, P00C13)<br>• Assessors' expertise (P00A9)<br>• Conduct follow up assessments (P00C13)	• Varies<br>○ Assessor's judgment (P00A9)<br>○ Guidance from CAMAP (P00A9)	• Yes<br>○ Patients have explored options to relieve suffering (P00C13)<br>○ Assessors make appropriate referrals to specialists (P00C13)<br>○ Patients are provided with brochures on palliative care and other support services (P00A9)
British Columbia - *Northern Health*	• From date first assessment begins to provision day (27)	• Yes<br>○ If both assessors agree patient is at risk of losing capacity (27)	• Conduct capacity assessments (27)	• Methods to determine suffering by assessors not described<br>○ Limited experience (P00A8)	• Timeframe to determine foreseeability of death not identified<br>○ Limited experience (P00A8)	• Requirement for exploring alternative treatment options not described<br>○ Limited experience (P00A8)
British Columbia - *Vancouver Coastal Health*	• From date first assessment begins to provision day (27)	• Yes<br>○ If both assessors agree patient is at risk of losing capacity (27)	• Provide options to relieve suffering (P00B16)<br>• Conduct capacity assessments (P00B15)	• Review medical records (P00B16)<br>• Ask questions related to suffering (P00B10)<br>• Clarify reasons for requesting MAiD (P00B10)<br>• Review treatment options explored by patients (P00B15)<br>• Rely on their expertise (P00B16)<br>• Conduct follow up assessments (P00B15)	• Timeframe to determine foreseeability of death not identified	• Yes<br>○ Patients are connected with resources to relieve suffering (P00B10)

Jurisdiction	Common elements within definitions: How is the 90-day waiting period calculated?	Can the 90-day waiting period be reduced?	What is the role of specialist consult?	How do assessors determine suffering?	What is timeframe to determine foreseeability of death?	Is there a requirement for the assessors to explore alternative treatment options with the patient?
British Columbia - *Provincial Health Services Authority*	• Not applicable<br>○ Provides services to patients whose deaths are foreseeable	• Not applicable<br>○ Provides services to patients whose deaths are foreseeable	• Not applicable<br>○ Provide services to patients whose deaths are foreseeable	• Not applicable<br>○ Provides services to patients whose deaths are foreseeable	• Not applicable<br>○ Provides services to patients whose deaths are foreseeable	• Not applicable<br>○ Provides services to patients whose deaths are foreseeable
Manitoba - *Shared Health*	• From date first assessment begins to provision day (P00C33)	• Yes<br>○ If both assessors agree patient is at risk of losing capacity (25)	• Provide options to relieve suffering (P00C19)	• Review medical records (P00C33)<br>• Review specialists consult notes (P00C33)<br>• Ask questions related to suffering (P00C34, P00B19)<br>• Clarify reasons for requesting MAiD (P00C34, P00B19)<br>• Confirm impact of medical conditions on quality of life (P00B19, P00C34)	• Life expectancy for cases varies (P00C11, P00C34, and P00C33).	• Yes<br>○ A psychosocial assessment is completed (P00C33)<br>○ Explore available treatment options that can reduce suffering (P00B19, P00C34)
New Brunswick - Horizon Health Network	• From date first assessment begins to provision day (9)	• Yes<br>○ If both assessors agree patient is at risk of losing capacity (P00C15)	• Provide options to relieve suffering (P00C15)	• Review medical records (P00C15)<br>• Examine current health status and prognosis (P00C15)	• Timeframe to determine foreseeability of death not identified	• Yes<br>○ Assessors make referrals to specialists (P00C15)
New Brunswick - *Vitalité Health Network*	• From date first assessment begins to provision day (P00C48)	• Yes<br>○ If both assessors agree patient is at risk of losing capacity and approval from Risk Management Committee (P00C48)	• Provide expertise on patient's medical condition (P00C48)	• Examine current health status and prognosis (P00C48)	• Timeframe to determine foreseeability of death not identified	• Requirement for exploring alternative treatment options not described
Newfoundland and Labrador - *Eastern Zone*	• From date first assessment begins to provision day (P00C43)	• Yes<br>○ If both assessors agree patient is at risk of losing capacity (P00C43)	• Provide options to relieve suffering (P00C43)	• Ask questions related to suffering (P00C43)<br>• Clarify reasons for requesting MAiD (P00C43)	• Timeframe to determine foreseeability of death not identified	• Yes<br>○ Assessors make referrals to specialists (P00C43)
Newfoundland and Labrador - *Western Zone*	• From date first assessment begins to provision day (P00C44)	• Yes<br>○ If both assessors agree patient is at risk of losing capacity (P00C44)	• Provide options to relieve suffering (P00C44)	• Review medical records (P00C44)<br>• Review specialist consult notes (P00C44)<br>• Ask questions related to suffering (P00C44)<br>• Confirm impact of medical conditions on mobility and quality of life (P00C44)	• Timeframe to determine foreseeability of death not identified	• Yes<br>○ Assessors make referrals to specialists (P00C44)
Newfoundland and Labrador - *Central Zone*	• From date first assessment begins to provision day (P00B22)	• Yes<br>○ If both assessors agree patient is at risk of losing capacity (P00B22)	• Role of specialist consults not described<br>○ Limited experience (P00B22)	• Methods to determine suffering by assessors not described<br>○ Limited experience (P00B22)	• Timeframe to determine foreseeability of death not identified<br>○ Limited experience (P00B22)	• Requirement for exploring alternative treatment options not described<br>○ Limited experience (P00B22)
Northwest Territories	• From date first assessment begins to provision day (11)	• Yes<br>○ If both assessors agree patient is at risk of losing capacity (11)	• Provide options to relieve suffering (P00A3)<br>• Inform assessors decision-making (11)	• Review medical records (P00A3)<br>• Use predictive tool (P00A3)<br>○ Edmonton Symptom Assessment System (ESAS) (P00A2)<br>○ Palliative Performance Scale (P00A3)<br>• Ask questions related to suffering (P00A2)	• Timeframe to determine foreseeability of death not identified	• Yes<br>○ Informed of available and appropriate means to relieve suffering (P00A3)<br>○ Assessors discuss treatment options to relieve suffering (P00A3)<br>○ Ensures patient has given considerations to means to relieve suffering (P00A3)<br>○ "Reasonable options" are considered on a case-by-case basis (P00C35)
Nova Scotia - *Nova Scotia Health*	• From date first assessment begins to provision day (13)	• Yes<br>○ If both assessors agree patient is at risk of losing capacity (13)	• Assist in determining eligibility or capacity (P00A12)	• Review medical records (P00C38)<br>• Use predictive tool<br>○ Complete the palliative performance scale (P00A12)<br>• Ask questions related to suffering (P00C38)<br>• Clarify reasons for requesting MAiD (P00C38)<br>• Confirm impact of medical condition on quality of life (P00C38)<br>• Discuss treatment options patient has explored (P00C38)	• Timeframe to determine foreseeability of death not identified	• Yes<br>○ Given consideration to other means to relieve suffering (P00C38)

Jurisdiction	Common elements within definitions<br>How is the 90-day waiting period calculated?	Can the 90-day waiting period be reduced?	What is the role of specialist consult?	How do assessors determine suffering?	What is timeframe to determine foreseeability of death?	Is there a requirement for the assessors to explore alternative treatment options with the patient?
Prince Edward Island - *Health PEI*	• From date first assessment begins to provision day (P00B17)	• Yes<br>○ If both assessors agree patient is at risk to losing capacity (P00B8)	• Determine if patients have been offered options to relieve suffering (P00B8)<br>• Assess patient's condition (P00B8)<br>• Assess patient's capacity (P00B8)	• Review medical record (22)<br>• Examine current health status and prognosis (P00B8)<br>• Review specialist consults note (P00B8)<br>• Clarify reason for requesting MAiD (P00B8)<br>• Confirm impact of medical conditions on function and quality of life (P00B17)<br>• Discuss treatment options patient has explored (P00B17)	• Timeframe to determine foreseeability of death not identified	• Yes<br>○ Assessors will request a specialist consult (P00B8)
Saskatchewan - *Saskatchewan Health Authority*	• From date first assessment begins to provision day (88)	• Yes<br>○ If both assessors agree patient is at risk of losing capacity (108)	• Assess patient's condition (P00C19)<br>• Provide additional information regarding condition (P00C19)	• Review medical records (P00C19)<br>• Confirm means to relieve suffering (P00B1)<br>• Ask questions related to suffering and reason for requesting MAiD (P00C19)<br>• Determine suffering based on diagnosis (P00C19)<br>• Conduct follow up assessment (P00A1)	• Timeframe to determine foreseeability of death not identified	• Yes<br>○ Assessors will request a specialist consult (P00C19)
Yukon - *Department of Health and Social Services*	• 90- clear days from the day after first assessment begins (90, 109)	• Yes<br>○ If both assessors agree patient is at risk of losing capacity (90, 109)	• Provide options to relieve suffering (P00C14)	• Review medical records (P00C14)<br>• Review consults notes (P00C14)<br>• Ask questions related to suffering (P00C14)<br>• Examine current health status and prognosis (P00C14)	• Timeframe to determine foreseeability of death not identified	• Yes<br>○ Given consideration to means to relieve suffering (P00C14)
Ontario - *Ministry of Health*	• From date first assessment begins to provision day (24) (P00B4)	• Yes<br>○ If both assessors agree patient is at risk of losing capacity (P00A4) (24)	• Assess patient's condition (P00A4)<br>• Informs assessors decision-making (P00A4)	• Ask questions related to cause of suffering (P00A4)	• Timeframe to determine foreseeability of death not identified	• Yes<br>○ Assessors will request a specialist consult (P00B4)
SERVICE ORGANIZATIONS/REGIONAL FACILITIES						
Ontario - *Home and Community Support Services, Central East*	• Calculation of 90-day waiting period not identified	• Reduction of 90-day waiting period not identified	• Confirm current health status and prognosis (P00C2)	• Ask questions about patients' medical history (P00C2)<br>• Confirm impact of medical conditions on quality of life (P00C2)	• Timeframe to determine foreseeability of death not identified	• Yes<br>○ Assessors will request a specialist consult (P00C2)<br>○ Patients are informed of options to relieve suffering (P00C2)
Ontario - *Home and Community Care Support Services, Waterloo Region*	• Calculation of 90-day waiting period not identified	• Reduction of 90-day waiting period not identified	• Role of specialist consults not described	• Confirm impact of medical conditions on function and quality of life<br>• Examine current health status and prognosis (P00C4)<br>• Discuss treatment options with patient (P00C4)	• Timeframe to determine foreseeability of death not identified	• Yes<br>○ Patients are informed of options to relieve suffering (P00C4)
Ontario - *Home and Community Care Support Services, South East*	• From date first assessment begins to provision day (P00C20)	• Reduction of 90-day waiting period not identified	• Provide options to relieve suffering (P00C20)	• Review medical records (P00C20)<br>• Asks questions about patients' experience (P00C20)<br>• Confirm impact of medical conditions on quality of life (P00C20)<br>• Conduct follow up assessments (P00C20)<br>• Discuss treatment options with patient (P00C20)	• Timeframe to determine foreseeability of death not identified	• Yes<br>○ Patients are informed of options to relieve suffering (P00C20)<br>○ Patients have tried treatment options or given consideration to treatment options (P00C20)
Ontario - *Home and Community Care Support Services, South West*	• Calculation of 90-day waiting period not identified	• Reduction of 90-day waiting period not identified	• Role of specialist consults not described	• Ask questions about patients' experience (P00C23)<br>• Confirm impact of medical conditions on quality of life (P00C23)	• Timeframe to determine foreseeability of death not identified	• Yes<br>○ Patients are aware of options to relieve suffering (P00C23)<br>○ Patients have attempted treatments (P00C23)

Jurisdiction	Common elements within definitions					
	**How is the 90-day waiting period calculated?**	**Can the 90-day waiting period be reduced?**	**What is the role of specialist consult?**	**How do assessors determine suffering?**	**What is timeframe to determine foreseeability of death?**	**Is there a requirement for the assessors to explore alternative treatment options with the patient?**
Ontario - *Champlain Regional MAiD Network, the Ottawa Hospital*	• From date first assessment begins to provision day (P00C5)	• Reduction of 90-day waiting period not identified	• Role of specialist consult not described	• Review medical records (P00C5)<br>• Clarify reasons for requesting MAiD (P00C5)<br>• Examine current health status and prognosis (P00C5)<br>• Discuss treatment options with patient (P00C5)	• Death can occur in years (P00C5)	• Requirement for exploring alternative treatment options not described
HEALTHCARE FACILITIES						
Ontario - *Peterborough Regional Health Care*	• Calculation of 90-day waiting period not identified	• Reduction of 90-day waiting period not identified	• Provide options to relieve suffering (P00C3)	• Review medical records (P00C3)<br>• Review specialists consult notes (P00C3)<br>• Examine current health status and prognosis (P00C3)	• Timeframe to determine foreseeability of death not identified	• Requirement for exploring alternative treatment options not described<br>○ Limited experience with not foreseeable death patients (P00C3)<br>○ Patients have already sought specialist consults before requesting MAiD (P00C3)
Ontario - *Mount Sinai Healthcare Facility, Toronto*	• Calculation of 90-day waiting period not identified	• Reduction of 90-day waiting period not identified	• Determine capacity to consent to MAiD (P00C36)<br>• Inform assessors decision-making (P00C36)	• Review medical records (P00C36)<br>• Ask questions about patients' medical history (P0C36)<br>• Clarify reasons for requesting MAiD (P00C36)<br>• Understand patients decision-making process (P00C36)	• Timeframe to determine foreseeability of death not identified	• Yes<br>○ Depends on assessors' expertise and patient condition (P00C36)<br>○ Patients' satisfaction with treatment options (P00C36)
Ontario - *University Health Network*	• Calculation of 90-day waiting period not identified	• Reduction of 90-day waiting period not identified	• Assess patient's condition (P00C8)<br>• Provide additional information regarding condition (P00C8)	• Review of medical records (P00C8)<br>• Examine current health status and prognosis (P00C8)<br>• Discuss treatment options with patient (P00C8)	• Timeframe to determine foreseeability of death not identified	• Yes<br>○ Patients are informed of options to relieve suffering (P00C8)
Ontario - *Grand River Hospital*	• No experience<br>○ Inpatients do not stay in the facility for this period of time (P00C50)	• No experience<br>○ Inpatients do not stay in the facility for this period of time (P00C50)	• No experience<br>○ Inpatients do not stay in the facility for this period of time (P00C50)	• No experience<br>○ Inpatients do not stay in the facility for this period of time (P00C50)	• No experience<br>○ Inpatients do not stay in the facility for this period of time (P00C50)	• No experience<br>○ Inpatients do not stay in the facility for this period of time (P00C50)
COMMUNITY OF PRACTICE						
Ontario - *Hamilton Family Health Team*	• Calculation of 90-day waiting period not identified	• Reduction of 90-day waiting period not identified	• Provide options to relieve suffering (P00C32)<br>• Determine standard treatments for specific conditions (P00C32)	• Review medical records (P00C32)<br>• Examine current health status and prognosis (P00C32)<br>• Ask questions about patient's medical history (P00C32)<br>• Confirm impact of medical conditions on quality of life (P00C32)<br>• Ask questions related to suffering (P00C32)<br>• Clarify reasons for requesting MAiD (P00C32)<br>• Discuss treatment options or consults explored (P00C32)	• Timeframe to determine foreseeability of death not identified	• Yes<br>○ Patients attempt treatments that are successful and accessible (P00B18)
Ontario - *Niagara Community MAiD Team, St. Catharine's, Niagara*	• Calculation of 90-day waiting period not identified	• Reduction of 90-day waiting period not identified	• Explore options to relieve suffering (P00C11)<br>• Inform assessors decision-making (P00C11)	• Review medical records (P00C11)<br>• Review specialists consult notes (P00C11)<br>• Examines current health status and prognosis (P00C11)<br>• Confirm impact of medical conditions on quality of life (P00C11)<br>• Clarify reasons for requesting MAiD (P00C11)<br>• Conduct follow up assessments (P00C11)<br>• Consult with patients' health care providers (P00C11)	• Timeframe to determine foreseeability of death not identified	• Yes<br>○ Patients are aware of options to relieve suffering (P00C11)<br>○ Patients' satisfaction with treatment options (P00C11)

Jurisdiction	Common elements within definitions					
	**How is the 90-day waiting period calculated?**	**Can the 90-day waiting period be reduced?**	**What is the role of specialist consult?**	**How do assessors determine suffering?**	**What is timeframe to determine foreseeability of death?**	**Is there a requirement for the assessors to explore alternative treatment options with the patient?**
GEOGRAPHICAL AREAS						
Ontario - *Oakville & Mississauga area*	• Calculation of 90-day waiting period not identified	• Reduction of 90-day waiting period not identified	• Inform assessors decision-making process (P00C18)	• Review medical records (P00C18)<br>• Review specialists consult notes (P00C18)<br>• Ask questions about patients' experience (P00C18)<br>• Rely on their expertise (P00C18)	• Timeframe to determine foreseeability of death not identified	• Yes<br>○ Explore means to relieve suffering (P00C18)
Ontario - *Oakville, Mississauga, & Burlington area*	• Calculation of 90-day waiting period not identified	• Reduction of 90-day waiting period not identified	• Provide options to relieve suffering (P00C21)	• Review medical records (P00C21)<br>• Review specialists consult notes (P00C21)	• Timeframe to determine foreseeability of death not identified	• Yes<br>○ Explore means to relieve suffering (P00C21)
Ontario - *Renfrew County*	• Calculation of 90-day waiting period not identified	• Reduction of 90-day waiting period not identified	• Provide options to relieve suffering (P00C22)<br>• Inform assessors decision-making patients (P00C22)	• Review medical records (P00C22)<br>• Review specialists consult notes (P00C22)<br>• Ask questions about patients' experience (P00C22)<br>• Clarify reasons for requesting MAiD (P00C22)	• Timeframe to determine foreseeability of death not identified	• Requirement for exploring alternative treatment options not described
Ontario - *Greater Toronto Area*	• Calculation of 90-day waiting period not identified	• Reduction of 90-day waiting period not identified	• Provide options to relieve suffering (P00C24)<br>• Inform assessors decision-making process (P00C24)	• Review medical records (P00C24)<br>• Ask questions related to suffering (P00C24)<br>• Confirm medical conditions impact on quality of life (P00C24)<br>• Ask questions about patients' experience (P00C24)	• Timeframe to determine foreseeability of death not identified	• Yes<br>○ Assessors suggest treatment options (P00C24)
Ontario - *Waterloo area*	• Calculation of 90-day waiting period not identified	• Reduction of 90-day waiting period not identified	• Provide options to relieve suffering (P00C25)	• Review medical records (P00C25)<br>• Review specialists consult notes and treatments explored (P00C25)<br>• Clarify reasons for requesting MAiD (P00C25)<br>• Confirm impact of medical conditions on quality of life (P00C25)	• Death within 5 years (P00C25)<br>○ Relies on nomogram calculators to back up assessors' judgement (P00C25)	• Yes<br>○ Treatment options depend on patients' condition (P00C25)<br>○ Given consideration to treatment options (P00C25)
Ontario - *Noelville, Sudbury, Elliot Lake, Sturgeon Falls area*	• Calculation of 90-day waiting period not identified	• Reduction of 90-day waiting period not identified	• Explore options to relieve suffering (P00C26)	• Review medical records (P00C26)<br>• Review specialists consult notes (P00C26)<br>• Clarify reasons for requesting MAiD (P00C26)<br>• Ask questions about patients' experience (P00C26)<br>• Conduct follow up assessments (P00C26)	• Timeframe to determine foreseeability of death not identified	• Yes<br>○ Explore means to relieve suffering (P00C26)<br>○ Assessors review treatments the patient has undergone or declined (P00C26)
Québec - *Ministry of Health and Social Services*	• Calculation of 90-day waiting period not identified	• Reduction of 90-day waiting period not identified	• Inform assessors decision-making process (103)	• Review medical records (103)<br>• Review specialist consult notes (103)<br>• Rely on their expertise (103)	• Timeframe to determine foreseeability of death not identified	• Requirement for exploring alternative treatment options not described
INTEGRATED HEALTH AND SOCIAL SERVICES CENTRES						
Québec – *CISS Montérégie*	• Calculation of 90-day waiting period not identified	• Reduction of 90-day waiting period not identified	• Inform assessors decision-making process (P00C27)	• Review medical records (P00C27)<br>• Review specialist consult notes (P00C27)<br>• Rely on their expertise (P00C27)	• Timeframe to determine foreseeability of death not identified	• Requirement for exploring alternative treatment options not described
INTEGRATED UNIVERSITY HEALTH AND SOCIAL SERVICES CENTRES						
Québec – *CIUSS Capitale-Nationale*	• From date first assessment begins to provision day (110)	• Yes<br>○ If both assessors agree patient is at risk of losing capacity (110)	• Provide options to relieve suffering (110)<br>• Inform assessors decision-making process (110)	• Review specialists consult notes and treatment explored (110)<br>• Rely on their expertise (110)<br>• Clarify reasons for requesting MAiD (110)	• Timeframe to determine foreseeability of death not identified	• Yes<br>○ Explore means to relieve suffering (110)<br>○ Ensures patient have given consideration to treatment options (110)
Québec – *University of Montreal Hospital Center*	• From date first assessment begins to provision day (111)	• Reduction of 90-day waiting period not identified	• Inform assessors decision-making process (P00C40)	• Review medical records (P00C40)<br>• Review specialist consult notes (P00C40)<br>• Rely on their expertise (P00C40)	• Timeframe to determine foreseeability of death not identified	• Requirement for exploring alternative treatment options not described

Jurisdiction	Common elements within definitions					
	How is the 90-day waiting period calculated?	Can the 90-day waiting period be reduced?	What is the role of specialist consult?	How do assessors determine suffering?	What is timeframe to determine foreseeability of death?	Is there a requirement for the assessors to explore alternative treatment options with the patient?
Québec – *McGill University Health Centre*	• Calculation of 90-day waiting period not identified	• Reduction of 90-day waiting period not identified	• Inform assessors decision-making process (P00C9)	• Review medical records (P00C9)<br>• Review specialists consult notes (P00C9)<br>• Rely on their expertise (P00C9)	• Death can take more than 18 months (P00C9)	• Yes<br>○ Ensures patient have given consideration to treatment options (P00C9)

Table 11: Assessment process

Jurisdiction	When do assessors conduct assessments?	Are the assessment forms used standardized?	Is it mandatory for one assessor to be the provider?	How does assessors resolve their disagreement on patients' eligibility?	What is the possible mode of assessments?
Alberta - *Alberta Health Services*	• Assessors and patient determine a time (P00C39)	• Yes<br>○ Provincially standardized (P00C6, P00C13, P00C39)	• Yes<br>○ One assessor provides MAiD (P00C6, P00B13)<br>▪ If this is not possible, provider completes an assessment prior to provision (P00C6, P00B13)	• Procedure described as follows:<br>1. Coordinator sets up meeting with disagreeing assessors (P00A5, P00C12)<br>2. Assessors discuss case and adjust assessments as necessary (P00A5, P00B9, P00C39, P00C12)<br>3. If disagreement persists, seek third assessment (P00A5, P00B9, P00C39)<br>○ Third assessor is not made aware by coordinators that this is a third assessment (P00B9, P00C12)	• In person (P00C39, P00C10)<br>• Virtual (P00C6, P00B9, P00B13)
British Columbia - *Ministry of Health*	• Not applicable<br>○ No involvement in service delivery	• Yes<br>○ Provincially standardized<br>▪ HLTH 1634 Form - "Prescriber" (78, 112)<br>▪ HLTH 1633 Form - "Assessor" (78, 112)	• Yes<br>○ One assessor provides MAiD (P00A4)	• Not applicable<br>○ Not involved in service delivery	• In person (P00A4)<br>• Virtual (P00A4)
British Columbia - *Fraser Health*	• Assessors and patient determine a time (P00A10, P00C17)	• Yes<br>○ Provincially standardized<br>▪ HLTH 1634 Form - "Prescriber" (78, 112)<br>▪ HLTH 1633 Form - "Assessor" (78, 112)	• Yes<br>○ One assessor provides MAiD (28)	• Procedure described as follows:<br>1. Coordinator clarifies eligibility assessment (P00A10)<br>2. If disagreement persists, Medical Director enters discussion (P00A10)<br>3. If disagreement persists or discussion with medical director is refused, seek third assessment (P00A10)	• In person (P00A10)<br>• Virtual (P00A10, P00C17)
British Columbia - *Interior Health*	• Assesors and patient determine a time (P00B14)	• Yes<br>○ Provincially standardized<br>▪ HLTH 1634 Form - "Prescriber" (78, 112)<br>▪ HLTH 1633 Form - "Assessor" (78, 112)	• Yes<br>○ One assessor provides MAiD (P00B14)	• No procedure described<br>• Process involved in resolving disagreement may include<br>○ Assessors discuss case (39) (P00C29)<br>○ Seek third assessment (39) (P00C29)	• In person (P00C29)
British Columbia - *Island Health*	• Assesors and patient determine a time (P00A9)	• Yes<br>○ Provincially standardized<br>▪ HLTH 1634 Form - "Prescriber" (78, 112)<br>▪ HLTH 1633 Form - "Assessor" (78, 112)	• Yes<br>○ One assessor provides MAiD (P00A9)	• No procedure described<br>• Process involved in resolving disagreement may include<br>○ Assessors discuss case (P00A9)<br>○ Consult Medical Lead (P00A9, P00C13)<br>○ Seek third assessment (P00C13)<br>○ Seek guidance from CAMAP (P00A9)	• In person (P00A9)<br>• Virtual (P00A9)
British Columbia - *Northern Health*	• Timeframe for conducting assessments not specified	• Yes<br>○ Provincially standardized<br>▪ HLTH 1634 Form - "Prescriber (78, 112)<br>▪ HLTH 1633 Form – "Assessor" (78, 112)	• Mandate for one assessor to be provider not specified	• No procedure described<br>• Process involved in resolving disagreement may include<br>○ Seek third assessment (P00A8)	• In person (P00A8)
British Columbia - *Vancouver Coastal Health*	• Assessors and patient determine a time (P00B12)	• Yes<br>○ Provincially standardized<br>▪ HLTH 1634 Form - "Prescriber" (78, 112)<br>▪ HLTH 1633 Form - "Assessor" (78, 112)	• Yes<br>○ One assessor provides MAiD (P00B15)	• Procedure described as follows:<br>1.Assessors discuss case (P00B10, P00B12)<br>2.If disagreement persists, seek third assessment (38, 40) (P00A7, P00B10, P00B12)	• In person<br>• Virtual (telehealth/telemedicine (38-40) (P00A7, P00B10)
British Columbia - *Provincial Health Services Authority*	• Timeframe for conducting assessments not described	• Yes<br>○ Provincially standardized<br>▪ HLTH 1634 Form - "Prescriber" (78, 112)<br>▪ HLTH 1633 Form - "Assessor" (78, 112)	• Yes<br>○ One assessor provides MAiD (P00A11)	• Approach to resolve disagreement on patients' eligibilty not specified	• In person<br>• Hospital (P00A11)<br>• Home (P00A11)
Manitoba - *Shared Health*	• Assessors and patient determine a time (P00C34)<br>○ Assessors provide weekly availability to MAiD team (P00C34)	• Yes<br>○ Provincially standardized (P00C34, P00B19, P00B21)	• Yes<br>○ One assessor provides MAiD (P00C33)	• Procedure described as follows:<br>1.Assessors discuss case (P00B19, P00C33)<br>2.If disagreement persists, seek third assessment (P00C33, P00B21, P00B19)	• In person (P00C34)<br>• Virtual (telehealth) (P00C33, P00B19)
New Brunswick - *Horizon Health Network*	• Assessors and patients determine a time (P00C15)	• Yes<br>○ Regionally standardized (P00C15)	• Yes<br>○ One assessor provides MAiD (P00C15, P00B11)	• Procedure described as follows:<br>1.Assessors discuss case (P00C15)<br>2.If disagreement persists, seek third assessment (P00C15)	• In person (P00C15)<br>• Virtual (113)

Jurisdiction	When do assessors conduct assessments?	Are the assessment forms used standardized?	Is it mandatory for one assessor to be the provider?	How does assessors resolve their disagreement on patients' eligibility?	What is the possible mode of assessments?
New Brunswick - *Vitalité Health Network*	• Assessors and patients determine a time (P00C48)	• Yes<br>○ Regionally standardized (P00C48)	• Yes<br>○ One assessor provides MAiD (P00C48)	• No procedure described<br>• Process involved in resolving disagreement may include<br>○ Assessors discuss case (P00C48)	• In person (P00C48)<br>• Virtual (P00C48)
Newfoundland and Labrador - *Department of Health and Community Services*	• Not applicable<br>○ No involvement in service delivery	• Not applicable<br>○ No involvement in service delivery	• Not applicable<br>○ No involvement in service delivery	• Not applicable<br>○ No involvement in service delivery	• Not applicable<br>○ No involvement in service delivery
Newfoundland and Labrador - *Eastern Zone*	• Determined by MAiD team (P00C43)<br>○ Depends on assessor workload (P00C43)	• Yes<br>○ Regionally standardized (114) (P00C43)	• Yes<br>○ First assessor provides MAiD (114) (P00C43)	• Approach to resolve disagreement on patients' eligibility not specified	• In person (P00C43)<br>• Virtual (P00C43)
Newfoundland and Labrador - *Western Zone*	• Assessors and patient determine a time (P00C44)	• Yes<br>○ Regionally standardized (115)	• Yes<br>○ First assessor provides MAiD (P00C44)	• No procedure described<br>• Process involved in resolving disagreement may include<br>○ Seek third assessment (P00C44)	• In person (P00C44)<br>○ First assessment (P00C44)<br>• Virtual (P00C44)<br>○ First assessment (P00C44)<br>○ Second assessment (P00C44)
Newfoundland and Labrador - *Central Zone*	• Assessors and coordinator determine a time (P00B22)	• Yes<br>○ Regionally standardized (116) (P00B22)	• Yes<br>○ First assessor provides MAiD (116) (P00B22)	• Procedure described as follows:<br>1.Assessors discuss case (P00B22)<br>2.If disagreement persists, seek third assessment (P00B22)	• In person (P00B22)<br>• Virtual (P00B22)
Northwest Territories	• Assessors and patient determine a time (P00A2, P00C35, P00B24)	• Yes<br>○ Territorially standardized (11)	• No<br>○ A practitioner other than assessor can provide MAiD (P00A3)	• Procedure described as follows:<br>○ Seek third assessment (P00B24)	• In person (P00A4)<br>• Virtual (P00A2, P00A3) (117)
Nova Scotia - *Nova Scotia Health*	• Assessors and patient determine a time (52)	• Yes<br>○ Provincially standardized (118) (P00A6, P00C23)	• Yes<br>○ One assessor provides MAiD (P00A12)	• No procedure described<br>• Process involved in resolving disagreement may include<br>○ Assessors discuss case (P00C38)<br>○ Seek third assessment (P00A6)	• In person (P00A12)
Prince Edward Island - *Health PEI*	• During specific hours (P00B17)<br>○ Appointment scheduled with clinic assessors (P00B17)<br>• Determined by MAiD team (P00B17)<br>○ Subject to patient location (P00B8)	• Yes<br>○ Provincially standardized (P00B17)	• Yes<br>○ One assessor provides MAiD (P00B17)	• No procedures described<br>• Process involved in resolving disagreement may include<br>○ Assessors discuss case (P00B8, P00B17)<br>○ Seek third assessment (P00B8, P00B17)<br>○ Review case with clinical advisory committee (P00B8)<br>○ Conduct complex case review with other MAiD providers and staff (P00B8)	• In person (P00B8, P00B17)<br>• Virtual (P00B8, P00B17)
Saskatchewan - *Saskatchewan Health Authority*	• Assessors and patient determine a time (P00A1)	• Yes<br>○ Provincially standardized (P00A1, P00C1)	• No<br>○ One assessor does not have to provide MAiD (P00A1)	• Procedure described as follows:<br>1.Assessors discuss case (P00A1)<br>2.Medical Director could be consulted (P00A1)<br>3.If disagreement persists, seek third assessment (P00A1, P00B1, P00B3, P00C1)<br>○ Third assessor blinded to previous assessments but knows that they are the third assessor (P00A1)	• In person (P00C19)<br>• Virtual (P00A1)
Yukon - *Department of Health and Social Services*	• Assessors and patient determine a time (P00C14)	• Yes<br>○ Territorially standardized (119) (P00C14)<br>▪ "Most Responsible Medical Practitioner for MAiD Patient Safeguards Administration" " (120, 121)<br>▪ "The Secondary Assessor Record for MAiD" (120, 121)	• Yes<br>○ One assessor provides MAiD (P00C7, P00C14)	• No procedures described<br>• Process involved in resolving disagreement may include<br>○ Assessors discuss case (P00C7, P00C14)<br>○ Seek third assessment (P00C7, P00C14)	• In person (P00C7)
Ontario - *Ministry of Health*	• Not applicable<br>○ No involvement in service delivery	• Yes<br>○ Voluntary provincially standardized<br>▪ Clinician Aid A (P00B4)<br>▪ Clinician Aid B (P00B4)<br>▪ Clinician Aid C (P00B4)	• Not applicable<br>○ No involvement in service delivery	• Not applicable<br>○ No involvement in service delivery	• Not applicable<br>○ No involvement in service delivery
SERVICE ORGANIZATIONS/REGIONAL FACILITIES					

Jurisdiction	When do assessors conduct assessments?	Are the assessment forms used standardized?	Is it mandatory for one assessor to be the provider?	How does assessors resolve their disagreement on patients' eligibility?	What is the possible mode of assessments?
Ontario - *Home and Community Care Support Services, Central East*	• Assessors and patient determine a time (P00C2)	• Yes<br>○ Provincially standardized<br>▪ Clinician Aid A (P00C2)<br>▪ Clinician Aid B (P00C2)<br>▪ Clinician Aid C (P00C2)<br>○ Institutional standardized<br>▪ Documentation form (P00C2)	• Yes<br>○ One assessor provides MAiD (P00C2)	• Procedure described as follows:<br>1. Notify care coordination service of disagreement (P00C2)<br>2. Regional care coordination service seeks third assessment (P00C2)	• In person (P00C2)
Ontario - *Home and Community Care Support Services, Waterloo Region*	• Assessors and patient determine a time (P00C4)	• Yes<br>○ Insitutionally standardized (P00C4)	• Yes<br>○ One assessor provides MAiD (P00C4)<br>▪ If this is not possible, provider completes an assessment prior to provision (P00C4)	• Procedure described as follows:<br>1. Assessors discuss case (P00C4)<br>2. If disagreement persists, seek third assessment (P00C4)	• In person (P00C4)
Ontario - *Home and Community Care Support Services, South East*	• Assessors and patient determine a time (P00C20)	• No<br>○ Assessors develop their own forms (P00C20)<br>▪ Can utilize provincial forms	• Yes<br>○ One assessor provides MAiD (P00C20)<br>▪ If this is not possible, provider completes an assessment prior to provision (P00C20)	• No procedure described<br>• Process involved in resolving disagreement may include<br>○ Seek third assessment (P00C20)	• In person (P00C20)<br>• Virtual (P00C20)
Ontario - *Home and Community Care Support Services, South West*	• Determined by MAiD team (P00C23)<br>○ Coordinating with assessors about time (P00C23)	• No<br>○ Assessors develop their own forms (P00C23)	• Yes<br>○ One assessor provides MAiD (P00C23)	• No procedures described<br>• Process involved in resolving disagreement may include<br>○ Assessors do not discuss cases (P00C23)<br>○ Seek third assessment (P00C23)	• In person (P00C23)<br>• Virtual (P00C23)
Ontario - *Champlain Regional MAiD Network, the Ottawa Hospital*	• Determined by MAiD team (P00C5)<br>○ Considers patient and assessors preferences (P00C5)	• Yes<br>○ Provincially standardized<br>▪ Clinician Aid A (P00C5)<br>▪ Clinician Aid B (P00C5)<br>▪ Clinician Aid C (P00C5)	• Yes<br>○ One assessor provides MAiD (P00C5)	• Procedure described as follows:<br>1. Assessors discuss case (P00C5)<br>2. If disagreement persists, seek third assessment (P00C5)	• In person (P00C5)
REGIONAL HEALTHCARE FACILITIES					
Ontario - *Peterborough Regional Health Centre*	• During specific hours (P00C3)<br>• Assessors and patient determine a time (P00C3)	• Yes<br>○ Insitutionally standardized<br>▪ Individual assesment templates, which have standarized form elements (P00C3)	• Yes<br>○ One assessor provides MAiD (P00C3)	• No procedure described<br>• Process involved in resolving disagreement may include<br>○ Seek third assessment (P00C3)	• In person (P00C3)
Ontario - *Mount Sinai Healthcare Facility, Toronto*	• Determined by MAiD team (P00C36)<br>○ Coordinating with assessors about time (P00C36)	• Yes<br>○ Provincially standardized (P00C36)	• Mandate for one assessor to be provider not specified	• Procedure described as follows:<br>1. Assessors discuss case (P00C36)<br>2. If disagreement persists, seek third assessment (P00C36)	• In person (P00C36)
Ontario - *University Health Network*	• Determined by MAiD team (P00C8)<br>○ Considers patient and assessors preferences (P00C8)	• Yes<br>○ Institutionally standardized<br>▪ Adapted and aligned with provincial forms (P00C8)	• Yes<br>○ One assessor provides MAiD (P00C8)	• No procedure described<br>• Process involved in resolving disagreement may include<br>○ Seek third assessment (P00C8)	• In person (P00C8)
Ontario - *Grand River Hospital*	• Determined by MAiD team (P00C50)<br>○ Coordinating with assessors about time (P00C50)	• Yes<br>○ Provincially standardized (P00C50)	• Yes<br>○ One assessor provides MAiD (P00C50)	• Approach to resolve disagreement on patients' eligibility not specified	• In person (P00C50)
COMMUNITY OF PRACTICE					
Ontario - *Hamilton Family Health Team*	• The assessor, patient, and coordinator determine a time (P00C32)	• Yes<br>○ Regionally standardized (P00C32)	• Yes<br>○ One assessor provides MAiD (P00C32)	• Procedure described as follows:<br>1. Assessors discuss case (P00C32)<br>2. If disagreement persists, seek third assessment (P00C32)<br>3. If third assessor deems patient ineligible, patient referred back to provincial care coordination service (P00C32)	• In person (P00C32)

Jurisdiction	When do assessors conduct assessments?	Are the assessment forms used standardized?	Is it mandatory for one assessor to be the provider?	How does assessors resolve their disagreement on patients' eligibility?	What is the possible mode of assessments?
Ontario - *Niagara Community MAiD Team, St. Catherine's, Niagara*	• Assessors and patient determine a time (P00C11)	• Yes<br>○ Provincially standardized<br>▪ Clinician Aid A (P00C11)<br>▪ Clinicians Aid C (P00C11)	• Yes<br>○ One assessor provides MAiD (P00C11)<br>▪ If this is not possible, provider completes assessment prior to provision (P00C11)	• No procedure described<br>• Process involved in resolving disagreement may include<br>○ Seek third assessment (P00C11)	• In person (P00C11)<br>• Virtual (P00C11)
GEOGRAPHICAL AREAS					
Ontario - *Oakville & Mississauga area*	• Assessors and patient determine a time (P00C18)	• Yes<br>○ Provincially standardized<br>▪ Clinician Aid A (P00C18)<br>▪ Clinician Aid B (P00C18)<br>▪ Clinician Aid C (P00C18)	• Yes<br>○ First assessors (primary assessor) provides MAiD (P00C18)	• No procedure described<br>• Process involved in resolving disagreement may include<br>○ Assessors discuss case (P00C18)<br>○ Seek third assessment (P00C18)	• In person (P00C18)
Ontario - *Oakville, Mississauga, & Burlington area*	• Assessors and patient determine a time (P00C21)	• Yes<br>○ Provincially standardized<br>▪ Clinician Aid A (P00C21)<br>▪ Clinician Aid B (P00C21)<br>▪ Clinician Aid C (P00C21)	• Mandate for one assessor to be provider not specified	• No procedure described<br>• Process involved in resolving disagreement may include<br>○ Assessors discuss case (P00C21)<br>○ Seek third assessment (P00C21)	• In person (P00C21)
Ontario - *Renfrew County*	• Assessors and patient determine a time (P00C22)	• Yes<br>○ Provincially standardized<br>▪ Clinician Aid A (P00C22)	• Yes<br>○ One assessor provides MAiD (P00C22)	• No procedure described<br>• Process involved in resolving disagreement may include<br>○ Assessors discuss case (P00C22)	• In person (P00C22)
Ontario - *Greater Toronto Area*	• During specific hours (P00C24)<br>• Appointment is scheduled with clinic assessors (P00C24)	• Yes<br>○ Provincially standardized (P00C24)	• No (P00C24)	• Procedure described as follows:<br>1. Assessors discuss case (P00C24)<br>2. If disagreement persists, seek third assessment (P00C24)	• In person (P00C24)<br>• Virtual (P00C24)
Ontario - *Waterloo area*	• Assessors and patient determine a time (P00C25)	• Yes<br>○ Provincially standardized (P00C25)	• Mandate for one assessor to be provider not specified	• No procedures described<br>• Process involved in resolving disagreement may include<br>○ Assessors discuss case (P00C25)	• In person (P00C25)<br>• Virtual (P00C25)
Ontario - *Noelville, Sudbury, Elliot Lake, Sturgeon Falls area*	• Assessors and patient determine a time (P00C26)	• Yes<br>○ Provincially standardized (P00C26)	• Mandate for one assessor to be provider not specified	• Procedure described as follows:<br>1. Assessors discuss case (P00C26)<br>2. If disagreement persists, seek third assessment (P00C26)	• In person (P00C26)<br>• Virtual (P00C26)
Québec - *Ministry of Health and Social Services*	• Not applicable<br>○ No involvement in service delivery	• Yes<br>○ Provincially standardized (P00C9, P00C27, P00C37, P00C40)<br>▪ Electronic medical record (P00C9, P00C27, P00C37, P00C40)	• Not applicable<br>○ No involvement in service delivery	• Not applicable<br>○ No involvement in service delivery	• Not applicable<br>○ No involvement in service delivery
INTEGRATED HEALTH AND SOCIAL SERVICES CENTRES					
Québec – *CISS Montérégie*	• Assessors and patient determine a time (P00C27)	• Yes<br>○ Provincially standardized (P00C27)<br>▪ Electronic medical record (P00C27)	• Yes<br>○ One assessor provides MAiD (P00C27)	• MAiD request is cancelled (P00C27)<br>○ Patient can initiate another request (P00C27)	• In person (P00C27)
INTEGRATED UNIVERSITY HEALTH AND SOCIAL SERVICES CENTRES					
Québec – *CIUSS Capitale-Nationale*	• Assessors and patient determine a time (P00C37)	• Yes<br>○ Provincially standardized (P00C37)<br>▪ Electronic medical record (P00C37)	• Yes<br>○ One assessor provides MAiD (P00C37)	• No process described<br>• Process involved in resolving disagreement may include<br>○ Assessors discuss case (P00C37)	• In person (P00C37)
Québec – *University of Montreal Hospital Center*	• Assessors and patient determine a time (P00C40)	• Yes<br>○ Provincially standardized (P00C40)<br>▪ Electronic medical record (P00C40)	• Yes<br>○ One assessor provides MAiD (P00C40)	• No procedure described<br>• Process involved in resolving disagreement may include<br>○ Assessors discuss case (P00C40)	• In person (P00C40)
Québec – *McGill University Health Centre*	• Assessors and patient determine a time (P00C9)	• Yes<br>○ Provincially standardized (P00C9)<br>▪ Electronic medical record (P00C9)	• Yes<br>○ One assessor provides MAiD (P00C9)	• No procedure described<br>• Process involved in resolving disagreement may include<br>○ Assesors discuss case (P00C9)<br>○ Seek third assessment (P00C9)	• In person (P00C9)

Table 12: Assessors and Providers background

Jurisdiction	What processes are used to assess suitability of potential assessors/providers?	What training processes do assessors/providers receive?	Who completes assessments/provisions?	Who is authorized to complete assessments/provisions?
Alberta - *Alberta Health Services*	• Formal interviews (P00C6)<br>○ For Alberta Health Services affiliated physicians/nurse practitioners (P00A5)<br>• Privileged by Medical Affairs<br>○ For Alberta Health Services affiliated physicians/nurse practitioners (P00B13)	• Mandatory<br>○ Mentorship/Shadowing (P00C6, P00C12)<br>▪ For Alberta Health Services affiliated assessors and providers (P00A5)<br>○ Online module (P00B13)<br>▪ Module by Alberta Health Services (P00B13)<br>▪ For Alberta Health Services affiliated assessors and providers (P00A5)<br>• Training for community-based assessors and providers varies (P00A5)	• Physicians (P00C6, P00C13, P00C39)<br>• Nurse practitioners (P00C6, P00C13, P00C39)	• Physicians (1)<br>• Nurse practitioners (77)
British Columbia - *Ministry of Health*	• Not applicable<br>○ No involvement in service delivery	• Not applicable<br>○ No involvement in service delivery	• Not applicable<br>○ No involvement in service delivery	• Not applicable<br>○ No involvement in service delivery
British Columbia - *Fraser Health*	• Informal discussion with MAiD manager and/or MAiD Medical Director (P00A10)<br>○ Physicians<br>• Formal interview with the MAiD team (P00A10)<br>○ Nurse practitioners	• Mandatory<br>○ Mentorship/Shadowing (P00A10)<br>▪ For physicians (providers) and nurse practitioners (P00A10)<br>○ Online module<br>▪ Learning hub with PHSA (P00A10)<br>- For physicians (providers) and nurse practitioners (P00A10)<br>• No further education and training are provided to physicians (assessors) (P00A10)	• Physicians (28)<br>• Nurse practitioners (28)	• Physicians (28)<br>• Nurse practitioners (28)
British Columbia - *Interior Health*	• Formal conversation with Senior Medical Director, Palliative care and MAiD (P00B14)<br>• Privileged by healthcare facilities<br>○ For Acute care practitioners (P00B14)	• Mandatory<br>○ Mentorship/Shadowing (P00B14)<br>○ Online module<br>▪ Learning hub with PHSA (P00B14)<br>▪ Module by College of Physicians and Surgeons of British Columbia (P00C29)<br>▪ For hospital-based practitioners (P00C29)<br>○ Other<br>• Voluntary<br>○ Mentorship/Shadowing<br>▪ Community-based practitioners (P00C29)<br>○ CAMAP module<br>▪ Community-based practitioners (P00C29)	• Physicians (34)<br>• Nurse practitioners (34)	• Physicians (32, 33)<br>• Nurse practitioners (32, 33)
British Columbia - *Island Health*	• Informal conversation with Medical Director, MAiD (P00A9)<br>○ Physicians<br>• Privileged by Medical Lead (P00A9, P00A13)<br>○ For hospital based practitioners (P00A9)	• Mandatory<br>○ Mentorship/Shadowing (P00A9, P00A13)<br>▪ For Island Health affiliated physicians (P00A9, P00A13)<br>○ Online module<br>▪ Learning hub with PHSA (P00A13)<br>▪ For Island Health affiliated practitioners (P00A9, P00A13)<br>○ Other<br>▪ Nurse practitioner training program (P00A9)<br>• Voluntary<br>○ CAMAP module (P00A9, P00A13)<br>• Training for community based assessors and providers varies (122)	• Physicians (35, 36, 107)<br>• Nurse practitioners (35, 36, 107)	• Physicians (107)<br>• Nurse practitioners (107)
British Columbia - *Northern Health*	• Expression of interest (P00A8)<br>• Privileged by Medical Lead (P00A8)	• Voluntary<br>○ CAMAP modules (P00A8)<br>○ Education sessions (P00A8)	• Physicians (P00A8)	• Physician (P00A8)<br>• Nurse practitioners (P00A8) (41, 79, 123)
British Columbia - *Vancouver Coastal Health*	• Privileged by health authority (P00B10, P00B16)<br>○ Following approval from Medical Director, MAiD (P00B10)<br>• Informal conversation with Medical Director, MAiD (P00B10)	• Mandatory<br>○ Mentorship/Shadowing<br>▪ For physicians and nurse practitioners (P00A7, P00B16, P00B10)<br>○ Online modules (P00A7, P00B15, P00B10, P00B16)<br>▪ Learning hub with PHSA (P00A7)<br>- For physicians (providers) and nurse practitioners (P00A7)<br>○ Preceptorships	• Physicians (38)<br>• Nurse practitioners (38)	• Physicians (38)<br>• Nurse practitioners (38)

Jurisdiction	What processes are used to assess suitability of potential assessors/providers?	What training processes do assessors/providers receive?	Who completes assessments/provisions?	Who is authorized to complete assessments/provisions?
		▪ For nurse practitioners (P00B10)<br>• Voluntary<br>○ Online modules (P00A7, P00B15, P00B10, P00B16)<br>▪ Learning hub with PHSA (P00A7)<br>- For physicians (assessors) (P00A7)<br>○ CAMAP modules (P00A7, P00B15, P00B10, P00B16)		
British Columbia - *Provincial Health Services Authority*	• Credentialed with the PHSA (P00A11)	• Mandatory<br>○ Learning hub with PHSA (P00A11)	• Physicians (P00A11, P00C42)<br>• Nurse practitioners (P00A11)	• Physicians (P00A11, P00C42)<br>• Nurse practitioners (P00A11)
Manitoba - *Shared Health*	• Formal interview with Medical Director, MAiD (P00C33, P00C34)	• Mandatory<br>○ Mentorship/Shadowing (P00C33, P00C34)<br>○ MAiD Orientation (P00B19)	• Physicians (7)<br>• Nurse practitioners (7)	• Physicians (7)<br>• Nurse practitioners (7)
New Brunswick - *Horizon Health Network*	• Conversation with Coordinator (Regional lead) (P00B11)	• Voluntary<br>○ Mentorship/Shadowing (P00B11, P00C15)<br>○ CAMAP modules (P00C15)	• Physicians (9)<br>• Nurse practitioners (9, 124)	• Physicians (9)<br>• Nurse practitioners (9, 124)
New Brunswick - *Vitalité Health Network*	• No formal process (P00C48)	• Voluntary<br>○ Mentorship/Shadowing (P00C48)	• Physicians (P00C48)<br>• Nurse practitioners (P00C48)	• Physicians (P00C48)<br>• Nurse practitioners (P00C48)
Newfoundland and Labrador - *Department of Health and Community Services*	• Not applicable<br>○ No involvement in service delivery	• Not applicable<br>○ No involvement in service delivery	• Not applicable<br>○ No involvement in service delivery	• Not applicable<br>○ No involvement in service delivery
Newfoundland and Labrador - *Eastern Zone*	• Formal conversation with Medical Director, MAiD (P00C43)<br>• Licensed and credentialed by appropriate regulatory body (P00C43)	• Mandatory<br>○ Mentorship/Shadowing (P00C43)<br>○ Online module (P00C43)<br>▪ Learning hub with Memorial University (P00C44)	• Physicians (48)<br>• Nurse practitioners (48)	• Physicians (48)<br>• Nurse practitioners (48)
Newfoundland and Labrador - *Western Zone*	• Licensed and credentialed by appropriate regulatory body (P00C43)	• Mandatory<br>○ Mentorship/Shadowing (P00C43)<br>○ Online module (P00C44)<br>▪ Learning hub with Memorial University (P00C44)<br>• Voluntary<br>○ CAMAP modules (P00C44)	• Physicians (115)<br>• Nurse practitioners (115)	• Physicians (115)<br>• Nurse practitioners (115)
Newfoundland and Labrador - *Central Zone*	• Licensed and credentialed by appropriate regulatory body (P00C43)	• Mandatory<br>○ Mentorship/Shadowing (P00C43)<br>○ Online module (P00C44)<br>▪ Learning hub with Memorial University (P00C44)	• Physicians (P00B22)<br>• Nurse practitioners (P00B22)	• Physicians (P00B22)<br>• Nurse practitioners (P00B22)
Northwest Territories	• Expression of interest (P00A3)<br>○ Requisite skills and capacity to assess and provide MAiD (P00A2)	• Voluntary<br>○ Mentorship/Shadowing (P00A2, P00A3)<br>○ CAMAP modules (P00A2)	• Physicians (11)<br>• Nurse practitioners (11)	• Physicians (11)<br>• Nurse practitioners (11)
Nova Scotia - *Nova Scotia Health*	• Expression of interest (P00A12)<br>• Informal conversation with program lead (P00A12)	• Voluntary<br>○ Mentorship/Shadowing (P00A6, P00A12)<br>○ CAMAP modules (P00A12)<br>○ Training sessions (P00A12)	• Physicians (125) (P00A12)<br>• Nurse practitioners (14, 125) (P00A12)	• Physicians (125) (P00A12)<br>• Nurse practitioners (14, 125) (P00A12)
Prince Edward Island - *Health PEI*	• Expression of interest (P00B8)<br>• Informal conversation with Medical Lead (P00B17)<br>• Licensed by appropriate regulatory body (22)	• Voluntary<br>○ Mentorship/Shadowing (P00B8, P00B17)<br>○ CAMAP module (P00B8, P00B17)	• Physicians (22, 53)<br>• Nurse practitioners (22, 53)	• Physicians (22, 53)<br>• Nurse practitioners (22, 53)
Saskatchewan - *Saskatchewan Health Authority*	• Informal conversation with Medical Director, MAiD (P00A1, P00C19)<br>○ Interested practitioners sign health authority practitioner agreement (P00A1, P00B1, P00B5)<br>• Licensed and credentialed by appropriate regulatory body (P00A1, P00B5, P00B1)<br>○ Assessors and providers submit credentialing request to college	• Mandatory<br>○ Mentoring/shadowing (P00B3)<br>○ Training/on boarding process (P00B3)<br>• Voluntary<br>○ CAMAP modules (P00C1)	• Physicians (88, 126)<br>• Nurse practitioners (88)	• Physicians (88)<br>• Nurse practitioners (88)

Jurisdiction	What processes are used to assess suitability of potential assessors/providers?	What training processes do assessors/providers receive?	Who completes assessments/provisions?	Who is authorized to complete assessments/provisions?
	• Privileged through Practitioner Staff Affairs (P00B1) (58)<br>○ For physicians (P00B1)<br>• Privileged by health authority (P00A1)<br>○ Nurse practitioners (P00A1)			
Yukon - *Department of Health and Social Services*	• Compliance with standards and guidelines of appropriate regulatory bodies for nurse practitioners (P00C14)	• Voluntary<br>○ Mentorship/Shadowing (P00C7, P00C14)	• Physicians (109)<br>• Nurse practitioners (109)	• Physicians (109)<br>• Nurse practitioners (109)
Ontario - *Ministry of Health*	• Not applicable<br>○ No involvement in service delivery	• Not applicable<br>○ No involvement in service delivery	• Not applicable<br>○ No involvement in service delivery	• Not applicable<br>○ No involvement in service delivery
SERVICE ORGANIZATIONS/REGIONAL FACILITIES				
Ontario - *Home and Community Care Support Services, Central East*	• Expression of interest (P00C2)<br>• Priveleged by healthcare facilities (P00C2)<br>○ For hospital affiliated practitioners (P00C2)	• Mandatory<br>○ Online module<br>▪ Learning hub with University of Toronto (P00C2)<br>○ CAMAP module (P00C2)	• Physicians (P00C2)<br>• Nurse practitioners (P00C2)	• Physicians (59)<br>• Nurse practitioners (P00C2)
Ontario - *Home and Community Care Support Services, Waterloo Region*	• Expression of interest (P00C4)	• Mandatory<br>○ Mentorship/Shadowing (P00C4)<br>• Voluntary<br>○ Educational workshops (P00C4)<br>○ CAMAP module (P00C4)	• Physicians (P00C4)<br>• Nurse practitioners (P00C4)	• Physicians (P00C4)<br>• Nurse practitioners (59)
Ontario - *Home and Community Care Support Services, South East*	• Expression of interest (P00C20)	• No additional training required (P00C20)	• Physicians (P00C20)<br>• Nurse practitioners (P00C20)	• Physicians (P00C20)<br>• Nurse practitioners (P00C20)
Ontario - *Home and Community Care Support Services, South West*	• Expression of interest (P00C23)	• Mandatory<br>○ Mentorship/Shadowing (P00C23)<br>○ CAMAP module (P00C23)<br>○ Education sessions (P00C23)	• Physicians (P00C23<br>• Nurse practitioners (P00C23)	• Physicians (P00C23)<br>• Nurse practitioners (P00C23)
Ontario - *Champlain Regional MAiD Network, the Ottawa Hospital*	• Expression of interest (P00C5)	• Mandatory<br>○ Mentorship/Shadowing (P00C5)<br>○ Education session (P00C5)<br>• Voluntary<br>○ CAMAP module (P00C5)	• Physicians (P00C5)<br>• Nurse practitioners (P00C5)	• Physicians (P00C5)<br>• Nurse practitioners (P00C5)
HEALTHCARE FACILITIES				
Ontario - *Peterborough Regional Health Centre*	• Hospital privileges (P00C3)	• Mandatory<br>○ Mentorship/Shadowing (P00C3)<br>○ Education sessions (P00C3)	• Physicians (P00C3)<br>• Nurse practitioners (P00C3)	• Physicians (P00C3)<br>• Nurse practitioners (P00C3)
Ontario - *Mount Sinai Healthcare Facility, Toronto*	• Confirm assessors have capacity and confidence to participate in MAiD (P00C36)	• Voluntary<br>○ Mentorship/Shadowing (P00C36)	• Physicians (P00C36)<br>• Nurse practitioners (P00C36)	• Physicians (P00C36)<br>• Nurse practitioners (59)
Ontario - *University Health Network*	• Expression of interest (P00C8)<br>• Confirm assessors/providers are capacity of fulfilling legal responsibilities (P00C8)	• Mandatory<br>○ Mentorship/Shadowing (P00C8)<br>○ CAMAP modules (P00C8)	• Physicians (P00C8)<br>• Nurse practitioners (P00C8)	• Physicians (P00C8)<br>• Nurse practitioners (P00C8)
Ontario - *Grand River Hospital*	• Informal discussion with MAiD coordinator (P00C50)<br>• Credentials checked by Chief of Staff (P00C50)	• Voluntary<br>○ Mentorship/Shadowing (P00C50)<br>○ CAMAP modules (P00C50)	• Physicians (P00C50)<br>• Nurse practitioners (P00C50)	• Physicians (P00C50)<br>• Nurse practitioners (P00C50)
COMMUNITY OF PRACTICE				
Ontario - *Hamilton Family Health Team*	• Process to assess suitability not specified	• Mandatory<br>○ Mentorship/Shadowing (P00B18)<br>○ CAMAP Module (P00B18)	• Physicians (P00B18)<br>• Nurse practitioners (P00B18)	• Physicians (P00B18)<br>• Nurse practitioners (59)
Ontario - *Niagara Community MAiD Team, St. Catherine's, Niagara*	• Process to assess suitability not specified	• Voluntary<br>○ Mentorship/Shadowing (P00C11)	• Physicians (P00C11)<br>• Nurse practitioners (P00C11)	• Physicians (59)<br>• Nurse practitioners (P00C11)
GEOGRAPHICAL AREAS				
Ontario - *Oakville & Mississauga area*	• Process to assess suitability not specified	• Voluntary	• Physicians (P00C18)	• Physicians (P00C18)

Jurisdiction	What processes are used to assess suitability of potential assessors/providers?	What training processes do assessors/providers receive?	Who completes assessments/provisions?	Who is authorized to complete assessments/provisions?
		○ Mentorship/Shadowing (P00C18)	• Nurse practitioners (P00C18)	• Nurse practitioners (P00C18)
Ontario - *Oakville, Mississauga, and Burlington area*	• Expression of interest (P00C21)	• Voluntary<br>○ Mentorship/Shadowing (P00C21)<br>○ CAMAP module (P00C21)	• Physicians (P00C21)<br>• Nurse practitioners (P00C21)	• Physicians (P00C21)<br>• Nurse practitioners (P00C21)
Ontario - *Renfrew County*	• Process to assess suitability not specified	• Voluntary<br>○ Mentorship/Shadowing (P00C22)	• Physicians (P00C22)<br>• Nurse practitioners (P00C22)	• Physicians (P00C22)<br>• Nurse practitioners (59)
Ontario - *Greater Toronto Area*	• Process to assess suitability not specified	• Voluntary<br>○ CAMAP module (P00C24)	• Physicians (P00C24)<br>• Nurse practitioners (P00C24)	• Physicians (P00C24)<br>• Nurse practitioners (59)
Ontario - *Waterloo area*	• Expression of interest (P00C25)<br>○ Confirm assessors are comfortable and capable of fulfilling legal responsibilities (P00C25)	• Voluntary<br>○ Mentorship/Shadowing (P00C25)	• Physicians (P00C25)<br>• Nurse practitioners (P00C25)	• Physicians (P00C25)<br>• Nurse practitioners (P00C25)
Ontario - *Noelville, Sudbury, Elliot Lake, Sturgeon Falls area*	• Process to assess suitability not specified	• No specific onboarding training identified	• Physicians (P00C26)<br>• Nurse practitioners (P00C26)	• Physicians (P00C26)<br>• Nurse practitioners (P00C26)
Québec - *Ministry of Health and Social Services*	• Not applicable<br>○ No involvement in service delivery	• Not applicable<br>○ No involvement in service delivery	• Not applicable<br>○ No involvement in service delivery	• Not applicable<br>○ No involvement in service delivery
INTEGRATED HEALTH AND SOCIAL SERVICES CENTRES				
Québec – *CISS Montérégie*	• Process to assess suitability not specified	• Mandatory<br>○ Mentorship/Shadowing (P00C27)<br>• Voluntary<br>○ CAMAP (P00C27)	• Physicians (P00C27)<br>• Nurse practitioners (127)	• Physicians (P00C27)<br>• Nurse practitioners (127)
INTEGRATED UNIVERSITY HEALTH AND SOCIAL SERVICES CENTRES				
Québec – *CIUSS Capitale-Nationale*	• Licensed and credentialed by appropriate regulatory body (73)	• Mandatory<br>○ Mentorship/Shadowing (P00C37)	• Physicians (P00C37)<br>• Nurse practitioners (127)	• Physicians (P00C37)<br>• Nurse practitioners (127)
Québec – *University of Montreal Hospital Center*	• Process to assess suitability not specified	• No specific onboarding training identified	• Physicians (P00C40)<br>• Nurse practitioners (127)	• Physicians (P00C40)<br>• Nurse practitioners (127)
Québec – *McGill University Health Centre*	• Hospital privileges (P00C9)	• Mandatory<br>○ Mentorship/Shadowing (P00C9)	• Physicians (P00C9)<br>• Nurse practitioners (127)	• Physicians (P00C9)<br>• Nurse practitioners (127)

Table 13: Independence of assessment

Jurisdiction	Do assessors discuss the case with each other before determining patient's eligibility?	What information does the two assessors share with each other before determining patients' eligibility?	Who assigns the first assessor?	Who assigns the second assessor?	What additional strategies are in place to ensure independence of assessment?
Alberta - *Alberta Health Services*	• No<br>○ Unless one assessor is a specialist (P00C12)	• Patients' condition only if one assessor is a relevant specialist (P00C12)	• Provincial care coordination team (P00C12)<br>• Individual physicians/nurse practitioners (P00A5)	• Provincial care coordination team (P00C12)<br>• First assessor (P00A5)	• No contact between assessors prior to assessment (P00C12)<br>• Assessment findings must not be stored in electronic healthcare network (P00A5)
British Columbia - *Ministry of Health*	• Not applicable<br>○ No involvement in service delivery	• Not applicable<br>○ No involvement in service delivery	• Not applicable<br>○ No involvement in service delivery	• Not applicable<br>○ No involvement in service delivery	• Not applicable<br>○ No involvement in service delivery
British Columbia - *Fraser Health*	• No (P00A10)	• Assessment findings (P00A10)	• Regional care coordination team (P00A4, P00A10, P00C17)	• Regional care coordination team (P00A4, P00A10, P00C17)	• Assessment must be conducted at different times (not together) (P00A10)
British Columbia - *Interior Health*	• Yes (P00B14, P00C29, P00C41)	• Information shared not specified (P00C29)	• Regional care coordination team (P00A4, P00C17)<br>• Individual physicians/nurse practitioners (P00A4)	• Regional care coordination team (P00A4, P00C17)<br>• First assessor (P00A4)	• No additional strategies identified
British Columbia - *Island Health*	• Yes (P00C13)<br>○ Depends on the assessor practice (P00A9)	• Assessment findings (P00C13)	• Regional care coordination team (P00A9)<br>• Individual physicians/nurse practitioners (P00A9)	• Regional care coordination team (P00A9)<br>• First assessor (P00A9)	• No additional strategies identified
British Columbia - *Northern Health*	• Discussion between assessors before determining eligibility not specified	• Assessment findings (P00A8)	• Regional care coordination team (P00A8)<br>• Individual physicians/nurse practitioners (P00A8)	• Regional care coordination team (P00A8)<br>• First assessor (P00A8)	• Depends on assessors (P00A8)
British Columbia - *Vancouver Coastal Health*	• Yes (P00A7, P00B12, P00B10)	• Assessment findings (P00B10)	• Regional care coordination team (P00C17, P00A7)<br>• Individual physicians/nurse practitioners (P00A7)	• Regional care coordination team (P00C17, P00A7)<br>• First assessor (P00A7)	• Depends on assessors (P00A7)<br>○ No contact between assessors prior to assessment (P00A7)
British Columbia - *Provincial Health Services Authority*	• Discussion between assessors before determining eligibility not specified	• Information shared not specified	• Regional care coordination team (P00A11)	• Regional care coordination team (P00A11)	• Methods to ensure independence of assessments not specified
Manitoba - *Shared Health*	• No (P00C34)<br>○ Exceptional cases exist (P00C34)	• Assessment findings (P00C34)	• Provincial care coordination team (P00C33, P00C34)	• Provincial care coordination team (P00C33, P00C34)	• No contact between assessors prior to assessment (128) (P00C34)<br>○ Exceptional cases exist (P00C34)
New Brunswick - *Horizon Health Network*	• No<br>○ Exceptional cases exist (P00B11)	• Assessment findings (in exceptional cases) (P00B11, P00C15)	• Regional care coordination team (P00B11)<br>• Individual physicians/nurse practitioners (P00C15)	• Regional care coordination team (P00B11)<br>• First assessor (P00C15)	• No contact between assessors prior to assessment<br>○ Exceptional cases exist (P00B11)
New Brunswick - *Vitalité Health Network*	• No (P00C48)	• Patients' medical chart (P00C48)	• Individual physicians/nurse practitioners (P00C48)	• First assessor (P00C48)	• No additional strategies identified
Newfoundland and Labrador - *Department of Health and Community Services*	• Not applicable<br>○ No involvement in service delivery	• Not applicable<br>○ No involvement in service delivery	• Not applicable<br>○ No involvement in service delivery	• Not applicable<br>○ No involvement in service delivery	• Not applicable<br>○ No involvement in service delivery
Newfoundland and Labrador - *Eastern Zone*	• Discussion between assessors before determining eligibility not specified	• Information shared not specified	• Regional care coordination team (P00C43)<br>• Individual physicians/nurse practitioners (P00C43)	• Regional care coordination team (P00C43)<br>• First assessor (P00C43)	• No formal process to ensure independence of assessments (P00C43)
Newfoundland and Labrador - *Western Zone*	• No (P00C44)	• No information shared (P00C44)	• Regional care coordination team (P00C44)<br>• Individual physicians/nurse practitioners (P00C44)	• Regional care coordination team (P00C44)<br>• First assessor (P00C44)	• No exchange of assessment findings (P00C44)
Newfoundland and Labrador - *Central Zone*	• Discussion between assessors before determining eligibility not specified	• Information shared not specified	• Regional care coordination team (P00B22)<br>• Individual physicians/nurse practitioners (P00B22)	• Regional care coordination team (P00B22)<br>• First assessor (P00B22)	• No formal process to ensure independence of assessments (P00B22)
Northwest Territories	• Yes (P00A2, P00A3, P00B24)	• Assessment findings (P00A2, P00A3)	• Territorial care coordination team (P00A2, P00A3, P00C35)	• Territorial care coordination team (P00A2, P00A3, P0035)	• No additional strategies identified

Jurisdiction	Do assessors discuss the case with each other before determining patient's eligibility?	What information does the two assessors share with each other before determining patients' eligibility?	Who assigns the first assessor?	Who assigns the second assessor?	What additional strategies are in place to ensure independence of assessment?
	• Simultaneous assessment is possible in some situations (P00B24)		• Individual physicians/nurse practitioners (P00A2)	• First assessor (P00C35)	
Nova Scotia - *Nova Scotia Health*	• No<br>○ Exceptional cases exist (P00A12)	• Assessment findings (in exceptional cases) (P00A12)<br>• Patients' medical chart (P00A12)<br>• Information on family dynamics (P00A12)	• Provincial care coordination team (P00A6, P00C38)	• Provincial care coordination team (P00A6, P00C38)	• No contact between assessors prior to assessment (P00A12)
Prince Edward Island - *Health PEI*	• No (P00B8, P00B17)<br>○ Simultaneous assessment is possible in some situations (e.g. patient quickly approaches end of life) (P00B17)	• Patients' medical chart (P00B8)	• Provincial care coordination team (P00B8)	• Provincial care coordination team (P00B17)	• No contact between assessors prior to assessment (P00B8)
Saskatchewan - *Saskatchewan Health Authority*	• No (P00A1, P00C1)	• No information shared (P00A1, P00C1)	• Provincial care coordination team (P00A1, P00C1)	• Provincial care coordination team (P00A1, P00C1)	• No contact between assessors prior to assessment (P00A1)
Yukon - *Department of Health and Social Services*	• No (P00C7, P00C14)	• No information shared (P00C7, P00C14)	• Individual physicians/nurse practitioners (P00C14)	• First assessor (P00C14)	• No contact between assessors prior to assessment (P00C7, P00C14)
Ontario - *Ministry of Health*	• Not applicable<br>○ No involvement in service delivery	• Not applicable<br>○ No involvement in service delivery	• Not applicable<br>○ No involvement in service delivery	• Not applicable<br>○ No involvement in service delivery	• Not applicable<br>○ No involvement in service delivery
SERVICE ORGANIZATIONS/REGIONAL FACILITIES					
Ontario - *Home and Community Care Support Services, Central East*	• Discussion between assessors before determining eligibility not specified	• Information shared not specified	• Regional care coordination team (P00C2)	• Regional care coordination team (P00C2)	• No formal process to ensure independence of assessments (P00C2)
Ontario - *Home and Community Care Support Services, Waterloo Region*	• Yes<br>○ Depends on assessor's comfort level and perceived needs (P00C4)	• Assessment findings<br>○ Accessed through electronic medical records (P00C4)	• Regional care coordination team (P00C4)	• Regional care coordination team (P00C4)	• Depends on assessors (P00C4)<br>○ No contact between assessors prior to assessment (P00C4)
Ontario - *Home and Community Care Support Services, South East*	• Yes<br>○ Depends on assessor's comfort level and perceived needs (P00C20)	• Patients' medical chart (P00C20)<br>• Assessment findings (P00C20)	• Regional care coordination team (P00C20)	• Regional care coordination team (P00C20)	• No contact between assessors prior to assessment (P00C20)
Ontario - *Home and Community Care Support Services, South West*	• No (P00C23)	• No information shared (P00C23)	• Regional care coordination team (P00C23)	• Regional care coordination team (P00C23)	• No additional strategies identified
Ontario - *Champlain Regional MAiD Network, the Ottawa Hospital*	• No (P00C5)	• Patients' medical chart<br>○ Accessed through electronic medical records (P00C5)<br>• Assessment findings<br>○ Accessed through electronic medical records (P00C5)	• Regional care coordination team (P00C5)	• Regional care coordination team (P00C5)	• No contact between assessors prior to assessment (P00C5)
HEALTHCARE FACILITIES					
Ontario - *Peterborough Regional Health Centre*	• Yes (P00C3)	• No information specified (P00C3)	• Institutional care coordination team (P00C3)	• Institutional care coordination team (P00C3)	• No contact between assessors prior to assessment (P00C3)
Ontario - *Mount Sinai Healthcare Facility, Toronto*	• Yes (P00C36)	• Assessment findings (P00C36)	• Institutional care coordination team (P00C36)	• Institutional care coordination team (P00C36)	• No additional strategies identified
Ontario - *University Health Network*	• No (P00C8)	• Assessment findings<br>○ Accessed through electronic medical records (P00C8)	• Institutional care coordination team (P00C8)	• Institutional care coordination team (P00C8)	• No additional strategies identified
Ontario - *Grand River Hospital*	• No (P00C50)	• Patients' medical chart (P00C50)	• Institutional care coordination team (P00C50)	• Institutional care coordination team (P00C50)	• No contact between assessors prior to assessment (P00C50)
COMMUNITY OF PRACTICE					
Ontario - *Hamilton Family Health Team*	• Yes (P00B18)<br>○ Patient can request not to share the assessment information with other assessors (P00B18)	• Assessment findings (P00C32)	• Institutional care coordination team (P00C32)	• Institutional care coordination team (P00C32)	• No contact between assessors prior to assessment (P00B18, P00C32)
Ontario - *Niagara Community MAiD Team, St. Catherine's, Niagara*	• Yes (P00C11)	• Assessment findings (P00C11)	• Individual physicians/nurse practitioners (P00C11)	• First assessor (P00C11)	• No additional strategies identified
GEOGRAPHICAL AREAS					

Jurisdiction	Do assessors discuss the case with each other before determining patient's eligibility?	What information does the two assessors share with each other before determining patients' eligibility?	Who assigns the first assessor?	Who assigns the second assessor?	What additional strategies are in place to ensure independence of assessment?
Ontario - *Oakville & Mississauga area*	• No (P00C18)<br>○ Exceptional cases exist (P00C18)	• Information shared not specified (P00C18)	• Provincial care coordination team (P00C18)<br>• Regional care coordination team (P00C18)<br>• Institutional care coordination team (P00C18)<br>• Individual physicians/nurse practitioner (P00C18)	• First assessor (P00C18)	• Assessment must be conducted at different times (not together) (P00C18)
Ontario - *Oakville, Mississauga & Burlington area*	• Yes (P00C21)	• Assessment findings (P00C21)	• Provincial care coordination team (P00C21)<br>• Regional care coordination team (P00C21)<br>• Individual physicians/nurse practitioners (P00C21)	• First assessor (P00C21)	• No formal process to ensure independence of assessments (P00C21)
Ontario - *Renfrew County*	• Yes (P00C22)	• Patients' medical chart (P00C22)	• Provincial care coordination team (P00C22)<br>• Regional care coordination team (P00C22)<br>○ Champlain MAiD network (P00C22)<br>• Individual physicians/nurse practitioners (P00C22)	• Provincial care coordination team (P00C22)<br>• First assessor (P00C22)	• No additional strategies identified
Ontario - *Greater Toronto Area*	• Yes<br>○ Depends on assessor's comfort level and perceived needs (P00C24)	• Assessment findings<br>○ Depends on assessor's comfort level and perceived needs (P00C24)	• Provincial care coordination team (P00C24)<br>• Individual physicians/nurse practitioners (P00C24)	• Provincial care coordination team (P00C24)<br>• First assessor (P00C24)	• No contact between assessors prior to assessment (P00C24)<br>○ Depends on assessors (P00C24)
Ontario - *Waterloo area*	• Yes<br>○ Only when there is disagreement about patient's foreseeability of death (P00C25)	• Patients' medical chart (P00C25)	• Provincial care coordination team (P00C25)<br>• Regional care coordination team (P00C25)<br>• Individual physicians/nurse practitioners (P00C25)	• First assessor (P00C25)	• No formal process to ensure independence of assessments (P00C25)
Ontario - *Noelville Sudbury, Elliot Lake, Sturgeon Falls area*	• Yes<br>○ Only when there is disagreement about patient's foreseeability of death (P00C26)	• Information shared not specified (P00C26)	• Provincial care coordination team (P00C26)<br>• Individual physicians/nurse practitioners (P00C26)	• First assessor (P00C26)	• No contact between assessors prior to assessment (P00C26)
Québec - *Ministry of Health and Social Services*	• Not applicable<br>○ No involvement in service delivery	• Not applicable<br>○ No involvement in service delivery	• Not applicable<br>○ No involvement in service delivery	• Not applicable<br>○ No involvement in service delivery	• Not applicable<br>○ No involvement in service delivery
INTEGRATED HEALTH AND SOCIAL SERVICES CENTRES					
Québec – *CISS Montérégie*	• Yes (P00C27)	• Patients' medical chart (P00C27)	• Individual physicians/nurse practitioners (P00C27)	• First assessor (127) (P00C27)	• No additional strategies identified
INTEGRATED UNIVERSITY HEALTH AND SOCIAL SERVICES CENTRES					
Québec – *CIUSS Capitale-Nationale*	• Yes<br>○ Only when there is disagreement about patient's foreseeability of death (P00C37)	• Patients' medical chart (P00C37)	• Interdisciplinary Support Group (P00C37)<br>• Individual physicians/nurse practitioners (P00C37)	• First assessor (127) (P00C37)	• No additional strategies identified
Québec – *University of Montreal Hospital Center*	• Yes (P00C40)	• Patients' medical chart (P00C40)	• Institutional care coordination team (P00C40)	• Institutional care coordination team (P00C40)<br>• First assessor (127)	• No additional strategies identified
Québec – *McGill University Health Centre*	• Yes (P00C9)	• Patients' medical chart (P00C9)	• Institutional care coordination team (P00C9)	• Institutional care coordination team (P00C9)<br>• First assessor (127)	• No additional strategies identified

Table 14: Specialist assessment

Jurisdiction	How are specialists involved in assessing patient's eligibility?	Who seeks specialists for consultation?	How do assessors determine if they require specialists to inform their decision on patients' eligibility?
Alberta - *Alberta Health Services*	• Provide expertise on how conditions may cause suffering (P00B13, P00C10)<br>• Offer alternative treatment options to relieve suffering (P00B13, P00C10)<br>• Determine patient's capacity to consent (P00C39, P00C10)	• Assessors (P00B13, P00C10)<br>○ Provincial care coordination team may assist to arrange specialist consultation (P00B13, P00C10)	• Lack of relevant expertise (77)<br>• Patient's death not reasonably foreseeable (77)
British Columbia - *Ministry of Health*	• Not applicable<br>○ No involvement in service delivery	• Not applicable<br>○ No involvement in service delivery	• Not applicable<br>○ No involvement in service delivery
British Columbia - *Fraser Health*	• Provide expertise on how conditions may cause suffering (6)	• Assessors (P00C17)<br>○ Regional care coordination team may assist to arrange specialist consultation (P00A10)	• Lack of relevant expertise (6)
British Columbia - *Interior Health*	• Provide expertise on how conditions may cause suffering (P00B14)<br>• Offer alternative treatment options to relieve suffering (P00B14)	• Assessors (P00B14)<br>○ Regional care coordination team may assist to arrange specialist consultation (P00C29)	• Lack of relevant expertise (P00B14)<br>• Patient's death not reasonably foreseeable (P00B14)
British Columbia - *Island Health*	• Provide expertise on how conditions may cause suffering (P00C13)<br>• Offer alternative treatment options to relieve suffering (P00C13)<br>• Determine patient's capacity to consent (35, 36)	• Assessors (P00C13)<br>○ Regional care coordination team may assist to arrange specialist consultation (P00C13)	• Lack of relevant expertise (P00C13)<br>• Patient's death not reasonably foreseeable (P00C13)<br>• Questioning patient's capacity to consent (35, 36)
British Columbia - *Northern Health*	• Provide expertise on how conditions may cause suffering (P00A8)	• Assessors (P00A8)<br>○ Regional care coordination team may assist to arrange specialist consultation (P00A8)	• Lack of relevant expertise (P00A8)
British Columbia - *Vancouver Coastal Health*	• Provide expertise on how conditions may cause suffering (P00B15)<br>• Offer alternative treatment options to relieve suffering (P00B16)<br>• Determine patient's capacity to consent (38-40)	• Assessors (P00B16)<br>○ By calling the Rapid Access to Consultative Expertise (RACE) (P00B16)	• Lack of relevant expertise (P00B15)<br>• Patient's death not reasonably foreseeable (P00B16)<br>• Questioning patient's capacity to consent (38-40)
British Columbia - *Provincial Health Services Authority*	• Provide expertise on how conditions may cause suffering (P00A11)	• Not applicable<br>○ They are the specialists	• Not applicable<br>○ They are the specialists
Manitoba - *Shared Health*	• Provide expertise on how conditions may cause suffering (P00B19)<br>• Offer alternative treatment options to relieve suffering (P00B21, P00C34)	• Assessors (P00C33)	• Lack of relevant expertise (128, 129) (P00C33, P00C34)<br>• Patient's death not reasonably foreseeable (P00B21, P00C33, P00C34)<br>• Questioning patient's capacity to consent (128) (P00B19, P00C33)
New Brunswick - *Horizon Health Network*	• Provide expertise on how conditions may cause suffering (P00C15)<br>• Offer alternative treatment options to relieve suffering (P00C15)	• Assessors (P00C15)	• Lack of relevant expertise (113) (P00C15)<br>• Patient's death not reasonably foreseeable (P00C15)<br>• Questioning patient's capacity to consent (113)
New Brunswick - *Vitalité Health Network*	• Provide expertise on how condition may cause suffering (P00C48) (130)	• Assessors (P00C48) (130)	• Lack of relevant expertise (P00C48) (130)
Newfoundland and Labrador - *Department of Health and Community Services*	• Not applicable<br>○ No involvement in service delivery	• Not applicable<br>○ No involvement in service delivery	• Not applicable<br>○ No involvement in service delivery
Newfoundland and Labrador - *Eastern Zone*	• Provide expertise on how conditions may cause suffering (P00C43)<br>• Offer alternative treatment options to relieve suffering (P00C43)	• Assessors (P00C43)<br>○ Regional care coordination team may assist to arrange specialist consultation (P00C43)	• Patient's death not reasonably foreseeable (P00C43)
Newfoundland and Labrador - *Western Zone*	• Provide expertise on how conditions may cause suffering (P00C44)<br>• Offer alternative treatment options to relieve suffering(P00C44)	• Assessors (P00C44)	• Patient's death not reasonably foreseeable (P00C44)
Newfoundland and Labrador - *Central Zone*	• Provide expertise on how conditions may cause suffering (P00B22)<br>• Offer alternative treatment options to relieve suffering (P00B22)	• Assessors (P00B22)	• Patient's death not reasonably foreseeable (P00B22)
Northwest Territories	• Provide expertise on how conditions may cause suffering (11) (P00A3)<br>• Offer alternative treatment options to relieve suffering (P00A3)	• Assessors (P00A3)	• Lack of relevant expertise (11) (P00A3)<br>• Patient's death not reasonably foreseeable (P00A3)<br>• Questioning patient's capacity to consent (11)

Jurisdiction	How are specialists involved in assessing patient's eligibility?	Who seeks specialists for consultation?	How do assessors determine if they require specialists to inform their decision on patients' eligibility?
Nova Scotia - *Nova Scotia Health*	• Provide expertise on how conditions may cause suffering (13, 14, 50) (P00A6)<br>• Determine patient's capacity to consent (P00A12)	• Assessors (P00A12)<br>○ Provincial care coordination team may assist to arrange specialist consultation (P00A12)	• Lack of relevant expertise (13, 14, 50) (P00A6)<br>• Patient's death not reasonably foreseeable (P00A12)<br>• Disagreement on patients' eligibility (13, 14, 50) (P00A6)
Prince Edward Island - Health PEI	• Offer alternative treatment options to relieve suffering (P00B8)<br>• Determine patient's capacity to consent (22) (P00B8)	• Assessors (P00B8)	• Based on patient's diagnosis, prognosis, treatment options (22) (P00B8)<br>• Questioning patient's capacity to consent (22) (P00B8)
Saskatchewan - *Saskatchewan Health Authority*	• Provide expertise on how conditions may cause suffering (108, 126)<br>• Determine patient's capacity to consent (108, 126)	• Assessors (P00A1)<br>○ Provincial care coordination team may assist to arrange specialist consultation (P00A1)	• Lack of relevant expertise (108, 126)<br>• Patient's death not reasonably foreseeable (108, 126)<br>• Questioning patient's capacity to consent (108, 126)
Yukon - *Department of Health and Social Services*	• Provide expertise on how conditions may cause suffering (90, 109) (P00C7)<br>• Offer alternative treatment options to relieve suffering (P00C7)	• Assessors (P00C7)	• Lack of relevant expertise (90, 109) (P00C7)<br>• Patient's death not reasonably foreseeable (P00C7)
Ontario - *Ministry of Health*	• Not applicable<br>○ No involvement in service delivery	• Not applicable<br>○ No involvement in service delivery	• Not applicable<br>○ No involvement in service delivery
SERVICE ORGANIZATIONS/REGIONAL FACILITIES			
Ontario - *Home and Community Care Support Services, Central East*	• Confirm diagnosis and prognosis (P00C2)<br>• Provide expertise on how conditions may cause suffering (P00C2)<br>○ Assess suicidality (psychiatric assessment) if required (P00C2)	• Assessors (P00C2)	• Patient's death not reasonably foreseeable (P00C2)<br>• Lack of relevant expertise (P00C2)<br>• Questioning patient's capacity to consent (P00C2)
Ontario - *Home and Community Care Support Services, Waterloo Region*	• Provide expertise on how conditions may cause suffering (P00C4)	• Assessors (P00C4)	• Lack of relevant expertise (P00C4)
Ontario - *Home and Community Care Support Services, South East*	• Provide expertise on how conditions may cause suffering (P00C20)<br>• Offer alternative treatment options to relieve suffering (P00C20)	• Assessors (P00C20)	• Lack of relevant expertise (P00C20)<br>• Patient's death not reasonably foreseeable (P00C20)
Ontario - *Home and Community Care Support Services, South West*	• No experience with specialist consultation	• No experience with specialist consultation	• No experience with specialist consultation
Ontario - *Champlain Regional MAiD Network, the Ottawa Hospital*	• Provide expertise on how conditions may cause suffering (P00C5)	• Assessors (P00C5)	• Lack of relevant expertise (P00C5)
HEALTHCARE FACILITIES			
Ontario - *Peterborough Regional Health Centre*	• Provide expertise on how conditions may cause suffering (P00C3)	• Assessors (P00C3)<br>○ Institutional care coordination team may assist to arrange specialist consultation (P00C3)	• Lack of relevant expertise (P00C3)<br>• Patient's death not reasonably foreseeable (P00C3)
Ontario - *Mount Sinai Healthcare Facility, Toronto*	• Provide expertise on how conditions may cause suffering (P00C36)<br>• Determine patient's capacity to consent (P00C36)	• Assessors (P00C36)	• Lack of relevant expertise (P00C36)<br>• Patient's death not reasonably foreseeable (P00C36)
Ontario - *University Health Network*	• Provide expertise on how conditions may cause suffering (P00C8)	• Assessors (P00C8)<br>○ Institutional care coordination team may assist to arrange specialist consultation (P00C8)	• Lack of relevant expertise (P00C8)<br>• Patient's death not reasonably foreseeable (P00C8)
Ontario - *Grand River Hospital*	• Provide expertise on how conditions may cause suffering (P00C50)	• Assessors (P00C50)<br>○ Institutional care coordination team may assist to arrange specialist consultation (P00C50)	• Based on patient's diagnosis, prognosis, treatment options (P00C50)
COMMUNITY OF PRACTICE			
Ontario - *Hamilton Family Health Team*	• Offer alternative treatment options to relieve suffering (P00B18, P00C32)	• Assessors (P00B18, P00C32)<br>○ Institutional care coordination team may assist to arrange specialist consultation (P00B18, P00C32)	• Lack of relevant expertise (P00C18, P00C32)<br>• Patient's death not reasonably foreseeable (P00C18, P00C32)
Ontario - *Niagara Community MAiD Team, St. Catherine's, Niagara*	• Offer alternative treatment options to relieve suffering (P00C11)	• Assessors (P00C11)	• Lack of relevant expertise (P00C11)
GEOGRAPHICAL AREAS			
Ontario - *Oakville & Mississauga area*	• Provide expertise on how conditions may cause suffering (P00C18)	• Assessors (P00C18)	• Lack of relevant expertise (P00C18)
Ontario - *Oakville, Mississauga, & Burlington area*	• Provide expertise on how conditions may cause suffering (P00C21)<br>• Offer alternative treatment options to relieve suffering (P00C21)	• Assessors (P00C21)	• Lack of relevant expertise (P00C21)<br>• Patient's death not reasonably foreseeable (P00C21)

Jurisdiction	How are specialists involved in assessing patient's eligibility?	Who seeks specialists for consultation?	How do assessors determine if they require specialists to inform their decision on patients' eligibility?
Ontario - *Renfrew County*	• Provide expertise on how conditions may cause suffering (P00C22)<br>• Offer alternative treatment options to relieve suffering (P00C22)	• Assessors (P00C22)	• Lack of relevant expertise (P00C22)<br>• Patient's death not reasonably foreseeable (P00C22)
Ontario - *Greater Toronto Area*	• Provide expertise on how conditions may cause suffering (P00C24)<br>• Offer alternative treatment options to relieve suffering (P00C24)<br>• Determine patient's capacity to consent (P00C24)	• Assessors (P00C24)	• Lack of relevant expertise (P00C24)<br>• Questioning patient's capacity to consent (P00C24)
Ontario - *Waterloo area*	• Offer alternative treatment options to relieve suffering (P00C25)	• Assessors (P00C25)	• Patient's death not reasonably foreseeable (P00C25)
Ontario -*Noelville Sudbury, Elliot Lake, Sturgeon Falls area*	• Offer alternative treatment options to relieve suffering (P00C26)	• Assessors (P00C26)	• Patient's death not reasonably foreseeable (P00C26)
Québec - *Ministry of Health and Social Services*	• Not applicable<br>○ No involvement in service delivery	• Not applicable<br>○ No involvement in service delivery	• Not applicable<br>○ No involvement in service delivery
INTEGRATED HEALTH AND SOCIAL SERVICES CENTRES			
Québec – *CISS Montérégie*	• No experience with specialist consultation	• No experience with specialist consultation	• No experience with specialist consultation
INTEGRATED UNIVERSITY HEALTH AND SOCIAL SERVICES CENTRES			
Québec – *CIUSS Capitale-Nationale*	• Provide expertise on how conditions may cause suffering (110) (P00C37)	• Assessors (110) (P00C37)	• Lack of relevant expertise (110) (P00C37)
Québec – *University of Montreal Hospital Center*	• Provide expertise on how conditions may cause suffering (P00C40)	• Assessors (P00C40)	• Lack of relevant expertise (P00C40)<br>• Disagreement on patients' eligibility (P00C40)
Québec – *McGill University Health Centre*	• Provide expertise on how conditions may cause suffering (P00C9)	• Assessors (P00C9)	• Disagreement on patients' eligibility (P00C9)

Table 15: Provision process

					Medication protocol								
	**Family dynamics**		**MAiD consent process**		**Intravenous**		**Oral**			**Pharmacy involved**		**Post-provision arrangements**	
**Jurisdiction**	**What are the strategies and guidelines MAiD team/assessors and providers utilize to address family dynamics**	**What supports are provided to patients who are alone?**	**Can final consent be waived for foreseeable natural death?**	**How are final consents provided?**	**What are the protocols for clinician-administered medication?**	**Which professionals are present for clinician administration?**	**What are the protocols for self-administrated medication?**	**What are the processes in place if a self-administrated medication fails?**	**Which professionals are present for self-administration?**	**Which pharmacies provide MAiD kit?**	**Does the pharmacy dispense a backup kit?**	**How is the death certificate completed?**	**Who is responsible for coordinating funeral arrangements?**
Alberta - *Alberta Health Services*	Strategies<br>• MAiD team<br>○ Coordinators<br>▪ Facilitate access to support services (P00B9, P00B13)<br>▪ Encourage patients to have conversations with families (P00A5, P00B13)	• MAiD team (P00B9)<br>○ Coordinators (nurse navigators)<br>▪ Identify existing supports available for patients (P00B9)<br>• Community/ hospital social workers (P00B9)<br>○ Provide support services to patients (P00B9)	• Yes (77, 131)<br>• If patient meets the following conditions before losing capacity to consent<br>○ Patient meets all requirements and safeguards for MAiD (77, 131)<br>○ Patient enters into written agreement with physicians/nurse practitioners to receive MAiD on or before a specified day (77, 131)<br>○ Patient is informed of risk of losing capacity to consent to receive MAiD prior to specified date (77, 131)	• Written (77)<br>• Alternatives to written consent:<br>○ Marking (77)	• Provincial standardized medication protocol<br>○ Lorazepam (1- 4mg)<br>○ Midazolam (2.5-10mg)<br>○ Lidocaine (40mg of 1% or 2%)<br>▪ or Magnesium sulfate (1000mg)<br>○ Propofol (1000 mg)<br>▪ or Phenobarbital (3000mg)<br>○ Rocuronium (200mg)<br>▪ or Cistracurium (30mg) (P00C39)<br>• Optional opioids for premedication include:<br>○ Fentanyl (25-500 mcg) (P00C39)	• Physicians/nurse practitioners (5)<br>○ Must stay until death is confirmed (P00C39)<br>• Registered nurse (P00A5)	• Provincial standardized medication protocol<br>○ Metoclopramide (20mg)<br>○ Ondansetron (8mg)<br>○ Lorazepam (0.25-0.5mg)<br>○ Coma-inducing compound<br>▪ Secobarbital sodium (15g) (P00C39)	• Advanced consent obtained (P00A5, P00B9, P00C12, P00B13)<br>○ Intravenous administration occurs after an agreed period of time (P00B9)<br>○ IV cannula placed prior to self-administration (P00B13)	• Physicians/nurse practitioners<br>○ Must stay until death is confirmed (P00C39)	• Hospital pharmacy (P00B9, P00B13)<br>• Community pharmacy (P00B9, P00B13)	• Yes (P00A5)<br>○ Unused medication is returned to pharmacy (P00A5)	• Responsible professional<br>○ Chief Medical Examiner (P00A5)<br>• Cause of death<br>○ Administration of drugs due to underlying illness, disease or disability (132)<br>• Manner of death<br>○ Unclassified (132)	• Patient's family (P00A5)<br>• Physicians/nurse practitioners (P00A5)<br>• MAiD team<br>○ Coordinators (Nurse navigators) (P00A5, P00C39)<br>• Community/hospital social workers (P00B9)<br>• Office of the Public Guardian (P00B9)
British Columbia - *Ministry of Health*	• Not applicable<br>○ Varies across health authorities (P00A4)	• Not applicable<br>○ Varies across health authorities (P00A4)	• Yes (122, 123, 133)<br>• If patient meets the following conditions before losing capacity to consent<br>○ Patient meets all requirements and safeguards for MAiD (134, 135)<br>○ Patient enters into written agreement with physicians/nurse practitioners to receive MAiD on or before a specified day (134, 135)	• Not applicable	• Provincial standardized medication protocol<br>○ Lidocaine (1% - 2%)<br>○ Bupivacaine (1 - 5mg/mL)<br>○ Midazolam (2.5-10mg)<br>○ Phenobarbital sodium (120mg/mL)<br>○ Propofol (10mg/mL)<br>○ Rocuronium bromide (10mg/mL)<br>○ Sodium chloride (0.9%) (136)	• Physicians/nurse practitioners (27)<br>○ Must stay until death is confirmed (123, 137)	• Provincial standardized medication protocol<br>○ Haloperiodol injection (5 mg/mL)<br>○ Lorazepam (0.5mg SL)<br>○ Metoclopramide (10mg)<br>○ Ondansetron (8mg)<br>○ Phenobarbital + chloral hydrate + morphine sulphate oral suspension	• Advanced Consent for Failed Self-Administration (122)<br>○ Written agreement between patient and provider<br>○ If patient loses capacity to consent, provider can administer intravenously after an agreed upon amount of time (122)	• Physicians/nurse practitioners (122)<br>○ Must stay until death is confirmed (123, 137)<br>• Registered nurse (123, 137)<br>• Pharmacists (123, 137)	• Hospital Pharmacy (P00A4)<br>• Community pharmacy (P00A4)	• Yes (P00A4)<br>○ Provincial requirement	• Responsible professional<br>○ Physicians/nurse practitioners (122, 138)<br>• Cause of death<br>○ MAiD due to underlying illness, diseases or disability (122)<br>• Manner of death<br>○ Natural (122)	• Not applicable

					Medication protocol								
	Family dynamics		MAiD consent process		Intravenous		Oral			Pharmacy involved		Post-provision arrangements	
**Jurisdiction**	**What are the strategies and guidelines MAiD team/assessors and providers utilize to address family dynamics**	**What supports are provided to patients who are alone?**	**Can final consent be waived for foreseeable natural death?**	**How are final consents provided?**	**What are the protocols for clinician-administered medication?**	**Which professionals are present for clinician administration?**	**What are the protocols for self-administrated medication?**	**What are the processes in place if a self-administrated medication fails?**	**Which professionals are present for self-administration?**	**Which pharmacies provide MAiD kit?**	**Does the pharmacy dispense a backup kit?**	**How is the death certificate completed?**	**Who is responsible for coordinating funeral arrangements?**
			○ Patient is informed of risk of losing capacity to consent to receive MAiD prior to specified date (122)<br>○ Waiver cannot be created on same day as provision (134, 135)<br>○ If an alternative prescriber becomes responsible for provision, another waiver must be signed (134, 135)<br>• MAiD is provided if patient<br>○ Becomes incapable of consenting (134, 135)<br>○ Shows no indication of declining MAiD (134, 135)				○ Secobarbital compound suspension (136)						
British Columbia - *Fraser Health*	Strategies<br>• MAiD team<br>○ Encourage patients to have conversations with families (P00A10)<br>Guidelines<br>• Practice guideline for family-related matters (P00A10)	• MAiD team<br>○ Coordinator (social worker)<br>▪ Identifies existing supports available for patients (P00A10)<br>• Community social workers<br>○ Provide support services to patients (P00A10)	• Yes (6, 28, 106)<br>○ Same with Ministry of Health	• Written (135)<br>• Alternatives to written consent:<br>○ Verbal (135)<br>○ Marking (139)	• Provincial standardized medication protocol (136)	• Physicians/nurse practitioners (28)<br>○ Must stay until death is confirmed (123, 137)	• Provincial standardized medication protocol (136)	• Same as provincial regulations (19) (P00A10)<br>• Provider monitors process (P00A10)<br>• Provider can administer intravenously after an agreed upon amount of time (P00A10)	• Physicians/nurse practitioners<br>○ Must stay until death is confirmed (28) (P00A10)<br>• MAiD support nurse (P00A10)	• Hospital pharmacy (P00A10)<br>• Community pharmacy (P00A10)	• Yes (P00A4)<br>○ Provincial requirement	• Responsible professional<br>○ Physicians/nurse practitioners (P00A10)<br>• Cause of death<br>○ MAiD due to underlying illness, diseases or disability (122)<br>• Manner of death<br>○ Natural (122)	• Patient's family (P00A10)<br>• Physicians (P00C17)<br>• Executor of will (P00A10)

Jurisdiction	Family dynamics		MAiD consent process		Medication protocol					Pharmacy involved		Post-provision arrangements	
					Intravenous		Oral						
	What are the strategies and guidelines MAiD team/assessors and providers utilize to address family dynamics	What supports are provided to patients who are alone?	Can final consent be waived for foreseeable natural death?	How are final consents provided?	What are the protocols for clinician-administered medication?	Which professionals are present for clinician administration?	What are the protocols for self-administrated medication?	What are the processes in place if a self-administrated medication fails?	Which professionals are present for self-administration?	Which pharmacies provide MAiD kit?	Does the pharmacy dispense a backup kit?	How is the death certificate completed?	Who is responsible for coordinating funeral arrangements?
British Columbia - *Interior Health*	Strategies<br>• MAiD team<br>○ Coordinators<br>▪ Encourage patients to have conversations with families (P00B14)<br>▪ Provide information on available resources to patients and families (P00B14)<br>• Providers<br>○ Provide support to patients and families (P00C29)	• MAiD team (P00B14)<br>○ Coordinator (social worker) (P00B14)<br>▪ Identifies existing supports available for patients (P00B14)<br>• Community/ hospital social worker<br>○ Provides support services to patients (P00B14)	• Yes (33)<br>○ Same with Ministry of Health	• Written (135)<br>• Alternatives to written consent:<br>○ Verbal (135)<br>○ Marking (139)	• Provincial standardized medication protocol (136)	• Physicians/nurse practitioners (33)<br>○ Must stay until death is confirmed (123, 137)	• Provincial standardized medication protocol (136)	• Same as provincial regulations<br>○ Written agreement between patient and provider is signed<br>○ If patient loses capacity to consent, provider can administer intravenously (P00C29)	• Physicians/nurse practitioner (33)<br>○ Must stay until death is confirmed (123, 137)<br>• Support nurse (P00B14)	• Hospital pharmacy (P00C29)<br>• Community pharmacy (P00C29)	• Yes (P00A4)<br>○ Provincial requirement	• Professional<br>○ Physicians/nurse practitioners (P00C29, P00C41)<br>• Cause of death<br>○ MAiD due to underlying illness, diseases or disability (122)<br>• Manner of death<br>○ Natural (122)	• Patient's family (P00A5)<br>• Patient (P00A5)<br>• MAiD team (P00A5)<br>○ Coordinator (social worker) (P00A5)
British Columbia - *Island Health*	Strategies<br>• MAiD team<br>○ Coordinators<br>▪ Facilitate access to support services (P00C13)<br>• Providers<br>○ Provide support to patients and families (P00C13)<br>○ Facilitate access to support services (e.g., social worker, ethics) (P00C13)	• MAiD team (P00A9)<br>○ Coordinators<br>▪ Identify existing supports available for patients (P00A9)<br>• Community/hospital social workers<br>○ Provide support services to patients (P00A9)<br>• Spiritual health provider (P00A9)<br>○ Provides support services to patients (P00A9)<br>• Home care nurse (P00A9)	• Yes<br>○ Same with Ministry of Health	• Written (135)<br>• Alternatives to written consent:<br>○ Verbal (135)<br>○ Marking (139)	• Provincial standardized medication protocol (136)	• Physicians/nurse practitioners (107)<br>○ Must stay until death is confirmed (123, 137)<br>• Registered nurse<br>○ Present to establish intravenous route (P00C13)	• Provincial standardized medication protocol (136)	• Same as provincial regulation<br>○ Written agreement between patient and provider<br>○ If patient loses capacity to consent, provider can administer intravenously after an agreed upon amount of time (122)	• Physicians/nurse practitioners<br>○ Must be within immediate vicinity when medication is administered (P00C13)	• Hospital pharmacy (P00A9, P00A13, P00C13)<br>• Community pharmacy (P00A9, P00A13, P00C13)	• Yes (P00A4)<br>○ Provincial requirement	• Responsible professional<br>○ Physicians/nurse practitioners (P00C13)<br>• Cause of death<br>○ MAiD due to underlying illness, diseases or disability (122)<br>• Manner of death<br>○ Natural (122)	• Patient's family (P00A9)<br>• Executor after death (P00A9)<br>• Office of the Public Trustee (P00A13)

Jurisdiction	Family dynamics: What are the strategies and guidelines MAiD team/assessors and providers utilize to address family dynamics	Family dynamics: What supports are provided to patients who are alone?	MAiD consent process: Can final consent be waived for foreseeable natural death?	MAiD consent process: How are final consents provided?	Medication protocol – Intravenous: What are the protocols for clinician-administered medication?	Medication protocol – Intravenous: Which professionals are present for clinician administration?	Medication protocol – Oral: What are the protocols for self-administered medication?	Medication protocol – Oral: What are the processes in place if a self-administrated medication fails?	Medication protocol – Oral: Which professionals are present for self-administration?	Pharmacy involved: Which pharmacies provide MAiD kit?	Pharmacy involved: Does the pharmacy dispense a backup kit?	Post-provision arrangements: How is the death certificate completed?	Post-provision arrangements: Who is responsible for coordinating funeral arrangements?
		○ Provides support services to patients (P00A9)											
British Columbia - *Northern Health*	Strategies<br>• Providers<br>○ Encourage patients to have conversations with families (P00A8)	• Community/hospital social workers<br>○ Provide support services to patients (P00A8)	• Yes (P00A8)<br>○ Same with Ministry of Health	• Written (135)<br>• Alternatives to written consent:<br>○ Verbal (135)<br>○ Marking (139)	• Provincial standardized medication protocol (136)	• Physicians/nurse practitioners (P00A8)<br>○ Must stay until death is confirmed (123, 137, 140)	• Provincial standardized medication protocol (136)	• Same as provincial regulation<br>○ Written agreement between patient and provider<br>○ If patient loses capacity to consent, provider can administer intravenously after an agreed upon amount of time (115)	• Physicians/nurse practitioners<br>○ Must stay until death is confirmed (140)	• Hospital pharmacy (P00A8)<br>• Community pharmacy (P00A8)	• Yes (P00A4)<br>○ Provincial requirement	• Responsible professional<br>○ Physicians/nurse practitioners (P00A8)<br>• Cause of death<br>○ MAiD due to underlying illness, diseases or disability (122)<br>• Manner of death<br>○ Natural (122)	• Responsibility for funeral arrangements not specified

	Family dynamics		MAiD consent process		Medication protocol					Pharmacy involved		Post-provision arrangements	
					Intravenous		Oral						
**Jurisdiction**	**What are the strategies and guidelines MAiD team/assessors and providers utilize to address family dynamics**	**What supports are provided to patients who are alone?**	**Can final consent be waived for foreseeable natural death?**	**How are final consents provided?**	**What are the protocols for clinician-administered medication?**	**Which professionals are present for clinician administration?**	**What are the protocols for self-administrated medication?**	**What are the processes in place if a self-administrated medication fails?**	**Which professionals are present for self-administration?**	**Which pharmacies provide MAiD kit?**	**Does the pharmacy dispense a backup kit?**	**How is the death certificate completed?**	**Who is responsible for coordinating funeral arrangements?**
British Columbia - *Vancouver Coastal Health*	Strategies<br>• MAiD team<br>○ Coordinators (social workers)<br>▪ Provide informal support services to patients and families (P00A7, P00B10, P00B15, P00B16)<br>▪ Facilitate access to support services (P00B15)<br>• Providers<br>▪ Facilitate access to support services (P00A7, P00B10, P00B12)	• MAiD team (P00B12)<br>○ Coordinators (social workers)<br>▪ Provide support services to patients (P00B12)<br>• Community/hospital social workers (P00B10)<br>○ Provide support services to patients (P00B10)	• Yes<br>○ Same with Ministry of Health	• Written (135)<br>• Alternatives to written consent:<br>○ Verbal (135)<br>○ Marking (139)	• Provincial standardized medication protocol (136)<br>• If IV insertion is difficult, a Peripherally Inserted Central Catheter line (PICC) is inserted (P00A7)	• Physicians/nurse practitioners (40)<br>○ Must stay until death is confirmed (40)<br>• Registered nurse (P00B15)	• Provincial standardized medication protocol (136)<br>• Support nurse must establish intravenous routes before provision (P00B10)<br>• Patient consume oral medication within 4 minutes (40)	• Same as provincial regulation<br>○ Written agreement between patient and provider<br>○ If patient does not tolerate medication, provider administers intravenously (40)	• Physicians/nurse practitioners (40)<br>○ Must stay until death is confirmed (40)<br>• Registered nurse (40)	• Hospital pharmacy (P00A7)<br>• Community pharmacy (P00A7)	• Yes (P00A4)<br>○ Provincial requirement	• Responsible professional<br>○ Physicians/nurse practitioners (P00A7)<br>• Cause of death<br>○ MAiD due to underlying illness, diseases or disability (122)<br>• Manner of death<br>○ Natural (122)	• Patient's family (P00B15, P00B10)<br>• Patients (P00B12)<br>• MAiD team (P00B15, P00B10)<br>○ Coordinators (social workers) (P00B15, P00B10)
British Columbia - *Provincial Health Services Authority*	• Strategies and guidelines not specified	• Supports for alone patients not specified	• Yes<br>○ Same with Ministry of Health	• Written (135)<br>• Alternatives to written consent:<br>○ Verbal (135)<br>○ Marking (139)	• Provincial standardized medication protocol (136)	• Physicians/nurse practitioners (27)<br>○ Must stay until death is confirmed (123, 137)	• Provincial standardized medication protocol (136)	• Same as provincial regulation<br>○ Written agreement between patient and provider<br>○ If patient loses capacity to consent, provider can administer intravenously after an agreed upon amount of time (122)	• Physicians/nurse practitioners (122)<br>○ Must stay until death is confirmed (123, 137)	• Hospital Pharmacy (P00A4)<br>• Community pharmacy (P00A4)	• Yes (P00A4)<br>○ Provincial requirement	• Responsible professional<br>○ Physicians/nurse practitioners (122, 138)<br>• Cause of death<br>○ MAiD due to underlying illness, diseases or disability (122)<br>• Manner of death<br>○ Natural (122)	• Responsibility for funeral arrangements not specified

					Medication protocol								
	Family dynamics		MAiD consent process		Intravenous		Oral			Pharmacy involved		Post-provision arrangements	
Jurisdiction	What are the strategies and guidelines MAiD team/assessors and providers utilize to address family dynamics	What supports are provided to patients who are alone?	Can final consent be waived for foreseeable natural death?	How are final consents provided?	What are the protocols for clinician-administered medication?	Which professionals are present for clinician administration?	What are the protocols for self-administered medication?	What are the processes in place if a self-administered medication fails?	Which professionals are present for self-administration?	Which pharmacies provide MAiD kit?	Does the pharmacy dispense a backup kit?	How is the death certificate completed?	Who is responsible for coordinating funeral arrangements?
Manitoba - *Shared Health*	Strategies<br>• MAiD team<br>○ Coordinators<br>▪ Facilitates access to support services (P00C34)<br>▪ Notifies families after patients' MAiD provision (P00C34)<br>○ Social workers<br>▪ Provide support and counselling services (P00B19, P00C33)	• MAiD team<br>○ Coordinators<br>▪ Mandate patients to notify any individual of their MAiD provision (P00C34)<br>○ Social workers (P00B19)<br>▪ Offer emotional support and practical assistance (P00B19)<br>• Hospital social workers (P00B19)<br>○ Provide support services to inpatients (P00B19)	• Yes (128)<br>• If patient meets the following conditions before losing capacity to consent<br>○ Patient meets all requirements and safeguards for MAiD (128)<br>○ Patient enters into written agreement with physicians/nurse practitioners to receive MAiD on or before a specified day (128)<br>○ Patient is informed of risk of losing capacity to consent to receive MAiD prior to specified date (128)<br>• MAiD is provided if patient<br>○ Becomes incapable of consenting (128)<br>○ Shows no indication of declining MAiD (128)	• Written (P00C33)<br>• Alternatives to written consent:<br>○ Verbal<br>○ Blinks<br>○ Hand squeezes (P00B19)	• Provincial standardized medication protocol (P00C33, P00C34)<br>○ Midazolam (10mg)<br>○ Lidocaine (100 mg 2%)<br>▪ Omitted if using central venous access (P00C34)<br>○ Propofol (500 mg)<br>○ Rocuronium (300 mg)<br>○ A PICC line is inserted if IV insertions are difficult (P00C33)<br>○ Existing port can be used (P00C33)	• Physicians/nurse practitioners (141)<br>• Registered nurse (141)<br>• MAID team<br>○ Social worker (141)	• MAiD medication for self-administration is unavailable (P00C34, P00C33)	• Not applicable<br>○ No self-administration protocol (P00C34, P00C33)	• Not applicable<br>○ No self-administration protocol (P00C34, P00C33)	• Hospital pharmacy (P00C33, P00B19, P00C34)<br>• Community pharmacy (P00C33, P00B19, P00C34)	• Yes (P00C33)<br>○ Must return kit if not used (P00C33)	• Responsible professional<br>○ Physicians/nurse practitioners (142)<br>• Cause of death<br>○ Underlying illness, disease or disability (P00C33)<br>• Manner of death<br>○ Natural<br>▪ Indicated as "MAiD was provided" (P00C33)	• Patient's family (P00C33)<br>• Patient (P00C33)<br>• Physicians/nurse practitioners (P00C34)<br>• MAiD team<br>○ Social workers (P00B19)
New Brunswick - *Horizon Health Network*	Strategies<br>• MAiD team<br>○ Coordinator<br>▪ Provides information on available resources to patients and families (P00B11)<br>▪ Encourages patients to	• MAiD team (P00C15)<br>○ Coordinator<br>▪ Identifies existing supports available for patients (P00C15)<br>○ Acting Medical Lead (P00C15)	• Yes (9, 113)<br>• If patient meets the following conditions before losing capacity to consent<br>○ Patient meets all requirements and safeguards for MAiD (9, 113)<br>○ Patient enters into written agreement with	• Verbal (9) (P00C15)	• Regional standardized medication protocol (P00C15)<br>○ Midazolam (20mg)<br>▪ Second dose of 20mg, if needed<br>○ Lidocaine (40 mg 2%)<br>▪ or Magnesium sulphate (1000mg)<br>○ Propofol (1000 mg)<br>▪ or Phenobarbital (1500mg)	• Physicians/nurse practitioners (P00B11)<br>• Registered Nurse<br>○ Home care nurse (P00C15)<br>○ Hospital nurse (P00C15)	• Regional standardized medication protocol<br>○ Metoclopramide (10mg) (143)<br>○ Ondansetron (8mg) (143)<br>○ Lorazepam (0.5mg) as needed (143)<br>○ Coma-inducing compound	• A written agreement occurs between patient and provider (P00C15)<br>○ Provider can administer intravenously after an agreed upon amount of time (P00C15)	• Physicians/nurse practitioners (P00C15)<br>• Registered nurse<br>○ Home care nurse (P00C15)<br>○ Hospital nurse (P00C15)	• Hospital pharmacy (P00B11, P00C15)	• No (P00C15)<br>○ Only a second dose of 2 medication is included (P00C15)	• Responsible professional<br>○ Physicians/nurse practitioners (P00C15)<br>• Cause of death<br>○ Drug toxicity due to MAiD due to underlying illness, disease, or disability (142)<br>• Manner of death	• Patient's family (P00B11)<br>• Patient (P00B11)

Jurisdiction	Family dynamics		MAiD consent process		Medication protocol: Intravenous		Medication protocol: Oral			Pharmacy involved		Post-provision arrangements	
	**What are the strategies and guidelines MAiD team/assessors and providers utilize to address family dynamics**	**What supports are provided to patients who are alone?**	**Can final consent be waived for foreseeable natural death?**	**How are final consents provided?**	**What are the protocols for clinician-administered medication?**	**Which professionals are present for clinician administration?**	**What are the protocols for self-administrated medication?**	**What are the processes in place if a self-administrated medication fails?**	**Which professionals are present for self-administration?**	**Which pharmacies provide MAiD kit?**	**Does the pharmacy dispense a backup kit?**	**How is the death certificate completed?**	**Who is responsible for coordinating funeral arrangements?**
	have conversations with families (P00B11)	▪ Identifies existing supports available for patients (P00C15)<br>• Community social workers (P00C15)<br>○ Provide support services to patients (P00C15)<br>• Home care nurse (P00C15)<br>○ Provides support services to patients (P00C15)	physicians/nurse practitioners to receive MAiD on or before a specified day (9, 113)<br>○ Patient is informed of risk of losing capacity to consent to receive MAiD prior to specified date (9, 113)<br>• MAiD is provided if patient<br>○ Becomes incapable of consenting (9, 113)<br>○ Shows no indication of declining MAiD (9, 113)		○ NaCl (0.9%)<br>○ Rocuronium (200mg)<br>▪ Or Cistracurium (30mg)<br>○ Bupivacaine (optional) (400mg) (P00C15)		▪ Secobarbital powder (15g)<br>▪ Sterile water (15mL)<br>▪ Ethyl alcohol (20mL)<br>▪ Propylene glycol (10mL)<br>▪ Saccharin (240mg)<br>▪ Anise oil (1 drop)<br>▪ Simple syrup (add to make total volume of 100mL) (143)<br>• Self-administration is not recommended (P00B11, P00C15)<br>• IV cannula placed prior to oral administration (P00B11)					○ Natural (142)	
New Brunswick - *Vitalité Health Network*	• Strategies and guidelines not specified	• Home care nurse (P00C48)<br>○ Provides support services to patients (P00C48)<br>• Community social workers (P00C48)<br>○ Provide support services to patients (P00C48)	• Yes (144)<br>○ If the patient meets the following conditions<br>▪ Meets all requirement and safeguards of MAiD (144)<br>▪ Enters into written agreement with physicians/nurse practitioners to provide MAiD on or before a specified date (144)	• Verbal (P00C48)	• Regional standardized medication protocol (P00C48) (145)<br>○ Lorazepam (2mg)<br>▪ or Midazolam (5mg) (145)<br>○ Lidocaine<br>▪ or Magnesium sulphate (145)<br>○ Propofol (10mg)<br>▪ or Phenobarbital (145)<br>○ Rocuronium (200mg)<br>▪ or Cisatracurium (30mg) (145)<br>○ Bupivacaine (400mg) (optional) (145)	• Physicians/nurse practitioner (P00C48)<br>• Home care nurse (P00C48)	• Regional standardized medication protocol<br>○ Metoclopramide (20mg)<br>▪ or Ondansetron (8mg) (145)<br>○ Lorazepam (0.5 – 1mg)<br>○ Coma-inducing compound<br>▪ Phenobarbital powder (20g)<br>▪ Chloral hydrate powder (20g)<br>▪ Morphine sulphate powder (3g)<br>▪ Acesulfame potassium (365mg)	• A written agreement occurs between patient and provider (P00C48)<br>○ Provider can administer intravenously after an hour (145) (P00C48)	• Physicians/nurse practitioners (145)	• Hospital pharmacy (P00C48)	• Availability of backup kit not specified	• Responsible professional<br>○ Physicians/nurse practitioners (P00C48)<br>• Cause of death<br>○ Drug toxicity due to MAiD due to underlying illness, disease, or disability (142)<br>• Manner of death<br>○ Natural (142)	• Hospital staff (P00C48)<br>• Home care nurse (P00C48)

Jurisdiction	Family dynamics: What are the strategies and guidelines MAiD team/assessors and providers utilize to address family dynamics	Family dynamics: What supports are provided to patients who are alone?	MAiD consent process: Can final consent be waived for foreseeable natural death?	MAiD consent process: How are final consents provided?	Medication protocol – Intravenous: What are the protocols for clinician-administered medication?	Medication protocol – Intravenous: Which professionals are present for clinician administration?	Medication protocol – Oral: What are the protocols for self-administrated medication?	Medication protocol – Oral: What are the processes in place if a self-administrated medication fails?	Medication protocol – Oral: Which professionals are present for self-administration?	Pharmacy involved: Which pharmacies provide MAiD kit?	Pharmacy involved: Does the pharmacy dispense a backup kit?	Post-provision arrangements: How is the death certificate completed?	Post-provision arrangements: Who is responsible for coordinating funeral arrangements?
							▪ Steviol Glycosides<br>▪ Magnasweet (360mg)<br>▪ Suspending liquid component (145)						
Newfoundland and Labrador - *Department of Health and Community Services*	• Not applicable<br>○ Varies across zones	• Not applicable<br>○ Varies across zones	• Yes (146, 147)<br>• If patient meets the following conditions before losing capacity to consent<br>○ Patient meets all requirements and safeguards for MAiD (146, 147)<br>○ Patient enters into written agreement with Physicians/nurse practitioners to receive MAiD on or before a specified day (146, 147)<br>○ Patient is informed of risk of losing capacity to consent to receive MAiD prior to specified date (146, 147)<br>• MAiD is provided if patient<br>○ Becomes incapable of consenting (146, 147)<br>○ Shows no indication of declining MAiD (146, 147)	• Patient must explicitly express their final consent to proceed (146, 147)	• No provincial standardized medication protocol (148)<br>• Regional protocols show slight variation (148)	• Physicians/nurse practitioners (146, 147)	• No provincially standardized protocol (148)	• Advanced Consent for Failed Self-Administration is obtained<br>○ Written agreement between patient and provider<br>○ If patient loses capacity to consent, provider can administer intravenously after an agreed upon amount of time (146)	• MAiD medication for self-administration is unavailable (P00C44)	• Hospital pharmacy (148)<br>• Community pharmacy (148)	• Availability of backup kit not specified	• Responsible professional<br>○ Physicians/nurse practitioners (148)<br>• Cause of death<br>○ Medication administered due to medical conditions for requesting MAiD (142)<br>• Manner of death<br>○ Natural (142)	• Not applicable<br>○ Varies across zones
Newfoundland and Labrador - *Eastern Zone*	Strategies<br>• MAiD team<br>○ Coordinators	• MAiD team<br>○ Coordinators (navigators)	• Yes (149)<br>• If patient meets the following conditions	• Patient must explicitly express their	• Regional standardized medication protocol (150)	• Physicians/nurse practitioners (48)	• MAiD medication protocol for self-administration is	• MAiD medication protocol for self-administration is	• MAiD medication protocol for self-administration is	• Hospital pharmacy (148)	• Availability of backup kit not specified	• Responsible professional	• Responsibility for funeral arrangements not specified

Jurisdiction	Family dynamics		MAiD consent process		Medication protocol					Pharmacy involved		Post-provision arrangements	
					Intravenous		Oral						
	What are the strategies and guidelines MAiD team/assessors and providers utilize to address family dynamics	What supports are provided to patients who are alone?	Can final consent be waived for foreseeable natural death?	How are final consents provided?	What are the protocols for clinician-administered medication?	Which professionals are present for clinician administration?	What are the protocols for self-administrated medication?	What are the processes in place if a self-administrated medication fails?	Which professionals are present for self-administration?	Which pharmacies provide MAiD kit?	Does the pharmacy dispense a backup kit?	How is the death certificate completed?	Who is responsible for coordinating funeral arrangements?
	▪ Facilitate access to support services (P00C43)<br>▪ Provide support to patients and families (P00C43)<br>• Assessors<br>○ Provide support to patients and families (P00C43)	▪ Identify existing supports available for patients (P00C43)<br>• Assessors<br>○ Identify existing supports available for patients (P00C43)<br>○ Provide support services to patients (P00C43)	before losing capacity to consent<br>○ Patient meets all requirements and safeguards for MAiD (149)<br>○ Patient enters into written agreement with physicians/nurse practitioners to receive MAiD on or before a specified day (149)<br>○ Patient is informed of risk of losing capacity to consent to receive MAiD prior to specified date (149)<br>• MAiD is provided if patient<br>○ Becomes incapable of consenting (149)<br>○ Shows no indication of declining MAiD (149)	final consent to proceed (146, 147)	○ Midazolam (2.5 mg - 10 mg)<br>○ Lidocaine (40 mg 2%) (150)<br>▪ Or Magnesium Sulphate (1000 mg dilute to 10mL with normal saline 0.9%)<br>○ Propofol (1000 mg) (150)<br>▪ Or Phenobarbital (3000 mg dilute to 50mL with normal saline 0.9%)<br>○ Rocuronium bromide (200 mg)<br>▪ Or Cisatracurium (30 mg) (150)		unavailable (P00C44)	unavailable (P00C44)	unavailable (P00C44)	• Community pharmacy (148)		○ Physicians/nurse practitioners (148)<br>• Cause of death<br>○ Medication administered due to medical conditions for requesting MAiD (142)<br>• Manner of death<br>○ Natural (142)	
Newfoundland and Labrador - *Western Zone*	Strategies<br>• MAiD team<br>○ Coordinators<br>▪ Facilitate access to support services (P00C44)<br>▪ Encourage patients to have conversatio ns with families (P00C44)	• MAiD team<br>○ Coordinators<br>▪ Identify existing supports available for patients (P00C44)	• Yes (149)<br>• If patient meets the following conditions before losing capacity to consent<br>○ Patient meets all requirements and safeguards for MAiD (149)<br>○ Patient enters into written agreement with physicians/nurse practitioners to receive MAiD on	• Patient must explicitly express their final consent to proceed (146, 147)	• Regional standardized medication protocol<br>○ Metoclopramide-Optional (10 mg) (151)<br>○ Midazolam (2.5 mg) (151)<br>○ Lidocaine (40 mg) (151)<br>○ Propofol (200 mg/ 20 mL) repeat for 5 doses (Propofol 1000 mg over 5 minutes) (151)	• Physicians/nurse practitioners (P00C44)	• MAiD medication protocol for self-administration is unavailable (P00C44)	• MAiD medication protocol for self-administration is unavailable (P00C44)	• MAiD medication protocol for self-administration is unavailable (P00C44)	• Hospital pharmacy (P00C44)<br>• Community pharmacy (P00C44)	• Yes (P00C44)	• Responsible professional<br>○ Physicians/nurse practitioners (148) (P00C40)<br>• Cause of death<br>○ Medication administered due to medical conditions for requesting MAiD (142) (P00C40)<br>• Manner of death<br>○ Natural (142) (P00C40)	• Patient's family (P00C44)<br>• Patients (P00C44)

Jurisdiction	Family dynamics		MAiD consent process		Medication protocol					Pharmacy involved		Post-provision arrangements	
					Intravenous		Oral						
	What are the strategies and guidelines MAiD team/assessors and providers utilize to address family dynamics	What supports are provided to patients who are alone?	Can final consent be waived for foreseeable natural death?	How are final consents provided?	What are the protocols for clinician-administered medication?	Which professionals are present for clinician administration?	What are the protocols for self-administered medication?	What are the processes in place if a self-administered medication fails?	Which professionals are present for self-administration?	Which pharmacies provide MAiD kit?	Does the pharmacy dispense a backup kit?	How is the death certificate completed?	Who is responsible for coordinating funeral arrangements?
	▪ Provide information on available resources to patients and families (P00C44)		or before a specified day (149)<br>○ Patient is informed of risk of losing capacity to consent to receive MAiD prior to specified date (149)<br>• MAiD is provided if patient<br>○ Becomes incapable of consenting (149)<br>○ Shows no indication of declining MAiD (149)		○ Rocuronium bromide (200 mg/20 mL) (151)<br>▪ Or Cisatracurium (20mg/10mL – 30 mg (15 mL))<br>○ Normal Saline (10 mL x 6 0.9%) (151)								
Newfoundland and Labrador - *Central Zone*	Strategies<br>• MAiD team<br>○ Coordinator (social worker)<br>▪ Facilitates access to support services (P00B22)	• MAiD team (P00B22)<br>○ Coordinator (social worker)<br>▪ Identifies existing supports available for patients (P00B22)<br>• Community/hospital social workers<br>○ Provide support services to patients (P00B22)<br>• Community nurse (P00B22)<br>○ Identifies existing supports available for patients (P00B22)	• Yes (149)<br>• If patient meets the following conditions before losing capacity to consent<br>○ Patient meets all requirements and safeguards for MAiD (149)<br>○ Patient enters into written agreement with physicians/nurse practitioners to receive MAiD on or before a specified day (149)<br>○ Patient is informed of risk of losing capacity to consent to receive MAiD prior to specified date (149)<br>• MAiD is provided if patient	• Patient must explicitly express their final consent to proceed (146, 147)	• Regional standardized medication protocol<br>○ Midazolam (2.5 mg - 10 mg) (152)<br>○ Lidocaine (40 mg 2%) (152)<br>▪ Or Magnesium Sulphate (1000 mg dilute to 10mL with normal saline 0.9%)<br>○ Propofol (1000 mg/ 100 mL) (152)<br>▪ Or Phenobarbital (120 mg/mL give 3000 mg dilute to 50mL with normal saline 0.9%)<br>○ Normal Saline (10 mL 0.9%) (148)<br>○ Rocuronium bromide (200 mg/20 mL) (152)<br>▪ Or Cisatracurium (30 mg / 15 mL)<br>○ Normal saline (10 mL 0.9%) (152)	• Physicians/nurse practitioners (P00B22)	• MAiD medication protocol for self-administration is unavailable (P00C44)	• MAiD medication protocol for self-administration is unavailable (P00C44)	• MAiD medication protocol for self-administration is unavailable (P00C44)	• Hospital pharmacy (148) (P00B22)<br>• Community pharmacy (148)	• Availability of backup kit not specified	• Responsible professional<br>○ Physicians/nurse practitioners (148)<br>• Cause of death<br>○ Medication administered due to medical conditions for requesting MAiD (142)<br>• Manner of death<br>○ Natural (142)	• Patient's family (P00B22)<br>• MAiD team (P00B22)<br>○ Coordinator (social worker) (P00B22)<br>• Community nurse (P00B22)

	Family dynamics		MAiD consent process		Medication protocol					Pharmacy involved		Post-provision arrangements	
					Intravenous		Oral						
Jurisdiction	What are the strategies and guidelines MAiD team/assessors and providers utilize to address family dynamics	What supports are provided to patients who are alone?	Can final consent be waived for foreseeable natural death?	How are final consents provided?	What are the protocols for clinician-administered medication?	Which professionals are present for clinician administration?	What are the protocols for self-administrated medication?	What are the processes in place if a self-administrated medication fails?	Which professionals are present for self-administration?	Which pharmacies provide MAiD kit?	Does the pharmacy dispense a backup kit?	How is the death certificate completed?	Who is responsible for coordinating funeral arrangements?
			○ Becomes incapable of consenting (149)<br>• Shows no indication of declining MAiD (149)										
Northwest Territories	Strategies<br>• MAiD team<br>○ Coordinator<br>▪ Facilitates access to support services (P00A3, P00C35)<br>▪ Encourages patients to have conversations with families (P00A2, P00C35)	• MAiD team (P00A3)<br>○ Coordinator (P00A3)<br>▪ Identifies existing supports available for patients (P00A3)<br>• Hospital social workers<br>○ Provide support services to inpatients (P00A2)<br>• Home care nurse (P00C35)<br>○ Provides support services to patients (P00C35)<br>• Holistic wellness advisor (P00C35)<br>○ Provides support services to patients (P00C35)<br>• Clergy/pastors (P00C35)<br>○ Provide support services to patients (P00C35)<br>• Mental health counsellors (P00C35)<br>○ Provide support services to	• Yes (11)<br>• If patient meets the following conditions before losing capacity to consent:<br>○ Patient meets all requirements and safeguards for MAiD (11)<br>○ Patient enters into written agreement with physicians/nurse practitioners to receive MAiD on or before a specified day (11)<br>○ Patient is informed of risk of losing capacity to consent to receive MAiD prior to specified date (11)<br>• MAiD is provided if patient:<br>○ Becomes incapable of consenting (11)<br>○ Shows no indication of declining MAiD (11)	• Written (11, 153)	• Territorial standardized medication protocol (P00A3) (11)<br>○ Midazolam (10mg) (154)<br>○ Lidocaine (40 mg) (154)<br>▪ Or Magnesium sulphate (1000mg)<br>○ Propofol (1000 mg) (154)<br>○ NaCl (10 mL) (optional) (154)<br>○ Rocuronium (200mg) (154)<br>▪ or Cisatracurium (30mg) (154)	• Physicians/nurse practitioners (11) (P00A3)<br>• Nurse to start IV (P00E24)	• Territorial standardized medication protocol<br>○ Metoclopramide (10mg)<br>○ Haloperidol (2mg)<br>○ Ondansetron (8mg)<br>○ Lorazepam (0.25-0.5mg) as needed<br>○ Coma-inducing compound<br>▪ Diazepam powder (1g),<br>▪ Digoxin tablets (50mg),<br>▪ Propranolol tablets (2g) (154)<br>▪ Morphine (15g) (optional)<br>• Potential medications for symptom management to be used in conjunction with self-administration<br>○ Haloperidol<br>○ Metoclopramide<br>○ Ondansetron<br>○ Glycopyrrolate<br>○ Scopolamine<br>○ Atropine<br>○ Midazolam<br>○ Lorazepam<br>○ Morphine<br>○ Hydromorphone<br>○ Fentanyl (P00A3) (154)	• If self-administration fails, provider can administer intravenously after an agreed upon amount of time (11)<br>○ If patient does not tolerate medication, provider administers through intravenous route (11)	• Physicians/nurse practitioners (154, 155)	• Hospital pharmacy (P00A2)<br>○ For hospital and community provisions (P00A2)	• Yes (P00A3)	• Responsible professional<br>○ Physicians/nurse practitioners (142)<br>• Cause of death<br>○ State "underlying medical condition" (P00A3)<br>• Manner of death<br>○ Natural (142)	• Patient's family (P00C35)<br>• MAiD team (P00A3, P00C35)<br>○ Coordinator (P00A3, P00C35)<br>• Hospital staff (P00B24)

Jurisdiction	Family dynamics		MAiD consent process		Medication protocol: Intravenous		Medication protocol: Oral			Pharmacy involved		Post-provision arrangements	
	What are the strategies and guidelines MAiD team/assessors and providers utilize to address family dynamics	What supports are provided to patients who are alone?	Can final consent be waived for foreseeable natural death?	How are final consents provided?	What are the protocols for clinician-administered medication?	Which professionals are present for clinician administration?	What are the protocols for self-administrated medication?	What are the processes in place if a self-administrated medication fails?	Which professionals are present for self-administration?	Which pharmacies provide MAiD kit?	Does the pharmacy dispense a backup kit?	How is the death certificate completed?	Who is responsible for coordinating funeral arrangements?
		patients (P00C35)											
Nova Scotia - *Nova Scotia Health*	Strategies<br>• MAiD team<br>○ Coordinators<br>▪ Facilitate access to support services (P00A12)<br>○ Social worker<br>▪ Provides support and counselling services to patients and families (P00A12)	• MAiD team (P00A12)<br>○ Social workers<br>▪ Provide support services to patients (P00A12)<br>• Ethicist (P00C38)<br>○ Identifies existing supports available for patients (P00C38)	• Yes (P00A12)<br>• If patient meets the following conditions before losing capacity to consent:<br>○ Patient meets all requirements and safeguards for MAiD (13, 14, 125)<br>○ Patient enters into written agreement with physicians/nurse practitioners to receive MAiD on or before a specified day (13, 14, 125)<br>○ Patient is informed of risk of losing capacity to consent to receive MAiD prior to specified date (13, 14, 125)<br>• MAiD is provided if patient:<br>○ Becomes incapable of consenting (13, 14, 125)<br>○ Shows no indication of declining MAiD (13, 14, 125)	• Verbal (P00C38)	• Provincial standardized medication protocol (P00A12)<br>○ Midazolam (20 mg)<br>○ Lidocaine 2% (40 mg)<br>▪ Or Magnesium Sulfate (1000 mg)<br>○ Propofol (1000 mg)<br>▪ Or Phenobarbital (3000 mg over 5 minutes),<br>○ NaCl 0.9% (10 ml),<br>○ Rocuronium (200 mg)<br>▪ Or Cistaracurium (30 mg)<br>○ NaCl 0.9% (10 mL) (19)<br>• Extra tubing may be used so that clinician can sit behind patients and families can gather around (P00A12)	• Physicians/nurse practitioners (P00A12)<br>• Registered nurse<br>○ Present if patient and clinician prefer (P00A12)	• MAiD medication for self-administration is unavailable (P00A12, P00A6)	• MAiD medication protocol for self-administration is unavailable (P00A12, P00A6)	• MAiD medication protocol for self-administration is unavailable (P00A12, P00A6)	• Hospital pharmacy (P00A6)<br>• Community pharmacy (P00A6)	• For IV protocol one additional kit is provided (156)	• Responsible professional<br>○ Physicians/nurse practitioners (P00A6, P00C38)<br>• Cause of death<br>○ Injection of drugs due to underlying illness, disease or disability (142)<br>• Manner of death<br>○ Natural (142)	• Patient's family (P00A12)<br>• Patient (P00A12)<br>• MAiD team (P00A12)<br>○ Social workers (P00A12)
Prince Edward Island - *Health PEI*	Strategies<br>• MAiD team<br>○ Coordinator<br>▪ Facilitates access to support services (P00B17)	• MAiD team (P00B17)<br>○ Coordinator (nurse navigator)<br>▪ Identifies existing	• Yes (23, 53)<br>• If patient meets the following conditions before losing capacity to consent:<br>○ Patient meets all requirements and safeguards for	• Verbal (P00B17)<br>• Written (P00B8)	• Provincial standardized medication protocol (P00B8)<br>• Medications include:<br>○ Midazolam<br>○ Lidocaine<br>○ Propofol<br>○ NaCl 0.9%	• Physicians/nurse practitioners (P00B8)<br>• MAiD team (P00B8)<br>○ Coordinator (nurse	• MAiD medication for self-administration is unavailable (P00B8)	• MAiD medication for self-administration is unavailable (P00B8)	• MAiD medication for self-administration is unavailable (P00B8)	• Hospital pharmacy (P00B8, P00B17)	• Yes<br>○ Must return kit if not used (P00B17)	• Responsible professional<br>○ Physicians/nurse practitioners (P00B17)<br>• Cause of death<br>○ Medications administered due	• Patient's family (P00B17)<br>• Patient (P00B17)<br>• Physicians/nurse practitioners (P00C25)<br>• MAiD team

					Medication protocol								
	Family dynamics		MAiD consent process		Intravenous		Oral			Pharmacy involved		Post-provision arrangements	
Jurisdiction	What are the strategies and guidelines MAiD team/assessors and providers utilize to address family dynamics	What supports are provided to patients who are alone?	Can final consent be waived for foreseeable natural death?	How are final consents provided?	What are the protocols for clinician-administered medication?	Which professionals are present for clinician administration?	What are the protocols for self-administrated medication?	What are the processes in place if a self-administrated medication fails?	Which professionals are present for self-administration?	Which pharmacies provide MAiD kit?	Does the pharmacy dispense a backup kit?	How is the death certificate completed?	Who is responsible for coordinating funeral arrangements?
	▪ Encourages patients to have conversations with families (P00B17)<br>○ Social worker<br>▪ Provides support and counselling services to patients and families (P00B8)	supports available for patients (P00B17)<br>○ Social worker (P00B8)<br>▪ Provides support services to patients (P00B17)	MAiD (23, 53)<br>○ Patient enters into written agreement with physician/nurse practitioner to receive MAiD on or before a specified day (23, 53)<br>○ Patient is informed of risk of losing capacity to consent to receive MAiD prior to specified date (23, 53)		○ Rocuronium<br>• Bupivacaine if a delayed cardio-respiratory risk is a concern (P00B8)<br>• Inserts 2 IVs either night before or on day of provision (P00B17)<br>• If IV insertions are difficult a PICC line is inserted (P00B17)	navigators) (P00B8)<br>○ Social workers (P00B8)						to medical condition that led to MAiD request (P00B17)<br>• Manner of death<br>○ No indication (P00B17)	○ Social worker (P00B17)
Saskatchewan - *Saskatchewan Health Authority*	Strategies<br>• MAiD team<br>○ Social workers<br>▪ Provide support and counselling services to patients and families (P00A1, P00B1)	• MAiD team (P00B7)<br>○ Social workers (P00A1)<br>▪ Provide support services to patients (P00A1)	• Yes (P00C1)<br>• If patient meets the following conditions before losing capacity to consent:<br>○ Patient meets all requirements and safeguards for MAiD (P00C1)<br>○ Patient enters into written agreement with physician/nurse practitioner to receive MAiD on or before a specified day (P00C1)	• Verbal (157)<br>• Physical gestures (157)<br>• Alternatives to consent when unable:<br>○ Visual boards (P00C1)<br>○ Eye movement (P00A1)<br>○ Facial movements (P00C1)	• Provincial standardized medication protocol (P00B5)<br>○ Midazolam (P00B5)<br>○ IV lidocaine (2ml) (P00C19)<br>○ Propofol (2 vials)<br>○ Rocuronium (4 vials) (P00B5)<br>○ EMLA on skin (P00B5)	• Physicians/nurse practitioners (88)<br>• Registered nurse (P00B1)<br>• MAiD team<br>○ Social worker (P00A1)	• MAiD medication for self-administration is unavailable (P00B3, P00B1, P00C19)	• MAiD medication for self-administration is unavailable (P00B3, P00B1, P00C19)	• MAiD medication for self-administration is unavailable (P00B3, P00B1, P00C19)	• Hospital pharmacy (P00B5)<br>• Remote pharmacy (P00B5)<br>○ Virtual dispensing is used for remote communities (P00B5)	• Yes<br>○ Must return kit if not used (P00C1, P00B5)	• Responsible professional<br>○ Physicians/nurse practitioners (P00C1)<br>• Cause of death<br>○ Drug toxicity (due to) MAiD (due to) underlying illness, disease or disability (142)<br>• Manner of death<br>○ Unclassified (P00B1, P00C19)	• Patient's family (P00B18)<br>• Patient (P00B18, P00C1)
Yukon - *Department of Health and Social Services*	Strategies<br>• Assessors and Providers<br>○ Facilitate access to support services (P00C7, P00C14)<br>○ Encourage patients to	• Community/hospital social workers<br>○ Provide support services to patients (P00C7, P00C14)	• Yes (P00B4)<br>• If patient meets the following conditions before losing capacity to consent:<br>○ Patient meets all requirements and safeguards for MAiD (90, 109)<br>○ Patient enters into written	• Verbal (90)	• Territorial standardized medication protocol (19)<br>○ Midazolam (10 mg)<br>○ Lidocaine (40 mg)<br>○ Magnesium Sulfate (1000 mg)<br>○ Propofol (1000 mg)<br>○ Phenobarbital (3000 mg)<br>○ NaCl 0.9% (10 ml)	• Physicians/nurse practitioners (15, 90, 109)	• Territorial standardized medication protocol (19)<br>○ Metoclopramide (2mg)<br>○ Haloperidol (2mg)<br>○ Lorazepam (0.25-0.5mg) as needed<br>○ Coma-inducing compound	• Advanced Consent for Failed Self-Administration is obtained<br>○ Written agreement between patient and provider (109)<br>○ If patient loses	• Physician or nurse practitioner (15, 109, 158)<br>○ Must be present until death is confirmed	• Hospital pharmacy (P00C7)<br>○ For hospital and community provisions (P00C14)	• Availability of backup kit not specified	• Responsible professional<br>○ Physicians/nurse practitioners (P00C7, P00C14)<br>• Cause of death<br>○ Underlying illness, disease or disability (142)<br>• Manner of death<br>○ MAiD (142)	• Patient's family (P00C7)<br>• Physicians/nurse practitioners (P00C7)<br>• Registered nurse (P00C7)

Jurisdiction	Family dynamics		MAiD consent process		Medication protocol					Pharmacy involved		Post-provision arrangements	
					Intravenous		Oral						
	What are the strategies and guidelines MAiD team/assessors and providers utilize to address family dynamics	What supports are provided to patients who are alone?	Can final consent be waived for foreseeable natural death?	How are final consents provided?	What are the protocols for clinician-administered medication?	Which professionals are present for clinician administration?	What are the protocols for self-administered medication?	What are the processes in place if a self-administered medication fails?	Which professionals are present for self-administration?	Which pharmacies provide MAiD kit?	Does the pharmacy dispense a backup kit?	How is the death certificate completed?	Who is responsible for coordinating funeral arrangements?
	have conversations with families (P00C7, P00C14)		agreement with physician/nurse practitioner to receive MAiD on or before a specified day (90, 109)<br>○ Patient is informed of risk of losing capacity to consent to receive MAiD prior to specified date (90, 109)<br>• MAiD is provided if patient:<br>○ Becomes incapable of consenting (90, 109)<br>○ Shows no indication of declining MAiD (90, 109)		○ Rocuronium (200 mg)<br>○ Cistaracurium (30 mg) (19)		▪ Diazepam (1mg)<br>▪ Digoxin tablets (50mg)<br>▪ Propranolol (2g)<br>▪ Morphine (15g) (19)	capacity to consent, provider can administer intravenously after an agreed upon amount of time (109)					
Ontario - *Ministry of Health*	Strategies<br>• MAiD team<br>○ Coordinators<br>▪ Facilitate access to support services (P00C16)<br>▪ Encourage patients to have conversations with families (P00C16)	• Not applicable	• Yes (P00B4)<br>• If patient meets the following conditions before losing capacity to consent:<br>○ Patient meets all requirements and safeguards for MAiD (24)<br>○ Patient enters into written agreement with physicians/nurse practitioners to receive MAiD on or before a specified day (24)<br>○ Patient is informed of risk of losing capacity to consent to receive MAiD	• Verbal (P00C16)	• No provincial standardized medication protocol (P00C16)<br>• Common medication includes<br>○ Midazolam (1mg/mL) (159)<br>○ Lidocaine 2% (159)<br>○ Magnesium sulfate (500mg/mL) (159)<br>○ Propofol (10mg)<br>▪ Or Phenobarbital (120mg/mL) (159)<br>○ Rocuronium bromide (10mg/mL)<br>▪ Or Cisatracurium besylate (2mg/mL) (P00C16) (159)	• Physicians/nurse practitioners (2) (P00B4)	• No provincial standardized medication protocol (P00C16)<br>• Common medication includes<br>○ Metoclopramide (10mg) (159)<br>○ Ondansetron (8mg) (159)<br>○ Propranolol (40mg) (159)<br>○ Morphine sulfate (30mg) (159)<br>○ Hydromorphone (1mg/mL) (159)<br>○ Hydromorphone (8mg) (159)	• Advanced Consent for Failed Self-Administration is obtained<br>○ A written agreement between patient and provider (P00C16)<br>○ If patient loses capacity to consent, provider can administer intravenously after an agreed upon amount of time (P00C16)	• No legal requirement that mandates provider's presence when patient consumes medications, unless an Advanced Consent for Failed Administration is in place (P00C16)	• Hospital pharmacy (P00C16)<br>• Community pharmacy (P00C16)	• Yes (159)	• Responsible professional<br>○ Physicians/nurse practitioners (142)<br>• Cause of death<br>○ Underlying illness, disease or disability (160)<br>• Manner of death<br>○ Natural (142)	• Patient's family (P00C16)<br>• Provider (P00C16)<br>○ Connects patients to funeral home (P00C16)

	Family dynamics		MAiD consent process		Medication protocol: Intravenous		Medication protocol: Oral			Pharmacy involved		Post-provision arrangements	
**Jurisdiction**	**What are the strategies and guidelines MAiD team/assessors and providers utilize to address family dynamics**	**What supports are provided to patients who are alone?**	**Can final consent be waived for foreseeable natural death?**	**How are final consents provided?**	**What are the protocols for clinician-administered medication?**	**Which professionals are present for clinician administration?**	**What are the protocols for self-administrated medication?**	**What are the processes in place if a self-administrated medication fails?**	**Which professionals are present for self-administration?**	**Which pharmacies provide MAiD kit?**	**Does the pharmacy dispense a backup kit?**	**How is the death certificate completed?**	**Who is responsible for coordinating funeral arrangements?**
			prior to specified date (24)<br>• MAiD is provided if patient:<br>○ Becomes incapable of consenting (24)<br>○ Shows no indication of declining MAiD (24)										
SERVICE ORGANIZATIONS/REGIONAL FACILITIES													
Ontario - *Home and Community Care Support Services, Central East*	Strategies<br>• MAiD team<br>○ Coordinators<br>▪ Facilitate access to support services (P00C2)<br>• Providers<br>○ Provide information on available resources to patients and families (P00C2)	• MAiD team (P00C2)<br>○ Coordinators<br>▪ Identify existing supports available for patients (P00C2)	• Yes (P00C2)<br>• If patient meets the following conditions before losing capacity to consent:<br>○ Patient meets all requirements and safeguards for MAiD (24)<br>○ Patient enters into written agreement with physicians/nurse practitioners to receive MAiD on or before a specified day (24)<br>○ Patient is informed of risk of losing capacity to consent to receive MAiD prior to specified date (24)<br>• MAiD is provided if patient:<br>○ Becomes incapable of consenting (24)<br>○ Shows no indication of declining MAiD (24)	• Verbal (P00C2)	• CAMAP medication protocol (19) (P00C2)	• Physicians/nurse practitioners (P00C2)<br>• Registered nurse (P00C2)	• Not applicable<br>○ MAiD medication protocol for self-administration is unavailable (P00C2)	• Not applicable<br>○ MAiD medication protocol for self-administration is unavailable (P00C2)	• Not applicable<br>○ MAiD medication protocol for self-administration is unavailable (P00C2)	• Community pharmacy (P00C2)	• Availability of backup kit not specified	• Responsible professional<br>○ Physicians/nurse practitioners (142) (P00C2)<br>• Cause of death<br>○ Underlying illness, disease or disability (160)<br>• Manner of death<br>○ Natural (142)	• Patient's family (P00C2)<br>• Patient (P00C2)<br>• Physicians/nurse practitioners (P00C2)

Jurisdiction	Family dynamics: What are the strategies and guidelines MAiD team/assessors and providers utilize to address family dynamics	Family dynamics: What supports are provided to patients who are alone?	MAiD consent process: Can final consent be waived for foreseeable natural death?	MAiD consent process: How are final consents provided?	Medication protocol – Intravenous: What are the protocols for clinician-administered medication?	Medication protocol – Intravenous: Which professionals are present for clinician administration?	Medication protocol – Oral: What are the protocols for self-administrated medication?	Medication protocol – Oral: What are the processes in place if a self-administrated medication fails?	Medication protocol – Oral: Which professionals are present for self-administration?	Pharmacy involved: Which pharmacies provide MAiD kit?	Pharmacy involved: Does the pharmacy dispense a backup kit?	Post-provision arrangements: How is the death certificate completed?	Post-provision arrangements: Who is responsible for coordinating funeral arrangements?
Ontario - *Home and Community Care Support Services, Waterloo Region*	Strategies<br>• Providers<br>○ Encourage patients to have conversations with families (P00C4)	• Supports for alone patients not specified	• Yes (P00C4)<br>• If patient meets the following conditions before losing capacity to consent:<br>○ Patient meets all requirements and safeguards for MAiD (24)<br>○ Patient enters into written agreement with physicians/nurse practitioners to receive MAiD on or before a specified day (24)<br>○ Patient is informed of risk of losing capacity to consent to receive MAiD prior to specified date (24)<br>• MAiD is provided if patient:<br>○ Becomes incapable of consenting (24)<br>○ Shows no indication of declining MAiD (24)	• Verbal (P00C4)	• Protocols for clinician-administered medication not specified	• Physicians/nurse practitioners (P00C4)	• Not applicable<br>○ MAiD medication protocol for self-administration is unavailable (P00C4)	• Not applicable<br>○ MAiD medication protocol for self-administration is unavailable (P00C4)	• Not applicable<br>○ MAiD medication protocol for self-administration is unavailable (P00C4)	• Hospital pharmacy (P00C4)<br>• Community pharmacy (P00C4)	• Availability of backup kit not specified	• Responsible professional<br>○ Physicians/nurse practitioners (142) (P00C4)<br>• Cause of death<br>○ Underlying illness, disease or disability (160)<br>• Manner of death<br>○ Natural (142)	• Patient's family (P00C4)
Ontario - *Home and Community Care Support Services, South East*	Strategies<br>• Providers<br>○ Encourage patients to have conversations with families (P00C20)	• No experience (P00C20)	• Yes (P00C20)<br>• If patient meets the following conditions before losing capacity to consent:<br>○ Patient meets all requirements and safeguards for MAiD (24)<br>○ Patient enters into written agreement with physicians/nurse	• Written (P00C20)<br>• Verbal (P00C20)	• CAMAP medication protocol (19) (P00C20)	• Physicians/nurse practitioners<br>• Additional healthcare professional to act as witness (P00C20)	• Not applicable<br>○ No experience with MAiD medication protocol for self-administration (P00C20)	• Not applicable<br>○ No experience with MAiD medication protocol for self-administration (P00C20)	• Not applicable<br>○ No experience with MAiD medication protocol for self-administration (P00C20)	• Hospital pharmacy (P00C20)<br>• Community pharmacy (P00C20)	• Yes<br>○ Varies depending on provider (P00C20)	• Responsible professional<br>○ Physicians/nurse practitioners (142) (P00C20)<br>• Cause of death<br>○ Underlying illness, disease or disability (160)<br>• Manner of death<br>○ Natural (142)	• Patient's family (P00C20)

Jurisdiction	Family dynamics: What are the strategies and guidelines MAiD team/assessors and providers utilize to address family dynamics	Family dynamics: What supports are provided to patients who are alone?	MAiD consent process: Can final consent be waived for foreseeable natural death?	MAiD consent process: How are final consents provided?	Medication protocol – Intravenous: What are the protocols for clinician-administered medication?	Medication protocol – Intravenous: Which professionals are present for clinician administration?	Medication protocol – Oral: What are the protocols for self-administrated medication?	Medication protocol – Oral: What are the processes in place if a self-administrated medication fails?	Medication protocol – Oral: Which professionals are present for self-administration?	Pharmacy involved: Which pharmacies provide MAiD kit?	Pharmacy involved: Does the pharmacy dispense a backup kit?	Post-provision arrangements: How is the death certificate completed?	Post-provision arrangements: Who is responsible for coordinating funeral arrangements?
			practitioners to receive MAiD on or before a specified day (24)<br>○ Patient is informed of risk of losing capacity to consent to receive MAiD prior to specified date (24)<br>• MAiD is provided if patient:<br>○ Becomes incapable of consenting (24)<br>○ Shows no indication of declining MAiD (24)										
Ontario - *Home and Community Care Support Services, South West*	Strategies<br>• Providers<br>○ Encourage patients to have conversations with families (P00C23)	• Providers<br>○ Provide support services to patients (P00C23)	• Yes (24)<br>• If patient meets the following conditions before losing capacity to consent:<br>○ Patient meets all requirements and safeguards for MAiD (24)<br>○ Patient enters into written agreement with physicians/nurse practitioners to receive MAiD on or before a specified day (24)<br>○ Patient is informed of risk of losing capacity to consent to receive MAiD prior to specified date (24)<br>• MAiD is provided if patient:	• Verbal (P00C23)	• Medication varies among providers (P00C16)<br>• Medications used include:<br>○ Midazolam<br>○ Propofol<br>○ Rocuronium<br>▪ Or Cisatracurium (P00C16) (19)<br>○ Lidocaine is not used due to provider preference and observation that patients are not anxious (P00C16)	• Physicians/nurse practitioners (P00C23)<br>○ Provider hires a private IV nurse (P00C23)	• Not applicable<br>○ No experience with MAiD medication protocol for self-administration (P00C23)	• Not applicable<br>○ No experience with MAiD medication protocol for self-administration (P00C23)	• Not applicable<br>○ No experience with MAiD medication protocol for self-administration (P00C23)	• Community pharmacy (P00C23)<br>○ Pharmacy delivers medications to patient's home in advance (P00C23)	• No (P00C23)	• Responsible professional<br>○ Physicians/nurse practitioners (142) (P00C23)<br>• Cause of death<br>○ Underlying illness, disease or disability (160)<br>• Manner of death<br>○ Natural (142)	• Patient's family (P00C23)<br>• Patient (P00C23)<br>• Appointed executor (P00C23)

	Medication protocol													
	Family dynamics		MAiD consent process		Intravenous		Oral			Pharmacy involved		Post-provision arrangements		
Jurisdiction	What are the strategies and guidelines MAiD team/assessors and providers utilize to address family dynamics	What supports are provided to patients who are alone?	Can final consent be waived for foreseeable natural death?	How are final consents provided?	What are the protocols for clinician-administered medication?	Which professionals are present for clinician administration?	What are the protocols for self-administrated medication?	What are the processes in place if a self-administrated medication fails?	Which professionals are present for self-administration?	Which pharmacies provide MAiD kit?	Does the pharmacy dispense a backup kit?	How is the death certificate completed?	Who is responsible for coordinating funeral arrangements?
			○ Becomes incapable of consenting (24)<br>○ Shows no indication of declining MAiD (24)										
Ontario - *Champlain Regional MAiD Network, The Ottawa Hospital*	Strategies<br>• MAiD team<br>○ Coordinators<br>▪ Facilitate access to support services (P00C5)	• MAiD team (P00C5)<br>○ Coordinators<br>▪ Identify existing supports available for patients (P00C5)<br>• Hospital social workers (P00C5)<br>○ Provide support services to inpatients (P00C5)<br>• Hospital registered nurses<br>○ Provide support services to patients (P00C5)	• Yes (P00C5)<br>• If patient meets the following conditions before losing capacity to consent:<br>○ Patient meets all requirements and safeguards for MAiD (24)<br>○ Patient enters into written agreement with physicians/nurse practitioners to receive MAiD on or before a specified day (24)<br>○ Patient is informed of risk of losing capacity to consent to receive MAiD prior to specified date (24)<br>• MAiD is provided if patient:<br>○ Becomes incapable of consenting (24)<br>○ Shows no indication of declining MAiD (24)	• Verbal (P00C5)	• Medication includes<br>○ Midazolam<br>○ Lidocaine<br>○ Propofol<br>○ Recuronium<br>▪ or Bupivacaine<br>• Protocol differs slightly in case of organ donation	• Physicians/nurse practitioners (P00C5)	• Not applicable<br>○ MAiD medication protocol for self-administration is unavailable (P00C5)	• Not applicable<br>○ MAiD medication protocol for self-administration is unavailable (P00C5)	• Not applicable<br>○ MAiD medication protocol for self-administration is unavailable (P00C5)	• Hospital pharmacy (P00C5)<br>• Community pharmacy (P00C5)	• No (P00C5)	• Responsible professional<br>○ Physicians/nurse practitioners (142) (P00C5)<br>• Cause of death<br>○ Underlying illness, disease or disability (160)<br>• Manner of death<br>○ Natural (142)	• Patient's family (P00C5)<br>• Hospital social worker (P00C5)
HEALTHCARE FACILITIES													
Ontario - *Peterborough Regional Health Centre*	Strategies<br>• MAiD team<br>○ Social worker<br>▪ Provides support and	• MAiD team (P00C3)<br>○ Social worker<br>▪ Provides support	• Yes (P00C3)<br>• If patient meets the following conditions before losing capacity to consent:	• Final consents not specified	• Protocols for clinician-administered medication not specified	• Physicians/nurse practitioners (P00C3)<br>• Registered nurse	• Access to MAiD medication for self-administration is prohibited in this institution (P00C3)	• Not applicable<br>○ Access to MAiD medication for self-administration	• Not applicable<br>○ Access to MAiD medication for self-administration	• Hospital pharmacy (P00C3)	• Availability of backup kit not specified	• Responsible professional<br>○ Physicians/nurse practitioners (142) (P00C3)	• Patient's family (P00C3)<br>• MAiD team (P00C3)<br>○ Social worker (P00C3)

Jurisdiction	Family dynamics: What are the strategies and guidelines MAiD team/assessors and providers utilize to address family dynamics	Family dynamics: What supports are provided to patients who are alone?	MAiD consent process: Can final consent be waived for foreseeable natural death?	MAiD consent process: How are final consents provided?	Medication protocol – Intravenous: What are the protocols for clinician-administered medication?	Medication protocol – Intravenous: Which professionals are present for clinician administration?	Medication protocol – Oral: What are the protocols for self-administrated medication?	Medication protocol – Oral: What are the processes in place if a self-administrated medication fails?	Medication protocol – Oral: Which professionals are present for self-administration?	Pharmacy involved: Which pharmacies provide MAiD kit?	Pharmacy involved: Does the pharmacy dispense a backup kit?	Post-provision arrangements: How is the death certificate completed?	Post-provision arrangements: Who is responsible for coordinating funeral arrangements?
	counselling services (P00C3)<br>• Providers<br>○ Encourage patients to have conversations with families (P00C3)	services to patients (P00C3)	○ Patient meets all requirements and safeguards for MAiD (24)<br>○ Patient enters into written agreement with physicians/nurse practitioners to receive MAiD on or before a specified day (24)<br>○ Patient is informed of risk of losing capacity to consent to receive MAiD prior to specified date (24)<br>• MAiD is provided if patient:<br>○ Becomes incapable of consenting (24)<br>○ Shows no indication of declining MAiD (24)			○ Establishes intravenous line, if needed (P00C3)		is prohibited in this institution (P00C3)	is prohibited in this institution (P00C3)	• Community pharmacy (P00C3)		• Cause of death<br>○ Underlying illness, disease or disability (160)<br>• Manner of death<br>○ Natural (142)	
Ontario - *Mount Sinai Healthcare Facility, Toronto*	Strategies<br>• Providers<br>○ Facilitate access to support services (P00C36)<br>○ Encourage patients to have conversations with families (P00C36)	• Providers<br>○ Identify existing supports available for patients (P00C36)	• Yes (24)<br>• If patient meets the following conditions before losing capacity to consent:<br>○ Patient meets all requirements and safeguards for MAiD (24)<br>○ Patient enters into written agreement with physicians/nurse practitioners to receive MAiD on or before a specified day (24)<br>○ Patient is	• Final consents not specified	• No standardized protocol (P00C36)<br>• Medication varies (P00C36)	• Physicians/nurse practitioners (P00C36)	• Not applicable<br>○ MAiD medication protocol for self-administration is unavailable (P00C36)	• Not applicable<br>○ MAiD medication protocol for self-administration is unavailable (P00C36)	• Not applicable<br>○ MAiD medication protocol for self-administration is unavailable (P00C36)	• Hospital pharmacy (P00C36)	• Availability of backup kit not specified	• Responsible professional<br>○ Physicians/nurse practitioners (142)<br>• Cause of death<br>○ Underlying illness, disease or disability (160)<br>• Manner of death<br>○ Natural (142)	• Responsibility for funeral arrangements not specified

	Family dynamics		MAiD consent process		Medication protocol					Pharmacy involved		Post-provision arrangements	
					Intravenous		Oral						
Jurisdiction	What are the strategies and guidelines MAiD team/assessors and providers utilize to address family dynamics	What supports are provided to patients who are alone?	Can final consent be waived for foreseeable natural death?	How are final consents provided?	What are the protocols for clinician-administered medication?	Which professionals are present for clinician administration?	What are the protocols for self-administrated medication?	What are the processes in place if a self-administrated medication fails?	Which professionals are present for self-administration?	Which pharmacies provide MAiD kit?	Does the pharmacy dispense a backup kit?	How is the death certificate completed?	Who is responsible for coordinating funeral arrangements?
			informed of risk of losing capacity to consent to receive MAiD prior to specified date (24)<br>• MAiD is provided if patient:<br>○ Becomes incapable of consenting (24)<br>○ Shows no indication of declining MAiD (24)										
Ontario - *University Health Network*	Strategies<br>• MAiD team<br>○ Coordinators<br>▪ Facilitate access to support services (P00C8)	• MAiD team (P00C8)<br>○ Coordinators (P00C8)<br>▪ Identify existing supports available for patients (P00C8)<br>• Hospital social workers<br>○ Provide support services to inpatients (P00C8)	• Yes (P00C8)<br>• If patient meets the following conditions before losing capacity to consent:<br>○ Patient meets all requirements and safeguards for MAiD (24)<br>○ Patient enters into written agreement with physicians/nurse practitioners to receive MAiD on or before a specified day (24)<br>○ Patient is informed of risk of losing capacity to consent to receive MAiD prior to specified date (24)<br>• MAiD is provided if patient:<br>○ Becomes incapable of consenting (24)<br>○ Shows no indication of	• Verbal (P00C8)<br>• Written (P00C8)	• Medication protocol<br>○ Midazolam<br>○ Lidocaine<br>○ Propofol<br>○ Rocuronium (P00C8)	• Physicians/nurse practitioners (P00C8)<br>• Registered nurse (P00C8)<br>○ Establishes intravenous line (P00C8)<br>○ Prepares, and collects medications (P00C8)	• Not applicable<br>○ MAiD medication protocol for self-administration is unavailable (P00C8)	• Not applicable<br>○ MAiD medication protocol for self-administration is unavailable (P00C8)	• Not applicable<br>○ MAiD medication protocol for self-administration is unavailable (P00C8)	• Hospital pharmacy (P00C8)	• Yes (P00C8)	• Responsible professional<br>○ Physicians/nurse practitioners (142) (P00C8)<br>• Cause of death<br>○ Underlying illness, disease or disability (160)<br>• Manner of death<br>○ Natural (142)	• Patient's family (P00C8)<br>• Patient (P00C8)

	Family dynamics		MAiD consent process		Medication protocol					Pharmacy involved		Post-provision arrangements	
					Intravenous		Oral						
**Jurisdiction**	**What are the strategies and guidelines MAiD team/assessors and providers utilize to address family dynamics**	**What supports are provided to patients who are alone?**	**Can final consent be waived for foreseeable natural death?**	**How are final consents provided?**	**What are the protocols for clinician-administered medication?**	**Which professionals are present for clinician administration?**	**What are the protocols for self-administrated medication?**	**What are the processes in place if a self-administrated medication fails?**	**Which professionals are present for self-administration?**	**Which pharmacies provide MAiD kit?**	**Does the pharmacy dispense a backup kit?**	**How is the death certificate completed?**	**Who is responsible for coordinating funeral arrangements?**
			declining MAiD (24)										
Ontario - *Grand River Hospital*	Strategies<br>• MAiD team<br>○ Coordinator<br>▪ Facilitates access to support services (P00C50)	• MAiD team (P00C50)<br>○ Coordinator<br>▪ Identifies existing supports available for patients (P00C50)<br>• Hospital social workers<br>○ Provide support services to inpatients (P00C50)	• Yes (24)<br>• If patient meets the following conditions before losing capacity to consent:<br>○ Patient meets all requirements and safeguards for MAiD (24)<br>○ Patient enters into written agreement with physicians/nurse practitioners to receive MAiD on or before a specified day (24)<br>○ Patient is informed of risk of losing capacity to consent to receive MAiD prior to specified date (24)<br>• MAiD is provided if patient:<br>○ Becomes incapable of consenting (24)<br>○ Shows no indication of declining MAiD (24)	• Written (P00C50)	• Medication protocol<br>○ Midazolam (5mg)<br>○ Lidocaine<br>▪ or Magnesium sulfate<br>○ Propofol<br>▪ Phenobarbital<br>○ Rocuronium (200mg)<br>▪ Cisatracurium (30mg) (P00C50)	• Physicians/nurse practitioners (P00C50)	• Not applicable<br>○ MAiD medication protocol for self-administration is unavailable (P00C50)	• Not applicable<br>○ MAiD medication protocol for self-administration is unavailable (P00C50)	• Not applicable<br>○ MAiD medication protocol for self-administration is unavailable (P00C50)	• Hospital pharmacy (P00C50)	• Availability of backup kit not specified	• Responsible professional<br>○ Physicians/nurse practitioners (142) (P00C50)<br>• Cause of death<br>○ Underlying illness, disease or disability (160)<br>• Manner of death<br>○ Natural (142)	• Patient's family (P00C50)<br>• MAiD team (P00C50)<br>○ Coordinator (P00C50)
COMMUNITY OF PRACTICE													
Ontario - *Hamilton Family Health Team*	Strategies<br>• MAiD team<br>○ Coordinators<br>▪ Encourage patients to	• MAiD team (P00B18, P00C32)<br>○ Coordinators<br>▪ Identify existing	• Yes (P00B32)<br>• If patient meets the following conditions before losing capacity to consent:	• Written (P00C32)	• Medication protocol<br>○ Midazolam (20mg) optional based on preference<br>○ Lidocaine 2%	• Physicians/nurse practitioners (P00B18)	• Not applicable<br>○ MAiD medication protocol for self-administration is	• Not applicable<br>○ MAiD medication protocol for self-	• Not applicable<br>○ MAiD medication protocol for self-	• Community pharmacy (P00C32)	• Availability of backup kit not specified	• Responsible professional<br>○ Physicians/nurse practitioners (142) (P00B18)	• Patient's family (P00B18)<br>• Patient (P00B18)

Jurisdiction	Family dynamics: What are the strategies and guidelines MAiD team/assessors and providers utilize to address family dynamics	Family dynamics: What supports are provided to patients who are alone?	MAiD consent process: Can final consent be waived for foreseeable natural death?	MAiD consent process: How are final consents provided?	Medication protocol – Intravenous: What are the protocols for clinician-administered medication?	Medication protocol – Intravenous: Which professionals are present for clinician administration?	Medication protocol – Oral: What are the protocols for self-administered medication?	Medication protocol – Oral: What are the processes in place if a self-administered medication fails?	Medication protocol – Oral: Which professionals are present for self-administration?	Pharmacy involved: Which pharmacies provide MAiD kit?	Pharmacy involved: Does the pharmacy dispense a backup kit?	Post-provision arrangements: How is the death certificate completed?	Post-provision arrangements: Who is responsible for coordinating funeral arrangements?
	have conversations with families (P00B18, P00C32)<br>▪ Provide information on available resources to patients and families (P00B18, P00C32)	supports available for patients (P00B18, P00C32)	○ Patient meets all requirements and safeguards for MAiD (24)<br>○ Patient enters into written agreement with physicians/nurse practitioners to receive MAiD on or before a specified day (24)<br>○ Patient is informed of risk of losing capacity to consent to receive MAiD prior to specified date (24)<br>• MAiD is provided if patient:<br>○ Becomes incapable of consenting (24)<br>○ Shows no indication of declining MAiD (24)		(Optional based on preference)<br>○ Magnesium Sulfate<br>○ Propofol (1000mg) (most commonly used)<br>▪ Or Phenobarbital (3000mg)<br>○ Rocuronium (200mg) (P00C32)<br>• Coordinators establish intravenous routes the day before or morning of provision<br>• Home care team establishes intravenous routes (P00C32)		unavailable (P00B18)	administration is unavailable (P00B18)	administration is unavailable (P00B18)	• Hospital pharmacy (P00C32)		• Cause of death<br>○ Underlying illness, disease or disability (160)<br>• Manner of death<br>○ Natural (142)	
Ontario - *Niagara Community MAiD Team, St. Catharines, Niagara*	Strategies<br>• Providers<br>○ Encourage patients to have conversations with families (P00C11)	• No experience (P00C11)	• Yes (P00C11)<br>• If patient meets the following conditions before losing capacity to consent:<br>○ Patient meets all requirements and safeguards for MAiD (24)<br>○ Patient enters into written agreement with physicians/nurse practitioners to receive MAiD on or before a specified day (24)<br>○ Patient is	• Verbal (P00C11)	• Medications used include<br>○ Midazolam (10mL)<br>○ Lidocaine (not used by all providers)<br>○ Propofol<br>○ Rocuronium<br>○ Bupivacaine is optional (P00C11)<br>• 2 IVs placed by provider to avoid an IV going interstitial (P00C11)	• Physicians/nurse practitioners (P00C11)	• Not applicable<br>○ MAiD medication protocol for self-administration is unavailable (P00C11)	• Not applicable<br>○ MAiD medication protocol for self-administration is unavailable (P00C11)	• Not applicable<br>○ MAiD medication protocol for self-administration is unavailable (P00C11)	• Community Pharmacy (P00C11)<br>○ All providers in Niagara use same community pharmacy (P00C11)	• Availability of backup kit not specified	• Responsible professional<br>○ Physicians/nurse practitioners (142) (P00C11)<br>• Cause of death<br>○ Underlying illness, disease or disability (160)<br>• Manner of death<br>○ Natural (142)	• Patient's family (P00C11)<br>• Patient (P00C11)

	Family dynamics		MAiD consent process		Medication protocol					Pharmacy involved		Post-provision arrangements	
					Intravenous		Oral						
**Jurisdiction**	**What are the strategies and guidelines MAiD team/assessors and providers utilize to address family dynamics**	**What supports are provided to patients who are alone?**	**Can final consent be waived for foreseeable natural death?**	**How are final consents provided?**	**What are the protocols for clinician-administered medication?**	**Which professionals are present for clinician administration?**	**What are the protocols for self-administrated medication?**	**What are the processes in place if a self-administrated medication fails?**	**Which professionals are present for self-administration?**	**Which pharmacies provide MAiD kit?**	**Does the pharmacy dispense a backup kit?**	**How is the death certificate completed?**	**Who is responsible for coordinating funeral arrangements?**
			informed of risk of losing capacity to consent to receive MAiD prior to specified date (24)<br>• MAiD is provided if patient:<br>○ Becomes incapable of consenting (24)<br>○ Shows no indication of declining MAiD (24)										
GEOGRAPHICAL AREAS													
Ontario - *Oakville & Mississauga area*	Strategies<br>• Providers<br>○ Facilitate access to support services (P00C18)	• No experience (P00C18)	• Yes (P00C18)<br>• If patient meets the following conditions before losing capacity to consent:<br>○ Patient meets all requirements and safeguards for MAiD (24)<br>○ Patient enters into written agreement with physicians/nurse practitioners to receive MAiD on or before a specified day (24)<br>○ Patient is informed of risk of losing capacity to consent to receive MAiD prior to specified date (24)<br>• MAiD is provided if patient:<br>○ Becomes incapable of consenting (24)	• Written (P00C18)<br>• Verbal (P00C18)<br>• Use of alternative options:<br>○ Ocular movement board (P00C18)	• Protocols for clinician-administered medication not specified	• Physicians/nurse practitioners (P00C18)	• Not applicable<br>○ MAiD medication protocol for self-administration is unavailable (P00C18)	• Not applicable<br>○ MAiD medication protocol for self-administration is unavailable (P00C18)	• Not applicable<br>○ MAiD medication protocol for self-administration is unavailable (P00C18)	• Community pharmacy (P00C18)<br>○ Unused medication will be returned to pharmacists by provider (P00C18)	• Yes (P00C18)	• Responsible professional<br>○ Physicians/nurse practitioners (142) (P00C18)<br>• Cause of death<br>○ Underlying illness, disease or disability (160)<br>• Manner of death<br>○ Natural (142)	• Patient's family (P00C18)<br>• Physician (P00C18)

	Family dynamics		MAiD consent process		Medication protocol					Pharmacy involved		Post-provision arrangements	
					Intravenous		Oral						
**Jurisdiction**	**What are the strategies and guidelines MAiD team/assessors and providers utilize to address family dynamics**	**What supports are provided to patients who are alone?**	**Can final consent be waived for foreseeable natural death?**	**How are final consents provided?**	**What are the protocols for clinician-administered medication?**	**Which professionals are present for clinician administration?**	**What are the protocols for self-administrated medication?**	**What are the processes in place if a self-administrated medication fails?**	**Which professionals are present for self-administration?**	**Which pharmacies provide MAiD kit?**	**Does the pharmacy dispense a backup kit?**	**How is the death certificate completed?**	**Who is responsible for coordinating funeral arrangements?**
			○ Shows no indication of declining MAiD (24)										
Ontario- *Oakville, Mississauga, & Burlington area*	Strategies<br>• Providers<br>○ Facilitate access to support services (P00C21)<br>○ Encourage patients to have conversations with families (P00C21)	• Providers<br>○ Identify existing supports available for patients (P00C21)<br>• Home care nurses<br>○ Provide support services to patients (P00C21)	• Yes (24)<br>• If patient meets the following conditions before losing capacity to consent:<br>○ Patient meets all requirements and safeguards for MAiD (24)<br>○ Patient enters into written agreement with physicians/nurse practitioners to receive MAiD on or before a specified day (24)<br>○ Patient is informed of risk of losing capacity to consent to receive MAiD prior to specified date (24)<br>• MAiD is provided if patient:<br>○ Becomes incapable of consenting (24)<br>○ Shows no indication of declining MAiD (24)	• Final consents not specified	• Protocols for clinician-administered medication not specified	• Physicians/nurse practitioners (P00C21)	• Yes (P00C21)<br>○ Secobarbital (15 g) (P0021)	• Processes in the event of self-administration failure not specified	• Physicians/nurse practitioners (P00C21)	• Community pharmacy (P00C21)<br>○ Unused medication returned to pharmacists by provider (P00C21)	• Yes	• Responsible professional<br>○ Physicians/nurse practitioners (142) (P00C21)<br>• Cause of death<br>○ Underlying illness, disease or disability (160)<br>• Manner of death<br>○ Natural (142)	• Responsibility for funeral arrangements not specified
Ontario - *Renfrew County*	Strategies<br>• Providers<br>○ Facilitate access to support services (P00C22)	• Providers (P00C22)<br>○ Identify existing supports available for patients (P00C22)	• Yes (P00C22)<br>• If patient meets the following conditions before losing capacity to consent:<br>○ Patient meets all requirements and safeguards for MAiD (24)	• Verbal (P00C22)<br>• Use of alternative options<br>○ Ocular movement board (P00C22)	• Protocols for clinician-administered medication not specified	• Physicians/nurse practitioners (P00C22)	• Not applicable<br>○ MAiD medication protocol for self-administration is unavailable (P00C22)	• Not applicable<br>○ MAiD medication protocol for self-administration is unavailable (P00C22)	• Not applicable<br>○ MAiD medication protocol for self-administration is unavailable (P00C22)	• Community pharmacy (P00C22)	• Availability of backup kit not specified	• Responsible professional<br>○ Physicians/nurse practitioners (142) (P00C22)<br>• Cause of death<br>○ Underlying illness, disease or disability (160)	• Patient's family (P00C22)<br>• Physician (P00C22)

Jurisdiction	Family dynamics: What are the strategies and guidelines MAiD team/assessors and providers utilize to address family dynamics	Family dynamics: What supports are provided to patients who are alone?	MAiD consent process: Can final consent be waived for foreseeable natural death?	MAiD consent process: How are final consents provided?	Medication protocol – Intravenous: What are the protocols for clinician-administered medication?	Medication protocol – Intravenous: Which professionals are present for clinician administration?	Medication protocol – Oral: What are the protocols for self-administrated medication?	Medication protocol – Oral: What are the processes in place if a self-administrated medication fails?	Medication protocol – Oral: Which professionals are present for self-administration?	Pharmacy involved: Which pharmacies provide MAiD kit?	Pharmacy involved: Does the pharmacy dispense a backup kit?	Post-provision arrangements: How is the death certificate completed?	Post-provision arrangements: Who is responsible for coordinating funeral arrangements?
		• Community/hospi tal social workers<br>○ Provide support services to patients (P00C22)	○ Patient enters into written agreement with physicians/nurse practitioners to receive MAiD on or before a specified day (24)<br>○ Patient is informed of risk of losing capacity to consent to receive MAiD prior to specified date (24)<br>• MAiD is provided if patient:<br>○ Becomes incapable of consenting (24)<br>○ Shows no indication of declining MAiD (24)									• Manner of death<br>○ Natural (142)	
Ontario - *Greater Toronto Area*	Strategies<br>• Providers<br>○ Facilitate access to support services (P00C24)	• Providers<br>○ Identify existing supports available for patients (P00C24)<br>• Hospital social workers<br>○ Provide support services for inpatients (P00C24)	• Yes (P00C24)<br>• If patient meets the following conditions before losing capacity to consent:<br>○ Patient meets all requirements and safeguards for MAiD (24)<br>○ Patient enters into written agreement with physicians/nurse practitioners to receive MAiD on or before a specified day (24)<br>○ Patient is informed of risk of losing capacity to consent to receive MAiD	• Verbal (P00C24)	• Protocols for clinician-administered medication not specified<br>○ Requisitions are developed by clinicians (P00C24)	• Physicians/nurse practitioners (P00C24)	• Not applicable<br>○ MAiD medication protocol for self-administration is unavailable (P00C24)	• Not applicable<br>○ MAiD medication protocol for self-administration is unavailable (P00C24)	• Not applicable<br>○ MAiD medication protocol for self-administration is unavailable (P00C24)	• Community pharmacy (P00C24)	• Availability of backup kit not specified	• Responsible professional<br>○ Physicians/nurse practitioners (142) (P00C24)<br>• Cause of death<br>○ Underlying illness, disease or disability (160)<br>• Manner of death<br>○ Natural (142)	• Patient's family (P00C24)<br>• Physician (P00C24)<br>• Appointed executor (P00C24)

Jurisdiction	Family dynamics: What are the strategies and guidelines MAiD team/assessors and providers utilize to address family dynamics	Family dynamics: What supports are provided to patients who are alone?	MAiD consent process: Can final consent be waived for foreseeable natural death?	MAiD consent process: How are final consents provided?	Medication protocol – Intravenous: What are the protocols for clinician-administered medication?	Medication protocol – Intravenous: Which professionals are present for clinician administration?	Medication protocol – Oral: What are the protocols for self-administrated medication?	Medication protocol – Oral: What are the processes in place if a self-administrated medication fails?	Medication protocol – Oral: Which professionals are present for self-administration?	Pharmacy involved: Which pharmacies provide MAiD kit?	Pharmacy involved: Does the pharmacy dispense a backup kit?	Post-provision arrangements: How is the death certificate completed?	Post-provision arrangements: Who is responsible for coordinating funeral arrangements?
			prior to specified date (24)<br>• MAiD is provided if patient:<br>○ Becomes incapable of consenting (24)<br>○ Shows no indication of declining MAiD (24)										
Ontario - *Waterloo area*	Strategies<br>• Providers<br>○ Facilitate access to support services (P00C25)<br>○ Encourage patients to have conversations with families (P00C25)	• Providers<br>○ Identify existing supports available for patients (P00C25)	• Yes (P00C25)<br>• If patient meets the following conditions before losing capacity to consent:<br>○ Patient meets all requirements and safeguards for MAiD (24)<br>○ Patient enters into written agreement with physicians/nurse practitioners to receive MAiD on or before a specified day (24)<br>○ Patient is informed of risk of losing capacity to consent to receive MAiD prior to specified date (24)<br>• MAiD is provided if patient:<br>○ Becomes incapable of consenting (24)<br>○ Shows no indication of declining MAiD (24)	• Verbal (P00C25)	• Medications used<br>○ Midazolam<br>○ Lidocaine<br>○ Propofol<br>○ Rocuronium (P00C25)<br>• If a central IV is used Lidocaine is not necessary (P00C25)	• Physicians/nurse practitioners (P00C25)<br>• Registered nurses<br>○ Establish intravenous line (P00C25)	• Not applicable<br>○ No experience with MAiD medication protocol for self-administration (P00C25)	• Not applicable<br>○ No experience with MAiD medication protocol for self-administration (P00C25)	• Not applicable<br>○ No experience with MAiD medication protocol for self-administration (P00C25)	• Hospital pharmacy (P00C25)<br>• Community pharmacy (P00C25)	• Yes<br>○ Must return kit if not used (P00C25)	• Responsible professional<br>○ Physicians/nurse practitioners (142) (P00C25)<br>• Cause of death<br>○ Underlying illness, disease or disability (160)<br>• Manner of death<br>○ Natural (142)	• Patient's family (P00C25)<br>• Physicians/nurse practitioners (P00C25)

	Family dynamics		MAiD consent process		Medication protocol					Pharmacy involved		Post-provision arrangements	
					Intravenous		Oral						
**Jurisdiction**	**What are the strategies and guidelines MAiD team/assessors and providers utilize to address family dynamics**	**What supports are provided to patients who are alone?**	**Can final consent be waived for foreseeable natural death?**	**How are final consents provided?**	**What are the protocols for clinician-administered medication?**	**Which professionals are present for clinician administration?**	**What are the protocols for self-administrated medication?**	**What are the processes in place if a self-administrated medication fails?**	**Which professionals are present for self-administration?**	**Which pharmacies provide MAiD kit?**	**Does the pharmacy dispense a backup kit?**	**How is the death certificate completed?**	**Who is responsible for coordinating funeral arrangements?**
Ontario - *Noelville, Sudbury, Elliot Lake, Sturgeon Falls area*	Strategies<br>• Providers<br>○ Encourage patients to have conversations with families (P00C25)	• Providers<br>○ Identify existing supports available for patients (P00C26)	• Yes (24)<br>• If patient meets the following conditions before losing capacity to consent:<br>○ Patient meets all requirements and safeguards for MAiD (24)<br>○ Patient enters into written agreement with physicians/nurse practitioners to receive MAiD on or before a specified day (24)<br>○ Patient is informed of risk of losing capacity to consent to receive MAiD prior to specified date (24)<br>• MAiD is provided if patient:<br>○ Becomes incapable of consenting (24)<br>○ Shows no indication of declining MAiD (24)	• Final consents not specified	• Protocols for clinician-administered medication not specified	• Physicians/nurse practitioners (P00C26)<br>• Support nurse<br>○ Establishes intravenous line (P00C26)	• Not applicable<br>○ MAiD medication protocol for self-administration is unavailable (P00C26)	• Not applicable<br>○ MAiD medication protocol for self-administration is unavailable (P00C26)	• Not applicable<br>○ MAiD medication protocol for self-administration is unavailable (P00C26)	• Community pharmacy (P00C26)	• Availability of backup kit not specified	• Responsible professional<br>○ Physicians/nurse practitioners (142) (P00C26)<br>• Cause of death<br>○ Underlying illness, disease or disability (160)<br>• Manner of death<br>○ Natural (142)	• Patient's family (P00C26)<br>• Friends (P00C26)
Québec - *Ministry of Health and Social Services*	• Not applicable	• Not applicable	• Yes (161)<br>• If patient meets the following conditions before losing capacity to consent:<br>○ Patient meets all requirements and safeguards for MAiD (161)<br>○ Patient enters into written agreement with physicians/nurse	• Not applicable	• Provincial standardized medication protocol<br>• Intravenous administration<br>○ Midazolam (1mg/ml) (162)<br>○ Lidocaine 2% (20 mg)<br>▪ Or Magnesium Sulfate (500mg/ml) (162)<br>○ Propofol (10mg/ml)<br>▪ Or Phenobarbital (120mg/ml over 5	• Physicians/nurse practitioners (163)<br>○ Must stay until death is confirmed (163)	• Not applicable<br>○ Self-administration is not allowed (163)	• Not applicable<br>○ Self-administration is not permitted by provincial law (163)	• Not applicable<br>○ Self-administration is not permitted by provincial law (163)	• Hospital pharmacy (P00C27)<br>• Community pharmacy (P00C27)	• Availability of backup kit not specified	• Responsible professional<br>○ Physicians/nurse practitioners (142)<br>• Cause of death<br>○ Underlying illness, disease or disability (142)<br>• Manner of death<br>○ Natural (142)	• Responsibility for funeral arrangements not specified

	Family dynamics		MAiD consent process		Medication protocol					Pharmacy involved		Post-provision arrangements	
					Intravenous		Oral						
Jurisdiction	What are the strategies and guidelines MAiD team/assessors and providers utilize to address family dynamics	What supports are provided to patients who are alone?	Can final consent be waived for foreseeable natural death?	How are final consents provided?	What are the protocols for clinician-administered medication?	Which professionals are present for clinician administration?	What are the protocols for self-administrated medication?	What are the processes in place if a self-administrated medication fails?	Which professionals are present for self-administration?	Which pharmacies provide MAiD kit?	Does the pharmacy dispense a backup kit?	How is the death certificate completed?	Who is responsible for coordinating funeral arrangements?
			practitioners to receive MAiD on or before a specified day (161)<br>○ Patient is informed of risk of losing capacity to consent to receive MAiD prior to specified date (161)		minutes) (162)<br>○ Rocuronium (10mg/ml)<br>▪ Or Cistaracurium (2mg/ml) (162)<br>○ NaCl 0.9% (10 mL) (162)<br>○ Bupivacaine (0.5%, 5mg/ml) (162)<br>○ Lorazepam (4mg/ml)<br>▪ Or Methotrimeprazine (25mg/ml) (162)<br>• Intraosseous administration<br>○ Midazolam (5mg/ml) (162)<br>○ Lidocaine (2%) (162)<br>○ NaCl 0.9% (162)								
INTEGRATED HEALTH AND SOCIAL SERVICES CENTRES													
Québec – *CISS Montérégie*	Strategies<br>• Providers<br>○ Encourage patients to have conversations with families	• Supports for alone patients not specified	• Yes (161)<br>• If patient meets the following conditions before losing capacity to consent:<br>○ Patient meets all requirements and safeguards for MAiD (161)<br>○ Patient enters into written agreement with physicians/nurse practitioners to receive MAiD on or before a specified day (161)<br>○ Patient is informed of risk of losing capacity to consent to receive MAiD prior to specified date (161)	• Verbal (P00C27)	• Provincial standardized medication protocol<br>• Intravenous administration<br>○ Midazolam (1mg/ml) (162)<br>○ Lidocaine 2% (20 mg)<br>▪ Or Magnesium Sulfate (500mg/ml) (162)<br>○ Propofol (10mg/ml)<br>▪ Or Phenobarbital (120mg/ml over 5 minutes) (162)<br>○ Rocuronium (10mg/ml)<br>▪ Or Cistaracurium (2mg/ml) (162)<br>○ NaCl 0.9% (10 mL) (162)<br>○ Bupivacaine (0.5%, 5mg/ml) (162)<br>○ Lorazepam (4mg/ml)<br>▪ Or Methotrimeprazine (25mg/ml) (162)	• Physicians/nurse practitioners (163) (P00C27)<br>○ Must stay until death is confirmed (163)<br>• Support nurses (P00C27)	• Not applicable<br>○ Self-administration is not allowed (163)	• Not applicable<br>○ Self-administration is not permitted by provincial law (163)	• Not applicable<br>○ Self-administration is not permitted by provincial law (163)	• Hospital pharmacy (P00C27)<br>• Community pharmacy (P00C27)	• Availability of backup kit not specified	• Responsible professional<br>○ Physicians/nurse practitioners (142)<br>• Cause of death<br>○ Underlying illness, disease or disability (142)<br>• Manner of death<br>○ Natural (142)	• Responsibility for funeral arrangements not specified

Jurisdiction	Family dynamics		MAiD consent process		Medication protocol					Pharmacy involved		Post-provision arrangements	
					Intravenous		Oral						
	**What are the strategies and guidelines MAiD team/assessors and providers utilize to address family dynamics**	**What supports are provided to patients who are alone?**	**Can final consent be waived for foreseeable natural death?**	**How are final consents provided?**	**What are the protocols for clinician-administered medication?**	**Which professionals are present for clinician administration?**	**What are the protocols for self-administrated medication?**	**What are the processes in place if a self-administrated medication fails?**	**Which professionals are present for self-administration?**	**Which pharmacies provide MAiD kit?**	**Does the pharmacy dispense a backup kit?**	**How is the death certificate completed?**	**Who is responsible for coordinating funeral arrangements?**
					• Intraosseous administration<br>○ Midazolam (5mg/ml) (162)<br>○ Lidocaine (2%) (162)<br>○ NaCl 0.9% (162)								
INTEGRATED UNIVERSITY HEALTH AND SOCIAL SERVICES CENTRES													
Québec – *CIUSS Capitale-Nationale*	• No experience (P00C37)	• Supports for alone patients not specified	• Yes (161)<br>• If patient meets the following conditions before losing capacity to consent:<br>○ Patient meets all requirements and safeguards for MAiD (161)<br>○ Patient enters into written agreement with physicians/nurse practitioners to receive MAiD on or before a specified day (161)<br>○ Patient is informed of risk of losing capacity to consent to receive MAiD prior to specified date (161)	• Written (161)<br>• Verbal (161)	• Provincial standardized medication protocol<br>• Intravenous administration<br>○ Midazolam (1mg/ml) (162)<br>○ Lidocaine 2% (20 mg)<br>▪ Or Magnesium Sulfate (500mg/ml) (162)<br>○ Propofol (10mg/ml)<br>▪ Or Phenobarbital (120mg/ml over 5 minutes) (162)<br>○ Rocuronium (10mg/ml)<br>▪ Or Cistaracurium (2mg/ml) (162)<br>○ NaCl 0.9% (10 mL) (162)<br>○ Bupivacaine (0.5%, 5mg/ml) (162)<br>○ Lorazepam (4mg/ml)<br>▪ Or Methotrimeprazine (25mg/ml) (162)<br>• Intraosseous administration<br>○ Midazolam (5mg/ml) (162)<br>○ Lidocaine (2%) (162)<br>○ NaCl 0.9% (162)	• Physicians/nurse practitioners (163) (P00C37)<br>○ Must stay until death is confirmed (163)	• Not applicable<br>○ Self-administration is not allowed (163)	• Not applicable<br>○ Self-administration is not permitted by provincial law (163)	• Not applicable<br>○ Self-administration is not permitted by provincial law (163)	• Hospital pharmacy (162)<br>• Community pharmacy (162)	• Availability of backup kit not specified	• Responsible professional<br>○ Physicians/nurse practitioners (142)<br>• Cause of death<br>○ Underlying illness, disease or disability (142)<br>• Manner of death<br>○ Natural (142)	• Responsibility for funeral arrangements not specified
Québec – *University of Montreal Hospital Center*	• No experience (P00C40)	• Supports for alone patients not specified	• Yes (161)<br>• If patient meets the following conditions before losing capacity to consent:<br>○ Patient meets all requirements and	• Final consents not specified	• Provincial standardized medication protocol<br>• Intravenous administration<br>○ Midazolam (1mg/ml) (162)<br>○ Lidocaine 2% (20 mg)	• Physicians/nurse practitioners (74, 163)<br>○ Must stay until death is confirmed (163)	• Not applicable<br>○ Self-administration is not allowed (163)	• Not applicable<br>○ Self-administration is not permitted by provincial law (163)	• Not applicable<br>○ Self-administration is not permitted by provincial law (163)	• Hospital pharmacy (P00C40)	• Availability of backup kit not specified	• Responsible professional<br>○ Physicians/nurse practitioners (142)<br>• Cause of death<br>○ Underlying	• Responsibility for funeral arrangements not specified

Jurisdiction	Family dynamics: What are the strategies and guidelines MAiD team/assessors and providers utilize to address family dynamics	Family dynamics: What supports are provided to patients who are alone?	MAiD consent process: Can final consent be waived for foreseeable natural death?	MAiD consent process: How are final consents provided?	Medication protocol – Intravenous: What are the protocols for clinician-administered medication?	Medication protocol – Intravenous: Which professionals are present for clinician administration?	Medication protocol – Oral: What are the protocols for self-administrated medication?	Medication protocol – Oral: What are the processes in place if a self-administrated medication fails?	Medication protocol – Oral: Which professionals are present for self-administration?	Pharmacy involved: Which pharmacies provide MAiD kit?	Pharmacy involved: Does the pharmacy dispense a backup kit?	Post-provision arrangements: How is the death certificate completed?	Post-provision arrangements: Who is responsible for coordinating funeral arrangements?
			safeguards for MAiD (161)<br>○ Patient enters into written agreement with physicians/nurse practitioners to receive MAiD on or before a specified day (161)<br>○ Patient is informed of risk of losing capacity to consent to receive MAiD prior to specified date (161)		▪ Or Magnesium Sulfate (500mg/ml) (162)<br>○ Propofol (10mg/ml)<br>▪ Or Phenobarbital (120mg/ml over 5 minutes) (162)<br>○ Rocuronium (10mg/ml)<br>▪ Or Cistaracurium (2mg/ml) (162)<br>○ NaCl 0.9% (10 mL) (162)<br>○ Bupivacaine (0.5%, 5mg/ml) (162)<br>○ Lorazepam (4mg/ml)<br>▪ Or Methotrimeprazine (25mg/ml) (162)<br>• Intraosseous administration<br>○ Midazolam (5mg/ml) (162)<br>○ Lidocaine (2%) (162)<br>○ NaCl 0.9% (162)							illness, disease or disability (142)<br>• Manner of death<br>○ Natural (142)	
Québec – *McGill University Health Centre*	Strategies<br>• MAiD team<br>○ Coordinator<br>▪ Facilitates access to support services (P00C9)	• Supports for alone patients not specified	• Yes (161)<br>• If patient meets the following conditions before losing capacity to consent:<br>○ Patient meets all requirements and safeguards for MAiD (161)<br>○ Patient enters into written agreement with physicians/nurse practitioners to receive MAiD on or before a specified day (161)<br>○ Patient is informed of risk of losing capacity	• Verbal (P00C9)	• Provincial standardized medication protocol<br>• Intravenous administration<br>○ Midazolam (1mg/ml) (162)<br>○ Lidocaine 2% (20 mg)<br>▪ Or Magnesium Sulfate (500mg/ml) (162)<br>○ Propofol (10mg/ml)<br>▪ Or Phenobarbital (120mg/ml over 5 minutes) (162)<br>○ Rocuronium (10mg/ml)<br>▪ Or Cistaracurium (2mg/ml) (162)<br>○ NaCl 0.9% (10 mL) (162)	• Physicians/nurse practitioners (163) (P00C9)<br>○ Must stay until death is confirmed (163)	• Not applicable<br>○ Self-administration is not allowed (163)	• Not applicable<br>○ Self-administration is not permitted by provincial law (163)	• Not applicable<br>○ Self-administration is not permitted by provincial law (163)	• Hospital pharmacy (P00C9)	• Yes (P00C9)	• Responsible professional<br>○ Physicians/nurse practitioners (142)<br>• Cause of death<br>○ Underlying illness, disease or disability (142)<br>• Manner of death<br>○ Natural (142)	• Responsibility for funeral arrangements not specified

Jurisdiction	Family dynamics		MAiD consent process		Medication protocol					Pharmacy involved		Post-provision arrangements	
					Intravenous		Oral						
	What are the strategies and guidelines MAiD team/assessors and providers utilize to address family dynamics	What supports are provided to patients who are alone?	Can final consent be waived for foreseeable natural death?	How are final consents provided?	What are the protocols for clinician-administered medication?	Which professionals are present for clinician administration?	What are the protocols for self-administrated medication?	What are the processes in place if a self-administrated medication fails?	Which professionals are present for self-administration?	Which pharmacies provide MAiD kit?	Does the pharmacy dispense a backup kit?	How is the death certificate completed?	Who is responsible for coordinating funeral arrangements?
			to consent to receive MAiD prior to specified date (161)		○ Bupivacaine (0.5%, 5mg/ml) (162)<br>○ Lorazepam (4mg/ml)<br>▪ Or Methotrimeprazine (25mg/ml) (162)<br>• Intraosseous administration<br>○ Midazolam (5mg/ml) (162)<br>○ Lidocaine (2%) (162)<br>○ NaCl 0.9% (162)								

Table 16: Patients and families preferences and rural considerations

Jurisdiction	What are the policies, standards, or practices in place to accommodate patients and families wishes regarding provision?	Who is involved in accommodating patients and families wishes?	How are patients and families wishes accommodated?	What accommodations are made for rural/remote areas?
Alberta - *Alberta Health Services*	• Practices<br>○ MAiD care coordination team and providers discuss wishes with patients and families (P00A5, P00C6, P00B13)<br>• Policies<br>○ Alberta Health Services MAiD Policy<br>▪ Professionals to respect patients' autonomy and assist patients and their families (3)	• MAiD care coordination team (P00A5, P00C6, P00B13)<br>○ Organize provision logistics (P00B13)<br>○ Liaise with provider, nursing staff, and health facility about patient preferences (P00B13)<br>○ Document requests from patients and families (P00B13)<br>• Physicians/nurse practitioners (P00A5, P00C6, P00B13)<br>○ Ask patients and families about wishes and preferences prior to provision (P00C6)	• Provision locations<br>○ Home (P00C6)<br>○ Hospital (P00C6)<br>○ Hospice (164)<br>○ Long-term care facilities (P00B13)<br>▪ Designated space (P00C39)<br>○ Meaningful location to patient (P00A5)<br>• Provision date and time<br>○ Patients can choose date and time of provision (P00B13)<br>• Cultural and religious requests<br>○ Document patient religious and ritual practices (P00C6)<br>▪ Involvement of clergy and end of life rites during provision (165)<br>• Special requests<br>○ Considerations for patient requests are made on case by case basis (P00A5)	• Travel accomodations<br>○ Ministry of Health<br>▪ Compensates physicians' mileage for travel to rural communities (P00A5)<br>○ Provincial Health Authority<br>▪ Funds travel for nurse practitioners (P00B9)
British Columbia - Ministry of Health	• Practices<br>○ Providers discuss wishes with patients and families (P00C17)<br>• Standards<br>○ British Columbia College of Nurses and Midwives (133)<br>▪ Nurse Practitioners assess cultural and spiritual needs and wishes of patients and explore meeting those needs (133)<br>○ College of Physicians and Surgeons of British Columbia (122)<br>▪ Registrants must treat patients with dignity and respect (122)	• Physicians/nurse practitioners (79, 122, 123)<br>○ Ask patients and families about wishes and preferences prior to provision (79, 122, 123)	• Provision locations<br>○ Home (P00A4)<br>○ Funeral homes (P00A4)<br>○ Meaningful location to patient (P00A4)	• Travel accomodations<br>○ Medical Assistance in Dying Travel and Training Assistance Program (MAiDTTAP) (166)<br>▪ Provides funding to assist health authorities in MAiD provision (166)<br>▪ Compensates visiting physicians for travel time and related costs, including meals and lodging (166)<br>▪ Physicans are eligibile if they travel ≥ 30 km (166)<br>▪ Nurse Practioners do not qualify for MAiDTTAP funding, health authorities determine travel compensation (166)<br>• Medications can be shipped to rural areas for provision through bonded courier (P00A4)
British Columbia - *Fraser Health*	• Practices<br>○ MAiD care coordination team and providers discuss wishes with patients and families (P00A10, P00C17)<br>• Policies<br>○ Fraser Health MAiD Policy<br>▪ Fraser Health committed to having conversations about patient values, beliefs, and goals of care (31)<br>▪ Support for patient preferences embedded in Fraser Health MAiD Policy (P00A10)	• MAiD care coordination team (P00A10)<br>○ Coordinate patient transfers (P00C17)<br>○ Liaises with provider about patient preferences (P00A10)<br>• Physicians/nurse practitioners (P00A10, P00C17)<br>○ Ask patients and families about wishes and preferences prior to provision (P00A10)	• Provision locations<br>○ Home (6, 28)<br>○ Hospital (6, 28)<br>○ Hospice (6, 28)<br>○ Long-term care facilities (6, 28)<br>▪ Designated MAiD space (P00A10)<br>○ Meaningful location to patient (6, 28)<br>• Provision date and time<br>○ Patients chooses date and time of provision based on provider availability (P00A10)<br>• Cultural and religious requests<br>○ Document patient religious and ritual practices (P00C17)<br>▪ Smudging ceremonies (P00C17)	• Travel accomodations<br>○ Medical Assistance in Dying Travel and Training Assistance Program (166)<br>▪ Compensates visiting physicians for travel time and related costs, including meals and lodging (166)
British Columbia - *Interior Health*	• Practices<br>○ MAiD care coordination team and providers discuss wishes with patients and families (P00B14)	• MAiD care coordination team (P00B14) (33)<br>○ Organize provision logistics (P00B14, P00C41)<br>○ Assess cultural and spiritual needs and wishes of patient (P00B14)<br>• Physicians/nurse practitioners (P00B14) (33)<br>○ Ask patients and families about wishes and preferences prior to provision (P00B14)	• Provision locations<br>○ Home (34)<br>○ Hospital (34)<br>○ Hospice (P00B14)<br>▪ Hospices in Kelowna have MAiD rooms (P00C41)<br>○ Long-term care (34)<br>○ Meaningful location to patient (34)<br>• Provision date and time<br>○ Provider will arrange to meet patient at a specific time (P00C29)<br>• Cultural and religious requests	• Travel accommodations<br>○ Medical Assistance in Dying Travel and Training Assistance Program (166)<br>▪ Compensates visiting physicians for travel time and related costs, including meals and lodging (166)<br>○ Regional Health Authority<br>▪ Compensates nurse practitioners for travel time and related costs (P00B14)

Jurisdiction	What are the policies, standards, or practices in place to accommodate patients and families wishes regarding provision?	Who is involved in accommodating patients and families wishes?	How are patients and families wishes accommodated?	What accommodations are made for rural/remote areas?
			○ Accomodate patient religious and cultural requests on case by case basis (P00B14)<br>• Special requests<br>○ Considerations for patient requests are made on case by case basis (P00C41)	
British Columbia-*Island Health*	• Practices<br>○ MAiD care coordination team and providers discuss wishes with patients and families (P00A13)	• MAiD care coordination team (P00A13)<br>○ Coordinate patients transfer (P00A13)<br>• Physicians/nurse practitioners (P00A13)<br>○ Ask patients and families about wishes and preferences prior to provision (P00A13)	• Provision locations<br>○ Home (35, 36)<br>○ Hospital (35, 36)<br>○ Long-term care facilities (35, 36)<br>○ Funeral homes (P00A9)<br>○ Meaningful location to patient (P00A13)<br>• Provision date and time<br>○ Patients can discuss preferences for time of provision (P00A13)	• Travel accommodations<br>○ Medical Assistance in Dying Travel and Training Assistance Program (MAiD TTAP) (166)<br>▪ Compensates visiting physicians for travel time and related costs including meals and lodging (166)<br>▪ Mileage is compensated for travel ≥ 30 km (P00A13)<br>○ Regional Health Authority<br>▪ Compensates nurse practitioners for cost of travel and travel time (P00A13)<br>▪ Funds travel to communities not covered through the MAiD TTAP (P00A9)
British Columbia - *Northern Health*	• Practices<br>○ Providers discuss wishes with patients and families (P00A8)	• Physicians (P00A8)<br>○ Ask patients and families about wishes and preferences prior to provision (P00A8)	• Provision locations<br>○ Home (P00A8)<br>• Cultural and religious requests<br>○ Document patient religious and ritual practices (P00A8)<br>▪ Involvement of spiritual care practitioners (P00A8)<br>▪ Smudging ceremonies (P00A8)	• Travel accommodations<br>○ Northern Isolation and Travel Assistance Outreach Program (P00A8)<br>▪ Compensates visiting physicians for travel time and expenses (P00A8)<br>○ Nurse practitioners have not been involved in provision and travel (P00A8)
British Columbia - *Vancouver Coastal Health*	• Practices<br>○ MAiD care coordination team and providers discuss wishes with patients and families (P00A7)	• MAiD care coordination team (P00A7)<br>○ Coordinate patients transfer (P00B15)<br>○ Organize provision logistics (P00A7)<br>○ Assess cultural and spiritual needs and wishes of patients and families (P00A7)<br>• Physicians/nurse practitioners (P00A7)<br>○ Ask patients and families about wishes and preferences during provision (P00A7)	• Provision locations<br>○ Home (P00B15)<br>○ Hospital (P00B15)<br>○ Long-term care facilities (167)<br>○ Meanignful location to patient (P00B15)<br>○ Assisted living (167)<br>○ Hospice (167)<br>○ Healthcare facilities (167)<br>○ Outpatient clinics (P00A7)<br>• Cultural and religious requests<br>○ Document patient religious and ritual practices (P00A7)<br>▪ Outdoor areas are provided in outpatient locations for ceremony (P00A7)	• Travel accommodations<br>○ Medical Assistance in Dying Travel and Training Assistance Program (166)<br>▪ Compensates visiting physicians for travel time and expenses including meals and lodging (166)<br>▪ Funds travel for physicians to rural communities (P00B10, P00B15)<br>○ Regional Health Authority<br>▪ Funds travel for nurse practitioners to rural communities (P00B10)
British Columbia - *Provincial Health Services Authority*	• Policies, standards or practices regarding patients and families wishes not specified	• Who accommodates patients and families wishes not specified	• Methods to accommodate patients and families wishes not specified	• Rural accommodations not specified
Manitoba - *Shared health*	• Practices<br>○ MAiD team and providers discuss wishes with patients and families (P00B21)<br>• Policies<br>○ The College of Physicians and Surgeons of Manitoba: Medical Assistance in Dying Standard of Practice (128)<br>▪ Physicians must consider patient's unique circumstances, perspectives, experiences, and religious or moral values and beliefs (128)	• MAiD team (P00B21)<br>○ Coordinate patient transfers (P00C33, P00C34)<br>○ Organize provision logistics (P00B19, P00C34)<br>○ Ask patients and families about wishes and preferences (P00C34)<br>○ Convey wishes to physician (P00C34)<br>• Physicians/nurse practitioners (128)<br>○ Ask patients and families about wishes and preferences during assessment (P00B21)	• Provision locations<br>○ Home (P00C33)<br>○ Hospital (P0C33)<br>○ Hospice (P00C33)<br>○ Funeral homes (P00C33)<br>○ Hotel (P00C33)<br>○ Meaningful location to patient (P00C33)<br>• Provision date and time<br>○ Provision hours: Monday- Friday 8 a.m. - 4.pm (P00B19)<br>○ Providers accommodate specific dates for patients (P00C34)<br>• Cultural and religious requests<br>○ Document patient religious and ritual practices (P00B19)<br>▪ Involvement of community spritual care provider if requested (P00B21)<br>• Special requests	• Travel accommodations<br>○ Ministry of Health<br>▪ Funds travel for physicians to rural communities (P00C33)<br>▪ Providers fly out to rural communities if patient is more than 3 hours away (P00B19, P00C33)<br>▪ Nurse practitioners have not been involved with travel (P00C33)

Jurisdiction	What are the policies, standards, or practices in place to accommodate patients and families wishes regarding provision?	Who is involved in accommodating patients and families wishes?	How are patients and families wishes accommodated?	What accommodations are made for rural/remote areas?
			○ Considerations for patient requests are made on case by case basis (P00C33)	
New Brunswick - *Horizon Health Network*	• Practices<br>○ MAiD care coordination team and providers discuss wishes with patients and families (P00B11)	• MAiD care coordination team (P00B11)<br>○ Coordinate patient transfers (P00B11)<br>○ Organize provision logistics (P00B11)<br>• Physicians/nurse practitioners (P00B11)<br>○ Ask patients and families about wishes and preferences during assessment (P00B11)<br>○ Notify MAiD coordinator about patient wishes (P00B11)	• Provision locations<br>○ Hospital (P0B11)<br>○ Hospice (P00B11)<br>○ Meaningful location to patient (P00B11)	• Travel accomodations<br>○ Ministry of Health<br>▪ Funds travel for physicians to rural communities (P00B11)<br>○ Nurse practitioners have not been involved in provision and travel (P00B11)
New Brunswick - *Vitalité Health Network*	• Practices<br>○ Providers discuss wishes with patients and families (P00C48)	• Home and community care nurses<br>○ Ask patients and families about wishes and preferences during assessment at home or in community (P00C48)	• Provision locations<br>○ Hospital (P00C48)<br>○ Home (P00C48)<br>○ Long-term care facilities (P00C48)	• Travel accomodation<br>○ Ministry of Health<br>▪ Funds travel for physicians to rural communities (P00C48)
Newfoundland and Labrador - *Department of Health and Community Services*	• Practices<br>○ Providers discuss wishes with patients and families (10)<br>• Professional Standards<br>○ Healthcare professionals must respect autonomy and dignity of patients<br>▪ College of Physicians and Surgeons of Newfoundland (168)<br>▪ Newfoundland and Labrador Pharmacy Board (21)<br>▪ College of Registered Nurses of Newfoundland and Labrador (147)	• Physicians/nurse practitioners<br>○ Ask patients and families about wishes and preferences during provision (10)	• Provision locations<br>○ Home (10)<br>○ Hospital (10)<br>○ Long-term care facilities (10)<br>○ Meaningful location to patient (10)	• Not applicable
Newfoundland and Labrador - *Eastern Health*	• Practices<br>○ Providers discuss wishes with patients and families (P00C43)	• MAiD care coordination team (P00C43)<br>○ Coordinate patient transfers (P00C43)<br>○ Organize provision logistics (P00C43)<br>• Physicians/nurse practitioners (P00C43)<br>○ Ask patients and families about wishes and preferences<br>○ Convey information to nurse coordinator (P00C43)	• Provision locations<br>○ Home (P00C43)<br>○ Hospital (P00C43)	• Travel accommodations<br>○ Ministry of Health<br>▪ Funds travel for physicians (P00C43)<br>○ Regional Health Authority<br>▪ Funds travel for nurse practitioners (P00C43)
Newfoundland and Labrador -*Western Zone*	• Practices<br>○ Providers discuss wishes with patients and families (P00C44)	• Physicians/nurse practitioners<br>○ Ask patients and families about wishes and preferences prior to provision (P00C44)<br>○ Support arrangement of provision location (P00C44)	• Provision locations<br>○ Home (P00C44)<br>○ Hospital (P00C44)<br>▪ Acute care bed (P00C44)<br>▪ Palliative care bed available for those who prefer procedure in hospital (P00C44)<br>○ Long term care facilities (P00C44)<br>○ Meaningful location to patient (P00C4)<br>• Provision date and time<br>○ Triage patients for urgency of MAiD provision (P00C44)	• Travel accommodations<br>○ Ministry of Health<br>▪ Funds travel for physicians (P00C43)<br>○ Regional Health Authority<br>▪ Funds travel for nurse practitioners (P00C43)
Newfoundland and Labrador - *Central Zone*	• Practices<br>○ MAiD care coordination team discuss wishes with patients and families (P00B22)	• MAiD care coordination team (P00B22)<br>○ Arrange provision locations (P00B22)	• Provision locations<br>○ Home (P00B22)<br>○ Hospital (P00B22)	• Travel accommodations<br>○ Ministry of Health<br>▪ Funds travel for physicians (P00B22, P00C43)<br>○ Regional Health Authority<br>▪ Funds travel for nurse practitioners (P00C43)
Northwest Territories	• Practices<br>○ MAiD care coordination team and providers discuss wishes with patients and families (P00A2, P00C35)<br>• Guidelines<br>○ Medical Assistance in Dying Guidelines for the Northwest Territories (11)	• MAiD care coordination team (P00A2)<br>○ Ask patients and families about wishes and preferences (P00A2)<br>• Physicians/nurse practitioners (P00C35)<br>○ Ask patients and families about wishes and preferences (P00C35)	○ Provision locations<br>○ Home (P00A2)<br>○ Hospital (P00A2)<br>○ Provide extra rooms or spaces to family (P00C35)<br>○ Long-term care facilities (P00C35)<br>○ Meaningful location to patient (P00A3)	• Travel accommodations<br>○ Ministry of Health<br>▪ Funds travel for physicians/nurse practitioners to remote communities (P00A2, P00A3)<br>- Pharmacy supplies travel medication box (P00A3)

Jurisdiction	What are the policies, standards, or practices in place to accommodate patients and families wishes regarding provision?	Who is involved in accommodating patients and families wishes?	How are patients and families wishes accommodated?	What accommodations are made for rural/remote areas?
	▪ Healthcare professionals must respect autonomy and dignity of patients (11)<br>▪ Healthcare professionals must respect patients cultural, linguistic, spiritual, and religious beliefs (11)		○ Provision time<br>○ Provision hours flexible (P00C35)<br>○ Assessments and provision can happen in same day (P00B24)<br>○ Cultural and religious requests<br>○ Accommodate patient religious and cultural requests on case-by-case basis (P00A3)	
Nova Scotia - *Nova Scotia Health*	• Practices<br>○ Providers discuss wishes with patients and families (P00A12, P00C38)<br>• Policies<br>○ Medical Assistance in Dying<br>▪ Healthcare professionals provide care that responds to needs, values, beliefs, and preferences of patients and their family members (169)<br>• Professional Standards<br>○ Provincial guidelines require healthcare professionals to respect autonomy and dignity of patients<br>▪ College of Physicians and Surgeons of Nova Scotia<br>▪ Nova Scotia College of Nursing<br>▪ Nova Scotia College of Pharmacists (13, 14, 156)	• MAiD care coordination team (P00C38)<br>○ Organize provision logistics (P00C38)<br>• Physicians/nurse practitioners (P00A12)<br>○ Ask patients and families about wishes and preferences prior to provision (P00C38)	• Provision locations<br>○ Home (P00C38)<br>○ Hospital (P00C38)<br>○ Hospice (P00C38)<br>○ Funeral home (P00A12)<br>○ Clinic (P00C38)<br>○ Meaningful location to patient (P00C38)<br>• Provision time<br>○ Provision hours are flexibile (P00C35)<br>▪ If patient is high priority, assessments and provision can happen in same day (P00A12)<br>• Cultural and religious requests<br>○ Document patient religious and ritual practices (P00A3)<br>▪ Smudging ceremonies (P00A12)	• Travel accommodations<br>○ Ministry of Health<br>▪ Funds travel for physicians (170) (P00A12)<br>○ Provincial Health Authority<br>▪ Funds travel for nurse practitioners (P00A12)
Prince Edward Island - *Health PEI*	• Practices<br>○ Providers discuss wishes with patients and families (P00B8)<br>• Policies<br>○ Health PEI MAiD Policy (P00B8)<br>▪ Patients must be respected (P00B8)<br>▪ MAiD provision can occur in any health PEI facility (P00B8)	• MAiD care coordination team (P00B17)<br>○ Organize provision logistics (P00B17)<br>• Physicians/nurse practitioners (P00B8)<br>○ Ask patients and families about wishes and preferences during assessment (P00B8)<br>○ Contact MAiD Coordinator with logistical information (P00B8)	• Provision locations<br>○ Home (P00B8)<br>○ Long-term care facilities (P00B8)<br>○ Cancer treatment center (P00B8)<br>○ Clinic (P00B8)<br>○ Palliative Care (P00B8)<br>○ Meaningful location to patient (P00B8)<br>• Cultural and Religious Requests<br>○ Accommodate patient religious and cultural requests on case-by-case basis (P00B8)<br>• Special requests<br>○ Considerations for patient requests are made on case-by-case basis (P00B8)	• Travel accommodations<br>○ Ministry of Health<br>▪ Funds travel for physicians (P00B8, P00B17)<br>○ Provincial Health Authority<br>▪ Nurse practitioners are salaried (P00B8)
Saskatchewan - *Saskatchewan Health Authority*	• Practices<br>○ MAiD care coordination team and providers discuss wishes with patients and families (P00B1)<br>• Professional Standards<br>○ Healthcare professionals required to treat patients and family with dignity and respect (171)<br>○ Consider patient perspectives including religious beliefs, and moral values (171)<br>○ College of Physicians and Surgeons of Saskatchewan (171)	• MAiD care coordination team (P00B1)<br>○ Coordinate patient transfers (58)<br>○ Ask patients and families about wishes and preferences (P00B7)<br>○ Work with social worker to arrange requests (P00B7)<br>• Physicians/nurse practitioners (P00A1)<br>○ Ask patients and families about wishes and preferences during assessment (P00A1)<br>○ Convey information to MAiD coordination team (P00A1)	• Provision locations<br>○ Home (P00B1)<br>○ Hospital (P00B1)<br>○ Hospice (P00B1)<br>○ Funeral home (P00B3)<br>○ Hotel (P00B1)<br>○ Meaningful location to patient (P00B7, P00C1)<br>• Provision time<br>○ Patients choose time for provision and notify MAiD coordination team 2-3 days in advance (P00C19)<br>• Cultural and religious requests<br>○ Accomodate patient religious and cultural requests on case by basis (P00A1)	• Travel accommodations<br>○ Ministry of Health<br>▪ Funds travel for physicians (172) (P00B3)<br>○ Regional Health Authority<br>▪ Nurse practitioners are salaried (P00C19)<br>• Medications dispensed remotely to providers at pharmacies without an on-site pharmacist present (P00B5)
Yukon - *Department of Health and Social Services*	• Practices<br>○ Providers discuss wishes with patients and families (P00B1)<br>• Standards of Practice MAiD<br>○ Yukon Medical council	• Physicians/nurse practitioners (P00C14)<br>○ Ask patients and families about wishes and preferences during assessment (P00C14)	• Provision locations<br>○ Home (P00C14)<br>○ Hospice (P00C14)<br>○ Palliative Care (P00C7)<br>○ Meaningful location to patient (P00C7, P00C14)<br>• Provision time	• Travel accommodations<br>○ Ministry of Health<br>▪ Funds travels for physicians and nurse practitioners (P00C7, P00C14)

Jurisdiction	What are the policies, standards, or practices in place to accommodate patients and families wishes regarding provision?	Who is involved in accommodating patients and families wishes?	How are patients and families wishes accommodated?	What accommodations are made for rural/remote areas?
	▪ Guidelines require healthcare professionals to respect autonomy and dignity of patients (119, 173)		○ MAiD is not provided on weekends (P00C14)<br>• Cultural and religious requests<br>○ Document patient religious and ritual practices (P00C7)<br>▪ Involvement of ministers and choirs (P00C7)	
Ontario - *Ministry of Health*	• Practices<br>○ Providers discuss wishes with patients and families (P00B4)	• Physicians/nurse practitioners (P00B4, P00C16)<br>○ Ask patients and families about wishes and preferences prior to provision (P00B4)	• Provision locations<br>○ Home (59)<br>○ Hospital (59)<br>○ Hospice (59)<br>○ Long-term care facilities (59)<br>○ Outdoor areas (P00C16)<br>• Cultural and religious requests<br>○ Accomodate patient religious and cultural requests on case by case basis (P00B4)	• Travel accommodations<br>○ Ministry of Health<br>▪ Funds travel time for physicians/nurse practitioners (P00C16)<br>▪ No reimbursements for flights, trains, or ferries (P00C16)
SERVICE ORGANIZATIONS/REGIONAL FACILITIES				
Ontario - *Home and Community Care Support Services, Central East*	• Practices<br>○ Providers discuss wishes with patients and families (P00C2)	• Physicians/nurse practitioners (P00C2)<br>○ Ask patients and families about wishes and preferences prior to provision (P00C2)	• Provision locations<br>○ Hospital (P00C2)<br>○ Hospice (P00C2)<br>○ Funeral home (P00C2)<br>• Cultural and religious requests<br>○ Accomodate patient religious and cultural requests on case by case basis (P00BC2)	• Travel accommodations<br>○ Ministry of Health<br>▪ Funds travel time for physicians/nurse practitioners (P00C2)
Ontario - *Home and Community Care Support Services, Waterloo Region*	• Practices<br>○ Providers discuss wishes with patients and families (P00C4)	• Physicians/nurse practitioners (P00C4)<br>○ Ask patients and families about wishes and preferences prior to provision (P00C4)	• Provision locations<br>○ Home (P00C4)<br>○ Hospital (P00C4)<br>○ Meaningful location to patient (P00C4)<br>• Provision time<br>○ Provision hours flexibile (P00C4)	• Travel accommodations<br>○ Funds travel time for physicians/nurse practitioners in rural or remote areas (P00C4)
Ontario - *Home and Community Care Support Services, South East*	• Practices<br>○ Providers discuss wishes with patients and families (P00C20)	• Physicians/nurse practitioners (P00C20)<br>○ Ask patients and families about wishes and preferences prior to provision (P00C20)	• Provision locations<br>○ Meaningful location to patient (P00C20)<br>• Cultural and religious requests<br>○ Accomodate patient religious and cultural requests on case by case basis (P00C20)	• Travel accommodations<br>○ Ministry of Health<br>▪ Funds travel time for physicians/nurse practitioners (P00C20)
Ontario - *Home and Community Care Support Services South West*	• Practices<br>○ Providers discuss wishes with patients and families (P00C23)	• Physicians/nurse practitioners (P00C23)<br>○ Organize provision logistics (P00C23)<br>○ Ask patients and families about wishes and preferences during assessment (P00C23)	• Provision locations<br>○ Home (P00C23)<br>○ Hospice (P00C23)<br>• Cultural and religious requests<br>○ Accomodate patient religious and cultural requests on case by case basis (P00C23)	• Travel accomodations<br>○ Ministry of Health<br>▪ Funds travel time for physicians (P00C23)<br>○ Nurse practitioners are salaried or have alternate funding arrangement (P00C23)
Ontario - *Champlain Regional MAiD Network, The Ottawa Hospital*	• Practices<br>○ MAiD care coordination team and providers discuss wishes with patients and families (P00C5)<br>○ Social workers ask patients about wishes and preferences (P00C5)	• MAiD care coordination team (P00C5)<br>○ Organize provision logistics (P00C5)<br>○ Ask patients and families about wishes and preferences prior to provision (P00C5)<br>• Physicians/nurse practitioners (P00C5)<br>○ Ask patients and families about wishes and preferences during provision (P00C5)	• Provision locations<br>○ Home (P00C5)<br>○ Hospital (P00C5)<br>• Cultural and religious requests<br>○ Document patient religious and ritual practices (P00C5)<br>▪ Involvement of spiritual care counsellors (P00C5)<br>▪ Smudging (P00C5)<br>▪ Music (P00C5)<br>▪ Artwork (P00C5)	• Travel accommodations<br>○ Regional Healthcare Facility<br>▪ Collaboration with physicians in remote communities for MAiD assessment and provision (P00C5)<br>▪ Nurse practitioners have not been involved in provision and travel (P00C5)
HEALTHCARE FACILITIES				
Ontario - *Peterborough Regional Health Centre*	• Practices<br>○ Providers discuss wishes with patients and families (P00C3)	• Physicians/nurse practitioners (P00C3)<br>○ Ask patients and families about wishes and preferences prior provision (P00C3)<br>○ Patients and families responsible for arrangement of requests	• Provision locations<br>○ Hospital (P00C3)<br>• Cultural and religious requests<br>○ Document patient religious and ritual practices (P00C3)	• Not applicable<br>○ MAiD is provided in the healthcare facility

Jurisdiction	What are the policies, standards, or practices in place to accommodate patients and families wishes regarding provision?	Who is involved in accommodating patients and families wishes?	How are patients and families wishes accommodated?	What accommodations are made for rural/remote areas?
			▪ Smudging ceremonies (P00C3)<br>▪ Music (P00C3)<br>▪ Artwork (P00C3)	
Ontario - *Mount Sinai Healthcare Facility, Toronto*	• Practices<br>○ Providers discuss wishes with patients and families (P00C36)	• MAiD care coordination team<br>○ Ask patients and families about wishes and preferences prior to provision (P00C36)	• Provision locations<br>○ Hospital (P00C36)<br>▪ Within palliative care unit (P00C36)	• No experience (P00C36)
Ontario - *University Health Network*	• Practices<br>○ MAiD coordination team and providers discuss wishes with patients and families (P00C8)	• MAiD care coordination team (P00C8)<br>○ Organize provision logistics (P00C8)<br>• Social worker and spiritual care team (P00C8)<br>○ Assess needs and wishes of patients and families<br>• Physicians/nurse practitioners (P00C8)<br>○ Ask patients and families about wishes and preferences prior to provision (P00C8)	• Provision locations<br>○ Hospital (P00C8)<br>• Cultural and religious requests<br>○ Document patient religious and ritual practices (P00C8)<br>▪ Involvement of spiritual care counsellors (P00C8)<br>▪ Prayers (P00C8)<br>▪ Smudging (P00C8)<br>▪ Music (P00C8)	• Not applicable<br>○ MAiD is provided in the healthcare facility
Ontario - *Grand River Hospital*	• Practices<br>○ Providers discuss wishes with patients and families (P00C50)	• MAiD care coordination team (P00C50)<br>○ Organize provision logistics (P00C50)<br>○ Ask patients and families about wishes and preferences (P00C50)<br>• Social worker and spiritual care workers (P00C50)<br>○ Assess needs and wishes of patients and families<br>• Physicians/nurse practitioners<br>○ Ask patients and families about wishes and preferences prior to provision (P00C50)	• Provision locations<br>○ Hospital (P00C50)<br>• Cultural and religious requests<br>○ Accommodate patient religious and cultural requests on case-by-case basis (P00C50)	• Not applicable<br>○ MAiD is provided in the healthcare facility
COMMUNITY OF PRACTICE				
Ontario - *Hamilton Family Health Team*	• Practices<br>○ Providers discuss wishes with patients and families (P00B18)	• Physicians/nurse practitioners (P00B18)<br>○ Ask patients and families about wishes and preferences prior to provision (P00B18)	• Provision locations<br>○ Home (P00B18)<br>○ Hospital (P00B18)<br>○ Hospice (P00B18)<br>○ Retirement center (P00B18)<br>○ Funeral home (P00B18)<br>• Provision time<br>○ Patients and providers discuss a date and time for provision (P00B18)<br>• Cultural and religious requests<br>○ Accomodate patient religious and cultural requests on case by case basis (P00B18)	• Travel accommodations<br>○ Ministry of Health<br>▪ Funds travel for physicians (P00B18)
Ontario - *Niagara Community MAiD Team, St. Catherine's, Niagara*	• Practices<br>○ Providers discuss wishes with patients and families (P00C11)	• Physicians/nurse practitioners (P00C11)<br>○ Ask patients and families about wishes and preferences prior to provision (P00C11)	• Provision locations<br>○ Home (P00C11)	• Travel accommodations<br>○ Ministry of Health<br>▪ Funds travel time for physicians/nurse practitioners (P00C11)<br>▪ Most travel occurs within 1.15 hours within urban area (P00C11)
GEOGRAPHICAL AREAS				
Ontario - *Oakville & Mississauga area*	• Practices<br>○ Providers discuss wishes with patients and families (P00C18)	• Physicians/nurse practitioners (P00C18)<br>○ Ask patients and families about wishes and preferences prior to provision (P00C18)	• Provision locations<br>○ Home (P00C18)<br>○ Hospital (P00C18)	• Travel accommodations<br>○ Ministry of Health<br>▪ Funds travel time for physicians (P00C18)<br>▪ Physicians travel to rural or remote patients (P00C18)
Ontario - *Oakville, Mississauga & Burlington area*	• Practices<br>○ Providers discuss wishes with patients and families (P00C21)	• Physicians/nurse practitioners (P00C21)<br>○ Ask patients and families about wishes and preferences prior to provision (P00C21)	• Provision locations<br>○ Home (P00C21)<br>○ Long-term care facilities (P00C21)<br>○ Retirement home<br>• Provision time<br>○ Provision hours flexibile (P00C21)	• No experience (P00C21)

Jurisdiction	What are the policies, standards, or practices in place to accommodate patients and families wishes regarding provision?	Who is involved in accommodating patients and families wishes?	How are patients and families wishes accommodated?	What accommodations are made for rural/remote areas?
			○ MAiD can be provided on weekends based on provider availability (P00C21)	
Ontario - *Renfrew County*	• Practices<br>○ Providers discuss wishes with patients and families (P00C22)	• Physicians/nurse practitioners (P00C22)<br>○ Ask patients and families about wishes and preferences prior to provision (P00C22)<br>○ Arrange location logistics (P00C22)	• Provision locations<br>○ Home (P00C22)<br>○ Hospital (P00C22)<br>○ Hospice (P00C22)<br>• Cultural and religious requests<br>○ Accomodate patient religious and cultural requests on case by case basis (P00C22)	• Travel accommodations<br>○ Ministry of Health<br>▪ Funds travel time for physicians (P00C22)<br>▪ Physicians travel to rural or remote patients (P00C22)
Ontario - *Greater Toronto Area*	• Practices<br>○ Providers discuss wishes with patients and families (P00C24)	• Physicians/nurse practitioners (P00C24)<br>○ Ask patients and families about wishes and preferences during assessment (P00C24)<br>○ Involved in helping arrange certain locations or spaces (P00C24)	• Provision locations<br>○ Home (P00C24)<br>○ Hospital (P00C24)<br>○ MAiDHouse (P00C24)<br>• Cultural and religious requests<br>○ Accomodate patient religious and cultural requests on case by case basis (P00C24)	• Travel accommodations<br>○ Ministry of Health<br>▪ Funds travel time for physicians (P00C24)<br>▪ Compensation for travel time is capped (P00C24)
Ontario - *Waterloo area*	• Practices<br>○ Providers discuss wishes with patients and families (P00C25)	• Physicians/nurse practitioners (P00C25)<br>○ Ask and accommodate patients and families about wishes and preferences during assessment (P00C25)	• Provision locations<br>○ Home (P00C25)<br>○ Hospital (P00C25)<br>○ Private outdoor area (P00C25)<br>• Provision time<br>○ Nurse availability can be limited on weekends (P00C25)<br>• Cultural and religious requests<br>○ Document patient religious and ritual practices (P00C25)<br>▪ Involvement of ministers and choirs (P00C25)	• Travel accommodations<br>○ Ministry of Health<br>▪ Funds travel time for physicians (P00C25)
Ontario - *Noelville, Sudbury, Elliot Lake, Sturgeon Falls area*	• Practices<br>○ Providers discuss wishes with patients and families (P00C26)	• Physicians/nurse practitioners (P00C26)<br>○ Ask and accommodate patients and families about wishes and preferences during assessment (P00C26)<br>○ Arrange and advocate for provision location	• Provision locations<br>○ Home (P00C26)<br>▪ Other locations are considered if patient does not have a home (P00C26)<br>• Provision time<br>○ Patients will choose a date for provision (P00C26)	• Travel accommodations<br>○ Ministry of Health<br>▪ Funds travel time for physicians (P00C26)<br>▪ Nurse practitioner from Home and Community Care Service travel to those living in distant areas once a month (P00C26)
Québec - *Ministry of Health and Social Services*	• Practices<br>○ Provider discuss wishes with patients and families (174)<br>• Provincial legislation<br>○ Healthcare professionals required to treat patients and family with dignity and respect (163)<br>○ Consider patient perspectives including religious beliefs, and moral values (163)	• Physicians/nurse practitioners (175)<br>○ Ask and accommodate patients and families about wishes and preferences during assessment (175)	• Provision locations<br>○ Home (163)<br>○ Hospital (163)<br>○ Palliative care homes (163)<br>○ Other locations with special authorization (174)	• Travel accommodations<br>○ Ministry of Health and Social Services<br>▪ Funds travel time for physicians (P00C27, P00C37)
INTEGRATED HEALTH AND SOCIAL SERVICES CENTRES				
Québec – *CISS Montérégie*	• Practices<br>○ Providers discuss wishes with patients and families (P00C27)	• Physicians/nurse practitioners (P00C27)<br>○ Ask and accommodate patients and families about wishes and preferences during assessment (P00C27)	• Provision locations<br>○ Home (P00C27)<br>○ Hospital (P00C27)<br>• Provision time<br>○ Patients and providers will choose a date for provision (P00C27)	• Travel accommodations<br>○ Ministry of Health and Social Services<br>▪ Funds travel time for physicians (P00C27)
INTEGRATED UNIVERSITY HEALTH AND SOCIAL SERVICES CENTRES				
Québec – *CIUSS Capitale-Nationale*	• Practices<br>○ Providers discuss wishes with patients and families (P00C37)	• Physicians/nurse practitioners (P00C37)<br>○ Ask and accommodate patients and families about wishes and preferences during assessment (P00C37)	• Provision locations<br>○ Home (P00C37)<br>○ Hospital (P00C37)<br>• Provision time<br>○ Patients and providers will choose a date for provision (P00C37)	• Travel accommodations<br>○ Ministry of Health and Social Services<br>▪ Funds travel time for physicians (P00C37)

Jurisdiction	What are the policies, standards, or practices in place to accommodate patients and families wishes regarding provision?	Who is involved in accommodating patients and families wishes?	How are patients and families wishes accommodated?	What accommodations are made for rural/remote areas?
Québec – *University of Montreal Hospital Center*	• Practices<br>○ Providers discuss wishes with patients and families (P00C40)	• Physicians/nurse practitioners (P00C40)<br>○ Ask and accommodate patients and families about wishes and preferences during assessment (P00C40)	• Provision locations<br>○ Hospital (P00C40)<br>• Provision time<br>○ Patients and providers will choose a date for provision (P00C40)	• Not applicable<br>○ MAiD is provided in the healthcare facility
Québec – *McGill University Health Centre*	• Practices<br>○ Providers discuss wishes with patients and families (P00C9)	• Physicians/nurse practitioners (P00C9)<br>○ Ask patients and families about wishes and preferences during assessment and accommodates (P00C9)	• Provision locations<br>○ Hospital (P00C9)<br>• Provision time<br>○ Patient will choose a date for provision (P00C9)<br>• Cultural and religious requests<br>○ Document patient religious and ritual practices (P00C9)	• Not applicable<br>○ MAiD is provided in the healthcare facility

Table 17: Delivery of bereavement services

Jurisdiction	Does the program have dedicated grief and bereavement services?	Does the MAiD team/assessors and providers support families and friends after MAiD provision?	What grief and bereavement services/support groups are families and friends referred to?	What grief and bereavement resources are families and friends referred to/provided with?
Alberta - *Alberta Health Services*	• No<br>○ Grief and bereavement services provided within health authority (P00A5)	• Yes<br>○ Families and friends are provided with immediate emotional support after provision (P00B13)<br>○ Families and friends are referred to community/hospital social workers (P00B9, P00B13, P00C39)<br>○ Families and friends have access to contact information of provider (P00C12)	• Families and friends are referred to support groups<br>○ Dying with Dignity (P00B9, P00B13, P00C10, P00C39)<br>○ Bridge C-14 (P00B9, P00B13, P00C10, P00C39)<br>○ MAID Family Support Society (P00B9, P00B13, P00C10, P00C39)<br>○ Alberta Health Services Grief Support Program (P00B9, P00B13, P00C10, P00C39)	• Bereavement brochures are provided to families and friends (P00B9)<br>• Families and friends are provided with grief and bereavement resources (P00B9, P00C12)
British Columbia - *Ministry of Health*	• Not applicable	• Not applicable	• Not applicable	• Not applicable
British Columbia - *Fraser Health*	• No (P00A10)	• Yes<br>○ Families and friends are referred to community social workers (P00A10)	• Families and friends are referred to support groups<br>○ Bridge C-14 (6)<br>○ MAID Family Support Society (6)<br>○ Virtual hospice (6)	• A bereavement guide available for families and friends (6)
British Columbia - *Interior Health*	• No<br>○ Grief and bereavement services provided within health authority (P00B14)	• Yes<br>○ Families and friends have access to contact information of provider (P00B14, P00C29, P00C41)<br>○ Families and friends are referred to community/hospital social workers (P00C29)	• Families and friends are referred to support groups and community organizations<br>○ MAID Family Support Society (P00B14, P00C29)<br>○ Bridge for You (P00B14, P00C29)	• Bereavement toolkit available to families and friends (P00C41)<br>• Families and friends are referred to available bereavement resources (P00C41)
British Columbia - *Island Health*	• Yes<br>○ MAiD program support group (P00A13)	• Yes<br>○ Families and friends are referred to community/hospital social workers (P00A9)	• Families and friends are referred to support groups<br>○ Dying with Dignity (P00A9)<br>○ Bridge C-14 (P00C13)<br>○ MAID Family Support Society (P00C13)<br>○ Hospice societies (P00A13)<br>○ Victoria hospice (P00A13, P00C13)<br>• Individual are referred to the MAiD program support group (P00A9)	• MAiD support and bereavement guide is available families and friends (107) (P00A9)<br>• Families and friends are referred to available bereavement resources (P00C13)<br>• Families and friends are referred to online resources (107)<br>• Individual are referred to the MAiD program support group (P00A9)
British Columbia - *Northern Health*	• No (P00A8)	• Yes<br>○ Families and friends have access to contact information of provider (P00A8)<br>○ Families and friends are provided with information on available resources (P00A8)	• Families and friends are referred to support groups<br>○ Dying with Dignity (P00A8)<br>○ Bridge C-14 (P00A8)<br>○ Victoria hospice (P00A8)	• Bereavement brochure available for families and friends (P00A8)
British Columbia - *Vancouver Coastal Health*	• No (P00A7, P00B10)	• Yes<br>○ Families and friends are referred to community/hospital social workers (P00A7, P00B10)<br>○ MAiD team connects with family members (P00A7, P00B10)<br>○ Families and friends referred to a spiritual care practitioner (P00B10, P00A7)	• Families and friends are referred to support groups<br>○ Dying with Dignity (P00B15, P00B10, P00B12, P00B16)<br>○ Bridge C-14 (P00B15, P00B10, P00B12, P00B16)	• Grief and bereavement guide available for families and friends (40)<br>• Families and friends are referred to MAiD bereavement support within hospice societies (P00B10)
British Columbia - *Provincial Health Services Authority*	• No (P00A11)	• No<br>○ Families and friends are referred to respective regional health authority supports (P00A11)	• Families and friends are referred to respective regional health authority supports (P00A11)	• Families and friends are referred to respective regional health authority supports (P00A11)
Manitoba - *Shared Health*	• Yes (P00B19)<br>○ Grief and bereavement support provided by the MAiD team (P00B19)	• Yes<br>○ Social workers (with MAiD team) provide up to six bereavement sessions for families and friends (P00B21, P00C33)<br>▪ Free virtual live sessions (P00B21, P00C33)	• Families and friends are referred to MAiD program grief and bereavement support sessions (P00B21, P00C33)<br>• Access to cancer resources for cancer patients (P00B21)	• Families and friends are referred to available resources (P00B21, P00C33)
New Brunswick - *Horizon Health Network*	• No (P00B11, P00C15)	• Yes<br>○ Coordinator (with MAiD team) connects with families and friends and provide information to available resources (P00C15)	• Families and friends are referred to support groups<br>○ MAID Family Support (P00B11)	• Families and friends are referred to available resources (P00B11)
New Brunswick - *Vitalité Health Network*	• No (P00C48)	• Yes<br>○ Families and friends are referred to home care nurses (P00C48)	• Families and friends are referred to hospital/community support services (P00C48)	• Families and friends are referred to community resources (P00C48)

Jurisdiction	Does the program have dedicated grief and bereavement services?	Does the MAiD team/assessors and providers support families and friends after MAiD provision?	What grief and bereavement services/support groups are families and friends referred to?	What grief and bereavement resources are families and friends referred to/provided with?
		○ Families and friends are referred to community social workers (P00C48)		
Newfoundland and Labrador - *Department of Health and Community Services*	• Not applicable<br>○ No involvement in service delivery	• Not applicable<br>○ No involvement in service delivery	• Not applicable<br>○ No involvement in service delivery	• Not applicable<br>○ No involvement in service delivery
Newfoundland and Labrador - *Eastern Zone*	• No<br>○ Grief and bereavement services provided within health zone (P00C43)	• Yes<br>○ Families and friends are provided with information on available resources (P00C43)	• Families and friends are referred to support groups<br>○ Grief services (P00C43)<br>○ Pastoral care (P00C43)<br>○ Pan-Canadian support groups (P00C43)	• Families and friends are referred to bereavement support services (P00C43)
Newfoundland and Labrador - *Western Zone*	• No<br>○ Clinical community support services provided within health zone (P00C44)	• Yes<br>○ Families and friends are referred to community social workers (P00C44)	• Families and friends are referred to support groups<br>○ Bridge C-14 (P00C44)<br>○ Pan-Canadian groups (P00C44)	• Families and friends are provided with relevant information (P00C44)
Newfoundland and Labrador - *Central Zone*	• No (P00B22)	• Yes<br>○ Families and friends are provided with information on available resources (P00B22)	• Referrals to support groups not specified	• Families and friends are referred to available resources (P00B22)<br>• Families and friends are provided with grief and bereavement resources (P00B22)
Northwest Territories	• No (P00A2)	• Yes<br>○ Families and friends are provided with information on available resources (P00A3, P00C35)	• Families and friends are referred to support groups<br>○ Bridge C-14 (P00A3)<br>○ MAID Family Support Society (P00A3)<br>○ Canadian Virtual Hospice (P00A3)<br>○ Pilgrims Hospice (P00A2)	• Bereavement guides available for families and friends (P00A3)
Nova Scotia - *Nova Scotia Health*	• Yes<br>○ MAiD program grief and bereavement services (P00A12)	• Yes<br>○ Social workers (with MAiD team) provide bereavement sessions on request of families (P00A12)<br>▪ Families and friends have access to grief and bereavement programs (P00A12)	• Families and friends are referred to MAiD program grief and bereavement services (P00A12)	• Families and friends are referred to MAiD program grief and bereavement services (P00A12)
Prince Edward Island - *Health PEI*	• No (P00B17)	• Yes<br>○ Social worker (with MAiD team) provides grief resources (P00B8, P00B17)	• Families and friends are referred to support groups<br>○ Bridge C-14 (P00B17)<br>○ Canadian Virtual Hospice (P00B17)<br>○ Other local grief support programs (P00B8)	• Grief and bereavement sheet provided to patients and families (54) (P00B17)<br>• Families and friends are provided with relevant information (P00B17)
Saskatchewan - *Saskatchewan Health Authority*	• No (P00B1)	• Yes<br>○ Social workers (with MAiD team) provide grief resources (P00A1, P00C1)	• Families and friends are referred to support groups<br>○ Christie's bereavement/funeral home (P00C19)	• Grief and bereavement brochure provided to patients, families and friends (P00C19)<br>• Families and friends are provided with relevant information through MAiD program (P00C19)
Yukon - *Department of Health and Social Services*	• No (P00C14)	• Yes<br>○ Families and friends have access to contact information of provider (P00C7)	• Families and friends are referred to support groups<br>○ BC Cancer (P00C14)<br>○ Hospice Yukon society (P00C14)<br>○ Family support program (P00C14)	• Families and friends are referred to community resources (P00C14)
Ontario - *Ministry of Health*	• No (P00B4, P00C16)	• Not applicable<br>○ No involvement in service delivery	• Not applicable<br>○ No involvement in service delivery	• Not applicable<br>○ No involvement in service delivery
SERVICES ORGANIZATIONS/ REGIONAL FACILITIES				
Ontario - *Home and Community Care Support Services, Central East*	• No (P00C2)	• Yes<br>○ Families and friends have access to contact information of provider (P00C2)	• Families and friends are referred to support groups<br>○ Bridge C-14 (P00C2)<br>○ Hospice (P00C2)	• Families and friends are referred to available resources (P00C2)
Ontario - *Home and Community Care Support Services, Waterloo Region*	• No<br>○ Grief and bereavement support provided within region (P00C4)	• Yes<br>○ Families and friends are provided with information on available resources (P00C4)	• Families and friends are referred to support groups<br>○ MAID Family Support Society (P00C4)	• Yes<br>○ Families and friends are referred to available resources (P00C4)
Ontario - *Home and Community Care Support Services, South East*	• No (P00C20)	• Yes<br>○ Families and friends are referred to support groups (P00C20)	• Families and friends are referred to support groups<br>○ Bridge C-14 (P00C20)<br>○ Canadian Virtual Hospice (P00C20)<br>○ Local funeral homes (P00C20)	• Families and friends are referred to available resources (P00C20)
Ontario - *Home and Community Care Support Services, South West*	• No (P00C23)	• Yes<br>○ Families and friends are referred to support groups<br>▪ Bridge C-14 (P00C23)	• Yes<br>○ Families and friends are referred to support groups<br>○ Bridge C-14 (P00C23)	• Families and friends are referred to available resources (P00C23)

Jurisdiction	Does the program have dedicated grief and bereavement services?	Does the MAiD team/assessors and providers support families and friends after MAiD provision?	What grief and bereavement services/support groups are families and friends referred to?	What grief and bereavement resources are families and friends referred to/provided with?
Ontario - *Champlain Regional MAiD Network, the Ottawa Hospital*	• No (P00C5)	• Yes<br>○ Hospital social workers provide grief and bereavement support (P00C5)	• Families and friends are referred to support groups<br>○ Dying with Dignity<br>○ Bridge C-14 (P00C5)	• A grief and bereavement is available to families and friends (P00C5)<br>• Families and friends are referred to available resources (P00C5)
HEALTHCARE FACILITIES				
Ontario - *Peterborough Regional Health Centre*	• No (P00C3)	• Yes<br>○ Social worker (with MAiD team) provides grief resources (P00C3)<br>○ Families and friends are referred to available resources (P00C3)	• Families and friends are referred to support groups<br>○ Residential Hospice (P00C3)<br>○ Peterborough hospice (P00C3)	• Families and friends are referred to available resources (P00C3)
Ontario - *Mount Sinai Healthcare Facility, Toronto*	• No (P00C36)	• Yes<br>○ Families and friends are referred to support programs and available resources (P00C36)	• Families and friends are referred to support groups<br>○ Caregivers support program (P00C36)	• Families and friends are referred to available resources (P00C36)
Ontario - *University Health Network*	• No (P00C8)	• Yes<br>○ Hospital social workers and spiritual care practitioners provide bereavement sessions (P00C8)	• Families and friends are referred to community support services (P00C8)	• Families and friends are referred to available resources (P00C8)
Ontario - *Grand River Hospital*	• No (P00C50)	• Yes<br>○ Families and friends are provided with information on available resources (P00C50)	• Families and friends are referred to community support services (P00C50)	• Families and friends are referred to available resources (P00C50)
COMMUNITY OF PRACTICE				
Ontario - *Hamilton Family Health Team*	• Yes<br>○ Hamilton Health Team provide support services (P00B18)	• Yes<br>○ Mental health nurses provide grief resources and counselling (P00B18)<br>○ Families and friends are provided with information on available resources (P00B18)	• Families and friends are referred to support groups<br>○ Bridge C-14 (P00B18)	• Families and friends are referred to available resources (P00B18)
Ontario - *Niagara Community MAiD Team, St. Catharines, Niagara*	• No (P00C11)	• Yes (P00C11)<br>○ Families and friends are referred to support groups (P00C11)	• Families and friends are referred to support groups<br>○ Bridge C-14 (P00C11)	• Families and friends are referred to available resources (P00C11)
GEOGRAPHICAL AREAS				
Ontario - *Oakville & Mississauga area*	• No (P00C18)<br>○ Grief and bereavement support provided through palliative care service (P00C18)	• Yes<br>○ Families and friends are referred to support programs (P00C18)	• Families and friends are referred to support groups<br>○ Canadian Virtual Hospice (P00C18)	• Families and friends are referred to grief and counselling services (P00C18)
Ontario - *Oakville, Mississauga & Burlington area*	Not specified	• Yes<br>○ Families and friends are referred to psycho-social/spiritual counsellors (P00C21)	• Families and friends are referred to support groups<br>○ Bridge C-14 (P00C21)<br>○ Nursing facilities (P00C21)	• Families and friends are referred to available resources (P00C21)
Ontario - *Renfrew County*	• No (P00C22)	• Yes<br>○ Families and friends are referred to community social workers (P00C22)	• Families and friends are referred to support groups<br>○ Bridge C-14 (P00C22)	• Families and friends are referred to community resources (P00C22)
Ontario - *Greater Toronto Area*	• No (P00C24)	• Yes<br>○ Families and friends are provided with information on available resources (P00C24)	• Families and friends are referred to support groups<br>○ Dying with Dignity (P00C24)<br>○ Bridge C-14 (P00C24)<br>○ MAiDHouse (P00C24)	• Families and friends are referred to online resources (P00C24)
Ontario - *Waterloo Area*	• No (P00C25)	• Yes<br>○ Families and friends have access to contact information of provider (P00C25)	• Families and friends are referred to peer support groups (P00C25)	• Families and friends are referred to available resources (P00C25)
Ontario - *Noelville, Sudbury, Elliot Lake, Sturgeon Falls area*	• No (P00C26)	• Yes<br>○ Families and friends are provided with information on available resources (P00C26)	• Families and friends referred to support groups<br>○ Dying with Dignity (P00C26)<br>○ Bridge C-14 (P00C26)	• Families and friends referred to online resources (P00C26)
Québec - *Ministry of Health and Social Services*	• Not applicable<br>○ No involvement in service delivery	• Not applicable<br>○ No involvement in service delivery	• Not applicable<br>○ No involvement in service delivery	• Not applicable<br>○ No involvement in service delivery
INTEGRATED HEALTH AND SOCIAL SERVICES CENTRES				

Jurisdiction	Does the program have dedicated grief and bereavement services?	Does the MAiD team/assessors and providers support families and friends after MAiD provision?	What grief and bereavement services/support groups are families and friends referred to?	What grief and bereavement resources are families and friends referred to/provided with?
Québec – *CISS Montérégie*	• No (P00C27)	• Yes<br>○ Families and friends have access to contact information of provider (P00C27)<br>○ Families and friends are provided with information on available resources (P00C27)	• Families and friends are referred to support groups<br>○ Local CSLC (P00C27)	• Families and friends are referred to available resources (P00C27)
INTEGRATED UNIVERSITY HEALTH AND SOCIAL SERVICES CENTRES				
Québec – *CIUSS Capitale-Nationale*	• No (P0037)	• Yes<br>○ Families and friends are provided with information on available resources (P00C37)	• Families and friends are referred to community support services (P00C37)	• Families and friends are referred to available resources (P00C37)
Québec – *University of Montreal Hospital Center*	• No (P00C40)	• Yes<br>○ Families and friends are provided with information on available resources (P00C40)	• Families and friends are referred to community support services (P00C40)	• Families and friends are referred to available resources (P00C40)
Québec – *McGill University Health Centre*	• No (P00C9)	• Yes<br>○ Families and friends are provided with information on available resources (P00C9)	• Families and friends are referred to community support services (P00C40)	• Families and friends are referred to available resources (P00C9)

Table 18: Special considerations – incarcerated population

Jurisdiction	Does the program have experience providing MAiD related support to this population?	Does the MAiD program have any policies in place to support this population?	How does the MAiD program work with population specific support?	How are assessments completed? (virtual, in person, not described)	Can the program accommodate patients and families' preferences and practices?
Alberta - *Alberta Health Services*	• Yes (P00B9, P00A5)	• No (P00A5)	• Collaborative effort between correctional facilities and MAiD team to deliver MAiD (P00B9, P00A5)<br>○ Adherence to federal and provincial policies governing MAiD within correctional facilities (P00C39)	• In person (P00B9, P00A5)<br>• Virtual (P00B9, P00A5)	• Not applicable<br>○ No provision done
British Columbia - *Ministry of Health*	• Not applicable<br>○ No involvement in service delivery	• Not applicable<br>○ No involvement in service delivery	• Not applicable<br>○ No involvement in service delivery	• Not applicable<br>○ No involvement in service delivery	• Not applicable<br>○ No involvement in service delivery
British Columbia - *Fraser Health Authority*	• Yes (P00A10)	• No (P00A10)	• Collaborative effort between correctional facilities and regional health authority to deliver MAiD (P00A10)	• In person (P00A10)<br>• Virtual (P00A10)	• Yes<br>○ Compassionate release to receive MAiD (P00A14)<br>○ Release subject to patient's security level and history (P00A10)
British Columbia - *Interior Health*	• No (P00C41)	• Policies supporting incarcerated populations not specified	• Not applicable	• Not applicable	• Not applicable
British Columbia - *Island Health*	• No (P00A13)	• No (P00A13)	• Not applicable	• Not applicable	• Not applicable
British Columbia - *Northern Health*	• Experience with incarcerated populations not specified	• Policies supporting incarcerated populations not specified	• Collaboration with incarcerated population support groups not specified	• Modality of assessments for incarcerated populations not specified	• Accommodation of wishes for incarcerated populations not specified
British Columbia - *Vancouver Coastal Health*	• No (P00A7, P00B15)	• No (P00A7, P00B15)	• Not applicable<br>○ No experience	• Not applicable<br>○ No experience	• Not applicable<br>○ No experience
British Columbia - *Provincial Health Services Authority*	• Experience with incarcerated populations not specified	• Policies supporting incarcerated populations not specified	• Collaboration with incarcerated population support groups not specified	• Modality of assessments for incarcerated populations not specified	• Accommodation of wishes for incarcerated populations not specified
Manitoba - *Shared Health*	• Yes (P00C34)	• No (P00B19)	• Collaborative effort between correctional facilities and MAiD team to deliver MAiD (P00B19)	• In person (P00C33)<br>• Virtual (P00B21)	• No<br>○ Patient and family preferences may not be accommodated due to constraints (P00C33)
New Brunswick - *Horizon Health Network*	• No (P00C15)	• No (P00C15)	• Not applicable<br>○ No experience	• Not applicable<br>○ No experience	• Not applicable<br>○ No experience
New Brunswick - *Vitalité Health Network*	• No (P00C48)	• No (P00C48)	• Not applicable<br>○ No experience	• Not applicable<br>○ No experience	• Not applicable<br>○ No experience
Newfoundland and Labrador - *Department of Health and Community Services*	• Not applicable	• Not applicable	• Not applicable	• Not applicable	• Not applicable
Newfoundland and Labrador - *Eastern Zone*	• No (P00C43)	• No (P00C43)	• Not applicable<br>○ No experience	• Not applicable<br>○ No experience	• Not applicable<br>○ No experience
Newfoundland and Labrador - *Western Zone*	• No (P00C44)	• No (P00C44)	• Not applicable<br>○ No experience	• Not applicable<br>○ No experience	• Not applicable<br>○ No experience
Newfoundland and Labrador - *Central Zone*	• No (P00B22)	• No (P00B22)	• Not applicable<br>○ No experience	• Not applicable<br>○ No experience	• Not applicable<br>○ No experience
Northwest Territories	• No (P00A3)	• No (P00A3)	• Not applicable<br>○ No experience	• Not applicable<br>○ No experience	• Not applicable<br>○ No experience
Nova Scotia - *Nova Scotia Health*	• Yes (P00A12)	• No (P00A12)	• Collaborative effort between correctional facilities and MAiD team to deliver MAiD (P00A12)	• In person (P00A12)	• Yes<br>○ Compassionate release to receive MAiD (P00A12)
Prince Edward Island - *Health PEI*	• No (P00B17)	• No (P00B17)	• Not applicable<br>○ No experience	• Not applicable<br>○ No experience	• Not applicable<br>○ No experience
Saskatchewan - *Saskatchewan Health Authority*	• Yes (P00A1, P00B5, P00C1)	• No (P00C1)	• Collaborative effort between correctional facilities and MAiD team to deliver MAiD (P00C1)<br>○ Collaborative effort between correctional facilities and health authority to streamline assessment and provision (P00C1)	• In person (P00C1)	• Yes<br>○ Compassionate release to receive MAiD (P00C1)<br>○ Logistics arrangements are made for patients and families (P00B5)

Jurisdiction	Does the program have experience providing MAiD related support to this population?	Does the MAiD program have any policies in place to support this population?	How does the MAiD program work with population specific support?	How are assessments completed? (virtual, in person, not described)	Can the program accommodate patients and families' preferences and practices?
Yukon - *Department of Health and Social Services*	• No (P00C7, P00C14)	• No (P00C7, P00C14)	• Not applicable	• Not applicable	• Not applicable
Ontario - *Ministry of Health*	• Not applicable<br>○ No involvement in service delivery	• Not applicable<br>○ No involvement in service delivery	• Not applicable<br>○ No involvement in service delivery	• Not applicable<br>○ No involvement in service delivery	• Not applicable<br>○ No involvement in service delivery
SERVICE ORGANIZATIONS/REGIONAL FACILITIES					
Ontario - *Home and Community Care Support Services, Central East*	• No (P00C2)	• No (P00C2)	• Not applicable	• Not applicable	• Not applicable
Ontario - *Home and Community Care Support Services, Waterloo Region*	• Yes (P00C4)	• Policies supporting incarcerated populations not specified	• Referral to provider for assessment and provision<br>○ Palliative care center (P00C4)	• In person (P00C4)	• Yes<br>○ Compassionate release to receive MAiD (P00C4)
Ontario - *Home and Community Care Support Services, South East*	• Yes (P00C20)	• No (P00C20)	• Referral to care coordination service or practitioners for assessment and provision	• In person (P00C20)	• Accommodation of wishes for incarcerated populations not specified
Ontario - *Home and Community Care Support Services, South West*	• No (P00C16, P00C23)	• No (P00C16, P00C23)	• Not applicable<br>○ No experience	• Not applicable<br>○ No experience	• Not applicable<br>○ No experience
Ontario - *Champlain Regional MAiD Network, the Ottawa Hospital*	• Yes (P00C5)	• No (P00C5)	• Collaborative effort between correctional facilities and health authority to streamline assessment and provision (P00C5)<br>• Referral to healthcare facility for assessment and provision<br>○ Correctional facility (P00C5)	• In person (P00C5)<br>• Virtual (P00C5)	• No<br>○ Patient and family preferences may not be accommodated due to constraints (P00C5)
HEALTHCARE FACILITIES					
Ontario - *Peterborough Regional Health Centre*	• No (P00C3)	• Policies supporting incarcerated populations not specified	• Not applicable<br>○ No experience	• Not applicable<br>○ No experience	• Not applicable<br>○ No experience
Ontario - *Mount Sinai Healthcare Facility, Toronto*	• No (P00B18)	• No (P00B18)	• Not applicable<br>○ No experience	• Not applicable<br>○ No experience	• Not applicable<br>○ No experience
Ontario - *University Health Network*	• No (P00C8)	• No (P00C8)	• Not applicable<br>○ No experience	• Not applicable<br>○ No experience	• Not applicable<br>○ No experience
Ontario - *Grand River Hospital*	• No (P00C50)	• No (P00C50)	• Not applicable<br>○ No experience	• Not applicable<br>○ No experience	• Not applicable<br>○ No experience
COMMUNITY OF PRACTICE					
Ontario - *Hamilton Family Health Team*	• No (P00B18)	• Policies supporting incarcerated populations not specified	• Not applicable<br>○ No experience	• Not applicable<br>○ No experience	• Not applicable<br>○ No experience
Ontario - *Niagara Community MAiD Team, St. Catharines, Niagara*	• No (P00C11)	• No (P00C11)	• Not applicable<br>○ No experience	• Not applicable<br>○ No experience	• Not applicable<br>○ No experience
GEOGRAPHICAL AREAS					
Ontario - *Oakville & Mississauga area*	• No (P00C18)	• Policies supporting incarcerated populations not specified	• Not applicable<br>○ No experience	• Not applicable<br>○ No experience	• Not applicable<br>○ No experience
Ontario - *Oakville, Mississauga & Burlington area*	• No (P00C21)	• Policies supporting incarcerated populations not specified	• Not applicable<br>○ No experience	• Not applicable<br>○ No experience	• Not applicable<br>○ No experience
Ontario - *Renfrew County*	• No (P00C22)	• No (P00C22)	• Not applicable<br>○ No experience	• Not applicable<br>○ No experience	• Not applicable<br>○ No experience
Ontario - *Greater Toronto Area*	• No (P00C24)	• No (P00C24)	• Not applicable<br>○ No experience	• Not applicable<br>○ No experience	• Not applicable<br>○ No experience
Ontario - *Waterloo area*	• No (P00C25)	• No (P00C25)	• Not applicable<br>○ No experience	• Not applicable<br>○ No experience	• Not applicable<br>○ No experience
Ontario - *Noelville, Sudbury, Elliot Lake, Sturgeon Falls area*	• No (P00C26)	• No (P00C26)	• Not applicable<br>○ No experience	• Not applicable<br>○ No experience	• Not applicable<br>○ No experience
Québec - *Ministry of Health and Social Services*	• Not applicable	• Not applicable<br>○ No involvement in service delivery	• Not applicable<br>○ No involvement in service delivery	• Not applicable<br>○ No involvement in service delivery	• Not applicable<br>○ No involvement in service delivery

Jurisdiction	Does the program have experience providing MAiD related support to this population?	Does the MAiD program have any policies in place to support this population?	How does the MAiD program work with population specific support?	How are assessments completed? (virtual, in person, not described)	Can the program accommodate patients and families' preferences and practices?
	○ No involvement in service delivery				
INTEGRATED HEALTH AND SOCIAL SERVICES CENTRES					
Québec – *CISS Montérégie*	• No (P00C27)	• No (P00C27)	• Not applicable<br>○ No experience	• Not applicable<br>○ No experience	• Not applicable<br>○ No experience
INTEGRATED UNIVERSITY HEALTH AND SOCIAL SERVICES CENTRES					
Québec – *CIUSS Capitale-Nationale*	• No (P00C37)	• No (P00C37)	• Not applicable<br>○ No experience	• Not applicable<br>○ No experience	• Not applicable<br>○ No experience
Québec – *University of Montreal Hospital Center*	• No (P00C40)	• No (P00C40)	• Not applicable<br>○ No experience	• Not applicable<br>○ No experience	• Not applicable<br>○ No experience
Québec – *McGill University Health Centre*	• No (P00C9)	• No (P00C9)	• Not applicable<br>○ No experience	• Not applicable<br>○ No experience	• Not applicable<br>○ No experience

Table 19 Special considerations – Indigenous population

Jurisdiction	Does the MAiD program have any policies in place to support this population specifically?	How does the MAiD program work with population-specific supports or resources in the community? (liaisons, representatives from communities, navigators, and specialized organizations, First Nations Health Authority, Elders, social workers)	Where can MAiD be provided?	Can the program accommodate cultural and religious practices?
Alberta - *Alberta Health Services*	• No (P00A5, P00B9, P00C6, P00B13)	• MAiD team collaborates with Indigenous liaison (Calgary hospitals) (P00A5, P00B9, P00C6, P00B13)<br>• MAiD team collaborates with Northern communities and First Nations Coordinators (P00B9)<br>○ To facilitate MAiD provision on reserves (P00B9)	• Reserves (P00B9)	• Yes<br>○ Patient and family preferences accommodated upon request (P00A5, P00B9, P00C6, P00B13)
British Columbia - *Ministry of Health*	• Not applicable	• Not applicable	• Not applicable	• Not applicable
British Columbia - *Fraser Health*	• Policies to support Indigenous populations not specified	• MAiD team collaborates with Indigenous liaison (P00A10)<br>○ To connect patients and families with community resources (P00A10)<br>• MAiD team engages First Nations Health Authority (P00A10)<br>○ To seek guidance regarding Indigenous MAiD cases (P00A10)	• Hospital (P00C17)<br>• Home (P00A10)<br>• Hospice (P00A10)<br>• Long term care (P00A10)<br>• Reserves (P00A10)<br>• Outdoor space (P00A10)	• Yes<br>○ Patient and family preferences accommodated upon request (P00C17)<br>▪ Smudging ceremonies (P00C17)
British Columbia - *Interior Health*	• No (P00B14, P00C41)	• MAiD team collaborates with Indigenous liaison (Indigenous leader at acute care centre) (P00B14)<br>○ To seek guidance and facilitate MAiD support for patients and families in a culturally sensitive way (P00B14)<br>• MAiD team engages First Nations Health Authority (P00B14)<br>○ To seek guidance regarding Indigenous MAiD cases (P00B14)	• Reserves (P00C29)<br>• Hospice (P00C41)<br>• Home (P00C41)	• Yes<br>○ Patient and family preferences accommodated upon request (P00B14, P00C41, P00C29)<br>▪ Spiritual support and rituals can be requested (P00B14)
British Columbia - *Island Health*	• No (P00A9)	• MAiD team collaborates with Indigenous liaison nurse (works with patients in acute care) (P00A9)<br>○ To facilitate MAiD support for patients and families in a culturally sensitive way (P00A9)<br>• MAiD team engages First Nations Health Authority (FNHA) (P00A9)<br>○ To facilitate traditional ceremonies during MAiD provision (P00A9)	• Reserves (P00A13)<br>• Hospital (P00A13)<br>• Home (P00A9)<br>• Patient and family preference (P00A9)	• Yes<br>○ Patient and family preferences accommodated upon request (P00A9)
British Columbia - *Northern Health*	• Policies to support Indigenous populations not specified	• Collaboration with Indigenous supports not specified	• Hospital (P00A8)<br>• Home (P00A8)	• Yes<br>○ Patient and family preferences accommodated upon request (P00A8)<br>▪ Smudging ceremonies (P00A8)
British Columbia - *Vancouver Coastal Health*	• No (P00A7, P00B15)	• MAiD team collaborates with Indigenous liaison (Regional Indigenous Engagement Lead) (P00A7, P00B10, P00B15)<br>○ To support in resolving difficult situations (community resistance to MAiD) and engages with community (P00B15)<br>○ To create engagement plan and support engagement with First Nations and Métis community (P00A7)<br>• MAiD team engages First Nations Health Authority<br>○ To provide indigenous-specific support like Elders (P00A7, P00B10)	• Reserves (P00B16)<br>• Hospital (P00B10)<br>• Home (P00A7)	• Yes<br>○ Patient preferences accommodated upon request (P00A7)
British Columbia - *Provincial Health Services Authority*	• Policies to support Indigenous populations not specified	• Collaboration with Indigenous supports not specified	• Hospital (P00A11)<br>• Home (P00A11)	• Cultural and religious accommodations not specified
Manitoba - *Shared Health*	• No (P00C33, P00C34)	• MAiD team collaborates with Indigenous liaisons (Indigenous physician MAiD providers) (P00B21, P00C33)<br>○ To facilitate MAiD support for patients and families in a culturally sensitive way (P00C33)<br>○ To facilitate MAiD assessment and provision for patients requiring Indigenous MAiD provider (P00B21)<br>• MAiD team collaborates with a network of community spiritual care providers (P00B21)<br>○ To connect patients and families with spiritual care providers	• Reserves (P00C33, P00C34, P00B21)	• Yes<br>○ Patient and family preferences accommodated upon request (P00C33, P00C34, P00B21)<br>▪ Involvement of spiritual care providers if requested (P00B21)

Jurisdiction	Does the MAiD program have any policies in place to support this population specifically?	How does the MAiD program work with population-specific supports or resources in the community? (liaisons, representatives from communities, navigators, and specialized organizations, First Nations Health Authority, Elders, social workers)	Where can MAiD be provided?	Can the program accommodate cultural and religious practices?
New Brunswick - *Horizon Health Network*	• No (P00B11)	• MAiD team collaborates with Indigenous liaisons (P00B11, P00C15)<br>○ To connect patients and families with Elders and accommodates Indigenous practices during MAiD provision (P00B11, P00C15)<br>○ To connect patients and families with community resources (P00B11)	• Patient and family preference (P00B11)	• Yes<br>○ Patient and family preferences accommodated upon request (P00B11)
New Brunswick - *Vitalité Health Network*	• No (P00C48)	• Providers connects patients and families with community resources (P00C48)	• Reserves (P00C48)<br>• Patient and family preference (P00C48)	• Yes<br>○ Patient and family preferences accommodated upon request (P00C48)
Newfoundland and Labrador - *Department of Health and Community Services*	• Not applicable	• Not applicable	• Not applicable	• Not applicable
Newfoundland and Labrador - *Eastern Zone*	• No (P00C43)	• Collaboration with Indigenous supports not specified	• Patient and family preference (P00C43)	• Yes<br>○ Patient and family preferences accommodated upon request (P00C43)
Newfoundland and Labrador - *Western Zone*	• No (P00C44)	• MAiD team collaborates with local Indigenous band office (P00C44)<br>○ To connect patients and families with community Elders and community social workers<br>• MAiD team collaborates with regional Indigenous patient navigators (P00C44)<br>○ To support patient and families navigate MAiD process (P00C44)	• Patient and family preference (P00C44)<br>○ Local Indigenous band office (P00C44)	• Yes<br>○ Patient and family preferences accommodated upon request (P00C44)
Newfoundland and Labrador - *Central Zone*	• Policies to support Indigenous populations not specified	• Collaboration with Indigenous supports not specified	• Where provisions can occur not specified	• Cultural and religious accommodations not specified
Northwest Territories	• No (P00A3)	• MAiD team connects patients and families with Indigenous patient advocates (P00A3)<br>• MAiD team connects patients and families with Stanton Indigenous wellness program (P00A3)	• Hospital (P00C35)<br>• Home (P00A3)<br>• Patient and family preference (P00A3)<br>○ Rooms and space for family (P00A3)	• Yes<br>○ Patient and family preferences accommodated upon request (P00A3)
Nova Scotia - *Nova Scotia Health*	• Policies to support Indigenous populations not specified	• Collaboration with Indigenous supports not specified	• Reserves (P00A12)	• Yes<br>○ Patient and family preferences accommodated upon request (P00A12)<br>▪ Smudging ceremonies (P00A12)
Prince Edward Island - *Health PEI*	• No (P00B8)	• MAiD team collaborates with Indigenous liaisons (Indigenous navigators working in PEI Health Authority) (P00B8)<br>○ To facilitate MAiD support for patients and families in a culturally sensitive way<br>• MAiD team collaborates with Indigenous Native Council (P00B8)<br>○ To facilitate access to MAiD information for Indigenous communities<br>▪ Give presentation on MAiD to Native Council, Indigenous health care teams and community members (P00B8)	• Hospital (P00B8)<br>• Patient and family preference (P00B8)	• Yes<br>○ Patient preferences accommodated upon request (P00B8)
Saskatchewan - *Saskatchewan Health Authority*	• No (P00A1, P00B1))	• MAiD team engages practitioners working with Indigenous community advisors (P00A1)<br>○ To inform MAiD practices facilitate provincial in a culturally sensitive way<br>• MAiD team collaborates with Health Canada (P00C1)<br>○ To build partnerships with Indigenous communities<br>• MAiD team collaborates with social workers in community (P00B3)<br>○ To facilitate MAiD assessments for patients<br>• MAiD team collaborates with First Nations Métis health partners (P00B7)<br>○ To facilitate MAiD support for patients and families	• Reserves (P00B1, P00C1)<br>• Patient and family preference (P00C1)	• Yes<br>○ Patient and family preferences accommodated upon request (P00A1)
Yukon - *Department of Health and Social Services*	• Policies to support Indigenous populations not specified	• Providers refers patients and families to Indigenous health program (First Nations Health Program) in hospital to support patient and families (P00C7, P00C14)	• Patient and family preference (P00C7)<br>• Hospitals (P00C7)	• Yes<br>○ Patient and family preferences accommodated upon request (P00C7)<br>▪ Involvement of ministers and choirs (P00C7)
Ontario - *Ministry of Health*	• Not applicable	• Not applicable	• Not applicable	• Not applicable

Jurisdiction	Does the MAiD program have any policies in place to support this population specifically?	How does the MAiD program work with population-specific supports or resources in the community? (liaisons, representatives from communities, navigators, and specialized organizations, First Nations Health Authority, Elders, social workers)	Where can MAiD be provided?	Can the program accommodate cultural and religious practices?
Ontario - *Home and Community Care Support Services, Central East*	• No (P00C2)	• MAiD team collaborates with Indigenous liaison to support Indigenous patients and families (P00C2)	• Patient preference (P00C2)	• Yes<br>○ Patient preferences accommodated upon request (P00C2)
Ontario - *Home and Community Care Support Services, Waterloo Region*	• No (P00C4)	• Collaboration with Indigenous supports not specified	• Patient preference (P00C4)	• Yes<br>○ Patient preferences accommodated upon request (P00C4)
Ontario - *Home and Community Care Support Services, South East*	• Policies to support Indigenous populations not specified	• Collaboration with Indigenous supports not specified	• Reserves (P00C20)<br>• Patient preference (P00C20)	• Yes<br>○ Patient preferences accommodated upon request (P00C20)
Ontario - *Home and Community Care Support Services, South West*	• No (P00C23)	• Collaboration with Indigenous supports not specified	• Reserve (P00C23)	• Yes<br>○ Patient preferences accommodated upon request (P00C23)
Ontario - *Champlain Regional MAiD Network, the Ottawa Hospital*	• No (P00C5)	• Collaboration with Indigenous supports not specified	• Hospital (P00C5)<br>• Patient preference (P00C5)	• Yes<br>○ Patient preferences accommodated upon request (P00C5)<br>▪ Smudging ceremonies (P00C5)
HEALTHCARE FACILITIES				
Ontario - *Peterborough Regional Health Centre*	• No (P00C3)	• MAiD team collaborates with Indigenous liaison (spiritual care provider in hospital) (P00C3)<br>○ To accommodates Indigenous practices during MAiD provision	• Patient preference (P00C3)	• Yes<br>○ Patient preferences accommodated upon request (P00C3)<br>▪ Smudging ceremonies (P00C3)
Ontario - *Mount Sinai Healthcare Facility, Toronto*	• Policies to support Indigenous populations not specified	• Collaboration with Indigenous supports not specified	• Where provisions can occur not specified	• Cultural and religious accommodations not specified
Ontario - *University Health Network*	• No (P00C8)	• MAiD team collaborates with Indigenous liaison (Indigenous navigator) (P00C8)<br>○ To facilitate MAiD support for patients and families in a culturally sensitive way<br>○ To accommodate Indigenous practices during MAiD provision	• Hospital (P00C8)	• Yes<br>○ Patient preferences accommodated upon request (P00C8)<br>▪ Smudging ceremonies (P00C8)<br>▪ Prayers (P00C8)
Ontario - *Grand River Hospital*	• No experience (P00C50)	• No experience (P00C50)	• No experience (P00C50)	• No experience (P00C50)
Ontario - *Hamilton Family Health Team*	• No (P00B18)	• MAiD team collaborates with Indigenous liaison (advocate) (P00B18)<br>○ To connect with Indigenous representatives (leaders) and healthcare providers (physicians) and provide MAiD support for patients and families in a culturally sensitive way (P00B18)	• Reserves (P00B18)<br>• Patient preference (P00B18)	• Yes<br>○ Patient preferences accommodated upon request (P00B18)
Ontario - *Niagara Community MAiD Team, St. Catharine's, Niagara*	• No (P00C11)	• Not applicable<br>○ No experience (P00C11)	• Not applicable<br>○ No experience (P00C11)	• Not applicable<br>○ No experience (P00C11)
GEOGRAPHICAL AREAS				
Ontario - *Oakville & Mississauga area*	• No experience (P00C18)	• Not applicable<br>○ No experience (P00C18)	• Not applicable<br>○ No experience (P00C18)	• Not applicable<br>○ No experience (P00C18)
Ontario - *Oakville, Mississauga & Burlington area*	• No experience (P00C21)	• Not applicable<br>○ No experience (P00C21)	• Not applicable<br>○ No experience (P00C21)	• Not applicable<br>○ No experience (P00C21)
Ontario - Renfrew County	• No (P00C22)	• Not applicable	• Not applicable	• Not applicable
Ontario - *Greater Toronto Area*	• No (P00C24)	• Not applicable	• Not applicable	• Not applicable
Ontario - *Waterloo Area*	• No (P00C25)	• Not applicable	• Hospital (P00C25)	• Yes<br>○ Patient preferences accommodated upon request (P00C25)

Jurisdiction	Does the MAiD program have any policies in place to support this population specifically?	How does the MAiD program work with population-specific supports or resources in the community? (liaisons, representatives from communities, navigators, and specialized organizations, First Nations Health Authority, Elders, social workers)	Where can MAiD be provided?	Can the program accommodate cultural and religious practices?
				▪ Involvement of ministers and choirs (P00C25)
Ontario - *Noelville, Sudbury, Elliot Lake, Sturgeon Falls area*	• No (P00C26)	• Provider collaborates with palliative care physician from Indigenous community (P00C26)<br>○ To facilitates MAiD support for patients and families in a culturally sensitive way	• Hospital (P00C26)<br>• Patient preference (P00C26)	• Yes<br>○ Patient preferences accommodated upon request (P00C26)
Québec - *Ministry of Health and Social Services*	• Not applicable	• Not applicable	• Not applicable	• Not applicable
INTEGRATED HEALTH AND SOCIAL SERVICES CENTRES				
Québec – *CISS Montérégie*	• No (P00C27)	• Not applicable	• Not applicable	• Not applicable
INTEGRATED UNIVERSITY HEALTH AND SOCIAL SERVICES CENTRES				
Québec – *CIUSS Capitale-Nationale*	• No (P00C37)	• Not applicable	• Not applicable	• Not applicable
Québec – *University of Montreal Hospital Center*	• No (P00C40)	• Not applicable	• Not applicable	• Not applicable
Québec – *McGill University Health Centre*	• No (P00C9)	• Not applicable	• Not applicable	• Not applicable

Table 20: Special considerations – unhoused population

Jurisdiction	Does the MAiD program have any policies in place to support this population specifically?	How does the MAiD program work with population-specific supports in the community?	What access points to MAiD exist for this specific population?
Alberta - *Alberta Health Services*	• No (P00A5)	• MAiD team collaborates with Palliative Care Program (PCP) in Calgary (P00B13)<br>○ To facilitate access to information<br>○ To connect with nurse liaison in PCP to locate patients (P00B13)<br>• MAiD team collaborates with community health center (Radius) (P00B13, P00C6)<br>○ To facilitate MAiD requests from patients and support patients and families to complete record of request and connect with MAiD navigators<br>• MAiD team collaborates with social workers in specialized palliative care facilities, who house vulnerable populations in Edmonton (P00C6)<br>○ To support patients and families to navigate the MAiD process with MAiD team	• Hospital (P00B13)<br>• Community partners (P00C6)<br>○ Health center (Radius) (P00C6)<br>○ Specialized palliative care facilities (P00C6)<br>○ Community health care clinics (P00B13)
British Columbia - *Ministry of Health*	• Not applicable	• Not applicable	• Not applicable
British Columbia - *Fraser Health*	• No (P00A10)	• MAiD team connects with available and existing resources (P00A10)<br>○ To support patients and families navigate MAiD process with MAiD team (P00A10)	• Hospital (P00A10)<br>• Community partner (P00A10)
British Columbia - *Interior Health*	• Policies supporting unhoused populations not specified	• MAiD team collaborates with community social workers (P00B14)<br>○ To locate patients, secure housing or facilitate MAiD provision (P00B14)	• Hospital (P00C41)<br>• Community social workers (P00C29)
British Columbia -*Island Health*	• Policies supporting unhoused populations not specified	• MAiD team connects with available resources in the community (outreach palliative worker in palliative end-of-life team) (P00A9)<br>○ To support patients and families to navigate the MAiD process with MAiD team	• Community partner (P00A9)<br>○ Outreach educator (P00A9
British Columbia - *Northern Health*	• Policies supporting unhoused populations not specified	• Collaboration with unhoused population supports not specified	• Access to MAiD for unhoused populations not specified
British Columbia - *Vancouver Coastal Health*	• Policies supporting unhoused populations not specified	• MAiD team connects with available resources in community (community support and outreach teams) (P00B16)<br>○ To support patients and families to navigate the MAiD process with MAiD team<br>• MAiD team collaborates with community social workers (community educator) (P00B12)<br>○ To locate patients and families (P00B12)	• Hospital (P00A7)<br>• Community partner (P00B16)<br>○ Outreach educator (P00B16)<br>○ Case managers (P00B16)<br>• Palliative and end-of-life care facilities (P00B15)
British Columbia - *Provincial Health Services Authority*	• Policies supporting unhoused populations not specified	• Collaboration with unhoused population supports not specified	• Access to MAiD for unhoused populations not specified
Manitoba - *Shared Health*	• No (P00B19)	• MAiD team collaborates with hospital social workers (P00B21)<br>○ To facilitate necessary arrangements (including paperwork) for inpatients to navigate MAiD process with MAiD team<br>• MAiD team collaborates with residential facilities providing temporary housing (P00B21)<br>○ To facilitate MAiD provision<br>• MAiD team collaborates with community social workers (in care facilities) (P00C33)<br>○ To supports patients and families and connect them with community resources	• Hospital (P00B21, P00C34)<br>○ Hospital social workers (P00B21)<br>• Community partner (P00C33, P00B21)<br>○ Community social workers in care facilities (P00C33)
New Brunswick - *Horizon Health Network*	• No (P00B11, P00C15)	• MAiD team collaborates with nurse practitioners in community clinic (Salvus Clinic) (P00B11)<br>○ To locate patients and families and provide case management support	• Hospital (P00C15)<br>• Community partner (P00B11)<br>○ Clinic (Salvus Clinic) (P00B11)
New Brunswick - *Vitalité Health Network*	• No (P00C48)	• Collaboration with unhoused population supports not specified	• Access to MAiD for unhoused populations not specified
Newfoundland and Labrador- *Department of Health and Community Services*	• Not applicable	• Not applicable	• Not applicable
Newfoundland and Labrador - *Eastern Zone*	• Policies supporting unhoused populations not specified	• Collaboration with unhoused population supports not specified	• Access to MAiD for unhoused populations not specified
Newfoundland and Labrador - *Western Zone*	• Policies supporting unhoused populations not specified	• Collaboration with unhoused population supports not specified	• Access to MAiD for unhoused populations not specified
Newfoundland and Labrador - *Central Health*	• Policies supporting unhoused populations not specified	• Collaboration with unhoused population supports not specified	• Hospital (P00B22)
Northwest Territories	• No (P00A3)	• MAiD team collaborates with outreach team in community (P00C35)<br>○ To locate patients and families and navigate MAiD process (P00A3)<br>○ Territorial MAiD specialists connect with patients and families in their preferred setting (P00A3)	• Hospitals (P00C35)<br>• Community (P00C35)<br>○ Shelters (P00A3)<br>○ Outreach team (non-medical) (P00A3)

Jurisdiction	Does the MAiD program have any policies in place to support this population specifically?	How does the MAiD program work with population-specific supports in the community?	What access points to MAiD exist for this specific population?
Nova Scotia - *Nova Scotia Health*	• Policies supporting unhoused populations not specified	• MAiD team collaborates with community organization (Mobile Outreach Street Health (MOSH) (P00A12)<br>○ To facilitate MAiD provision in community healthcare facilities (P00A12)	• Community partner (P00A12)<br>○ Mobile Outreach Street Health (P00A12)
Prince Edward Island -*Health PEI*	• No (P00B8, P00B17)	• MAiD team collaborates with community partners in care facilities (long-term care facilities, clinics, cancer treatment centers) (P00B8)<br>○ To facilitate MAiD assessment and provision with MAiD team<br>• MAiD team collaborates with Allied Health team (P00B8)<br>○ To connect patients and families with MAiD team and their services<br>• MAiD team collaborates with specialists (mental health, addictions, drug rehabilitation) in community (P00B17)<br>○ To support patients and families navigate MAiD process	• Hospital (P00B8)<br>• Community partners (P00B17)<br>○ Community social workers in care facilities (P00B8)
Saskatchewan - *Saskatchewan Health Authority*	• Policies supporting unhoused populations not specified	• MAiD team identifies resources in community (P00C1)<br>○ To arrange locations for MAiD assessment and provision<br>• MAiD team collaborates with social workers in community (P00B3)<br>○ To facilitate MAiD assessment with MAiD team	• Hospital (P00A1)
Yukon - *Department of Health and Social Services*	• Policies supporting unhoused populations not specified	• Providers collaborates with specialized community clinic (P00C14)<br>○ To identify unhoused patients and families with dual diagnosis and facilitate outreach services in homeless shelter	• Hospital (P00C14)<br>• Community partner (P00C14)<br>○ Clinic (Whitehorse) (P00C14)
Ontario - *Ministry of Health*	• Not applicable	• Not applicable	• Not applicable
SERVICE ORGANIZATIONS/ REGIONAL FACILITIES			
Ontario - *Home and Community Care Support Services, Central East*	• No (P00C2)	• MAiD team collaborates with an agency (Fourcast) to deliver health or MAiD services (P00C2)	• Community partner (P00C2)<br>○ Agency (Fourcast) (P00C2)
Ontario - *Home and Community Care Support Services, Waterloo Region*	• Policies supporting unhoused populations not specified	• Collaboration with unhoused population supports not specified	• Access to MAiD for unhoused populations not specified
Ontario - *Home and Community Care Support Services, South East*	• Policies supporting unhoused populations not specified	• Collaboration with unhoused population supports not specified	• Access to MAiD for unhoused populations not specified
Ontario - *Home and Community Care Support Services, South West*	• Policies supporting unhoused populations not specified	• Collaboration with unhoused population supports not specified	• Access to MAiD for unhoused populations not specified
Ontario - *Champlain Regional MAiD Network, the Ottawa Hospital*	• No (P00C5)	• No experience (P00C5)	• Not applicable
HEALTHCARE FACILITIES			
Ontario - *Peterborough Regional Health Centre*	• No (P00C3)	• Collaboration with unhoused population supports not specified	• Access to MAiD for unhoused populations not specified
Ontario - *Mount Sinai Healthcare Facility, Toronto*	• Policies supporting unhoused populations not specified	• Collaboration with unhoused population supports not specified	• Access to MAiD for unhoused populations not specified
Ontario - *University Health Network*	• No (P00C8)	• Not applicable	• Not applicable
Ontario - *Grand River Hospital*	• No (P00C50)	• Not applicable	• Not applicable
COMMUNITY OF PRACTICE			
Ontario - *Hamilton Family Health Team*	• Policies supporting unhoused populations not specified	• MAiD team collaborates with community partners (funeral home) (P00B18)<br>○ To facilitate MAiD provision with MAiD team	• Community partner (P00B18)<br>○ Funeral home (Circle of Life) (P00B18)
Ontario - *Niagara Community MAiD Team, St. Catharine's, Niagara*	• Policies supporting unhoused populations not specified	• Collaboration with unhoused population supports not specified	• Access to MAiD for unhoused populations not specified
GEOGRAPHICAL AREAS			
Ontario - *Oakville & Mississauga area*	• Not applicable	• Not applicable	• Not applicable
Ontario - *Oakville, Mississauga & Burlington area*	• Not applicable	• Collaboration with unhoused population supports not specified	• Hospital (P00C21)
Ontario - *Renfrew County*	• Not applicable	• Provider collaborates with community partners (local hospital, hospice, and community paramedics) (P00C22)<br>○ To facilitate MAiD assessment and provision	• Hospital (P00C22)<br>• Community partners (P00C22)<br>○ Community paramedics (P00C22)
Ontario - *Greater Toronto Area*	• Not applicable	• Provider collaborates with a local facility (MAiDHouse) (P00C24)<br>○ To facilitate MAiD provision	• Hospital (P00C24)<br>• Community partner (P00C24)<br>○ MAiDHouse (P00C24)

Jurisdiction	Does the MAiD program have any policies in place to support this population specifically?	How does the MAiD program work with population-specific supports in the community?	What access points to MAiD exist for this specific population?
Ontario - *Waterloo area*	• Not applicable	• Provider facilitates connection with community supports (P00C25)	• Community partners (P00C25)<br>○ Community social workers in care facilities (P00C25)
Ontario - *Noelville, Sudbury, Elliot Lake, Sturgeon Falls Areas*	• Not applicable	• Not applicable	• Not applicable
Québec - *Ministry of Health and Social Services*	• Not applicable	• Not applicable	• Not applicable
INTEGRATED HEALTH AND SOCIAL SERVICES CENTRES			
Québec – *CISS Montérégie*	• No (P00C27)	• Not applicable	• Not applicable
INTEGRATED UNIVERSITY HEALTH AND SOCIAL SERVICES CENTRES			
Québec – *CIUSS Capitale-Nationale*	• No (P00C37)	• Not applicable	• Not applicable
Québec – *University of Montreal Hospital Center*	• No (P00C40)	• Not applicable	• Not applicable
Québec – *McGill University Health Centre*	• No (P00C9)	• Not applicable	• Not applicable

Table 21: Training and education for MAiD team and other healthcare professionals

Jurisdiction	What types of training does the MAiD program offer?	What kind of educational sessions does the MAiD program offer?
Alberta - *Alberta Health Services*	• Location<br>○ Virtual<br>• Support MAiD-related medication dispensation and reporting<br>○ Pharmacists (Community and hospital) (P00C12)	• Location<br>○ In person (P00B13)<br>• Offer in-services to healthcare providers and facilities (P00B13)<br>○ Tailor services to specific need (P00B13)
British Columbia - *Ministry of Health*	• Not applicable	• Not applicable
British Columbia - *Fraser Health*	• Location<br>○ Online (P00A10)<br>○ In person (P00A10)<br>• Support learning on MAiD process (P00A10)<br>○ Healthcare staff (P00A10)	• Location<br>○ In person (P00A10)<br>• Educate healthcare providers about MAiD process<br>○ Lunch and learns (P00A10)<br>○ Community of practice sessions (P00A10)
British Columbia - *Interior Health*	• Training for other healthcare professionals not specified	• Education for other healthcare professionals not specified
British Columbia - *Island Health*	• Location<br>○ Online (P00A13)<br>○ In person (P00A9)<br>• Support learning on MAiD process (P00A13)<br>○ MAiD program team (P00A13)<br>▪ Compensates nurse practitioners and coordination team with CAMAP registration costs (P00A9)	• Location<br>○ In person<br>• Educate healthcare providers about MAiD process and available resources for patients and families (MAiD program, pamphlets, brochures, instruction booklet)<br>○ Frontline healthcare providers (P00A9, P00C13)<br>○ Social workers and nursing colleges (P00A9)<br>○ Care coordinators, legal professionals and speech pathologists (P00C13)<br>○ Contracted healthcare clinic staff (P00A9)
British Columbia - *Northern Health*	• Training for other healthcare professionals not specified	• Education for other healthcare professionals not specified
British Columbia - *Vancouver Coastal Health*	• Location<br>○ Virtual (P00B15)<br>• Available to support learning on MAiD process<br>○ Healthcare staff (P00B15)	• Location<br>○ In person<br>• Educate healthcare providers about MAiD process and available resources (P00B12)<br>○ Frontline healthcare providers (P00B12)
British Columbia - *Provincial Health Services Authority*	• Training for other healthcare professionals not specified	• Education for other healthcare professionals not specified
Manitoba - *Shared Health*	• Location<br>○ In person (P00B19)<br>○ Virtual (P00B19)<br>• Orientation and onboarding (P00B19)<br>• Referral to educational resources (P00B19)<br>○ CAMAP (P00B19)	• Location<br>○ In person<br>• Educate healthcare providers about MAiD process and available resources (P00C34)<br>○ Frontline healthcare providers (P00C34)
New Brunswick - *Horizon Health Network*	• Training for other healthcare professionals not specified	• Location<br>○ In person<br>• Educate healthcare providers about MAiD process and available resources (P00B11)<br>○ Community nurses (P00B11)
New Brunswick - *Vitalité Health Network*	• None (P00C48)	• Not applicable
Newfoundland and Labrador - *Department of Health and Community Services*	• Not applicable	• Not applicable
Newfoundland and Labrador - *Eastern Zone*	• Training for other healthcare professionals not specified	• Education for other healthcare professionals not specified
Newfoundland and Labrador - *Western Zone*	• Training for other healthcare professionals not specified	• Education for other healthcare professionals not specified
Newfoundland and Labrador - *Central Zone*	• Training for other healthcare professionals not specified	• Education for other healthcare professionals not specified
Northwest Territories	• Location<br>○ In person (P00A3)<br>○ Virtual (P00A3)<br>• Referral to educational resources<br>○ CAMAP (P00A3)<br>• Support for registered nurses to acquire scope-specific skills (P00A3)	• Location<br>○ In person (P00A3)<br>○ Virtual (P00A3)<br>• Educate healthcare providers with MAiD process, their responsibilities and available resources<br>○ Frontline healthcare providers (P00A2)<br>○ Home care staff (P00A2)<br>○ Allied Health staff (P00A2, P00A3)<br>• Provide presentations at grand rounds (P00A3)
Nova Scotia - *Nova Scotia Health*	• Training for other healthcare professionals not specified	• Education for other healthcare professionals not specified

Jurisdiction	What types of training does the MAiD program offer?	What kind of educational sessions does the MAiD program offer?
Prince Edward Island - *Health PEI*	• Location<br>○ In person (P00B8)<br>○ Virtual (P00B8)<br>• Orientation and onboarding (P00B8)<br>○ For Clinical MAiD program staff (P00B8)	• Location<br>○ In person<br>• Educate healthcare staff, specific populations (P00B8)<br>○ Home care nurses (P00B17)
Saskatchewan - *Saskatchewan Health Authority*	• Location<br>○ In person (P00B1)<br>○ Virtual (P00B1)<br>• Education and resources for healthcare staff (P00A1)	• Location<br>○ In person<br>• Educate healthcare staff about MAiD process (P00A1)<br>○ Palliative care physicians (P00A1)
Yukon - *Department of Health and Social Services*	• Training for other healthcare professionals not specified	• Education for other healthcare professionals not specified
Ontario - *Ministry of Health*	• Location<br>○ Online (P00B4)<br>• Referral to education resources<br>○ CAMAP (P00B4)	• Not applicable
SERVICE ORGANIZATIONS/REGIONAL FACILITIES		
Ontario - *Home and Community Care Support Services, Central East*	• None (P00C2)	• Location<br>○ In person<br>• Educate healthcare staff about MAiD process<br>○ Long-term care staff (P00C2)
Ontario - *Home and Community Care Support Services, Waterloo Region*	• Training for other healthcare professionals not specified	• Education for other healthcare professionals not specified
Ontario - *Home and Community Care Support Services, South East*	• Location<br>○ Virtual (P00C20)<br>• Support learning on MAiD process<br>○ Healthcare staff (P00C20)	• Location<br>○ In person<br>• Educate healthcare staff about MAiD process, eligibility, understanding of the law<br>○ Home care staff (P00C20)<br>○ MAiD care coordinators (P00C20)
Ontario - *Home and Community Care Support Services, South West*	• Training for other healthcare professionals not specified	• Education for other healthcare professionals not specified
Ontario - *Champlain Regional MAiD Network, the Ottawa Hospital*	• None (P00C5)	• None (P00C5)
HEALTHCARE FACILITIES		
Ontario - *Peterborough Regional Health Centre*	• Training for other healthcare professionals not specified	• Education for other healthcare professionals not specified
Ontario - *Mount Sinai Healthcare Facility, Toronto*	• Training for other healthcare professionals not specified	• Education for other healthcare professionals not specified
Ontario - *University Health Network*	• Location<br>○ Online (P00C8)<br>• Orientation and onboarding (P00C8)<br>○ MAiD coordination team (P00C8)<br>• Education and resources for staff (P00C8)	• Location<br>○ In person<br>○ Virtual<br>• Educate healthcare staff about MAiD process, patient referral process, conscientious objection<br>○ Unit leaders (P00C8)<br>○ Educators (P00C8)
Ontario - *Grand River Hospital*	• Location<br>○ Online (P00C50)<br>• Orientation and onboarding (P00C50)<br>○ MAiD coordination team (P00C50)<br>• Education and resources for staff (P00C50)	• Location<br>○ In person (P00C50)<br>○ Virtual (P00C50)<br>• Educate healthcare staff about MAiD process, patient referral process (P00C50)
COMMUNITY OF PRACTICE		
Ontario - *Hamilton Family Health Team*	• Training for other healthcare professionals not specified	• Education for other healthcare professionals not specified
Ontario - *Niagara Community MAiD Team, St. Catharine's, Niagara*	• Training for other healthcare professionals not specified	• Location<br>○ In person<br>• Provide presentation at grand rounds (P00C11)

Jurisdiction	What types of training does the MAiD program offer?	What kind of educational sessions does the MAiD program offer?
Québec - *Ministry of Health and Social Services*	• Training for other healthcare professionals not specified	• Education for other healthcare professionals not specified
INTEGRATED HEALTH AND SOCIAL SERVICES CENTRES		
Québec – *CISS Montérégie*	• Training for other healthcare professionals not specified	• Education for other healthcare professionals not specified
INTEGRATED UNIVERSITY HEALTH AND SOCIAL SERVICES CENTRES		
Québec – *CIUSS Capitale-Nationale*	• Training for other healthcare professionals not specified	• Education for other healthcare professionals not specified
Québec – *University of Montreal Hospital Center*	• Training for other healthcare professionals not specified	• Education for other healthcare professionals not specified
Québec – *McGill University Health Centre*	• Training for other healthcare professionals not specified	• Education for other healthcare professionals not specified

Table 22: Public education and patient/family feedback

Jurisdiction	What proactive (initiated by MAiD program) strategies does the MAiD program utilize?	What reactive (in response to a request) strategies does the MAiD program utilize?	Does the MAiD program collect feedback?
Alberta - *Alberta Health Services*	Access to MAiD information<br>• Provincial MAiD Lead (P00A5)	Educational sessions<br>• One-on-one sessions<br>○ Patients and families (P00B13, P00B9)<br>• Group sessions<br>○ Community<br>▪ Individuals/organizations involved<br>- Dying with Dignity (P00C39)<br>- Alzheimer society (P00C39)<br>- Primary Care Networks (P00C39)<br>○ Care facilities<br>▪ Location<br>- Hospitals (P00B13, P00B9)<br>- Long-term care facilities (P00B9, P00B13)<br>- Continuing care facilities (P00B9, P00B13)<br>○ Religious institutions<br>▪ Location<br>- Churches (P00C39)<br>Access to MAiD information<br>• Online resources<br>○ Alberta Health Services website (P00B13)<br>○ Email (26) (P00A5)<br>○ Educational resources<br>▪ Pamphlet (detailing MAiD process) (4)<br>▪ Support guide (for patients and families) (3)<br>• Healthcare providers (P00B13, P00C10, P00B9)<br>• Phone calls (26) (P00B9, P00B13, P00C39)<br>○ Health link (811) (P00A5)<br>• News (P00B13)<br>• Advocacy organizations<br>○ Dying with Dignity (P00B13, P00C39, P00C6)	• Feedback collection not specified<br>○ Health authority collects feedback to rate experiences of services received (176)
British Columbia - *Ministry of Health*	• Proactive outreach strategies not specified	Access to MAiD information<br>• Online resources<br>○ Provincial website (27) (P00A4)<br>○ Email (P00A11)<br>• Healthcare providers (P00A4)	• Not applicable<br>○ Not involved in service delivery
British Columbia - *Fraser Health*	• Proactive outreach strategies not specified	Educational sessions<br>• Group sessions<br>○ Community<br>▪ Individuals/organizations involved<br>- Hospice societies (P00A10)<br>- Pension groups (P00A10)<br>▪ Location<br>- Community centers (P00A10)<br>- Library (P00A10)<br>Access to MAiD information<br>• Online resources<br>○ Regional website (P00A4)<br>○ Email (6) (P00A10)<br>○ Educational resources<br>▪ Pamphlet (detailing MAiD process) (28)<br>▪ Fact sheet (29)<br>▪ Support guide (for patients and families) (30, 106, 177)	• Yes (6)<br>○ Optional experience survey (6)<br>▪ Patient survey to rate experience of MAiD services received (6)<br>▪ Family and caregiver survey to rate experience of MAiD services received (6)

Jurisdiction	What proactive (initiated by MAiD program) strategies does the MAiD program utilize?	What reactive (in response to a request) strategies does the MAiD program utilize?	Does the MAiD program collect feedback?
		• Phone calls (6)<br>• Advocacy organizations<br>○ Dying with Dignity (P00A10)	
British Columbia - *Interior Health*	• Proactive outreach strategies not specified	Educational sessions<br>• Group sessions<br>○ Community<br>▪ Location<br>- Senior centers (P00C29)<br>Access to MAiD information<br>• Online resources<br>○ Regional website (P00A4)<br>○ Email (32) (P00A11)<br>○ Educational resources<br>▪ Pamphlet (detailing MAiD process) (34)<br>▪ Support guide (for patients and families) (33)<br>• Healthcare providers (P00C41)<br>• Phone calls (32) (P00C41)	• No<br>○ Health authority collects feedback to rate experience of services received (178)<br>○ Current plans to develop feedback surveys for MAiD services received (P00B14)
British Columbia - *Island Health*	Access to MAiD information<br>• Medical Lead (P00A13)<br>• Program Lead (P00A13)	Educational sessions<br>• Group sessions<br>○ Community<br>▪ Individuals/organizations involved<br>- Community groups (P00A9)<br>- Senior groups (P00A9)<br>○ Educational institutes<br>▪ Location<br>- Allied Health Institutes (P00A9)<br>- Nursing colleges (P00A9)<br>Access to MAiD information<br>• Online resources<br>○ Regional website (P00A9)<br>○ Email (35) (P00A9)<br>○ Educational resources<br>▪ Pamphlet (detailing MAiD process) (36) (P00A9)<br>▪ Support guide (for patients and families) (107) (P00A9)<br>▪ Instruction booklet (P00A9)<br>• Healthcare providers (P00A9)<br>• Phone calls (35) (P00A9)	• Feedback collection not specified<br>○ Health authority collects feedback to rate experience of services received (179)
British Columbia - *Northern Health*	• Proactive outreach strategies not specified	Access to MAiD information<br>• Online resources<br>○ Regional website (P00A4)<br>○ Provincial government website (P00A4) (37)<br>○ Email (37) (P00A11)<br>• Phone calls (37)	• Feedback collection not specified<br>○ Health authority collects feedback to rate experience of services received (180)
British Columbia - *Vancouver Coastal Health*	• Proactive outreach strategies not specified	Educational sessions<br>• Group sessions<br>○ Community (P00A7)<br>▪ Individuals/organizations involved<br>- Private organizations (P00B10)<br>- Indigenous communities (Indigenous-led sessions) (P00B10)<br>○ Care facilities<br>- Long-term care facilities (P00B10)<br>○ Educational institutions<br>▪ Location<br>- Universities (P00B10)	• Yes (181) (P00B10)<br>○ Optional experience survey (181)<br>▪ Patients' families rate experience of MAiD service received<br>○ Interview with family members (P00B10)

Jurisdiction	What proactive (initiated by MAiD program) strategies does the MAiD program utilize?	What reactive (in response to a request) strategies does the MAiD program utilize?	Does the MAiD program collect feedback?
		- Colleges (P00B10)<br>○ Religious institutions<br>▪ Location<br>- Churches (P00B10)<br>Access to MAiD information<br>• Online resources<br>○ Regional website (P00B9)<br>○ Email (38)<br>○ Educational resources<br>▪ Online courses (P00B15)<br>▪ Pamphlet detailing MAiD process (39)<br>▪ MAiD support guide patients and families (40)<br>• Healthcare providers (P00A7)<br>• Phone calls (P00B10)<br>• News (P00A7)	
British Columbia - *Provincial Health Services Authority*	• Proactive outreach strategies not specified	Access to MAiD information<br>• Online resources<br>○ Provincial website (P00A11) (41)<br>○ Email (41)<br>• Phone calls (P00A11) (41)	• No (P00A11)<br>○ Health authority collects feedback to rate experience of services received (182)
Manitoba - *Shared Health*	• Proactive outreach strategies not specified	Educational sessions<br>• Group sessions<br>○ Community<br>▪ Individuals/organizations involved<br>- Senior's legion (P00B21)<br>- Alzheimer's Society of Manitoba Board (P00C34)<br>○ Care facilities<br>▪ Location<br>- Hospitals (P00C33)<br>- Long-term care facilities (P00C33)<br>- Nursing homes (129)<br>▪ Individuals involved<br>- Patients and families (P00C34)<br>○ Educational institutions<br>▪ Location<br>- University (P00B21)<br>Access to MAiD information<br>• Online resources<br>▪ Provincial website (183) (P00C34, P00B21)<br>○ Educational resources<br>▪ Online courses (for healthcare providers) (P00C34)<br>▪ Pamphlet detailing MAiD process (43)<br>• Healthcare providers (P00C34, P00B21)<br>• Phone calls (P00C34) (7)<br>• News (P00C34)	• Yes (P00B21)<br>○ Phone calls with family members<br>▪ Social worker follows up after MAiD provision to get patient's family experience
New Brunswick - *Horizon Health Network*	• Proactive outreach strategies not specified	Educational sessions<br>• Group sessions<br>○ Community<br>▪ Location<br>- Death cafe (P00C15)<br>Access to MAiD information<br>• Online resources<br>○ Regional website (45) (P00B11)<br>○ Email (45) (P00B11)	• Feedback collection not specified<br>○ Health authority collects feedback to rate experience of services received (184)

Jurisdiction	What proactive (initiated by MAiD program) strategies does the MAiD program utilize?	What reactive (in response to a request) strategies does the MAiD program utilize?	Does the MAiD program collect feedback?
		○ Educational resources<br>▪ Online courses (for healthcare providers) (P00B11)<br>▪ Pamphlet detailing MAiD process (9)<br>• Healthcare providers (P00C15)<br>• Phone calls (P00B11)	
New Brunswick - *Vitalité Health Network*	• None (P00C48)	Access to MAiD information<br>• Online resources<br>○ Regional website (46)<br>○ Email (46)<br>○ Educational resources<br>▪ Pamphlet detailing MAiD process (47)<br>• Healthcare providers (P00C48)	• Feedback collection not specified (P00C48)<br>○ Health authority collects feedback to rate experience of services received (185)
Newfoundland and Labrador - *Department of Health and Community Services*	• Proactive outreach strategies not specified	Access to MAiD information<br>• Online resources<br>○ Educational resources<br>▪ MAiD FAQ document (10)	• Not applicable<br>○ Not involved in service delivery
Newfoundland and Labrador - *Eastern Zone*	• Proactive outreach strategies not specified	Access to MAiD information<br>• Online resources<br>○ Website (48)<br>○ Email (48)<br>○ Educational resources<br>▪ MAiD FAQ document (10) (P00C43)<br>• Phone calls (48)	• Feedback collection not specified<br>○ Health authority collects feedback to rate experience of services received (186, 187)
Newfoundland and Labrador - *Western Zone*	• Proactive outreach strategies not specified	Access to MAiD information<br>• Online resources<br>○ Email (P00C44)<br>○ Educational resources<br>▪ MAiD FAQ document (10)<br>• Healthcare providers (P00C44)<br>• Phone calls (P00C44)	• Feedback collection not specified<br>○ Health authority collects feedback to rate experience of services received (187, 188)
Newfoundland and Labrador - *Central Zone*	• Proactive outreach strategies not specified	Access to MAiD information<br>• Online resources<br>○ Website (189) (P00B22)<br>○ Educational resources<br>▪ MAiD FAQ document (10)<br>• Healthcare providers (P00B22)	• Feedback collection not specified<br>○ Health authority collects feedback to rate experience of services received (187)
Northwest Territories	Educational sessions<br>• Connect with NGOs partners and provide education sessions	Educational sessions<br>• Group sessions<br>○ Care facilities<br>▪ Location<br>- Home care facilities (for home care staff) (P00A2)<br>- Online (multiple sessions to different health care providers and Allied Health groups) (P00A3)<br>- Aurora college (nursing students) (P00A3)<br>Access to MAiD information<br>• Online resources<br>○ Provincial website (190)<br>○ Email (190)<br>○ Online resources<br>▪ Pamphlet detailing MAiD process (49) (P00A3)<br>• Healthcare provider (P00A3)<br>• Phone calls (P00C35)	• Yes (P00A2)<br>○ Follow up with family members

Jurisdiction	What proactive (initiated by MAiD program) strategies does the MAiD program utilize?	What reactive (in response to a request) strategies does the MAiD program utilize?	Does the MAiD program collect feedback?
Nova Scotia - *Nova Scotia Health*	• Proactive outreach strategies not specified	Educational sessions<br>• Group sessions<br>○ Community<br>▪ Individuals/organizations involved<br>- Community groups (P00A6, P00A12)<br>○ Care facilities<br>▪ Location<br>- Hospitals (P00A6, P00A12)<br>Access to MAiD information<br>• Online resources<br>○ Provincial website (51) (P00A12)<br>○ Email (51) (P00A12)<br>○ Educational resources<br>▪ MAiD patient and family guide (52) (P00A12)<br>• Healthcare provider (P00A12)<br>• Phone calls (51) (P00A12)	• Yes (P00A12)<br>○ Follow up with family members (P00A12)<br>○ Documents experiences of family members (P00A12)<br>○ Collect feedback from family members (P00A12)
Prince Edward Island - *Health PEI*	Educational Sessions<br>• Group education and training sessions for general and specific population (P00B17)	Educational sessions<br>• Group sessions<br>○ Community<br>▪ Location<br>- Women's Institute (P00B17)<br>- Residency schools (medical students/residents) (P00B8)<br>- Nursing schools (nursing students) (P00B8)<br>○ Care facilities<br>▪ Individuals involved<br>- Patients and families (P00B17)<br>▪ Location<br>- Hospital (grand rounds) (P00B17)<br>Access to MAiD information<br>• Online resources<br>○ Provincial website (P00B17)<br>○ Email (53)<br>○ Educational resources<br>▪ MAiD patient and family guide (54)<br>▪ MAiD information booklet (P00B8)<br>• Healthcare providers (P00B17)<br>• News (P00B8)	• Yes (P00B8)<br>○ Follow up with families (P00B8)
Saskatchewan - *Saskatchewan Health Authority*	• Proactive outreach strategies not specified	Educational sessions<br>• One-on-one sessions<br>○ Patients and families (P00A1, P00B1)<br>• Group sessions<br>○ Community<br>▪ Individuals/organizations involved<br>- Estate planners (P00A1)<br>- Lawyers (P00C1)<br>- Community pharmacist (P00B5)<br>- Assisted living facilities (P00A1)<br>- Disability services (P00C1)<br>▪ Location<br>- Funeral homes (P00A1)<br>○ Care facilities<br>▪ Location<br>- Nursing homes (P00C1)<br>○ Educational institutions<br>▪ Location	• Yes (P00B1)<br>○ Patients and families call or email MAiD program to provide feedback (P00B1)

Jurisdiction	What proactive (initiated by MAiD program) strategies does the MAiD program utilize?	What reactive (in response to a request) strategies does the MAiD program utilize?	Does the MAiD program collect feedback?
		- Universities (P00A1)<br>Access to MAiD information<br>• Online resources<br>○ Provincial website (55) (P00A1, P00B7)<br>○ Educational materials<br>▪ MAiD support guide (for patients and families) (57)<br>▪ Pamphlet (detailing MAiD grief and bereavement resources) (56)<br>• Healthcare providers (P00B1)<br>• Phone calls (55) (P00A1)	
Yukon - *Department of Health and Social Services*	• Proactive outreach strategies not specified	Educational sessions<br>• Group sessions<br>○ Community<br>▪ Location<br>- Radio station (P00C7)<br>○ Religious institutions<br>▪ Location<br>- Churches (P00C7)<br>Access to MAiD information<br>• Online resources<br>○ Provincial website (15) (P00C7)<br>• Healthcare providers (P00C7)	• Feedback collection not specified<br>○ Ministry collects feedback to rate experience of services received (191)
Ontario - *Ministry of Health*	• None (P00C16)	Ministry of Health<br>Access to MAiD information<br>• Online resources<br>○ Provincial website (59)<br>○ Educational materials (P00A4)<br>○ MAiD support (for patients and families) (192)<br>○ Healthcare providers (P00A4)<br>Care coordination services<br>Educational sessions<br>• Group sessions<br>○ Community<br>▪ Individuals/organizations involved<br>- Parkinson's group (P00C16)<br>Access to MAiD information<br>• Healthcare providers (P00C16)<br>• Phone calls (59)<br>• News (P00C16)<br>• Advocacy organizations<br>○ Dying with Dignity (P00C16)	• Feedback collection not specified<br>○ Health authority collects feedback to rate experience of services received (193)
SERVICE ORGANIZATIONS/REGIONAL FACILITIES			
Ontario - *Home and Community Care Support Services, Central East*	• None (P00C2)	Access to MAiD information<br>• Online resources<br>○ Email (61)<br>• Healthcare providers (P00C2)<br>• Phone calls (61) (P00C2)<br>• News (P00C2)	• No (P00C2)<br>○ Health authority collects feedback to rate experience of services received (193)
Ontario - *Home and Community Care Support Services, Waterloo Region*	• Proactive outreach strategies not specified	Educational sessions<br>• Group sessions<br>○ Community<br>▪ Individuals/organizations involved<br>- Community groups (P00C4)<br>○ Care facilities	• Yes<br>○ Patients and families can share their feedback with their care coordinator (62)

Jurisdiction	What proactive (initiated by MAiD program) strategies does the MAiD program utilize?	What reactive (in response to a request) strategies does the MAiD program utilize?	Does the MAiD program collect feedback?
		▪ Location<br>- Nursing homes (P00C4)<br>- Retirement homes (P00C4)<br>Access to MAiD information<br>• Online resources<br>○ Email (62)<br>• Phone call (62)<br>• News (P00C4)	
Ontario - *Home and Community Care Support Services, South East*	• Proactive outreach strategies not specified	Educational sessions<br>• Group sessions<br>○ Educational institutions<br>▪ Location<br>- University (P00C20)<br>Access to MAiD information<br>• Online resources<br>○ Website (P00C20)<br>○ Email (P00C20)<br>• Healthcare providers (P00C20)<br>○ Home care staff (P00C20)<br>○ MAiD care coordination staff (P00C20)<br>• Phone calls (63) (P00C20)<br>• News (P00C20)	• Yes<br>○ Patients and families can share their feedback with their care coordinator (63)
Ontario - *Home and Community Care Support Services, South West*	• Proactive outreach strategies not specified	Educational sessions<br>• Group sessions<br>○ Community<br>▪ Location<br>- Community events (P0023)<br>Access to MAiD information<br>• Online resources<br>○ Email (64)<br>• Healthcare providers (P0023)<br>• Phone calls (64) (P0023)	• Yes<br>○ Patients and families can share their feedback with their care coordinator (64)
Ontario - *Champlain Regional MAiD Network, the Ottawa Hospital*	• None (P00C5)	Educational sessions<br>• Group sessions<br>○ Care facilities<br>▪ Location<br>- Hospitals (P00C5)<br>○ Educational institutions<br>▪ Location<br>- Nursing institutes (P00C5)<br>Access to MAiD information<br>• Phone calls (66)	• Feedback collection not specified<br>○ Healthcare facility collects feedback to rate experience of services received (194)
HEALTHCARE FACILITIES			
Ontario - *Peterborough Regional Health Centre*	• Proactive outreach strategies not specified	Educational sessions<br>• Group sessions<br>○ Educational institutions<br>▪ Location<br>- University (P00C3)<br>Access to MAiD information<br>• Online resources<br>○ Email (62)<br>○ Educational resources<br>▪ Brochure (P00C3)<br>• Healthcare providers (palliative care physicians) (P00C3)	• Feedback collection not specified<br>○ Healthcare facility collects feedback to rate experience of services received (195)

Jurisdiction	What proactive (initiated by MAiD program) strategies does the MAiD program utilize?	What reactive (in response to a request) strategies does the MAiD program utilize?	Does the MAiD program collect feedback?
		• Phone calls (62)<br>• Printed resources	
Ontario - *Mount Sinai Healthcare Facility, Toronto*	• Proactive outreach strategies not specified	Access to MAiD information<br>• Online resources<br>○ Email (68)<br>• News (P00C36)	• Feedback collection not specified<br>○ Healthcare facility collects feedback to rate experience of services received (196)
Ontario - *University Health Network*	• None (P00C8)	Educational sessions<br>• One-on-one sessions<br>○ Patients and families (P00C8)<br>Access to MAiD information<br>• Online resources<br>○ Institutional website (69) (P00C8)<br>○ Educational resources<br>▪ Online courses (P00C8)<br>• Healthcare providers (69) (P00C8)	• Feedback collection not specified<br>○ Healthcare facility collects feedback to rate experience of services received (P00C8)
Ontario - *Grand River Hospital*	• None (P00C50)	Educational sessions<br>• One-on-one sessions<br>○ Patients and families (P00C50)<br>• Group sessions<br>○ Care facilities (P00C50)<br>Access to MAiD information<br>• Online resources (P00C50)<br>○ Educational resources<br>▪ Online courses (P00C50)	• No (P00C50)<br>○ Healthcare facility collects feedback to rate experience of services received (197)
COMMUNITY OF PRACTICE			
Ontario - *Hamilton Family Health Team*	• Proactive outreach strategies not specified	Educational sessions<br>• Group sessions<br>○ Care facilities<br>▪ Location<br>- Long-term care homes (P00B18)<br>- Community healthcare centers (P00B18)<br>○ Religious institutions<br>▪ Location<br>- Churches (P00B18)<br>Access to MAiD information<br>• Online resources<br>○ Website (P00B18)<br>○ Dying with Dignity's website (P00B18)<br>○ Email (70) (P00B18)<br>• Phone calls (70)<br>• News (P00B18)	• Feedback collection not specified<br>○ Organization collects feedback to rate experience of services received (198)
Ontario - *Niagara Community MAiD Team, St. Catharine's, Niagara*	• Proactive outreach strategies not specified	Educational sessions<br>• Group sessions<br>○ Community<br>▪ Individuals/organizations involved<br>- Community groups (P00C11)<br>- Support group (Pulmonary Fibrosis support group) (P00C11)<br>Access to MAiD information<br>• Healthcare providers (P00C11)<br>• Online resources<br>○ Email (70)	• Feedback collection not specified

Jurisdiction	What proactive (initiated by MAiD program) strategies does the MAiD program utilize?	What reactive (in response to a request) strategies does the MAiD program utilize?	Does the MAiD program collect feedback?
		• Phone calls (70) (P00C11)<br>• Advocacy organizations<br>○ Dying with Dignity (P00C11)	
GEOGRAPHICAL AREAS			
Ontario - *Oakville & Mississauga area*	• Not applicable<br>○ Not associated with MAiD program (P00C18)	Access to MAiD information<br>• Online resources<br>○ Email (70)<br>• Phone calls (70)	• Not applicable<br>○ Not associated with MAiD program (P00C18)
Ontario - *Oakville, Mississauga & Burlington area*	• Not applicable<br>○ Not associated with MAiD program (P00C21)	Access to MAiD information<br>• Phone calls (P00C21)	• Not applicable<br>○ Not associated with MAiD program (P00C21)
Ontario - *Renfrew County*	• Not applicable<br>○ Not associated with MAiD program (P00C22)	Educational sessions<br>• Group sessions<br>○ Community<br>▪ Individuals/organizations involved<br>- Seniors (P00C22)<br>Access to MAiD information<br>• Online resources<br>○ Educational resources<br>▪ Dying with Dignity booklet (P00C22)<br>• Phone calls (66) (P00C22)	• Not associated with MAiD program (P00C22)<br>○ No feedback collected (P00C22)
Ontario - *Greater Toronto Area*	• Not applicable<br>○ Not associated with MAiD program (P00C24)	Access to MAiD information<br>• Online resources<br>○ MAiDHouse website (P00C24)<br>○ Email (P00C24)<br>• Healthcare providers (P00C24)<br>• Phone calls (P00C24)	• Not applicable<br>○ Not associated with MAiD program (P00C24)
Ontario - *Waterloo area*	• Not applicable<br>○ Not associated with MAiD program (P00C25)	Educational sessions<br>• Group sessions<br>○ Community<br>▪ Individuals/organizations involved<br>- Organizations for seniors (P00C25)<br>○ Care facilities<br>▪ Location<br>- Hospitals (for healthcare providers and specialist teams)<br>- Hospices (P00C25)<br>○ Educational institutions<br>▪ Location<br>- High schools (P00C25)<br>○ Religious institutions<br>▪ Location<br>- Churches (P00C25)<br>Access to MAiD information<br>• Online resource<br>○ Email (62)<br>• Phone calls (62)	• Not applicable<br>○ Not associated with MAiD program (P00C25)
Ontario - *Noelville, Sudbury, Elliot Lake, Sturgeon Falls area*	• Not applicable<br>○ Not associated with MAiD program (P00C26)	Access to MAiD information<br>• Phone calls (199)<br>• Advocacy organizations<br>○ Dying with Dignity (P00C26)<br>○ Bridge C-14 (P00C26)	• Not applicable<br>○ Not associated with MAiD program (P00C26)

Jurisdiction	What proactive (initiated by MAiD program) strategies does the MAiD program utilize?	What reactive (in response to a request) strategies does the MAiD program utilize?	Does the MAiD program collect feedback?
Québec - *Ministry of Health and Social Services*	• Proactive outreach strategies not specified	Access to MAiD information<br>• Online resources<br>○ Website (127)<br>○ Email (127)<br>• Healthcare providers (127)	• Not applicable<br>○ Not involved in service delivery
INTEGRATED HEALTH AND SOCIAL SERVICES CENTRES			
Québec – *CISS Montérégie*	• Proactive outreach strategies not specified	Access to MAiD information<br>• Online resources<br>○ Website (72)<br>○ Email (111)<br>○ Educational resources<br>▪ Brochure (111)<br>• Healthcare providers (P0027)<br>• Phone calls<br>○ Health link (811) (P0027)	• Feedback collection not specified<br>○ Health authority collects feedback to rate experiences of services received (72)
INTEGRATED UNIVERSITY HEALTH AND SOCIAL SERVICES CENTRES			
Québec – *CIUSS Capitale-Nationale*	• Proactive outreach strategies not specified	Access to MAiD information<br>• Online resources<br>○ Website (73)<br>○ Email (73)<br>• Healthcare providers (P0037)<br>• Phone calls<br>○ Health link (811) (P0037)<br>• News (P00C37)	• Feedback collection not specified<br>○ Health authority collects feedback to rate experiences of services received (200)
Québec – *University of Montreal Hospital Center*	• Proactive outreach strategies not specified	Access to MAiD information<br>• Online resources<br>○ Website (74)<br>○ Email (74)<br>○ Educational resources<br>▪ Brochure (74)<br>• Healthcare providers (P00C9)<br>• Phone calls<br>○ Health link (811) (P00C9)	• Feedback collection not specified<br>○ Health institution collects feedback to rate experiences of services received (201)
Québec – *McGill University Health Centre*	• Proactive outreach strategies not specified	Access to MAiD information<br>• Online resources<br>○ Website (75)<br>○ Email (75)<br>○ Educational resources<br>▪ Policy (18)<br>▪ Brochure (P00C40)<br>• Healthcare providers (P00C40)<br>• Phone calls<br>○ Health link (811) (P0040)	• Feedback collection not specified<br>○ Health institution collects feedback to rate experiences of services received (202)

Reference List

1. Canadian Association of MAiD Assessors and Providers. The Interpretation and Role of "Reasonably Foreesable" in MAiD Practice. 2022. 10 p. Available from: https://camapcanada.ca/wp-content/uploads/2022/03/The-Interpretation-and-Role-of-22Reasonably-Foreseeable22-in-MAiD-Practice-Feb-2022.pdf. (accessed 8 Jan 2026).
2. Wu JSY, Pinilla J, Watson M, Verma S, Olivotto IA. Medical assistance in dying for cancer patients one year after legalization: a collaborative approach at a comprehensive cancer centre. Current Oncology (Toronto, Ont). 2018;25(5):e486-e9. DOI: https://doi.org/10.3747/co.25.4118
3. Alberta Health Services. Medical Assistance in Dying and Other End-Of-Life Options. 2022. 25 p. Available from: https://www.albertahealthservices.ca/assets/info/hp/maid/if-maid-and-other-end-of-life-options.pdf. (accessed 8 Jan 2026).
4. Alberta Health Services. Medical Assistance in Dying: How Do I Access Medical Assistance in Dying Services in Alberta? 2021. 1 p. Available from: https://www.albertahealthservices.ca/assets/info/maid/if-maid-how-access-medical-assistance-in-dying-in-alberta.pdf. (accessed 8 Jan 2026).
5. Alberta Health Services. Medical Assistance in Dying: Frequently Asked Questions for Patients and Family Members. 2016. Available from: https://www.albertahealthservices.ca/info/Page14382.aspx. (accessed 8 Jan 2026).
6. Fraser Health. Medical assistance in dying. 2023. Available from: https://www.fraserhealth.ca/health-topics-a-to-z/end-of-life-care/medical-assistance-in-dying. (accessed 8 Jan 2026).
7. Shared Health Manitoba. Contact MAiD. 2023. Available from: https://sharedhealthmb.ca/services/maid/contact-us/. (accessed 8 Jan 2026).
8. Manitoba College of Social Workers. Medical Assistance in Dying: Information Summary. 2022. Available from: https://mcsw.ca/wp-content/uploads/2024/12/MAiD-Information-Summary-Revised-June-2022.pdf. (accessed 31 Mar 2025).
9. Horizon Health Network. Patient Information: Medical Assistance in Dying. 2022. 10 p. Available from: https://horizonnb.ca/wp-content/uploads/2022/04/Maid-EN.pdf. (accessed 8 Jan 2026).
10. Government of Newfoundland and Labrador. Medical Assistance in Dying: Frequently Asked Questions. 2023. 9 p. Available from: https://www.gov.nl.ca/hcs/files/Medical-Assistance-in-Dying-MAiD.pdf. (accessed 8 Jan 2026).
11. Government of Northwest Territories. Medical Assistance in Dying - Guidelines for the Northwest Territories. 2025. 47. Available from: https://www.hss.gov.nt.ca/sites/hss/files/resources/maid-interim-guidelines.pdf. (accessed 8 Jan 2026).
12. Crumley ET, Kelly S, Young J, Phinney N, McCarthy J, Gubitz G. How is the medical assistance in dying (MAID) process carried out in Nova Scotia, Canada? A qualitative process model flowchart study. BMJ open. 2021;11(7):e048698.DOI: https://doi.org/10.1136/bmjopen-2021-048698
13. College of Physicians and Surgeons of Nova Scotia. Medical Assistance in Dying - Professional Standard Regarding Medical Assistance in Dying (MAiD). 2021. Available from: https://cpsns.ns.ca/resource/medical-assistance-in-dying/. (accessed 8 Jan 2026).
14. Nova Scotia College of Nursing. Practice Guideline for Nurses: Medical Assistance in Dying. 2025. 13 p. Available from: https://www.nscn.ca/sites/default/files/documents/resources/MAID_Practice_Guideline_for_LPNs_RNs.pdf. (accessed 8 Jan 2026).
15. Government of Yukon. Find Information About Medical Assistance in Dying. 2023. Available from: https://yukon.ca/en/health-and-wellness/find-information-about-medical-assistance-dying#how-to-start-the-process. (accessed 8 Jan 2026).
16. Centre intégré universitaire de Santé et des Services sociaux de la Capitale-Nationale. À L'Aide Médicale À Mourir de la Région De La Capitale-Nationale.2024. 1 p. Available from: https://www.ciusss-capitalenationale.gouv.qc.ca/sites/d8/files/docs/ProfSante/SPSFV/affiche%20promotionnel%20GIS.pdf. (accessed 8 Jan 2026).

17. Gouvernement du Québec. Act Respecting End-of-Life Care. 2023. Available from: https://www.quebec.ca/en/health/health-system-and-services/end-of-life-care/act-respecting-end-of-life-care. (accessed 8 Jan 2026).
18. McGill University Health Centre. End-of-Life Care Policy. 2025. 17 p. Available from: https://muhc.ca/sites/default/files/docs/End_Life_Care_Policy_En.pdf. (accessed 8 Jan 2026).
19. Canadian Association of MAiD Assessors and Providers. Intravenous MAiD Medication Protocols in Canada: Review and Recommendations. 2020. 41 p. Available from: https://camapcanada.ca/wp-content/uploads/2022/02/IV-protocol-final.pdf. (accessed 8 Jan 2026).
20. New Brunswick College of Pharmacists. Position Statement: Medical Assistance in Dying (MAiD). 2018. 4 p. Available from: https://nbpharmacists.ca/wp-content/uploads/2022/04/GM-PP-MAiD-01PositionStatementSept-242018EN_Oct22.pdf?x90867. (accessed 8 Jan 2026).
21. Newfoundland and Labrador Pharmacy Board. Newfoundland and Labrador Pharmacy Board Standards of Practice: Medical Assistance in Dying. 2023. p. 4. Available from: https://web.archive.org/web/20240505224245/https://nlpb.ca/media/SOPP-MAID-June2023.pdf. (accessed 8 Jan 2026).
22. The College of Physicians and Surgeons of Prince Edward Island. Medical Assistance In Dying (MAiD). 2024. 15 p. Available from: https://www.cpspei.ca/wp-content/uploads/2024/07/Medical-Assistance-in-Dying-Policy-MAiD-June-2024.pdf. (accessed 8 Jan 2026)
23. Prince Edward Island College of Pharmacy. Medical Assistance in Dying (MAiD): Guideline for Pharmacists and Pharmacy Technicians. 2016. 8 p. Available from: https://pepharmacists.ca/wp-content/uploads/2022/08/MAID-Guidance-Council-March-2022.pdf. (accessed 8 Jan 2026).
24. Ontario Ministry of Health. Medical Assistance in Dying and End-Of-Life Decisions Support For Medical Professionals. 2024. Available from: https://www.ontario.ca/page/medical-assistance-dying-and-end-life-decisions-support-medical-professionals. (accessed 8 Jan 2026).
25. Ontario College of Pharmacists. Medical Assistance in Dying: Guide for Pharmacists and Pharmacist Technicians. 2021. 9 p. Available from: https://www.ocpinfo.com/wp-content/uploads/2021/04/Medical-Assistance-in-Dying.pdf. (accessed 8 Jan 2026).
26. Alberta Health Services. Health Professional Information: Medical Assistance in Dying. 2023. Available from: https://www.albertahealthservices.ca/info/Page14381.aspx. (accessed 8 Jan 2026).
27. Government of British Columbia. Medical Assistance in Dying. 2024. Available from: https://www2.gov.bc.ca/gov/content/health/accessing-health-care/home-community-care/care-options-and-cost/end-of-life-care/medical-assistance-in-dying. (accessed 8 Jan 2026).
28. Fraser Health. Medical Assistance in Dying Brochure. 2021. 2 p. Available from: https://patienteduc.fraserhealth.ca/file/medical-assistance-in-dying-maid-brochure-203010.pdf. (accessed 8 Jan 2026).
29. Fraser Health. Medical Assistance in Dying. 2021. 2 p. Available from: https://patienteduc.fraserhealth.ca/file/medical-assistance-in-dying-maid-factsheet-407434.pdf. (accessed 8 Jan 2026).
30. Fraser Health. Medical Assistance in Dying - The Process. 2021. 1 p. Available from: https://patienteduc.fraserhealth.ca/file/medical-assistance-in-dying-maid-the-process-407435.pdf. (accessed 8 Jan 2026).
31. Fraser Health. Medical Assistance in Dying Policy. 2020. 5 p. Available from: https://www.fraserhealth.ca/-/media/Project/FraserHealth/FraserHealth/About-Us/Accountability/Policies/MAiDPolicy.pdf?rev=4d9ef47f298341d6ae71e351b8fc6493&hash=0BC35DEC3F17261F9AD23D2EDE308EFC664C20AD. (accessed 8 Jan 2026).
32. Interior Health. Medical Assistance in Dying. 2025. Available from: https://www.interiorhealth.ca/health-and-wellness/palliative-and-end-of-life-care/medical-assistance-in-dying#learning-more-about-medical-assistance-in-dying. (accessed 8 Jan 2026).
33. Interior Health. A Shared Journey: A Resource for Individuals, Families and Loved Ones About Medical Assistance in Dying. 2025. 12 p. Available from: https://www.interiorhealth.ca/sites/default/files/PDFS/821455-a-shared-journey-a-resource-for-individual-families-and-loved-ones-about-medical-assistance-in-dying.pdf. (accessed 8 Jan 2026).
34. Interior Health. Medical Assistance in Dying Brochure. 2023. 2 p. Available from: https://www.interiorhealth.ca/sites/default/files/PDFS/821318-patient-brochure.pdf. (accessed 8 Jan 2026).

35. Island Health. Medical Assistance in Dying (MAiD). 2023. Available from: https://www.islandhealth.ca/learn-about-health/medical-assistance-dying/medical-assistance-dying. (accessed 8 Jan 2026).
36. Island Health. Medical Assistance in Dying: Information for Island Health Patients and Families. 2023. 2 p. Available from: https://www.islandhealth.ca/sites/default/files/MAiD/documents/maid-public-handout.pdf. (accessed 8 Jan 2026).
37. Northern Health. Medical Assistance in Dying (MAiD). 2023. Available from: https://www.northernhealth.ca/health-topics/medical-assistance-dying-maid. (accessed 8 Jan 2026).
38. Vancouver Coastal Health. Medical Assistance in Dying (MAiD). 2026. Available from: https://www.vch.ca/en/service/medical-assistance-dying. (accessed 8 Jan 2026).
39. Vancouver Coastal Health. Medical Assistance In Dying (MAiD). 2025. Available from: https://vch.eduhealth.ca/en/viewer?file=%2fmedia%2fVCH%2fGV%2fGV.160.A88.pdf#search=GV.160.A88&phrase=false. (accessed 8 Jan 2026).
40. Vancouver Coastal Health. Guide for Medical Assistance in Dying (MAiD). 2025. 40 p. Available from: https://vch.eduhealth.ca/en/viewer?file=%2fmedia%2fVCH%2fGV%2fGV.160.G85.pdf - search=MAiD booklet&phrase=false. (accessed 8 Jan 2026).
41. Provincial Health Services Authority. Medical Assistance in Dying. 2026. Available from: http://www.phsa.ca/health-info/medical-assistance-in-dying. (accessed 8 Jan 2026).
42. Shared Health Manitoba. Process When Natural Death is Reasonably Foreseeable. 2026. Available from: https://sharedhealthmb.ca/services/maid/access/natural-death-is-reasonably-foreseeable/. (accessed 8 Jan 2026).
43. Shared Health Manitoba. Medical Assistance in Dying (MAiD) in Manitoba. 2024. 1 p. Available from: https://sharedhealthmb.ca/files/maid-information-pamphlet-for-patients.pdf. (accessed 8 Jan 2026).
44. Nursing College Practice Consultants. Medical Assistance in Dying: Guidelines for Manitoba Nurses. 2021. 22 p. Available from: https://www.crnm.mb.ca/wp-content/uploads/2022/01/MAID-guideline-July142021.pdf. (accessed 8 Jan 2026).
45. Horizon Health Network. Medical Assistance in Dying (MAID). 2026. Available from: https://horizonnb.ca/patients-visitors/patient-information-resources/medical-assistance-in-dying-maid/. (accessed 8 Jan 2026).
46. Vitalité Health Network. Medical Assistance in Dying. 2024. Available from: https://vitalitenb.ca/en/patients-and-visitors/patients/medical-assistance-in-dying. (accessed 8 Jan 2026).
47. Vitalité Health Network HHN. Patient Information: Medical Assistance in Dying. 2022. 8 p. Available from: https://vitalitenb.ca/images/files/documents/vitalite_infomaid.pdf. (accessed 8 Jan 2026).
48. Eastern Health. Medical Assistance in Dying (MAiD). 2023. Available from: https://peolc.easternhealth.ca/ourservices/maid/. (accessed 8 Jan 2026).
49. Government of Northwest Territories. Medical Assistance in Dying: Information for Patients and Families. 2024. 10 p. Available from: https://www.hss.gov.nt.ca/sites/hss/files/resources/maid-information-patients-families_0.pdf. (accessed 8 Jan 2026).
50. Nova Scotia Health Authority. Procedure: Medical Assistance in Dying. 2022. 15 p. Available from: https://policy.nshealth.ca/Site_Published/nsha/document_render.aspx?documentRender.IdType=6&documentRender.GenericField=&documentRender.Id=96720. (accessed 8 Jan 2026).
51. Nova Scotia Health. Medical Assistance in Dying (MAiD). 2026. Available from: https://www.nshealth.ca/maid. (accessed 8 Jan 2026).
52. Nova Scotia Health Authority. Patient and Family Guide: Medical Assistance in Dying (MAiD).2021. 14 p. Available from: https://physicians.nshealth.ca/sites/default/files/2022-03/MAID%20Family%20and%20Paitient%20Guide.pdf. (accessed 8 Jan 2026).
53. Government of Prince Edward Island. Medical Assistance in Dying. 2025. Available from: https://www.princeedwardisland.ca/en/information/health-pei/medical-assistance-in-dying. (accessed 8 Jan 2026).

54. Health PEI. Medical Assistance in Dying: Patient and Family Guide. 2024. 24 p. Available from: https://www.princeedwardisland.ca/sites/default/files/publications/maid_patient_and_family_guide_pei_2024.pdf. (accessed 8 Jan 2026).
55. Saskatchewan Health Authority. Medical Assistance in Dying. 2023. Available from: https://www.saskhealthauthority.ca/your-health/conditions-diseases-services/medical-assistance-dying. (accessed 8 Jan 2026).
56. Saskatchewan Health Authority. Grief and Bereavement Resources. 2026. 2 p. Available from: https://www.saskhealthauthority.ca/sites/default/files/2024-01/CS-PIER-0147-Grief-and-Bereavement-Resources.pdf. (accessed 8 Jan 2026).
57. Saskatchewan Health Authority. Information for Patients and Families: Medical Assistance in Dying. 2026. 6 p. Available from: https://www.saskhealthauthority.ca/sites/default/files/2024-01/CS-PIER-0132-MAID-Info-Patients-Families-Booklet.pdf. (accessed 8 Jan 2026).
58. Saskatchewan Health Authority. Provincial Medical Assistance in Dying (MAiD) Program: A Resource for Facilities and Caregivers. 2022. 4 p. Available from: https://www.saskhealthauthority.ca/sites/default/files/2022-10/A-Resource-for-Facilities-and-Caregivers.pdf. (accessed 8 Jan 20268).
59. Ontario Ministry of Health. Medical Assistance in Dying and End-Of-Life Decisions. 2023. Available from: https://www.ontario.ca/page/medical-assistance-dying-and-end-life-decisions. (accessed 8 Jan 2026).
60. Ontario Ministry of Health. Medical Assistance in Dying: General Information. 2023. 6 p. Available from: https://files.ontario.ca/moh-maid-general-information-en-2023-07-19.pdf. (accessed 8 Jan 2026).
61. Home and Community Care Support Services. Central East: Information and Referral. Available from: https://healthcareathome.ca/region/central-east/. (accessed 31 Mar 2025).
62. Home and Community Care Support Services. Waterloo Wellington: Information and Referral 2024. Available from: https://healthcareathome.ca/region/waterloo-wellington/. (accessed 31 Mar 2025).
63. Home and Community Care Support Services. South East: Information and Referral. 2024. Available from: https://healthcareathome.ca/region/south-east/. (accessed 31 Mar 2025).
64. Home and Community Care Support Services. South West: Information and Referral. 2024. Available from: https://healthcareathome.ca/region/south-west/. (accessed 31 Mar 2025).
65. The Ottawa Hospital. Medical Assistance in Dying: Information for Patients and Loved Ones. 2026. Available from: https://www.ottawahospital.on.ca/en/clinical-services/deptpgrmcs/departments/medical-assistance-in-dying-maid/information-for-patients-and-loved-ones/. (accessed 8 Jan 2026).
66. Home and Community Care Support Services. Champlain: Information and Referral. 2024 Available from: https://healthcareathome.ca/region/champlain/. (accessed 31 Mar 2025).
67. Peterborough Regional Health Centre. Medical Assistance in Dying: FAQ for Patients and Families. 2024. 20 p. Available from: https://www.prhc.on.ca/wp-content/uploads/2022/10/MAID-Booklet-4809.pdf. (accessed 31 Mar 2025).
68. Home and Community Care Support Services. Toronto Central: Information and Referral. 2024. Available from: https://healthcareathome.ca/region/toronto-central/. (accessed 31 Mar 2025).
69. University Health Network. Medical Assistance In Dying (MAID). 2024. Available from: https://www.uhn.ca/PatientsFamilies/Patient_Services/MAiD_Patients. (accessed 31 Mar 2025).
70. Home and Community Care Support Services. Hamilton Niagara Haldimand Brant: Information and Referral. 2024. Available from: https://healthcareathome.ca/region/hamilton-niagara-haldimand-brant/. (accessed 31 Mar 2025).
71. Gouvernement du Québec. Requirements - Medical Aid in Dying. 2022. Available from: https://www.quebec.ca/en/health/health-system-and-services/end-of-life-care/medical-aid-in-dying/requirements. (accessed 31 Mar 2025).
72. Government of Québec. Complaints, Satisfaction and Mistreatment - CISSS de la Montérégie-Centre. 2026. Available from: https://www.santemonteregie.qc.ca/en/center/organisation/plaintes-et-amelioration-des-services. (accessed 12 Jan 2026).
73. Integrated University Health and Social Services Center of the Capitale-Nationale. Medical assistance in dying. 2024. Available from: https://www.ciusss-

capitalenationale.gouv.qc.ca/personnel-sante/soins-palliatifs-fin-vie/aide-medicale-mourir. (accessed 31 Mar 2025).
74. University of Montreal Hospital Center. Medical aid in dying: Making a Contemporaneous Request 2025. 2 p. Available from: https://www.chumontreal.qc.ca/sites/default/files/2021-07/498-2-medical-aid-in-dying.pdf. (accessed 31 Mar 2025).
75. McGill University Health Centre. End-of-life care at the McGill University Health Centre. 2025 Available from: https://muhc.ca/patients/end-life-care-mcgill-university-health-centre. (accessed 31 Mar 2025).
76. Alberta Health Services. Record of Request for Medical Assistance in Dying (MAiD). 5 p. 2023. Available from: https://www.albertahealthservices.ca/frm-22073.pdf. (accessed 12 Jan 2026).
77. Alberta Health Services. Medical Assistance in Dying (MAiD) Policy. 2016. Available from: https://extranet.ahsnet.ca/teams/policydocuments/1/clp-med-assist-in-death-hcs-165-01.pdf. (accessed 31 Mar 2025).
78. Province of British Columbia. Medical Assistance in Dying - Information for Health-Care Providers 2023. Available from: https://www2.gov.bc.ca/gov/content/health/accessing-health-care/home-community-care/care-options-and-cost/end-of-life-care/medical-assistance-in-dying/information-for-providers. (accessed 31 Mar 2025).
79. British Columbia College of Nurses and Midwives. Medical Assistance in Dying: Limits and Conditions. 2024. Available from: https://www.bccnm.ca/NP/ScopePractice/part2/maid/Pages/maid_limits_conditions.aspx. (accessed 31 Mar 2025).
80. British Columbia Ministry of Health. HLTH 1632 - Request for MAiD: Instructions for Completion-Medical Assistance in Dying. 2023. 7 p. Available from: https://www2.gov.bc.ca/assets/gov/health/forms/1632_instructions.pdf. (accessed 12 Jan 2026).
81. British Columbia Ministry of Health. Assessment Record - Instructions for Completion - Medical Assistance in Dying. 2023. 7 p. Available from: https://www2.gov.bc.ca/assets/gov/health/forms/1633_instructions.pdf. (accessed 12 Jan 2026).
82. Shared Health. Frequently Asked Questions. 2024. Available from: https://sharedhealthmb.ca/services/maid/faq/. (accessed 31 Mar 2025).
83. Vitalité Health Network. Patient Request: Medical Assistance in Dying (MAiD). 2022. 2 p. Available from: https://vitalitenb.ca/images/files/documents/patient_request-maid_rc-74e.pdf. (accessed 12 Jan 2026).
84. Western Health. Medical Assistance in Dying (MAiD) Patient Request and Consent Form. 2 p. 2024. [Available from: https://mdcme.ca/lms/courses/maid/forms/Western/3.%20MAID%20Patient%20Request%20and%20Consent%20Form%20-%20Initial%20Consent.pdf. (accessed 31 Mar 2025).
85. Government of Northwest Territories. Medical Assistance in Dying - Formal Written Request. 2023. 6 p. Available from: https://www.hss.gov.nt.ca/sites/hss/files/resources/formal-written-request-by-patient.pdf. (accessed 31 Mar 2025).
86. Nova Scotia Health Authority. NSH Medical Assistance in Dying (MAiD) Referral Form. 2021. 1 p. Available from: https://www.nshealth.ca/sites/default/files/2023-08/ns_maidrf.pdf. (accessed 12 Jan 2026).
87. Health PEI. Request for Medical Assistance in Dying. 2023. Available from: https://www.princeedwardisland.ca/sites/default/files/forms/medical_assistance_in_dying_patient_request_and_consent_form.pdf. (accessed 31 Mar 2025).
88. Saskatchewan Health Authority. Accessing MAiD in Saskatchewan. 2022. Available from: https://www.saskhealthauthority.ca/your-health/conditions-diseases-services/all-z/medical-assistance-dying/accessing-maid-saskatchewan. (accessed 31 Mar 2025).
89. Yukon Medical Council. Patient Request for Medical Assistance in Dying (MAiD). 2021. 2 p. Available from: https://yukon.ca/sites/yukon.ca/files/hss/hss-forms/patient_request_for_maid_05-21.pdf. (accessed 12 Jan 2026).
90. Government of Yukon. Find information about medical assistance in dying: What is the process? 2023. Available from: https://yukon.ca/en/health-and-wellness/find-information-about-medical-assistance-dying#what-is-the-process. (accessed 31 Mar 2025).

91. Government of Ontario. Medical Assistance in Dying and End-Of-Life Decisions. 2026. Available from: https://www.ontario.ca/page/medical-assistance-dying-and-end-life-decisions. (accessed 12 Jan 2026).
92. Government of British Columbia. Request for Medical Assistance in Dying. 2023. 3 p. Available from: https://www2.gov.bc.ca/assets/gov/health/forms/1632fil.pdf. (accessed 12 Jan 2026).
93. Shared Health Manitoba. Written Request Form. 2022.
94. Horizon Health Network. Medical Assistance in Dying (MAiD) Patient Request Form. 2021. 1 p. Available from: https://horizonnb.ca/wp-content/uploads/2021/12/Medical-Assistance-in-Dying-Patient-Request-Form.pdf. (accessed 12 Jan 2026).
95. Government of Newfoundland and Labrador. Medical Assistance in Dying (MAiD) Patient Request Record. 2021. 3 p. Available from: https://www.centralhealth.nl.ca/_files/ugd/d55165_94eb295ea9ad44ef9d05335f5330ee1d.pdf. (accessed 12 Jan 2026).
96. Nova Scotia Health. Medical Assistance in Dying (MAiD): Patient Request and Informed Consent Form - Reasonably Foreseeable Natural Death. 2023. 2 p. Available from: https://physicians.nshealth.ca/sites/default/files/2023-06/MAID%20Request%20and%20Consent%20Forms%20RFND.pdf. (accessed 12 Jan 2026).
97. Nova Scotia Health. Medical Assistance in Dying (MAiD) - Patient Request and Informed Consent Form - Non-Reasonably Foreseeable Natural Death. 2024. Available from: https://www.nshealth.ca/sites/default/files/2023-08/maid_request_and_consent_-_death_not_reasonably_foreseeable_-_23_july_2021.pdf. (accessed 31 Mar 2025).
98. Saskatchewan Health Authority. Patient Written Request. 2023.
99. Ontario Ministry of Health. Clinician Aid A: Patient Request for Medical Assistance in Dying. 2021 [Available from: https://forms.mgcs.gov.on.ca/en/dataset/014-3889-22. (accessed 31 Mar 2025).
100. Quebec Ministry of Health and Social Services. Request for Medical Assistance in Dying Form. 2025. Available from: https://web.archive.org/web/20240406011215/https://www.ciusss-capitalenationale.gouv.qc.ca/sites/d8/files/docs/ProfSante/SPSFV/AH-881_AMM_francais.pdf . (accessed 31 Mar 2025).
101. Vitalité Health Network. Official languages. 2024. Available from: https://vitalitenb.ca/en/health-network/about/official-languages. (accessed 31 Mar 2025).
102. Government of Canada. Medical Assistance in Dying - Glossary. 2016. Available from: https://www.justice.gc.ca/eng/cj-jp/ad-am/glos.html. (accessed 31 Mar 2025).
103. Commission on end-of-life Quebec. Conditions of Eligibility of Medical Aid in Dying in Quebec: Consistency In The Evolution of The Law Concerning End-Of-Life Care. 2025. Available from: https://csfv.gouv.qc.ca/fileadmin/docs/autres_rapports/csfv_lcsfv_conditions_amm_2023-06-29.pdf. (accessed 31 Mar 2025).
104. Government of Canada. Model Practice Standard for Medical Assistance in Dying (MAID) 2023. Available from: https://www.canada.ca/en/health-canada/services/publications/health-system-services/model-practice-standard-medical-assistance-dying.html#a28. (accessed 12 Jan 2026).
105. Government of Canada. Canada's Medical Assistance in Dying (MAID) Law. 2024. Available from: https://www.justice.gc.ca/eng/cj-jp/ad-am/bk-di.html#s2_2. (accessed 12 Jan 2026).
106. Fraser Health. Medical Assistance in Dying (MAiD) - A Guide to Support People Requesting MAiD. 2021. 4 p. Available from: https://patienteduc.fraserhealth.ca/file/medical-assistance-in-dying-maid-a-guide-to-suppo-460459.pdf. (accessed 12 Jan 2026).
107. Island Health. Medical Assistance in Dying: Support and Bereavement Guide. 2021. Available from: https://www.islandhealth.ca/sites/default/files/MAiD/documents/maid-brochure-bereavement-pt-families-april-2021.pdf. (accessed 31 Mar 2025).
108. College of Physicians and Surgeons of Saskatchewan. Medical Assistance in Dying Policy: Patient's Death is Not Reasonably Foreseeable. 2021. 11 p. Available from: https://uat.cps.sk.ca/iMIS/Documents/Legislation/Policies/POLICY - Medical Assistance in Dying - Patient%27s death is not reasonably foreseeable.pdf. (accessed 12 Jan 2026).
109. Yukon Medical Council. Standard of Practice: Medical Assistance in Dying. 2023. 13 p. Available from: https://www.yukonmedicalcouncil.ca/pdfs/MD.YMC_MAID_final_March_2023.pdf. (accessed 12 Jan 2026).

110. Centre intégré universitaire de Santé et des Services sociaux de la Capitale-Nationale. Aide Médicale à Mourir: Critères d'Admissibilité et Mesures de Sauvegarde. 2025. Available from: https://www.ciusss-capitalenationale.gouv.qc.ca/sites/d8/files/docs/ProfSante/SPSFV/Criteres-admissibilite-mesures-sauvegarde.pdf. (accessed 31 Mar 2025).
111. Centre intégré de santé et de services sociaux de la Montérégie-Ouest. Medical Assistance in Dying. 2025. [Available from: https://www.santemonteregie.qc.ca/sites/default/files/2023/01/brochure_aide_medicale_mourir_11_22_ang.pdf. (accessed 12 Jan 2026).
112. College of Physicians and Surgeons of British Columbia. Update to MAiD Provincial Reporting Forms. 2023. Available from: https://www.cpsbc.ca/news/publications/college-connector/2023-V11-01/02. (accessed 12 Jan 2026).
113. Nurses Association of New Brunswick. FAQ: Medical Assistance in Dying (MAiD). 2024. 7 p. Available from: https://nanb.nb.ca/media/documents/NANB-FAQ-MAID-Jul-21-Oct-24-E.pdf. (accessed 12 Jan 2026).
114. Eastern Health. First Practitioner (Primary) Opinion for Medical Assistance in Dying (MAiD) Part 1. 2017. Available from: https://mdcme.ca/lms/courses/maid/forms/Eastern/2.%20Ch-1711%20First%20Practitioner%20(Primary)%20Opinion%20for%20Medical%20Assistancein%20Dying%20(MAiD).pdf. (accessed 31 Mar 2025).
115. Western Health. Medical Assistance in Dying (MAiD) Assessment Form. 2024. Available from: https://mdcme.ca/lms/courses/maid/forms/Western/5.%20MAID%20Assessment%20Form.pdf. (accessed 12 Jan 2026).
116. Central Health. Medical Assistance in Dying: First Practitioner Assessment Record. 2024. Available from: https://mdcme.ca/lms/courses/maid/forms/Central/2.%20FRM-MED002%20Assessment%20Record%20(First%20Practitioner).pdf. (accessed 12 Jan 2026).
117. Government of Northwest Territories. Medical Assistance in Dying: Questions and Answers for Patients and Families. 2023. 8 p. Available from: https://www.hss.gov.nt.ca/sites/hss/files/resources/maid-qas-patients-families.pdf. (accessed 12 Jan 2026).
118. Nova Scotia Health Authority. Medical Assistance in Dying (MAiD) - Assessment Form. 2021. 2 p. Available from: https://physicians.nshealth.ca/sites/default/files/2023-06/maid_assessment_forms.pdf. (accessed 12 Jan 2026).
119. Government of Yukon. Find Information About Medical Assistance in Dying: Resources. 2023. Available from: https://yukon.ca/en/health-and-wellness/find-information-about-medical-assistance-dying#resources. (accessed 31 Mar 2025).
120. Government of Yukon. Secondary Assessor Record for Medical Assistance in Dying. 2024. 3 p. Available from: https://yukon.ca/sites/yukon.ca/files/hss/hss-forms/secondary_assessor_record_maid_05-21.pdf. (accessed 12 Jan 2026).
121. Yukon Medical Council. Most Responsible Medical Practitioner Record for Medical Assistance in Dying: Patients Safeguards and Administration. 2024. 7 p. Available from: https://yukon.ca/sites/yukon.ca/files/hss/hss-forms/most_responsible_practitioner_record_maid_safeguards_administration_05-21.pdf. (accessed 12 Jan 2026).
122. College of Physicians and Surgeons of British Columbia. Practice Standard: Medical Assistance in Dying. 2022. Available from: https://www.cpsbc.ca/files/pdf/PSG-Medical-Assistance-in-Dying.pdf. (accessed 31 Mar 2025).
123. Province of British Columbia. Medical Assistance in Dying. 2024. Available from: https://www2.gov.bc.ca/gov/content/health/accessing-health-care/home-community-care/care-options-and-cost/end-of-life-care/medical-assistance-in-dying. (accessed 12 Jan 2026).
124. Nurses Association of New Brunswick. Practice Guideline for Registered Nurses and Nurse Practitioners: Medical Assistance in Dying. 2023. 16 p. Available from: https://web.archive.org/web/20230402191102/https://www.nanb.nb.ca/wp-content/uploads/2022/08/NANB-PracticeGuidelines-RNsNPs-MAID-June21-E-1.pdf. (accessed 12 Jan 2026).

125. Nova Scotia Health Authority. Medical Assistance in Dying (MAiD): Patient and Family Guide. 2021. 14 p. Available from: https://physicians.nshealth.ca/sites/default/files/2022-03/MAID%20Family%20and%20Paitient%20Guide.pdf. (accessed 12 Jan 2026).
126. College of Physicians and Surgeons of Saskatchewan. Medical Assistance in Dying Policy: Patient's Death is Reasonably Foreseeable. 2021. 13 p. Available from: https://www.cps.sk.ca/iMIS/Documents/Legislation/Policies/POLICY%20-%20MAiD%20-%20Patient%E2%80%99s%20Death%20is%20Reasonably%20Foreseeable.pdf. (accessed 12 Jan 2026).
127. Gouvernement du Québec. Medical Aid in Dying. 2022. Available from: https://www.quebec.ca/en/health/health-system-and-services/end-of-life-care/medical-aid-in-dying/description. (accessed 31 Mar 2025).
128. College of Physicians and Surgeons of Manitoba. Standard of Practice: Medical Assistance in Dying (MAiD). 2024. 16 p. Available from: https://cpsm.mb.ca/assets/Standards%20of%20Practice/Standard%20of%20Practice%20Medical%20Assistance%20in%20Dying%20(MAID).pdf. (accessed 12 Jan 2026).
129. Manzhura A. Accessing Medical Assistance in Death (MAiD): An interpretative phenomenological analysis of experiences with MAiD by family members and providers in Manitoba 2021. 234 p. Available from: https://winnspace.uwinnipeg.ca/bitstream/handle/10680/1934/Manzhura_Aleksandra_Final_thesis_2021.pdf?sequence=3&isAllowed=y. (accessed 12 Jan 2026).
130. Vitalité Health Network. Guide for Professionals: Medical Assistance in Dying (MAID). 2024. 10 p. Available from: https://vitalitenb.ca/en/content-page/download/494/808/77?method=view. (accessed 12 Jan 2026).
131. Alberta Health Services. Consent to Treatment Plan or Procedure. 2023. Available from: https://www.albertahealthservices.ca/frm-09741.pdf. (accessed 31 Mar 2025).
132. Alberta Health Services. Role of the Medical Examiner Related to Medical Assistance in Dying 2018. 4 p. Available from: https://www.albertahealthservices.ca/assets/info/hp/maid/if-hp-maid-medical-examiner-role.pdf. (accessed 12 Jan 2026).
133. British Columbia College of Nurses and Midwives. Medical Assistance in Dying. 2026. Available from: https://www.bccnm.ca/NP/ScopePractice/part2/maid/Pages/Default.aspx. (accessed 12 Jan 2026).
134. British Columbia Ministry of Health. HLTH 1645 - Waiver of Final Consent - Instructions for Completion. 2022. 5 p. Available from: https://www2.gov.bc.ca/assets/gov/health/forms/1645_instructions.pdf. (accessed 12 Jan 2026).
135. Government of British Columbia. Medical Assistance in Dying - Information for Health-Care Providers. 2023. Available from: https://www2.gov.bc.ca/gov/content/health/accessing-health-care/home-community-care/care-options-and-cost/end-of-life-care/medical-assistance-in-dying/information-for-providers. (accessed 31 Mar 2025).
136. Government of British Columbia. Medical Assistance in Dying (MAiD) PINs. 2026. Available from: https://www2.gov.bc.ca/gov/content/health/practitioner-professional-resources/pharmacare/pharmacies/product-identification-numbers/maid-pins. (accessed 12 Jan 2026).
137. Province of British Columbia. Request for Medical Assistance in Dying. 2023. 3 p. Available from: https://www2.gov.bc.ca/assets/gov/health/forms/1632fil.pdf. (accessed 12 Jan 2026).
138. Brown J, Thorpe, L., Goodridge, D. Completion of Medical Certificates of Death After an Assisted Death: An Environmental Scan of Practices. Healthc Policy. 2018;14(2):59-67. DOI: https://doi.org/10.12927/hcpol.2018.25685
139. British Columbia Ministry of Health. HLTH 1634 - Assessment Record (Prescriber): Instructions for Completion - Medical Assistance in Dying. 2023. Available from: https://www2.gov.bc.ca/assets/gov/health/forms/1634_instructions.pdf. (accessed 31 Mar 2025).
140. Northern Health. Clinical Practice Standard: Medical Assistance in Dying. 2017 [Available from: https://physicians.northernhealth.ca/sites/physicians/files/physician-resources/maid/documents/nh-maid-policy.pdf. (accessed 12 Jan 2026).
141. Shared Health Manitoba. Frequently Asked Questions. 2026. Available from: https://sharedhealthmb.ca/services/maid/faq/. (accessed 12 Jan 2026).

142. Brown J, Thorpe, L., Goodridge, D. Completion of Medical Certificates of Death after an Assisted Death: An Environmental Scan of Practices. Healthc Policy. 2018;14(2):59-67. DOI: https://doi.org/10.12927/hcpol.2018.25685
143. Horizon Health Network. Prescription for Medical Assistance in Dying: Self Administered. 2021.
144. Vitalité Health Network. Medical Assistance in Dying: Waiver of Final Consent. 2021. 2 p. Available from: https://vitalitenb.ca/en/content-page/download/491/808/77?method=view. (accessed 12 Jan 2026).
145. Vitalité Health Network. Medical Assistance in Dying (MAiD) Procedure Checklist for Administration of Medications. 2020. 4 p. Available from: https://vitalitenb.ca/en/content-page/download/490/808/77?method=view. (accessed 12 Jan 2026).
146. College of Physicians and Surgeons of Newfoundland and Labrador. Standard of Practice: Medical Assistance in Dying. 2023. 1 p. Available from: https://cpsnl.ca/wp-content/uploads/Standards-and-Guidelines/Medical-Assistance-in-Dying.pdf. (accessed 12 Jan 2026).
147. College of Registered Nurses of Newfoundland and Labrador. Medical Assistance in Dying (MAiD). 2022. 22 p. Available from: https://crnnl.ca/site/uploads/2023/03/medical-assistance-in-dying.pdf. (accessed 12 Jan 2026).
148. Memorial University. MAiD in Newfoundland and Labrador: Provision of MAiD. 2024. Available from: https://mdcme.ca/lms/courses/maid/maidNL.asp. (accessed 12 Jan 2026).
149. Eastern Health Authority. Medical Assistance in Dying (MAiD) Waiver of Final Consent Agreement (Part I). 2024.Available from: https://mdcme.ca/lms/courses/maid/forms/Eastern/MAiD%20Waiver%20of%20Final%20Consent.pdf. (accessed 31 Mar 2025).
150. Eastern Health. Medication Administration Record: Medical Assistant in Dying Intravenous Drug Protocol. 2024. Available from: https://mdcme.ca/lms/courses/maid/forms/Eastern/6.%20Ch-1876%20Medication%20Administration%20Record%20(MAR)%20Medical%20Assistance%20in%20Dying%20(MAiD)%20Intravenous(IV)%20Drug%20Protocol%20fillable.pdf. (accessed 12 Jan 2026).
151. Western Health. Medical Assistance in Dying Intravenous Medication Protocol. 2024. 4 p. Available from: https://mdcme.ca/lms/courses/maid/forms/Western/7.MAiD%20Pharamcy%20order%20sheet%20-%20Pharmacy%20Revision%204.pdf. (accessed 12 Jan 2026).
152. Central Health. Medical Assistance in Dying Intravenous Protocol. 2024. Available from: https://mdcme.ca/lms/courses/maid/forms/Central/7.%20FRM-PHA017%20MAiD%20Standardized%20Prescription%20Protocols%20Form.pdf. (accessed 31 Mar 2025).
153. Government of Northwest Territories. Medical Assistance in Dying - Express Consent by Patients to Receive Medical Assistance in Dying. 2023. 3 p. Available from: https://www.hss.gov.nt.ca/professionals/sites/professionals/files/resources/express-consent-patient-receive-medical-assistance-dying.pdf.pdf. (accessed 12 Jan 2026).
154. Government of Northwest Territories. Interim Medication Protocols for the Northwest Territories. 2023. 27 p. Available from: https://www.hss.gov.nt.ca/professionals/sites/professionals/files/resources/maid-interim-medication-protocols.pdf. (accessed 12 Jan 2026).
155. Medical Assistance in Dying - Guidelines for the Northwest Territories: Government of Northwest Territories; 2025. 47 p. Available from: https://www.hss.gov.nt.ca/sites/hss/files/resources/maid-interim-guidelines.pdf. (accessed 12 Jan 2026).
156. Nova Scotia College of Pharmacy. Standards of Practice: Medical Assistance in Dying. 2021. 17 p. Available from: https://www.nspharmacists.ca/wp-content/uploads/2016/07/SOP_MedicalAssistanceInDying.pdf. (accessed 12 Jan 2026).
157. Saskatchewan Health Authority. Information for Patients and Families. 2023. 6 p. Available from: https://www.saskhealthauthority.ca/sites/default/files/2021-05/Information-for-Patients-and-Families.pdf. (accessed 12 Jan 2026).
158. Government of Yukon. Find Information About Medical Assistance in Dying: What's Medical Assistance in Dying? 2024. Available from: https://yukon.ca/en/health-and-wellness/find-

information-about-medical-assistance-dying#whats-medical-assistance-in-dying. (accessed 31 Mar 2025).
159. Ontario Ministry of Health. Updated - Frequently Asked Questions for Dispensers: Dispensing Publicly Funded Kits for Medical Assistance in Dying (MAiD) and Claims Submission using the Health Network System. 2022. Available from: https://files.ontario.ca/moh-questions-answers-executive-officer-notice-en-2022-04-01.pdf. (accessed 12 Jan 2026).
160. College of Physicians and Surgeons of Ontario. Medical Assistance in Dying. 2023. Available from: https://www.cpso.on.ca/Physicians/Policies-Guidance/Policies/Medical-Assistance-in-Dying#Policy. (accessed 12 Jan 2026).
161. Québec Ministry of Health and Social Services. Medical Assistance in Dying at End of Life Consent in The Event of Incapacity. 2025. Available from: https://www.ciusss-capitalenationale.gouv.qc.ca/sites/d8/files/docs/ProfSante/SPSFV/AMM_Form_Consent-event-incapacity_English.pdf. (accessed 31 Mar 2025).
162. Centre intégré universitaire de Santé et des Services sociaux de la Capitale-Nationale. Medical Assistance in Dying: Standardized Individual Order. 2025 [Available from: https://www.ciusss-capitalenationale.gouv.qc.ca/sites/d8/files/docs/ProfSante/SPSFV/CN00266-Aide-medicale-mourir-AMM-OIS.pdf. (accessed 31 Mar 2025).
163. Government of Québec. Act Respecting End-Of-Life Care. 2025. 28 p. Available from: https://www.legisquebec.gouv.qc.ca/en/pdf/cs/S-32.0001.pdf. (accessed 12 Jan 2026).
164. Mellett J, Macdonald ME. Medical assistance in dying in hospice: A qualitative study. BMJ supportive & palliative care. 2024(14):1497-502. DOI: https://doi.org/10.1136/bmjspcare-2021-003191?urlappend=%3Futm_source=researchgate.net%26utm_medium=article
165. Silvius JL, Memon A, Arain M. Medical Assistance in Dying: Alberta Approach and Policy Analysis. Canadian journal on aging = La revue canadienne du vieillissement. 2019;38(3):397-406.DOI: https://doi.org/10.1017/s0714980819000175
166. British Columbia Ministry of Health. Medical Assistance in Dying Travel and Training Assistance Program Policy. 2023. Available from: https://www2.gov.bc.ca/assets/gov/health/practitioner-pro/maidttap_policy.pdf. (accessed 31 Mar 2025).
167. Vancouver Coastal Health. Frequently Asked Questions for Medical Assistance in Dying (MAiD). 2024. Available from: https://www.vch.ca/en/frequently-asked-questions-medical-assistance-dying-maid. (accessed 12 Jan 2026).
168. The College of Physicians and Surgeons of Newfoundland and Labrador. Standard of Practice: Medical Assistance in Dying. 2023. 1 p. Available from: https://imis.cpsnl.ca/web/files/2017-Mar-11%20-%20MAID.pdf. (accessed 12 Jan 2025).
169. Nova Scotia Health Authority. Medical Assistance in Dying (MAiD) Policy. 2022. 10 p. Available from: https://policy.nshealth.ca/site_published/nsha/document_render.aspx?documentRender.IdType=6&documentRender.GenericField=&documentRender.Id=74151. (accessed 12 Jan 2026).
170. Doctors Nova Scotia. Travel/Redeployment Expenses. 2024. Available from: https://doctorsns.com/contract-and-support/physician-agreement/programs-funding/travel-redeployment. (accessed 12 Jan 2025).
171. College of Physicians and Surgeons of Saskatchewan. Medical Assistance in Dying Policy. 2018. 14 p. https://camapcanada.ca/wp-content/uploads/2022/03/POLICY20-20Medical20Assistance20in20Dying1.pdf. (accessed 12 Jan 2025).
172. Government of Saskatchewan. Billing Information Sheet: Physician Remuneration for the Provision of Legislated MAiD Services. 2024. Available from: https://www.ehealthsask.ca/services/resources/establish-operate-practice/Documents/MAID-Physician-Remuneration-Billing-Information-Sheet-%28October-2018%29.pdf. (accessed 31 Mar 2025).
173. Government of Yukon. Medical Assistance in Dying (MAiD) Info Sheet. 2017. 12 p. Available from: https://yukon.ca/sites/yukon.ca/files/hss/hss-medical-assistance-dying-info-sheet.pdf. (accessed 12 Mar 2026).
174. Éducaloi. Medical Aid in Dying: General Rules. 2025. Available from: https://educaloi.qc.ca/en/capsules/medical-aid-in-dying/. (accessed 31 Mar 2025).

175. Government of Québec. Procedure For Making A Request for Medical Assistance in Dying. 2022. Available from: https://www.quebec.ca/en/health/health-system-and-services/end-of-life-care/medical-aid-in-dying/procedure. (accessed 31 Mar 2025).
176. Alberta Health Services. Patient Concerns & Feedback. 2026. Available from: https://www.albertahealthservices.ca/about/patientfeedback.aspx. (accessed 12 Jan 2026).
177. Fraser Health. Talking to Children About Medical Assistance in Dying. 2020. 2 p. Available from: https://patienteduc.fraserhealth.ca/file/talking-to-children-about-medical-assistance-in-dyi-459752.pdf. (accessed 12 Jan 2026).
178. Interior Health. Submit Patient Care Feedback. 2026. Available from: https://www.interiorhealth.ca/information-for/patients-and-visitors/patient-care-quality-office/submit-patient-care-feedback. (accessed 12 Jan 2026).
179. Island Health. Listening to You. 2026. Available from: https://www.islandhealth.ca/about-us/accountability/quality-care/listening-you. (accessed 12 Jan 2026).
180. Northern Health. Patient Care Quality Office. 2026. Available from: https://www.northernhealth.ca/contact/patient-care-quality-office. (accessed 12 Jan 2026).
181. Vancouver Coastal Health. Assisted Dying Program Quality Improvement for Patient. 2017. [Available from: https://surveys.vch.ca/Survey.aspx?s=a651dd821cb74c22a5f489aefac2b22b&_gl=1*hvs8yn*_gcl_au*MjA2MDI3MDUwMi4xNzE1ODc3OTg4*_ga*ODA0MTU2MDUwLjE3MTU4Nzc5ODk.*_ga_TM76Z5SCLW*MTcxNTg3Nzk4OC4xLjEuMTcxNTg3ODEyNi4wLjAuMA. (accessed 12 Jan 2026).
182. Provincial Health Services Authority. Care Quality Compliments and Complaints. 2026. Available from: http://www.phsa.ca/about/patient-experience-quality-of-care/patient-experience/compliments-complaints. (accessed 12 Jan 2026).
183. Shared Health Manitoba. Medical Assistance in Dying (MAiD). 2026. Available from: https://sharedhealthmb.ca/services/maid/. (accessed 12 Jan 2026).
184. Horizon Health Network. Patient Relations Department. 2026. Available from: https://horizonnb.ca/patients-visitors/patient-representative-services/. (accessed 12 Jan 2026).
185. Vitalité Health Network. Comments, Concerns or Complaints. 2025. Available from: https://vitalitenb.ca/en/health-network/quality-and-safety/comments-concerns-or-complaints. (accessed 12 Jan 2026).
186. Eastern Health. Compliments and Complaints. 2021. Available from: https://www.easternhealth.ca/prc/your-say/compliments-and-complaints/. (accessed 12 Jan 2026).
187. Government of Newfoundland and Labrador. Regional Health Authorities - Client Relations. 2025. Available from: https://www.gov.nl.ca/hcs/regional-health-authorities-client-relations/. (accessed 12 Jan 2026).
188. NL Health Services. Patient Feedback. 2025. Available from: https://nlhealthservices.ca/share-feedback-about-your-care-patient-relations/. (accessed 12 Jan 2026).
189. Central Health. Medical Assistance in Dying (MAiD). 2024. Available from: https://www.centralhealth.nl.ca/medical-assistance-in-dying. (accessed 31 Mar 2025).
190. Government of Northwest Territories. Medical Assistance in Dying (MAID). 2023. Available from: https://www.hss.gov.nt.ca/en/services/medical-assistance-dying-maid. (accessed 12 Jan 2026).
191. Government of Yukon. Give Feedback About Continuing Care Services. 2026. Available from: https://yukon.ca/en/your-government/contact-and-follow-government/give-feedback-about-continuing-care-services. (accessed 12 Jan 2026).
192. Ontario Ministry of Health. Medical Assistance in Dying: General Information. 2023. 6 p. Available from: https://files.ontario.ca/moh-maid-general-information-en-2023-07-19.pdf. (accessed 12 Jan 2026).
193. Ontario Health atHome. Feedback, Complaints and Appeals: Information for Patients and Families. 2024. Available from: https://ontariohealthathome.ca/wp-content/uploads/2024/07/ESC-Feedback-Complaints-Appeals-home-and-community-care-EN.pdf. (accessed 12 Jan 2026).
194. The Ottawa Hospital. Patient Relations. 2025. Available from: https://www.ottawahospital.on.ca/en/patients-visitors/patient-relations/. (accessed 12 Jan 2026).
195. Peterborough Regional Health Centre. Patient Relations. 2024. Available from: https://www.prhc.on.ca/patient-relations/. (accessed 12 Jan 2026).

196. Sinai Health. Patient Feedback. 2026. Available from: https://www.sinaihealth.ca/patients-and-visitors/patient-feedback. (accessed 12 Jan 2026).
197. Grand River Hospital. Patient relations and feedback. 2025. Available from: https://www.grhosp.on.ca/care/visitors/compliments-and-concerns. (accessed 31 Mar 2025).
198. Hamilton Family Health Team. Patient Feedback. 2025. Available from: https://hamiltonfht.ca/patient-experience-survey/. (accessed 12 Jan 2026).
199. Home and Community Care Support Services. North East: Information and Referral. 2024. Available from: https://healthcareathome.ca/region/north-east/. (accessed 31 Mar 2025).
200. Centre intégré universitaire de santé et de services sociaux de la Capitale-Nationale. Commentaires, Plaintes. 2026.Available from: https://www.ciusss-capitalenationale.gouv.qc.ca/services/commentaires-et-plaintes. (accessed 12 Jan 2026).
201. Centre hospitalier de l'Université de Montréal. Commissaire locale aux plaintes et à la qualité des services. 2026. Available from: https://www.chumontreal.qc.ca/repertoire/commissaire-locale-aux-plaintes-qualite-services (accessed 12 Jan 2026).
202. McGill University Health Centre. Contact Us. 2026. Available from: https://muhc.ca/patients-committee/contact. (accessed 12 Jan 2026).
